# Electrochemical Umpolung C–H Functionalization of Oxindoles

**DOI:** 10.1021/acs.joc.1c02616

**Published:** 2021-12-28

**Authors:** Miryam Pastor, Marie Vayer, Harald Weinstabl, Nuno Maulide

**Affiliations:** † Christian Doppler Laboratory for Entropy-Oriented Drug Design, Institute of Organic Chemistry, University of Vienna, Währinger Strasse 38, 1090 Vienna, Austria; ‡ Boehringer-Ingelheim RCV, Doktor-Boehringer-Gasse 5-11, 1120 Vienna, Austria

## Abstract

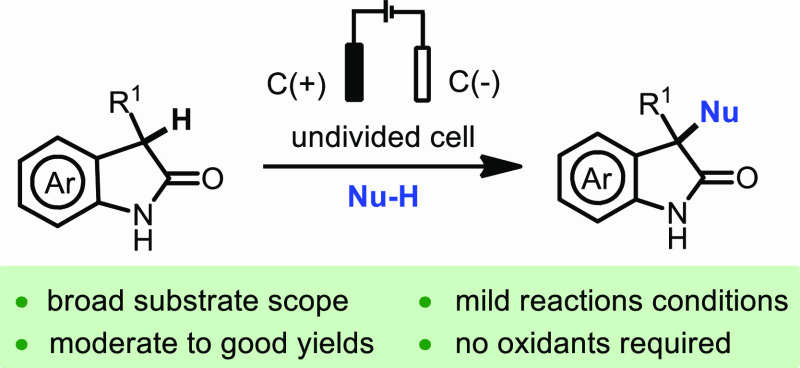

Herein, we present a general electrochemical method to access unsymmetrical 3,3-disubstituted oxindoles by direct C–H functionalization where the oxindole fragment behaves as an electrophile. This Umpolung approach does not rely on stoichiometric oxidants and proceeds under mild, environmentally benign conditions. Importantly, it enables the functionalization of these scaffolds through C–O, and by extension to C–C or even C–N bond formation.

## Introduction

**Scheme 1:**
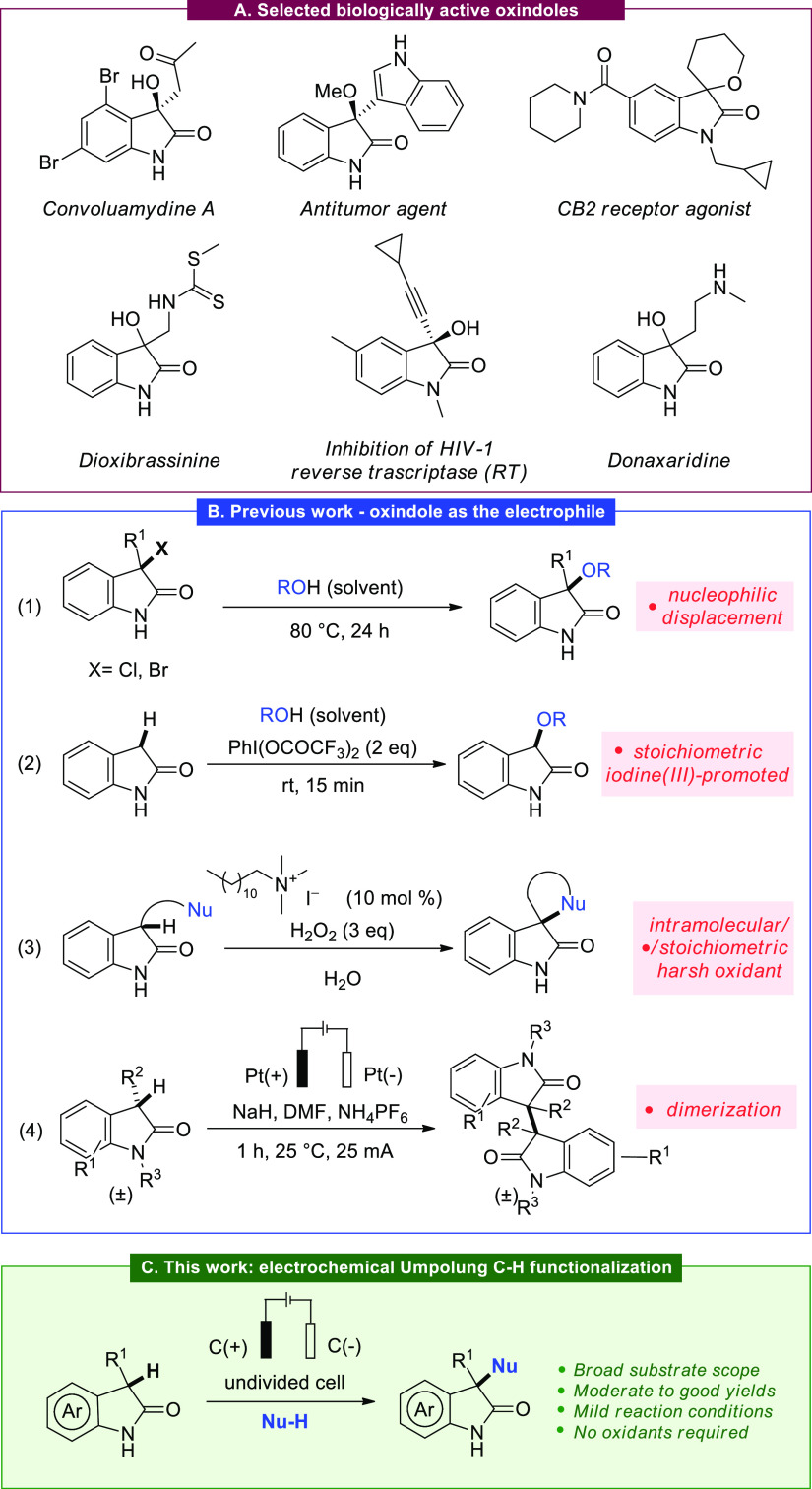
Context and Strategy for the Direct C–H Functionalization of Oxindoles

3-Oxa and 3-hydroxy-2-oxindoles constitute privileged classes of aromatic alkaloids that are encountered in numerous natural products and pharmaceuticals.^1^ This is particularly true for 3,3-disubstituted oxindole derivatives, which possess a documented broad range of biological and pharmacological activities that are intrinsically tied to that structural feature (Scheme 1A).^2^ For Convoluamydine A, a naturally occurring example, the biological activity mostly results from substitution at C-3.^3^

Given the valuable properties of these structures, significant effort has been devoted to the development of synthetic methods to access 3-oxygenated 2-oxindoles.^2b,2c^ While manifold methods for the synthesis of hydroxy derivatives exist,^4^ there is a dearth of methods to directly access the 3 alkoxy congeners.^5^ Recently, Liu and Zhou described an efficient thermal substitution of 3-halooxindoles (Scheme 1B, eq 1),^6^ relying on the *in situ* formation of a dearomatized Michael acceptor as an intermediate, followed by an S_N_1 reaction using various alcohols.

The direct functionalization of unsubstituted, “naked” oxindoles represents an attractive approach to afford such motifs. Relevant transformations of the C-3 position, invariably employing the oxindole fragment as a nucleophile, include arylation,^7^ alkynylation,^8^ alkylation,^9^ fluorination,^10^ trifluoromethylation,^11^ nitration,^12^ azidation,^13^ amination,^14^ and thiolation.^15^ When it comes to its use as an electrophilic synthon, Kotagiri has described the stoichiometric use of an hypervalent iodine reagent [PhI(OCOCF_3_)_2_] for the oxidative alkoxylation of oxindoles (Scheme 1B, eq 2),^16^ and more recently, the oxidative intramolecular α-oxygenation and α-amination of oxindoles was reported by Zhong, employing a micellar catalytic system based on amphiphilic bifunctional iodide salts in water (Scheme 1B, eq 3),^17^ featuring H_2_O_2_ as the terminal oxidant. Few methods were also reported for the direct CH-functionalization of oxindoles using electrochemistry.^18^ In particular, a wide range of dimeric 2-oxindoles were recently prepared by an oxidative C–C coupling reaction (Scheme 1B, eq 4).^18b^

**Table 1:**
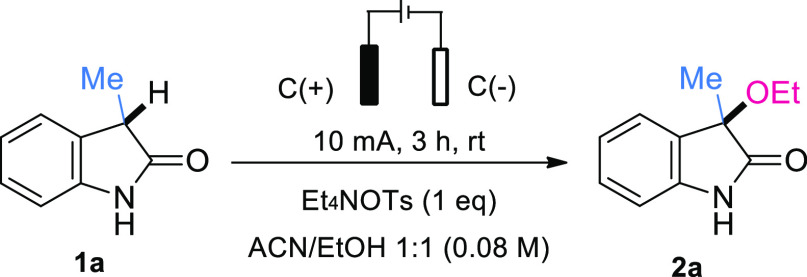
Optimization of Reaction Conditions^a^

entry	deviation from above	yield^b^ (%)
1	Pt cathode	40
2	none	57 (72% brsm)
3	*n*Bu_4_NOTs instead of Et_4_NOTs	50
4	*n*Bu_4_NPF_6_ instead of Et_4_NOTs	39
5	*n*Bu_4_NClO_4_ instead of Et_4_NOTs	22
6	AcOH (1 equiv.) as an additive	46
7	AgPF_6_ (1 equiv.) as an additive	32
8	maintained at 0–10 °C	43
9	3 mA, 12 h instead of 10 mA, 3 h	29
10	15 mA instead of 10 mA	10
11	constant potential of 1.8 V for 28 h	53
12	3 Å MS	37
13	no electricity	NR^c^

a Standard conditions: undivided cell, C-SK50 anode and cathode constant current = 10 mA, **1a** (0.4 mmol), Et_4_NOTs (1.0 equiv), ACN/EtOH 1:1 (0.08 M), rt, 3 h.

b Isolated yield.

c NR = no reaction.

As part of a research program focused on novel approaches to design drugs and given our interest in the development of umpoled synthons, we became interested in the sustainable preparation of 3,3-disubstituted oxindole derivatives, envisaging electrochemistry as a powerful tool to tackle this problem. The appeal of electrosynthesis lies mainly on its eco-friendly nature and generally mild reaction conditions, therefore, unsurprisingly, its use has gained significant traction in recent years.^19^

## Results and Discussion

Initial studies of the redox behavior of 3-methylindolin-2-one **1a** using cyclic voltammetry (CV) revealed an irreversible anodic oxidation peak at 1.8 V (see Supporting Information, Figure S1). This immediately hinted at the possibility of using electrochemistry for the direct C(sp^3^)–H functionalization of 2-oxindoles and related compounds.^20^ Herein, we report the synthesis of unsymmetrical 3,3-disubstituted oxindoles by direct electrochemical Umpolung C–H functionalization.

We commenced our search for optimal reaction conditions using **1a** and relying on a simple undivided cell setup based on the ElectraSyn 2.0 package with a graphite (C) anode and a platinum (Pt) cathode (Table 1). These electrodes were initially used under a constant current of 10 mA, in the presence of tetraethylammonium *p*-toluenesulfonate (Et_4_NOTs) as the electrolyte in a mixture of MeCN/EtOH. Under these initial conditions, an ethoxylated product **2a** was directly produced in 40% yield (entry 1). Replacing the platinum (Pt) cathode by an inexpensive graphite (C) cathode had a positive impact on the reaction outcome, yielding **2a** in 57% or 72% based on recovered **1a** (entry 2). On the other hand, the use of different electrolytes led to a decreased yield (entries 5–7 and see Supporting Information, Table S2). Neither the addition of stoichiometric acids (entry 6) nor the use of different silver salts as sacrificial oxidants (entry 7 and see Supporting Information, Table S3) delivered improved results. Various well-established electrochemical mediators were also tested, but to no avail (see Supporting Information, Table S3).^21^

Lowering the temperature of the reaction, hoping to prevent potential deleterious decomposition of the precursor, proved detrimental to the outcome, as did changing the intensity of the current or the reaction time (entries 8–10). Working at a constant potential of 1.8 V afforded the product in 53% yield without recovering of the starting material (entry 11). A control experiment in the absence of electricity led to no product being detected (entry 13). It is noteworthy that the cyclic voltammogram of **2a** showed an oxidation peak at 2.0 V, very close to that observed for **1a**. In agreement with this, achieving full conversion of **1a** without notable decomposition of product **2a** proved unattainable.^22^

**Scheme 2:**
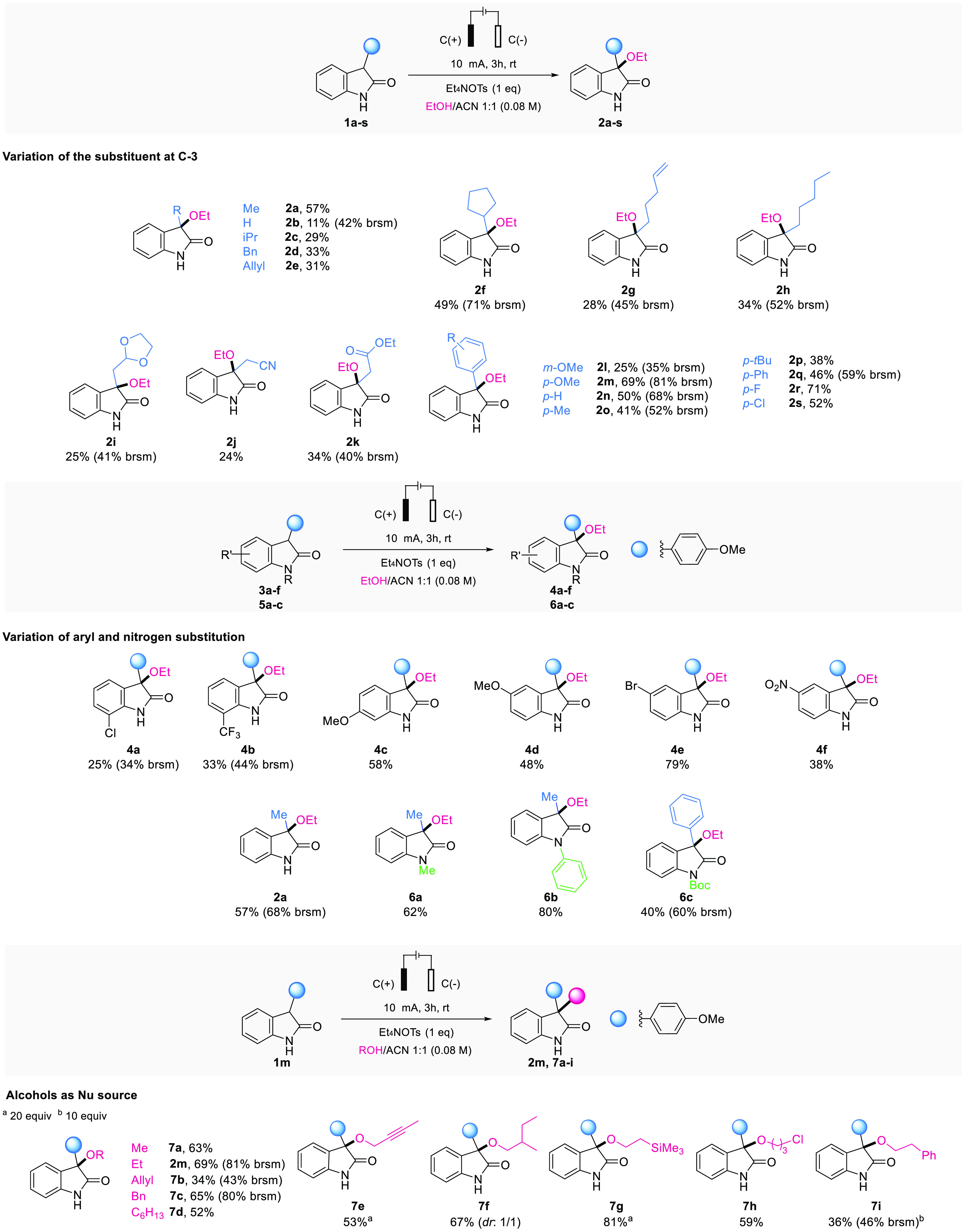
Scope of the Reaction

With the optimized reaction conditions in hand, the scope of this transformation was explored, as illustrated in Scheme 2. At the onset, the tolerance of various substituents at the C-3 position of the oxindole core was evaluated using EtOH as the nucleophile. Alkyl-substituted oxindoles were amenable to this reaction, delivering products **2a–h**. Diverse functional groups, including an acetonide (**2i**), a nitrile (**2j**), and an ester (**2k**) were compatible with the reaction conditions and we observed that aryl substitution led to a slight increase of the yields (**2i–s**). Further functional-group modifications on the aromatic portion of the oxindole were tolerated under the standard conditions, notably including halides (**4a–b**, **4e**), methoxy (**4c**, **4d**), and nitro (**4f**) groups. In addition, the reaction is not limited to unprotected oxindoles but can be expanded to include alkyl- and aryl-substituted nitrogen atoms (**6a–b**), as well as an acid-labile carbamate protecting group (**6c**).

The ability to use either unprotected (NH) or diversely N-substituted oxindoles is a special feature of this method. In addition, a range of aliphatic alcohol nucleophiles was employed, affording products **7** in good to high yields. In particular, benzylic (**7c**) and propargylic (**7e**) alcohols were well tolerated, as well as alcohols bearing silyl (**7g**) and halide (**7h**) groups. It is worth mentioning that for alcohols of higher molecular weight, the amount of nucleophile could be reduced to 10–20 equivalents without any significant drop in the yield.

It should be noted that conditions were not reoptimized for each product **2**–**4**–**7**; this is reflected in the broad yield range observed. We believe that the versatility of the process and the unique character of this oxidative transformation are bound to prove very useful to the synthetic practitioner.

**Scheme 3:**
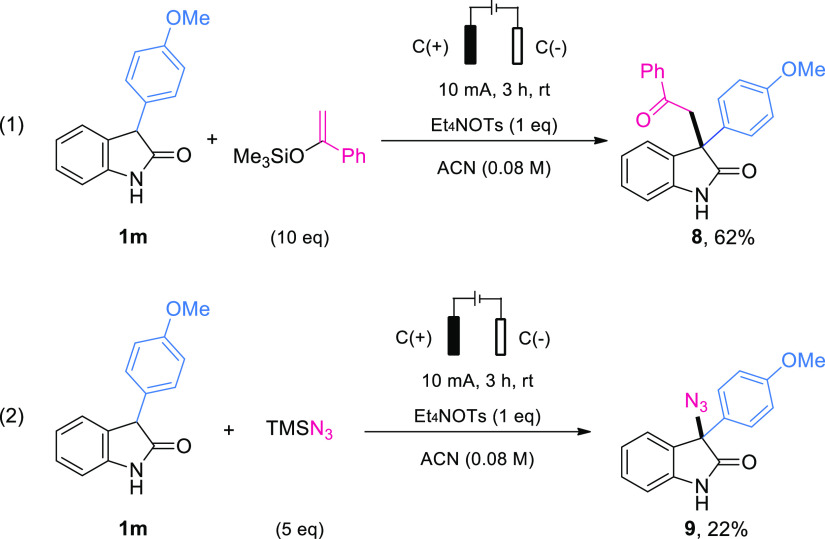
Extension to a Silyl Enol Ether and TMSN_3_ as Nucleophiles

Finally, extension of this electrochemical transformation to the formation of C–C bonds and C–N bonds was also investigated (Scheme 3). Gratifyingly, C–C bond formation was well within reach of the reaction, proceeding when a silyl enol ether was used as the nucleophile, affording the desired product **8** in a 62% yield.^23^ It was also possible to carry out an azidation reaction, leading to **9**. Despite its low yield, this is an appealing direct C–N bond formation.

**Scheme 4:**
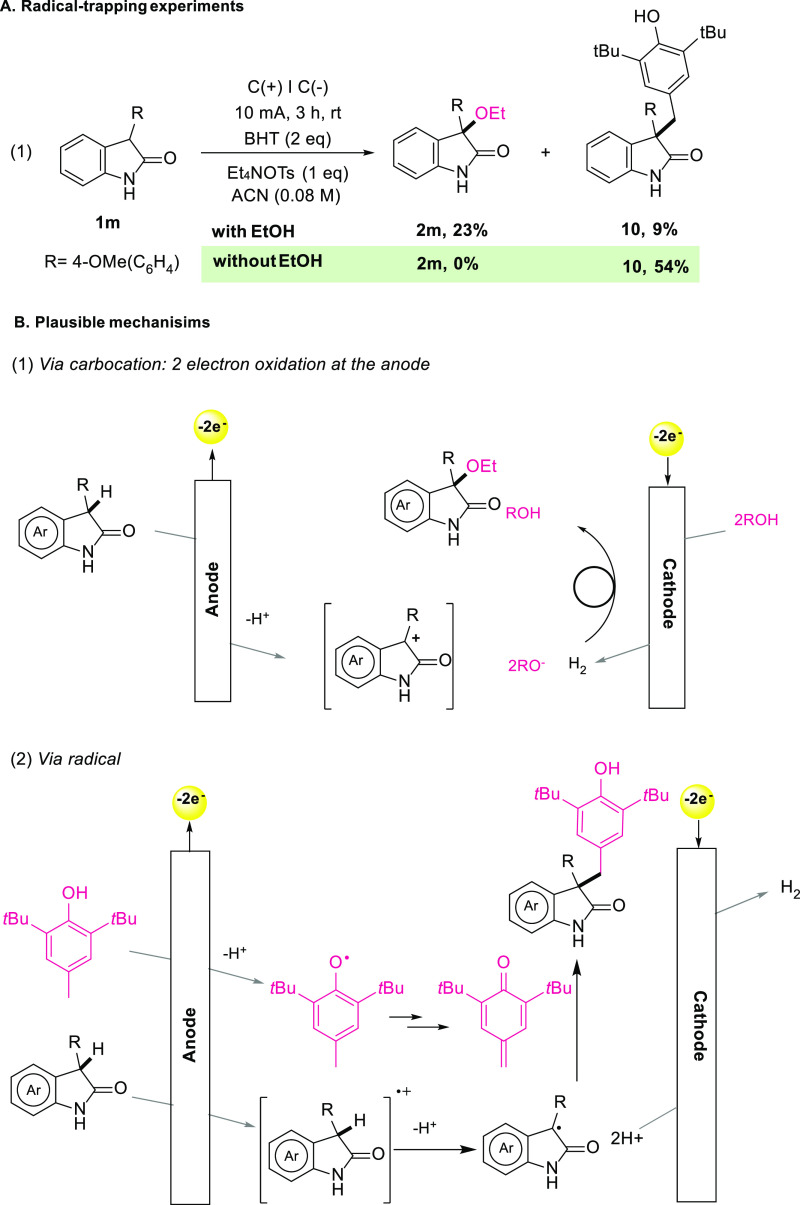
Mechanistic Experiments and Plausible Mechanism

To gain further insights into the reaction mechanism, several control experiments were conducted (Scheme 4A). In the presence of a radical scavenger such as 2,2,6,6-tetramethylpiperidine-1-oxyl (TEMPO), no product was formed, although all the starting material was consumed. In the presence of butylated hydroxytoluene (BHT), however, **2m** was obtained in a low yield alongside the coupling product **10** (9%; obtained in 54% in the absence of ethanol) [Scheme 4A(1)]. These results suggest the possibility of a radical mechanism being involved in our transformation.^24^

Based on the above and previous reports,^25^ we proposed two mechanistic scenarios depending on the nucleophilic source used (Scheme 4B). One possibility is that the substrates undergo a two-electron oxidation at the anode, forming the corresponding carbocation which can be subsequently trapped [Scheme 4B(1)]. This could explain the need for excess amounts of the nucleophile (in some cases used as a co-solvent), justified due to its role of acting as a proton source for hydrogen evolution at the cathode. We hypothesize that depending on the nucleophile and its oxidation potential, a second possible pathway might become available: single-electron oxidation at the anode leading to formation of a radical cation followed by a loss of a proton would generate a captodative radical intermediate [Scheme 4B(2)]. This radical’s competence for C–C bond formation is showcased by the products formed (*vide supra*) when even comparably small amounts of BHT are employed—whereby we surmise the transient formation of a BHT-derived *p*-quinonemethide.

## Conclusions

In conclusion, we have developed a general electrochemical method to access unsymmetrical 3,3-disubstituted oxindoles by direct C–H functionalization. This approach does not rely on stoichiometric oxidants and proceeds under mild, environmentally benign conditions. Importantly, it enables the functionalization of these scaffolds through C–O, and by extension to C–C or even C–N bond formation.

## Experimental Section

### General Procedure to Access 3,3-Disubstituted Oxindoles

With no precautions to exclude air or moisture, the ElectraSyn vial (10 mL) was charged with 3-susbtituted indolin-2-one **1a–s**, **3a–f** or **5a–c** (0.40 mmol, 1.0 equiv), Et4NOTs (121.0 mg, 0.40 mmol, 1.0 equiv), ROH (2.5 mL), and MeCN (2.5 mL). The ElectraSyn vial cap equipped with the anode (graphite) and cathode (graphite) was inserted into the mixture. The reaction mixture was electrolyzed at a constant current of 10 mA for 3 h. The ElectraSyn vial cap was removed, and electrodes were rinsed with DCM (2.0 mL), which was combined with the crude mixture. Then, the crude mixture was concentrated under reduced pressure and purified by FC over silica gel (heptane/ethyl acetate, 100/0 to 50/50, gradient) to furnish the desired products **2a–s**, **4a–f**, **6a–c** or **7a–i**.

We do not possess any electrochemical devices which would allow for running these reactions in a scale larger than that reported herein.

## Supporting Information Available

Additional optimization tables, experimental procedures, ^1^H and ^13^C NMR spectra, and characterization data of compounds (PDF)
