# Supplementary material for: Electrochemical Umpolung C–H Functionalization of Oxindoles
Source: J Org Chem. 2021 Dec 28;87(1):606–12. doi: 10.1021/acs.joc.1c02616 (PMC8749966; doi:10.1021/acs.joc.1c02616)
Supplement: Supplementary file 1 — jo1c02616_si_001.pdf [file jo1c02616_si_001.pdf]

Electrochemical Umpolung C–H Functionalization of Oxindoles

Supporting Information

Miryam Pastor,[†, ‡] Marie Vayer,[†, ‡] Harald Weinstabl[§] and Nuno Maulide*[,†]

[†] *Christian Doppler Laboratory for Entropy-Oriented Drug Design, Institute of Organic Chemistry, University of Vienna, Währinger Strasse 38, 1090 Vienna, Austria and* [§] *Doktor-Boehringer-Gasse 5-11, 1120 Vienna, Austria.*

[‡] These authors contributed equally.

nuno.maulide@univie.ac.at

Table of Contents

1. GENERAL REMARKS S2

2. CYCLIC VOLTAMMETRY S3

3. OPTIMIZATION OF REACTION PARAMETERS FOR ELECTROCHEMICAL FORMATION OF 3,3-DISUSBTITUTED OXINDOLES S4

4. GENERAL PROCEDURE FOR ELECTROCHEMICAL FORMATION OF 3,3-DISUSBTITUTED OXINDOLES S6

5. EXPERIMENTAL PROCEDURES AND CHARACTERIZATION DATA OF THE STARTING MATERIALS 1C-S, 3A-F AND 5A-M S6

6. CHARACTERIZATION DATA OF 3,3-SUBSTITUTED OXINDOLES 2A-S, 4A-F, 6A-C AND 7A-I S25

7. PROCEDURES AND CHARACTERIZATION DATA OF 3,3-SUBSTITUTED OXINDOLES 8 AND 9 (SCHEME 3) S43

8. ADDITIONAL EXPERIMENTS WITH DIFFERENT TYPE OF ALKENES S45

9. BHT TRAPPING EXPERIMENT (SCHEME 4) S46

10. NMR SPECTRA S48

11. REFERENCES S116

1. General Remarks

All solvents were distilled from appropriate drying agents prior to use or directly taken from commercial sealed bottles under an atmosphere of argon. All reagents were used as received from commercial suppliers (*Alfa Aesar*, *Sigma Aldrich* or *TCI*) unless otherwise stated. Reaction progress was monitored by thin layer chromatography (TLC) performed on aluminum plates coated with silica gel F_{254} with 0.2 mm thickness. Chromatograms were visualized by fluorescence quenching with UV light at 254 nm and/or by staining using vanillin. Flash column chromatography was performed using silica gel 60 (230-400 mesh, Merck and co.). Yields refer to chromatographically and spectroscopically pure compounds. ^{1}H NMR, ^{13}C NMR and ^{19}F NMR spectra were recorded using a Bruker AV-400 and AV-600 spectrometer at 300 K. ^{1}H NMR chemical shifts are reported in ppm using residual solvent peak as reference ($CDCl_3$: δ = 7.26 ppm or DMSO-d_6: δ = 2.50 ppm). Data for ^{1}H NMR are presented as follows: chemical shift δ (ppm), multiplicity (s = singlet, d = doublet, t = triplet, m = multiplet, br = broad), coupling constant *J* (Hz) and integration; ^{13}C NMR spectra were recorded at 100 or 150 MHz using broadband proton decoupling and chemical shifts are reported in ppm using residual solvent peaks as reference ($CDCl_3$: δ = 77.16 ppm or DMSO-d_6: δ = 39.52 ppm). Multiplicity was defined by recorded a ^{13}C NMR spectra using the attached proton test (APT). Neat infra-red spectra were recorded using a Brucker Vertex 70 FT-IR spectrometer. Wavenumbers are reported in cm^{-1}. Mass spectra were obtained using a Finnigan MAT 8200 or (70 eV) or an Agilent 5973 (70 eV) spectrometer, using electrospray ionization (ESI) and a maXis UHR-TOF analyzer.

Electrolysis general information

Electrochemical reactions were performed with ElectraSyn 2.0 package (IKA) using the constant current mode. The reactions were conducted in a 10 mL vial with a magnetic stir bar and a graphite-SK-50 (5.0 x 0.8 x 0.2 cm) working electrode and counter-electrode with a distance of 0.6 cm between the two electrodes.

2. Cyclic Voltammetry

Cyclic voltammetry was recorded with Graphite-SK-50 (5.0 x 0.8 x 0.2 cm) working electrode, platinum counter-electrode and aqueous Ag/AgCl reference electrode. Scan rate: 200 mV/s.

Conditions: 0.1 M Et_4NOTs in MeCN as solvent.

Cyclic voltammogram of **1a** showing an oxidation potential of 1.8 V.

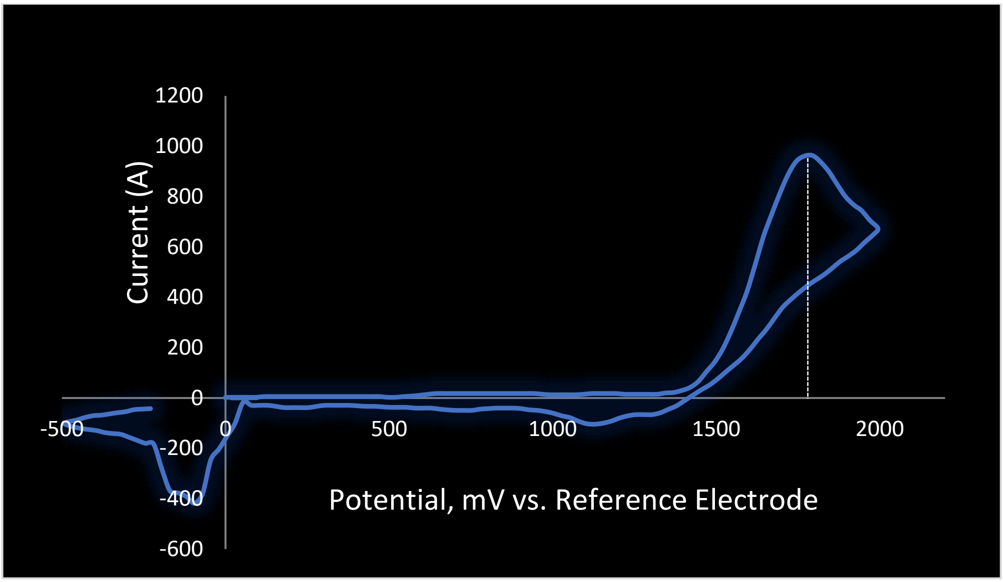

Figure S1. Cyclic voltammogram of **1a**

Cyclic voltammogram of **2a** showing an oxidation potential of 2.0 V.

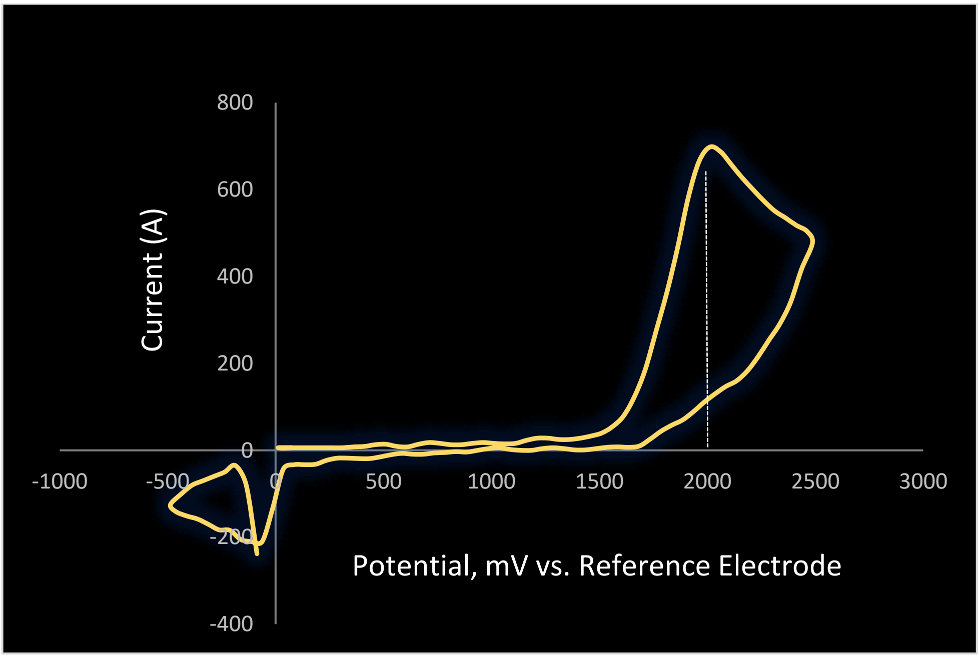

Figure S2. Cyclic voltammogram of **2a**

3. Optimization of Reaction Parameters for Electrochemical Formation of 3,3-Disusbtituted Oxindoles

All optimization reactions were carried out on 0.40 mmol scale. The crude reaction mixture was purified by FC (10 g SiO_2, heptane/ethyl acetate: 100/0 to 70/30, 35 CV).

Primary Evaluation of solvents (**Table S1**)

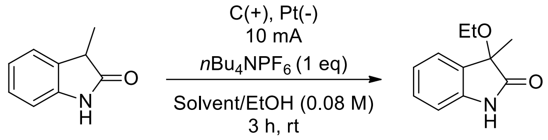

Entry	Solvent	Yield [%][a]
1	THF	37
2	ACN	40
3	DMF	ND
4	DCM	30

[a] Isolated yield.

Evaluation of electrolytes (**Table S2**)

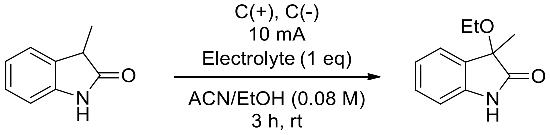

Entry	Electrolyte	Yield [%][a]
1	Et_4NOTs	57 (72%brsm)
2	*n*Bu_4NOTs	50
3	*n*Bu_4NPF_6	40
4	*n*Bu_4NClO_4	22
5	*n*Bu_4NOAc	ND
6	*n*Bu_4NBr	10
7	PPTS	47
8	$NaPF_6$	39
9	$AgPF_6$	27
10	KBF_4	ND

[a] Isolated yield.

Evaluation of additives (**Table S3**)

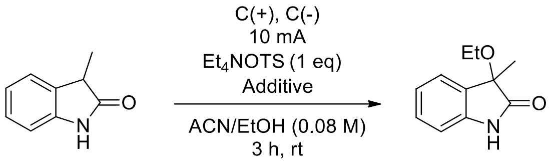

Entry	Additive	Yield [%][a]
1	ABNO (10 mol%)	52
2	KetoABNO (10 mol%)	ND
3	Quinuclidine (20 mol%)	43
4	DDQ (20 mol%)	17
5	DABCO (20 mol%)	35
6	N-Hydroxytetrachlorophthalimide (10 mol%)	52
7	HOBt (10 mol%)	43
8	2,4,5-Triphenylimidazole (10 mol%)	ND
9	$AgPF_6$ (1 eq)	32
10	AgOTf (1 eq)	41
11	$AgNTf_2$ (1 eq)	38
12	$AgClO_4$ (1 eq)	37
13	$AgSbF_6$ (1 eq)	45
14	$AgBF_4$ (1 eq)	48
15	TFA (0.5 eq)	33
16	AcOH (0.5 eq)	35
17	AcOH (1 eq)	46
18	K_2CO_3 (1 eq)	23
19	2,6-Lutidine (1 eq)	22

[a] Isolated yield.

4. General Procedure for Electrochemical Formation of 3,3-Disusbtituted Oxindoles

General procedure (A) to access 3,3-disusbtituted oxindoles

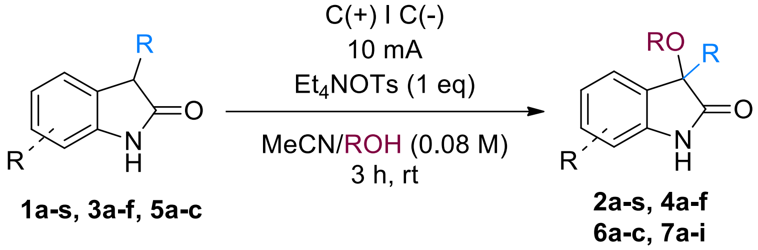

With no precautions to exclude air or moisture, the ElectraSyn vial (10 mL) was charged with 3-susbtituted indolin-2-one **1a-s, 3a-f** or **5a-c** (0.40 mmol, 1.0 equiv.), Et_4NOTs (121.0 mg, 0.40 mmol, 1.0 equiv.), ROH (2.5 mL) and MeCN (2.5 mL). The ElectraSyn vial cap equipped with anode (graphite) and cathode (graphite) were inserted into the mixture. The reaction mixture was electrolyzed at a constant current of 10 mA for 3 h. The ElectraSyn vial cap was removed, and electrodes were rinsed with DCM (2.0 mL), which was combined with the crude mixture. Then, the crude mixture was concentrated under reduced pressure and purified by FC over silica gel (heptane/ethyl acetate, 100/0 to 50/50, gradient) to furnish the desired products **2a-s, 4a-f, 6a-c** or **7a-i**.

5. Experimental Procedures and Characterization Data of the Starting Materials 1c-s, 3a-f and 5a-m

General procedure (B) to access 3-hydroxysusbtituted oxindoles (**S1l-s, S3a-f, S5b**)

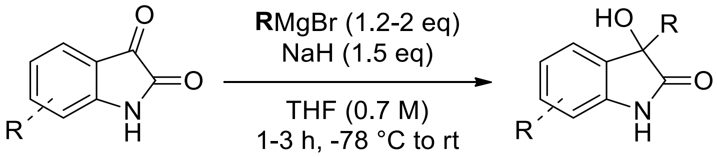

To a solution of isatin derivate (1.0 equiv.) in THF (0.7 M), NaH (1.5 equiv.) was added at -78 °C. The mixture was stirred for 30 min. Then, the Grignard reagent (1.2-2.0 equiv., commercially available or previously prepared) was added dropwise at this temperature. The reaction was warmed up to rt and stirred for 3 h. The mixture was quenched with NH_4Cl, extracted with ethyl acetate, washed with H_2O, dried over $MgSO_4$ and concentrated in vacuo. DCM was added to the solids obtained. Solids were filtered off and the resulting filtrate was dried in vacuo to afford the desired substrate **S1l-s**, **S3a-f** and **S5b**.

General procedure (C) to access 3-susbtituted indolin-2-one (**1l-s, 3a-f, 5b**)

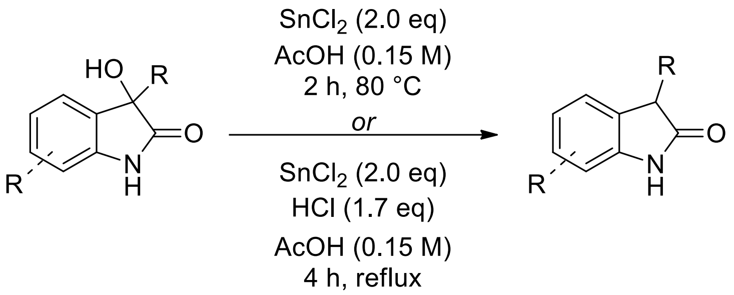

Following a modified reported procedure,[1] 3-hydroxysubstituted oxindole (1.0 equiv.) was dissolved in acetic acid (0.15 M) and $SnCl_2$ (2.0 equiv.) was added in one portion. The reaction mixture was stirred at 80 °C in an oil bath for 2 h and then cooled to rt and concentrated in vacuo. The product was extracted with ethyl acetate and the organic layer was washed with $NaHCO_3$, dried over $MgSO_4$, and then concentrated to afford **1l-s**, **3a-f** and **5b**. Compounds where purified by FC over silica gel (heptane/ethyl acetate, 100/0 to 70/30, gradient) to furnish the desired products.

3-Isopropylindolin-2-one **1c**

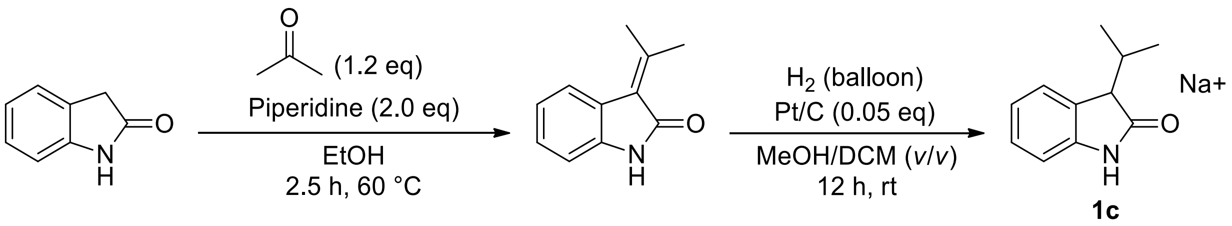

To a solution of oxindole (2.0 g, 15.0 mmol, 1.0 equiv.) in EtOH (15.0 mL, 0.10 M) were added acetone (1.34 mL, 18.0 mmol, 1.2 equiv.) and piperidine (2.97 mL, 30.0 mmol, 2.0 equiv.). The reaction was then heated at reflux in an oil bath for 2.5 h. After cooling to 0 °C, the orange-colored precipitate formed was filtered, washed with cold EtOH and dried under vacuum. 3-(Propan-2-ylidene)indolin-2-one is obtained as a yellow solid (1.3 g, 7.51 mmol, 50%) and used in next step without further purification.

3-(Propan-2-ylidene)indolin-2-one (1.30 g, 7.51 mmol, 1.0 equiv.) was dissolved in a mixture of MeOH/DCM (50 mL, *v*/*v*). Pt/C (73.2 mg, 0.380 mmol, 0.05 equiv.) was added to this solution, and the resulting mixture was stirred under hydrogen atmosphere (balloon) for 12 h at rt. The reaction mixture was then passed through celite, washed with DCM and concentrated in vacuo to afford **1c** as a pale-yellow solid (725 mg, 4.14 mmol, 55%).

^{1}H NMR (600 MHz, $CDCl_3$): δ 8.83 (br s, 1H), 7.25 (d, J = 7.2 Hz, 1H), 7.21 (t, J = 7.7 Hz, 1H), 7.01 (td, J = 7.7, 0.7 Hz, 1H), 6.90 (d, J = 7.7 Hz, 1H), 3.40 (d, J = 3.6 Hz, 1H), 2.51 (dtd, J = 13.8, 6.8, 3.6 Hz, 1H), 1.13 (d, J = 6.8 Hz, 3H), 0.92 (d, J = 6.8 Hz, 3H). **^{13}C{^{1}H} NMR (150 MHz, $CDCl_3$):** δ 180.2 (C=O), 142.2 (C), 128.5 (C), 127.9 (CH), 124.8 (CH), 122.2 (CH), 109.7 (CH), 52.3 (CH), 30.9 (CH), 20.0 (CH_3), 18.1 (CH_3). **HRMS (ESI$^+$):** *m/z* calcd. for $C_{11}H_{13}NONa$ [M+Na]$^+$ 198.0889, found 198.0890. Spectral and physical data are in accordance with literature.[2]

***3-Benzylindolin-2-one* 1d**

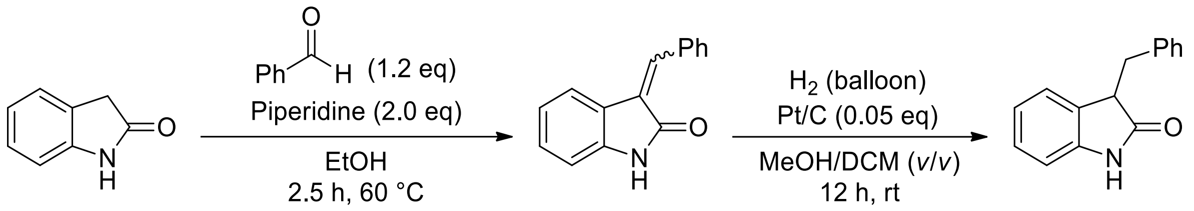

To a solution of oxindole (500 mg, 3.76 mmol, 1.0 equiv.) in EtOH (4.0 mL, 0.10 M) were added benzaldehyde (0.458 mL, 4.51 mmol, 1.2 equiv.) and piperidine (0.742 mL, 7.51 mmol, 2.0 equiv.). The reaction was then heated to reflux in an oil bath for 2.5 h. After cooling to 0 °C, the orange-colored precipitate formed was filtered, washed with cold EtOH and dried under vacuum. 3-Benzylideneindolin-2-one is obtained as a yellow solid (415 mg, 1.88 mmol, 50%) and used in next step without further purification.

Benzylideneindolin-2-one (415 g, 1.88 mmol, 1.0 equiv.) was dissolved in a mixture of MeOH/DCM (16 mL, *v*/*v*). Pt/C (18.0 mg, 0.090 mmol, 0.05 equiv.) was added to this solution, and the resulting mixture was stirred under hydrogen atmosphere (balloon) for 12 h at rt. The reaction mixture was then filtered through celite, washed with DCM and concentrated in vacuo to afford **1d** as a pale-yellow solid (419 mg, 1.88 mmol, quant.).

^{1}H NMR (400 MHz, $CDCl_3$): δ 8.80 (brs, 1H), 7.20-7.13 (m, 3H), 7.12-7.05 (m, 3H), 6.81 (t, *J* = 7.5 Hz, 1H), 6.77 (d, *J* = 7.8 Hz, 1H), 6.66 (d, *J* = 7.5 Hz, 1H), 3.67 (dd, *J* = 9.3, 4.6 Hz, 1H), 3.42 (dd, *J* = 13.6, 4.6 Hz, 1H), 2.86 (dd, *J* = 13.6, 9.3 Hz, 1H). **^{13}C{^{1}H} NMR (100 MHz, $CDCl_3$):** δ 179.9 (C=O), 141.6 (C), 137.9 (C), 129.6 (2CH), 129.1 (C), 128.5 (2CH), 128.1 (CH), 126.8 (CH), 125.0 (CH), 122.1 (CH), 109.9 (CH), 47.7 (CH), 36.7 (CH_2). **HRMS (ESI$^+$):** *m/z* calcd. for $C_{15}H_{13}NONa$ $[M+Na]^+$ 246.0889, found 246.0890. Spectral and physical data are in accordance with literature.[2]

***3-Allylindolin-2-one* 1e**

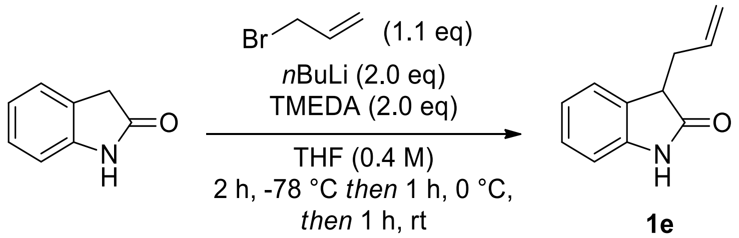

To a solution of oxindole (500 mg, 3.76 mmol, 1.0 equiv.) in THF (15.0 mL) at -78 ºC was added *n*BuLi (2.5 M in hexanes, 3.0 mL, 7.51 mmol, 2.0 equiv.) and TMEDA (1.13 mL, 7.51 mmol, 2.0 equiv.) and the resulting solution was stirred for 2 h. Allyl bromide (0.357 mL, 4.13 mmol, 1.1 equiv.) was then added dropwise and the solution was slowly warmed up to 0 ºC and stirred for 1 h at that temperature and for 1 h at rt. The reaction was then quenched with aq. NH_4Cl and extracted with ethyl acetate. The combined organic layers were washed with brine, dried over $MgSO_4$, and concentrated in vacuo. The crude was purified by FC (40 g SiO_2, heptane/ethyl acetate: 100/0 to 70/30, 20 CV) to afford **1e** as a white solid (305 mg, 1.76 mmol, 47%).

^{1}H NMR (400 MHz, CDCl$_3$): *δ* 8.85 (brs, 1H), 7.29-7.24 (m, 1H), 7.21 (t, *J* = 7.8 Hz, 1H), 7.01 (t, *J* = 7.5 Hz, 1H), 6.91 (d, *J* = 7.8 Hz, 1H), 5.92-5.68 (m, 1H), 5.13 (dd, *J* = 17.0, 1.2 Hz, 1H), 5.06 (dd, *J* = 10.1, 1.2 Hz, 1H), 3.54 (dd, *J* = 7.2, 5.2 Hz, 1H), 2.87-2.80 (m, 1H), 2.65-2.56 (m, 1H). **^{13}C{^{1}H} NMR (100 MHz, CDCl$_3$):** *δ* 180.0 (C=O), 141.7 (C), 134.0 (CH), 129.4 (C), 128.1 (CH), 124.6 (CH), 122.3 (CH), 118.2 (CH$_2$), 109.9 (CH), 45.9 (CH), 34.9 (CH$_2$). **HRMS (ESI$^+$):** *m/z* calcd. for $C_{11}H_{11}NONa$ [M+Na]$^+$ 196.0738, found 196.0733. Spectral and physical data are in accordance with literature.[3]

***3-Cyclopentylindolin-2-one* 1f**

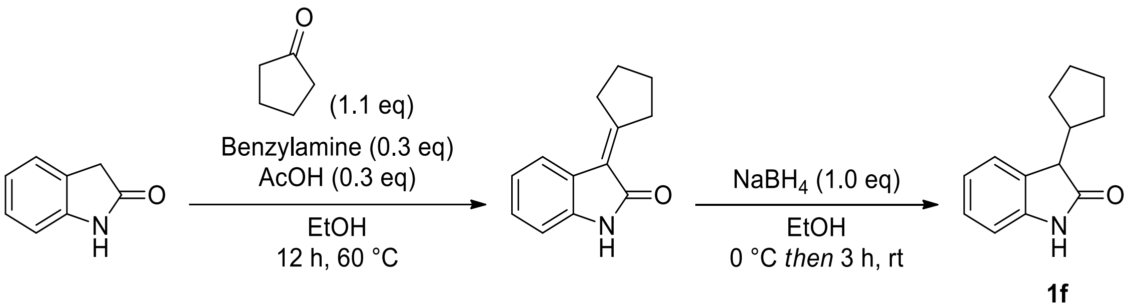

Following a procedure previously reported,[4] to a 100 mL screw-capped pressure tube was introduced oxindole (1.0 g, 7.51 mmol, 1.0 equiv.), EtOH (15.0 mL) and cyclopentanone (0.731 mL, 8.26 mmol, 1.1 equiv.). Then, benzylamine (0.246 mL, 1.0 mmol, 0.30 equiv.) and acetic acid (0.129 mL, 1.0 mmol, 0.30 equiv.) were added. The tube was sealed and was heated to 60 °C in an oil bath for 12 h. After the solvent was removed, the resulting crude mixture was dissolved in DCM and washed with H_2O. The aqueous phase was extracted with DCM, dried over $MgSO_4$ and concentrated in vacuo. 3-Cyclopentylideneindolin-2-one was obtained as a brown solid (1.50 g, 7.50 mmol, quant.) and used directly in the next step without further purification.

The crude mixture was dissolved in 20 mL of EtOH, and $NaBH_4$ (285 mg, 7.50 mmol, 1.0 equiv.) was added at 0 °C in one portion. The resulting mixture was warm up to rt and vigorously stirred for 3 h. The reaction is quenched with saturated aq. NH_4Cl, extracted with DCM, dried over $MgSO_4$ and concentrated in vacuo. The residue was purified by FC (40 g SiO_2, heptane/ethyl acetate, 100/0 to 70/30, 20 CV) to afford **1f** as a white solid (1.12 g, 5.56 mmol, 74%).

^{1}H NMR (400 MHz, CDCl$_3$): *δ* 8.23 (brs, 1H), 7.30-7.24 (m, 1H), 7.20 (t, *J* = 7.7 Hz, 1H), 7.00 (td, *J* = 7.6, 0.8 Hz, 1H), 6.87 (d, *J* = 7.8 Hz, 1H), 3.52 (d, *J* = 5.3 Hz, 1H), 2.54-2.41 (m, 1H), 1.97-1.87 (m, 1H), 1.80-1.72 (m, 1H), 1.68-1.49 (m, 5H), 1.39-1.29 (m, 1H). **^{13}C{^{1}H} NMR (100 MHz, CDCl$_3$):** *δ* 180.0 (C=O), 141.8 (C), 129.3 (C), 127.9 (CH), 124.9 (CH), 122.2 (CH), 109.6 (CH), 49.2 (CH), 42.0 (CH), 30.0 (CH$_2$), 28.5 (CH$_2$), 25.2 (CH$_2$), 25.2 (CH$_2$). **HRMS (ESI$^+$):** *m/z* calcd. for $C_{13}H_{15}NONa$ [M+Na]$^+$ 224.1046, found 224.1044. Spectral and physical data are in accordance with literature.[4]

***3-(Pent-4-en-1-yl)indolin-2-one* 1g**

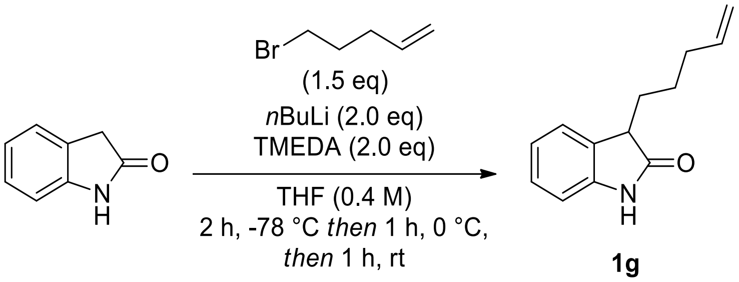

To a solution of oxindole (2.0 g, 15.0 mmol, 1.0 equiv.) in THF (60.0 mL) at -78 ºC was added *n*BuLi (2.5 M in hexanes, 12.0 mL, 30.0 mmol, 2.0 equiv.) and TMEDA (4.51 mL, 30.0 mmol, 2.0 equiv.) and the resulting solution was stirred for 2 h. 5-Bromo-1-pentene (2.67 mL, 22.5 mmol, 1.1 equiv.) was then added dropwise and the solution was slowly warmed up to 0 ºC and stirred for 1 h at that temperature and for 1 h at rt. The reaction was then quenched with aq. NH_4Cl and extracted with ethyl acetate. The combined organic layers were washed with brine, dried over $MgSO_4$, and concentrated in vacuo. The crude was purified by FC (80 g SiO_2, heptane/ethyl acetate, 100/0 to 70/30, 20 CV) to afford **1g** as an orange oil (326 mg, 1.62 mmol, 11%).

^{1}H NMR (400 MHz, $CDCl_3$): δ 8.69 (brs, 1H), 7.24-7.18 (m, 2H), 7.03 (t, J = 7.5 Hz, 1H), 6.90 (d, J = 7.6 Hz, 1H), 5.76 (ddt, J = 16.9, 10.2, 6.7 Hz, 1H), 5.07-4.89 (m, 2H), 3.48 (t, J = 6.0 Hz, 1H), 2.08 (q, J = 7.1 Hz, 2H), 1.98 (ddd, J = 16.4, 10.6, 6.0 Hz, 2H), 1.57-1.37 (m, 2H). **^{13}C{^{1}H} NMR (100 MHz, $CDCl_3$):** δ 180.6 (C=O), 141.7 (C), 138.3 (CH), 129.9 (C), 128.0 (CH), 124.3 (CH), 122.4 (CH), 115.0 (CH_2), 109.8 (CH), 46.1 (CH), 33.8 (CH_2), 30.1 (CH_2), 25.1 (CH_2). **FT-IR (neat, cm^{-1}):** 3204, 2924, 1698, 1619, 1485, 1439, 1336, 1217, 1100. **HRMS (ESI$^+$):** *m/z* calcd. for $C_{13}H_{15}NONa$ [M+Na]$^+$ 224.1046, found 224.1043.

***3-Pentylindolin-2-one* 1h**

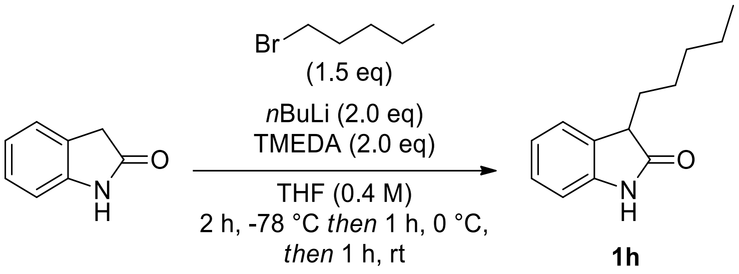

To a solution of oxindole (2.0 g, 15.0 mmol, 1.0 equiv.) in THF (60 mL) at -78 ºC was added *n*BuLi (2.5 M in hexanes, 12.0 mL, 30.0 mmol, 2.0 equiv.) and TMEDA (4.51 mL, 30.0 mmol, 2.0 equiv.) and the resulting solution was stirred for 2 h. 1-Bromopentane (2.79 mL, 22.5 mmol, 1.1 equiv.) was then added dropwise and the solution was slowly warmed up to 0 ºC and stirred for 1 h at that temperature and for 1 h at rt. The reaction was then quenched with aq. NH_4Cl and extracted with ethyl acetate. The combined organic layers were washed with brine, dried over $MgSO_4$, and concentrated in vacuo. The crude was purified by FC (80 g SiO_2, heptane/ethyl acetate, 100/0 to 70/30, 20 CV) to afford **1h** as a light yellow oil (1.18 g, 5.8 mmol, 39%).

^{1}H NMR (600 MHz, $CDCl_3$): δ 9.37 (brs, 1H), 7.21 (m, 2H), 7.02 (t, J = 7.5 Hz, 1H), 6.93 (d, J = 7.8 Hz, 1H), 3.48 (t, J = 6.0 Hz, 1H), 2.02-1.90 (m, 2H), 1.46-1.39 (m, 1H), 1.36-1.31 (m,

1H), 1.31-1.27 (m, 4H), 0.85 (dd, J = 9.3, 4.7 Hz, 3H). **$^{13}C\{^1H\}$ NMR (150 MHz, $CDCl_3$):** δ 181.4 (C=O), 141.9 (C), 130.1 (C), 127.9 (CH), 124.2 (CH), 122.3 (CH), 110.0 (CH), 46.4 (CH), 31.9 (CH_2), 30.6 (CH_2), 25.6 (CH_2), 22.5 (CH_2), 14.1 (CH_3). **HRMS (ESI^+):** *m/z* calcd. for $C_{13}H_{17}NONa$ $[M+Na]^+$ 226.1202, found 226.1202. Spectral and physical data are in accordance with literature.[2]

***3-((1,3-Dioxolan-2-yl)methyl)indolin-2-one* 1i**

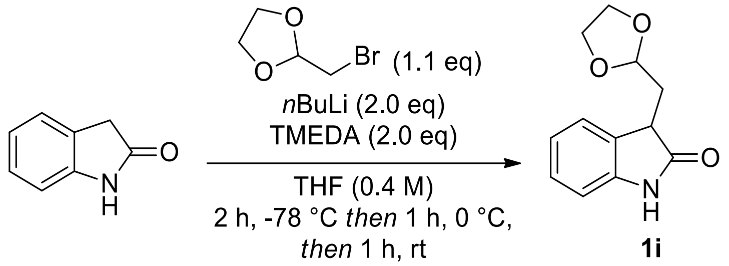

To a solution of oxindole (2.0 g, 15.0 mmol, 1.0 equiv.) in THF (60 mL) at -78 ºC was added *n*BuLi (2.5 M in hexanes, 12.0 mL, 30.0 mmol, 2.0 equiv.) and TMEDA (4.51 mL, 30.0 mmol, 2.0 equiv.) and the resulting solution was stirred for 2 h. 2-Bromomethyl-1,3-dioxolane (1.71 mL, 16.5 mmol, 1.1 equiv.) was then added dropwise and the solution was slowly warmed up to 0 ºC and stirred for 1 h at that temperature and for 1 h at rt. The reaction was then quenched with aq. NH_4Cl and extracted with ethyl acetate. The combined organic layers were washed with brine, dried over $MgSO_4$, and concentrated in vacuo. The crude was purified by FC (80 g SiO_2, heptane/ethyl acetate: 100/0 to 60/40, 20 CV) to afford **1i** as a white solid (529 mg, 2.41 mmol, 16%).

^1H NMR (400 MHz, $CDCl_3$): δ 7.87 (brs, 1H), 7.35 (d, J = 7.4 Hz, 1H), 7.21 (t, J = 7.8 Hz, 1H), 7.02 (t, J = 7.2 Hz, 1H), 6.87 (d, J = 7.8 Hz, 1H), 5.20 (dd, J = 5.9, 3.8 Hz, 1H), 4.04-3.82 (m, 4H), 3.64 (t, J = 6.2 Hz, 1H), 2.41 (ddd, J = 14.2, 5.8, 3.8 Hz, 1H), 2.17 (dt, J = 14.2, 6.2 Hz, 1H). **$^{13}C\{^1H\}$ NMR (100 MHz, $CDCl_3$):** δ 179.7 (C=O), 141.3 (C), 129.4 (C), 128.1 (CH), 125.1 (CH), 122.4 (CH), 109.6 (CH), 102.3 (CH), 66.2 (CH_2), 66.0 (CH_2), 42.2 (CH), 34.4 (CH_2). **FT-IR (neat, cm^{-1}):** 3174, 3130, 3078, 3035, 2953, 2888, 2849, 1694, 1619, 1470, 1140. **HRMS (ESI^+):** *m/z* calcd. for $C_{12}H_{13}NO_3Na$ $[M+Na]^+$ 242.0788, found 242.0785.

***2-(2-Oxoindolin-3-yl)acetonitrile* 1j**

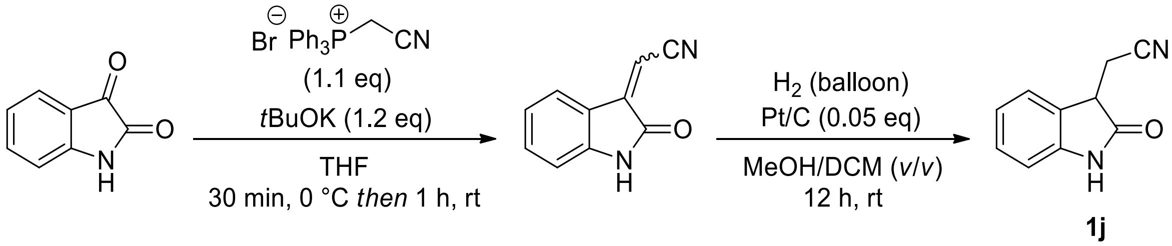

In a two-neck flask, under argon, (cyanomethyl)triphenylphosphonium chloride (3.79 g, 11.2 mmol, 1.1 equiv.) was dissolved in THF (10 mL) and cooled in an ice-bath. *t*BuOK (1.42 g, 12.2 mmol; 1.2 equiv.) was added and the mixture was stirred at 0 °C for 30 min. Then, isatin (1.5 g, 10.2 mmol, 1.0 equiv.) was added portion wise and stirred at rt for 1 h. The reaction was quenched with H_2O, extracted with ethyl acetate, dried over $MgSO_4$ and concentrated under

reduced pressure. 2-(2-Oxoindolin-3-ylidene)acetonitrile (1.2 g, 7.08 mmol, 69%) was obtained as an orange solid.

2-(2-Oxoindolin-3-ylidene)acetonitrile (1.2 g, 7.08 mmol, 1.0 equiv.) was dissolved in a mixture of MeOH/DCM (24 mL, *v*/*v*). Pt/C (69.0 mg, 0.350 mmol, 0.05 equiv.) was added to this solution, and the resulting mixture was stirred under hydrogen atmosphere (balloon) for 12 h at rt. The reaction mixture was then passed through celite, washed with DCM and concentrated in vacuo. The brown sticky solid was recrystallized in DCM and filtrated to afford **1j** as a white solid (426 g, 2.47 mmol, 35%).

^{1}H NMR (600 MHz, $CDCl_3$): δ 8.37 (brs, 1H), 7.49 (d, J = 7.5 Hz, 1H), 7.31 (t, J = 7.7 Hz, 1H), 7.11 (t, J = 7.5 Hz, 1H), 6.94 (d, J = 7.7 Hz, 1H), 3.71 (dd, J = 8.9, 4.8 Hz, 1H), 3.10 (dd, J = 16.9, 4.8 Hz, 1H), 2.75 (dd, J = 16.9, 8.9 Hz, 1H). **^{13}C{^{1}H} NMR (150 MHz, $CDCl_3$):** δ 176.4 (C=O), 141.3 (C), 129.6 (CH), 126.3 (C), 124.7 (CH), 123.4 (CH), 117.2 (C), 110.4 (CH), 41.9 (CH), 19.0 (CH_2). **HRMS (ESI$^+$):** *m/z* calcd. for $C_{10}H_8N_2ONa$ $[M+Na]^+$ 195.0529, found 195.0530. Spectral and physical data are in accordance with literature.[5]

Ethyl 2-(2-oxoindolin-3-yl)acetate 1k

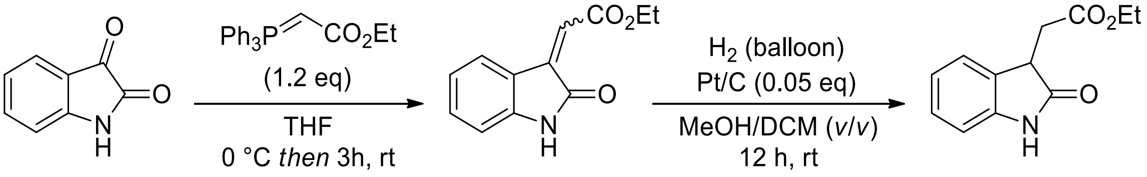

In a two-neck flask, under argon, ethyl(triphenylphosphoranylidene)acetate (2.8 g, 8.20 mmol, 1.2 equiv.) was dissolved in THF (10.0 mL) and cooled in an ice-bath. Then, isatin (1.0 g, 6.80 mmol, 1.0 equiv.) was added portion wise and stirred at rt for 3 h. The reaction was quenched with H_2O, extracted with ethyl acetate, dried over $MgSO_4$ and concentrated under reduced pressure. The crude product was purified by FC (40 g SiO_2, heptane/ethyl acetate, 100/0 to 70/30, 15 CV) to provide ethyl 2-(2-oxoindolin-3-ylidene)acetate (1.06 g, 4.88 mmol, 72%) as an orange solid.

Ethyl 2-(2-oxoindolin-3-ylidene)acetate (1.0 g, 4.6 mmol, 1.0 equiv.) was dissolved in a mixture of MeOH/DCM (20 mL, *v*/*v*). Pt/C (44.9 mg, 0.23 mmol, 0.05 equiv.) was added to this solution, and the resulting mixture was stirred under hydrogen atmosphere (balloon) for 12 h at rt. The reaction mixture was then passed through celite, washed with DCM and concentrated in vacuo to afford **1k** as a pale yellow solid (1.0 g, 4.6 mmol, 99%).

^{1}H NMR (400 MHz, $CDCl_3$): δ 8.89 (brs, 1H), 7.29-7.21 (m, 2H), 7.02 (t, J = 7.5 Hz, 1H), 6.92 (d, J = 7.7 Hz, 1H), 4.21-4.11 (m, 2H), 3.86-3.79 (m, 1H), 3.09 (dd, J = 16.9, 3.7 Hz, 1H), 2.85 (dd, J = 16.9, 7.9 Hz, 1H), 1.22 (td, J = 7.1, 0.9 Hz, 3H). **^{13}C{^{1}H} NMR (100 MHz, $CDCl_3$):** δ 179.5 (C=O), 171.2 (C=O), 141.8 (C), 128.9 (C), 128.4 (CH), 124.2 (CH), 122.6 (CH), 110.0 (CH), 61.1 (CH_2), 42.5 (CH), 34.9 (CH_2), 14.2 (CH_3). **HRMS (ESI$^+$):** *m/z* calcd. for $C_{12}H_{13}NO_3Na$ $[M+Na]^+$ 242.0788, found 242.0788. Spectral and physical data are in accordance with literature.[6]

***3-(3-Methoxyphenyl)indolin-2-one* 1l**

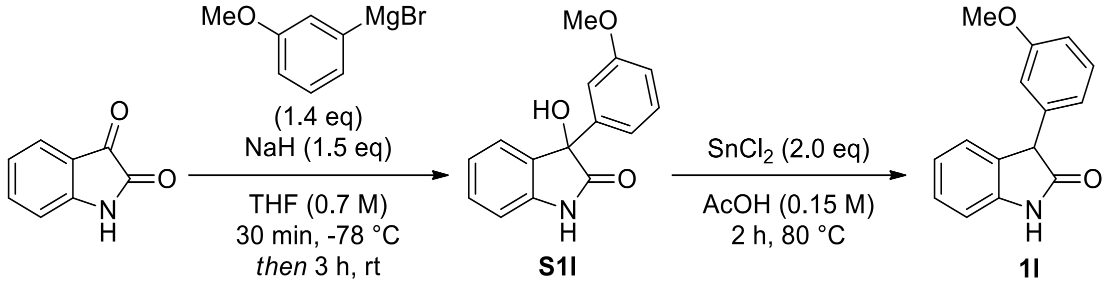

General procedure **B** was followed with isatin (2.0 g, 13.6 mmol) and 3-methoxyphenylmagnesium bromide (1 M in 2-MeTHF, 19.0 mL, 19.0 mmol) to afford **S1l** as a white solid (2.75 g, 10.8 mmol, 79%). **^{1}H NMR (400 MHz, DMSO-d$_6$):** δ 10.39 (s, 1H), 7.24 (td, J = 7.7, 1.1 Hz, 1H), 7.19 (t, J = 8.0 Hz, 1H), 7.09 (d, J = 7.2 Hz, 1H), 6.98-6.93 (m, 2H), 6.89 (d, J = 7.7 Hz, 1H), 6.83 (dd, J = 8.0, 2.2 Hz, 1H), 6.69 (d, J = 7.8 Hz, 1H), 6.62 (s, 1H), 3.72 (s, 3H). **^{13}C{^{1}H} NMR (100 MHz, DMSO-d$_6$):** δ 178.3 (C=O), 159.1 (C), 143.1 (C), 141.9 (C), 133.7 (C), 129.2 (CH), 129.2 (CH), 124.7 (CH), 122.0 (CH), 117.5 (CH), 112.5 (CH), 111.5 (CH), 109.8 (CH), 77.2 (C), 55.0 (CH_3). **HRMS (ESI$^+$):** *m/z* calcd. for $C_{15}H_{13}NO_3Na$ $[M+Na]^+$ 278.0788, found 278.0804. Spectral and physical data are in accordance with literature.[9]

The general procedure **C** was followed with **S1l** (2.0 g, 7.83 mmol). Purification was performed with flash column chromatography over silica gel (40 g SiO_2, heptane/EtOAc, 100/0 to 70/30, gradient) to afford **1l** as a white solid (1.84 g, 7.69 mmol, 98%). **^{1}H NMR (400 MHz, $CDCl_3$):** δ 8.69 (brs, 1H), 7.25 (m, 2H), 7.13 (d, J = 7.5 Hz, 1H), 7.02 (t, J = 7.5 Hz, 1H), 6.93 (d, J = 7.8 Hz, 1H), 6.84 (dd, J = 12.8, 5.0 Hz, 2H), 6.77-6.75 (m, 1H), 4.61 (s, 1H), 3.78 (s, 3H). **^{13}C{^{1}H} (100 MHz, $CDCl_3$):** δ 178.5 (C=O), 160.1 (C), 141.7 (C), 138.0 (C), 130.1 (CH), 129.6 (C), 128.6 (CH), 125.5 (CH), 122.9 (CH), 121.0 (CH), 114.5 (CH), 113.2 (CH), 110.1 (CH), 55.4 (CH_3), 52.7 (CH). **HRMS (ESI$^+$):** *m/z* calcd. for $C_{15}H_{13}NO_2Na$ $[M+Na]^+$ 262.0838, found 262.0838. Spectral and physical data are in accordance with literature.[9]

***3-(4-Methoxyphenyl)indolin-2-one* 1m**

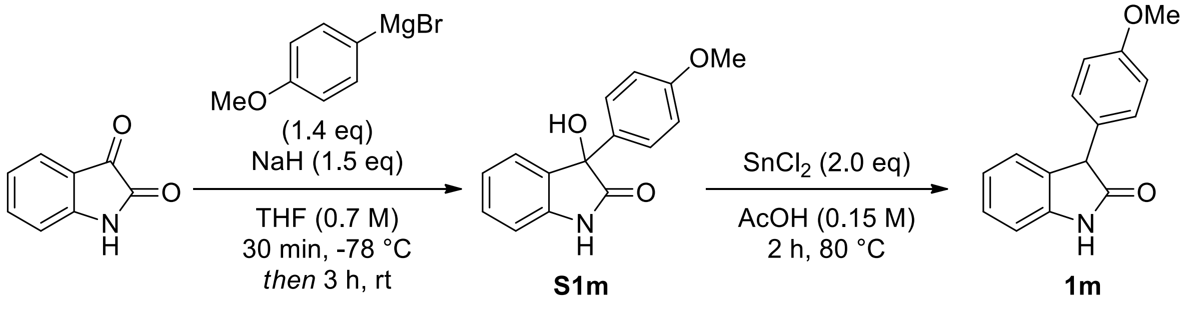

The general procedure **B** was followed with isatin (3.0 g, 20.4 mmol) and 4-bromoanisole (3.57 mL, 28.5 mmol) to afford **S1m** as a white solid (4.08 g, 16.0 mmol, 78%). **^{1}H NMR (400 MHz, DMSO-d$_6$):** δ 10.32 (s, 1H), 7.24 (td, J = 7.6, 1.1 Hz, 1H), 7.18 (d, J = 8.8 Hz, 2H), 7.10 (d, J = 7.2 Hz, 1H), 6.96 (t, J = 7.6 Hz, 1H), 6.89-6.85 (m, 3H), 6.51 (s, 1H), 3.71 (s, 3H). **^{13}C{^{1}H} NMR (100 MHz, DMSO-d$_6$):** δ 178.6 (C=O), 158.7 (C), 141.9 (C), 133.7 (C), 133.5 (C), 129.1 (CH), 126.8 (2CH), 124.8 (CH), 121.9 (CH), 113.4 (2CH), 109.8 (CH), 76.9 (C),

55.1 (CH_3). **FT-IR (neat, cm^{-1}):** 3270, 1718, 1684, 1610, 1509, 1466, 1347, 1297, 1250, 1177, 1123, 1106, 1076, 1033. **HRMS (ESI$^+$):** *m/z* calcd. for $C_{15}H_{13}NO_3Na$ [M+Na]$^+$ 278.0788, found 278.0775. Spectral and physical data are in accordance with literature.[8]

The general procedure **C** was followed with **S1m** (3.5 g, 13.7 mmol) Purification was performed with flash column chromatography over silica gel (40 g SiO_2, heptane/EtOAc, 100/0 to 70/30, gradient) to afford **1m** as a white solid (2.7 g, 11.7 mmol, 82%). **^{1}H NMR (400 MHz, $CDCl_3$):** δ 8.61 (brs, 1H), 7.24 (dd, *J* = 11.1, 3.4 Hz, 1H), 7.16-7.11 (m, 3H), 7.03 (td, *J* = 7.7, 0.6 Hz, 1H), 6.93 (d, *J* = 7.7 Hz, 1H), 6.88 (d, *J* = 8.7 Hz, 2H), 4.59 (s, 1H), 3.79 (s, 3H). **^{13}C{^{1}H} NMR (100 MHz, $CDCl_3$):** δ 179.1 (C=O), 159.3 (C), 141.6 (C), 130.0 (C), 129.7 (2CH), 128.5 (CH + C), 125.4 (CH), 122.9 (CH), 114.6 (2CH), 110.1 (CH), 55.4 (CH_3), 52.0 (CH). **HRMS (ESI$^+$):** *m/z* calcd. for $C_{15}H_{13}NO_2Na$ [M+Na]$^+$ 262.0838, found 262.0838. Spectral and physical data are in accordance with literature.[7]

***3-Phenylindolin-2-one* 1n**

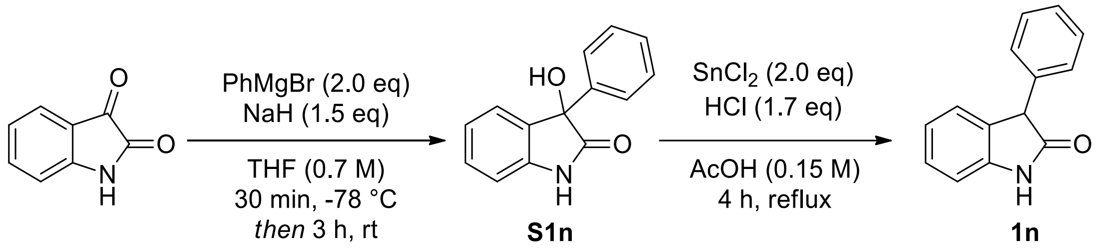

The general procedure **B** was followed with isatin (2.0 g, 13.6 mmol) and phenylmagnesium bromide (3 M in Et_2O, 9.1 mL, 27.2 mmol) to afford **S1n** as a yellow solid (1.2 g, 5.33 mmol, 39%). **^{1}H NMR (400 MHz, DMSO-d$_6$):** δ 10.40 (s, 1H), 7.33-7.23 (m, 6H), 7.10 (d, *J* = 6.9 Hz, 1H), 6.96 (td, *J* = 7.7, 1.1 Hz, 1H), 6.91 (d, *J* = 7.7 Hz, 1H), 6.63 (s, 1H). **^{13}C{^{1}H} NMR (100 MHz, DMSO-d$_6$):** δ 178.5 (C=O), 142.0 (C), 141.6 (C), 133.8 (C), 129.2 (CH), 128.1 (2CH), 127.4 (CH), 125.4 (2CH), 124.8 (CH), 122.1 (CH), 109.9 (CH), 77.3 (C). **FT-IR (neat, cm^{-1}):** 3408, 1703, 1615, 1467, 1360, 1338, 1180, 1156, 1119, 1067. **HRMS (ESI$^+$):** *m/z* calcd. for $C_{14}H_{11}NO_2Na$ [M+Na]$^+$ 248.0682, found 248.0670. Spectral and physical data are in accordance with literature.[8]

The general procedure **C** was followed with **S1n** (500 mg, 2.22 mmol) Purification was performed with flash column chromatography over silica gel (40 g SiO_2, heptane/EtOAc, 100/0 to 70/30, gradient) to afford **1n** as a white solid (420 mg, 2.01 mmol, 90%). **^{1}H NMR (600 MHz, DMSO-d$_6$):** δ 10.51 (brs, 1H), 7.36-7.31 (m, 2H), 7.29-7.25 (m, 1H), 7.24-7.20 (m, 1H), 7.16-7.12 (m, 2H), 7.03 (d, *J* = 7.4 Hz, 1H), 6.94 (td, *J* = 7.5, 1.0 Hz, 1H), 6.93-6.90 (m, 1H), 4.74 (s, 1H). **^{13}C{^{1}H} NMR (150 MHz, DMSO-d$_6$):** δ 177.2 (C=O), 142.7 (C), 137.7 (C), 130.0 (C), 128.7 (2CH), 128.3 (2CH), 128.1 (CH), 127.1 (CH), 124.8 (CH), 121.7 (CH), 109.5 (CH), 51.8 (CH). **HRMS (ESI$^+$):** *m/z* calcd. for $C_{14}H_{11}NONa$ [M+Na]$^+$ 232.0733, found 232.0732. Spectral and physical data are in accordance with literature.[9]

***3-(p-Tolyl)indolin-2-one* 1o**

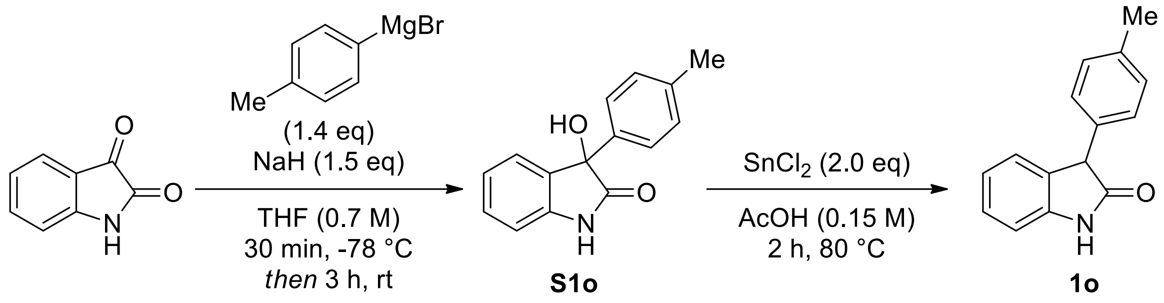

The general procedure **B** was followed with isatin (1.0 g, 6.80 mmol) and 4-bromotoluene (1.17 mL, 9.52 mmol) to afford **S1o** as a yellow solid (690 mg, 2.88 mmol, 42%). **^{1}H NMR (600 MHz, CDCl$_3$):** *δ* 8.20 (brs, 1H), 7.29 (d, *J* = 8.1 Hz, 2H), 7.27-7.22 (m, 2H), 7.13 (d, *J* = 8.1 Hz, 2H), 7.04 (t, *J* = 7.6 Hz, 1H), 6.89 (d, *J* = 7.6 Hz, 1H), 2.31 (s, 3H). **^{13}C{^{1}H} NMR (150 MHz, CDCl$_3$):** *δ* 179.8 (C=O), 140.6 (C), 138.4 (C), 137.0 (C), 132.3 (C), 130.0 (CH), 129.5 (2CH), 125.4 (CH), 125.4 (2CH), 123.7 (CH), 110.6 (CH), 78.3 (C), 21.3 (CH$_3$). **HRMS (ESI$^+$):** *m/z* calcd. for $C_{15}H_{13}NO_2Na$ [M+Na]$^+$ 262.0839, found 262.0841. Spectral and physical data are in accordance with literature.[8]

The general procedure **C** was followed with **S1o** (600 mg, 2.51 mmol) Purification was performed with flash column chromatography over silica gel (40 g SiO_2, heptane/EtOAc, 100/0 to 70/30, gradient) to afford **1o** as a white solid (553 mg, 2.48 mmol, 99%). **^{1}H NMR (400 MHz, CDCl$_3$):** *δ* 8.87 (brs, 1H), 7.24 (t, *J* = 7.8 Hz, 1H), 7.16 (d, *J* = 7.9 Hz, 2H), 7.13-7.10 (m, 3H), 7.02 (t, *J* = 7.5 Hz, 1H), 6.93 (d, *J* = 7.8 Hz, 1H), 4.60 (s, 1H), 2.34 (s, 3H). **^{13}C{^{1}H} NMR (100 MHz, CDCl$_3$):** *δ* 179.1 (C=O), 141.8 (C), 137.5 (C), 133.6 (C), 130.0 (C), 129.8 (2CH), 128.5 (2CH), 128.4 (CH), 125.4 (CH), 122.8 (CH), 110.1 (CH), 52.5 (CH), 21.3 (CH$_3$). **HRMS (ESI$^+$):** *m/z* calcd. for $C_{15}H_{13}NONa$ [M+Na]$^+$ 246.0889, found 246.0886. Spectral and physical data are in accordance with literature.[9]

***3-(4-(Tert-butyl)phenyl)indolin-2-one* 1p**

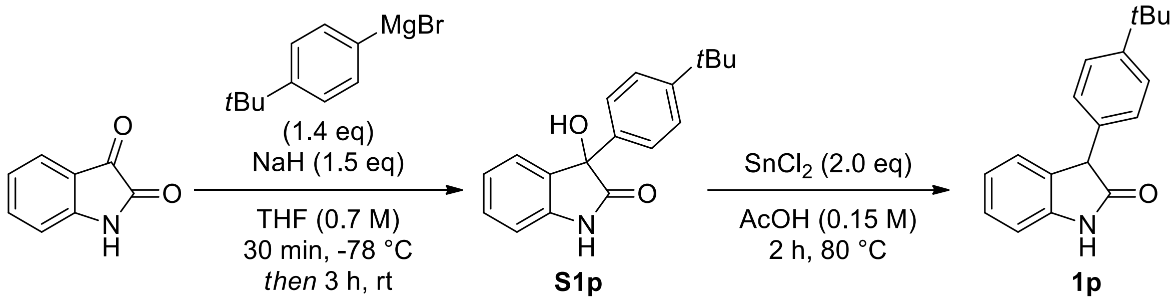

The general procedure **B** was followed with isatin (1.0 g, 6.80 mmol) and 1-bromo-4-tert-butylbenzene (1.65 mL, 9.52 mmol) to afford **S1p** as a yellow solid (1.11 g, 3.95 mmol, 58%). **^{1}H NMR (600 MHz, DMSO-d$_6$):** *δ* 10.36 (s, 1H), 7.32 (d, *J* = 8.5 Hz, 2H), 7.24 (td, *J* = 7.7, 0.8 Hz, 1H), 7.19 (d, *J* = 8.5 Hz, 2H), 7.10 (d, *J* = 7.4 Hz, 1H), 6.96 (t, *J* = 7.4 Hz, 1H), 6.88 (d, *J* = 7.7 Hz, 1H), 6.53 (s, 1H), 1.24 (s, 9H). **^{13}C{^{1}H} NMR (150 MHz, DMSO-d$_6$):** *δ* 178.6 (C=O), 149.8 (C), 141.9 (C), 138.6 (C), 133.8 (C), 129.2 (CH), 125.2 (2CH), 124.9 (2CH), 124.8 (CH), 122.0 (CH), 109.8 (CH), 77.2 (C), 34.2 (C), 31.1 (3CH$_3$). **HRMS (ESI$^+$):**

m/z calcd. for $C_{18}H_{19}NO_2Na$ $[M+Na]^+$ 304.1308, found 304.1306. Spectral and physical data are in accordance with literature.[10]

The general procedure **C** was followed with **S1p** (800 mg, 2.84 mmol) Purification was performed with flash column chromatography over silica gel (40 g SiO_2, heptane/EtOAc, 100/0 to 70/30, gradient) to afford **2p** as a white solid (720 mg, 2.71 mmol, 91%). **^{1}H NMR (400 MHz, $CDCl_3$):** δ 8.36 (brs, 1H), 7.36 (d, J = 8.3 Hz, 2H), 7.27-7.22 (m, 1H), 7.17-7.13 (m, 3H), 7.03 (t, J = 7.5 Hz, 1H), 6.94 (d, J = 7.8 Hz, 1H), 4.62 (s, 1H), 1.30 (s, 9H). **^{13}C{^{1}H} NMR (100 MHz, $CDCl_3$):** δ 178.7 (C=O), 150.6 (C), 141.6 (C), 133.4 (C), 129.9 (C), 128.5 (CH), 128.2 (2CH), 126.1 (2CH), 125.1 (CH), 122.9 (CH), 110.0 (CH), 52.3 (CH), 34.7 (C), 31.5 ($3CH_3$). **HRMS (ESI$^+$):** *m/z* calcd. for $C_{18}H_{19}NONa$ $[M+Na]^+$ 288.1359, found 288.1356. Spectral and physical data are in accordance with literature.[7]

***3-([1,1'-Biphenyl]-4-yl)indolin-2-one* 1q**

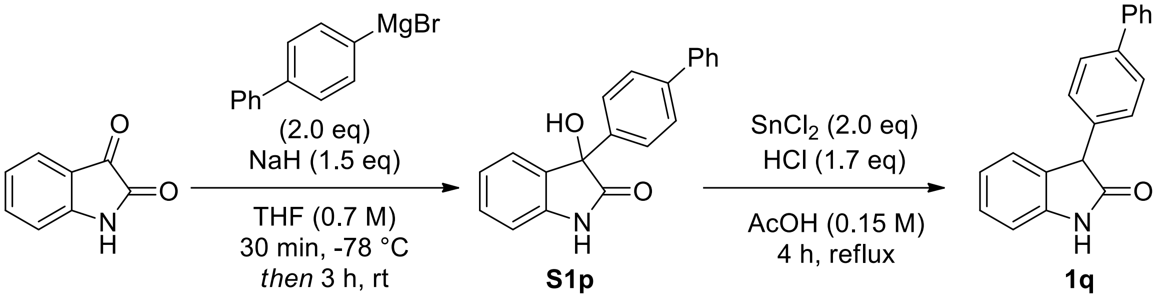

General procedure **B** was followed with isatin (1.0 g, 6.80 mmol) and 4-bromobiphenyl (3.4 mL, 13.6 mmol) to afford **S1q** as a white solid (1.07 g, 3.54 mmol, 52%). **^{1}H NMR (600 MHz, DMSO-d$_6$):** δ 10.45 (s, 1H), 7.69-7.59 (m, 4H), 7.52-7.42 (m, 2H), 7.42-7.33 (m, 3H), 7.33-7.24 (m, 1H), 7.16 (d, J = 6.7 Hz, 1H), 7.05-6.89 (m, 2H), 6.70 (s, 1H). **^{13}C{^{1}H} NMR (150 MHz, DMSO-d$_6$):** δ 178.4 (C=O), 142.0 (C), 140.7 (C), 139.9 (C), 139.4 (C), 133.6 (C), 129.3 (CH), 128.9 (CH), 127.5 (CH), 126.7 (CH), 126.5 (CH), 126.1 (CH), 124.8 (CH), 122.1 (CH), 109.9 (CH), 77.2 (C). **HRMS (ESI$^+$):** *m/z* calcd. for $C_{20}H_{15}NO_2Na$ $[M+Na]^+$ 324.0995, found 324.0991. Spectral and physical data are in accordance with literature.[10]

The general procedure **C** was followed with **S1q** (400 mg, 1.33 mmol) Purification was performed with flash column chromatography over silica gel (40 g SiO_2, heptane/EtOAc, 100/0 to 70/30, gradient) to afford **1q** as a white solid (337 mg, 1.18 mmol, 89%). **^{1}H NMR (600 MHz, DMSO-d$_6$):** δ 10.55 (s, 1H), 7.68-7.59 (m, 4H), 7.48-7.42 (m, 2H), 7.37-7.34 (m, 1H), 7.23 (dd, J = 11.9, 5.4 Hz, 3H), 7.08 (d, J = 7.4 Hz, 1H), 6.97 (td, J = 7.5, 0.9 Hz, 1H), 6.93 (d, J = 7.8 Hz, 1H), 4.81 (s, 1H). **^{13}C{^{1}H} NMR (150 MHz, DMSO-d$_6$):** δ 177.2 (C=O), 142.8 (C), 139.9 (C), 139.1 (C), 136.9 (C), 130.0 (C), 129.0 (2CH), 129.0 (2CH), 128.2 (CH), 127.5 (CH), 127.1 (2CH), 126.7 (2CH), 124.9 (CH), 121.8 (CH), 109.6 (CH), 51.5 (CH). **FT-IR (neat, cm^{-1})**: 3080, 2918, 1700, 1612, 1466, 1402, 1297, 1264, 1220, 1070. **HRMS (ESI$^+$):** *m/z* calcd. for $C_{20}H_{15}NONa$ $[M+Na]^+$ 308.1046, found 308.1044.

***3-(4-Fluorophenyl)indolin-2-one* 1r**

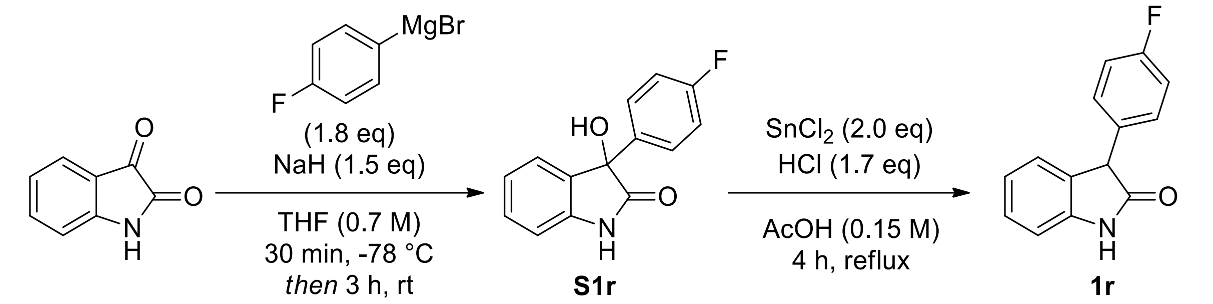

General procedure **B** was followed with isatin (500 mg, 3.40 mmol) and 4-fluorophenylmagnesium bromide (1.0 M in THF, 6.12 mL, 6.12 mmol) to afford **S1r** as a light yellow solid (452 mg, 1.86 mmol, 55%). **^{1}H NMR (600 MHz, DMSO-d$_6$):** δ 10.42 (s, 1H), 7.32-7.27 (m, 2H), 7.26 (td, J = 7.7, 1.2 Hz, 1H), 7.17-7.09 (m, 3H), 6.98 (td, J = 7.5, 0.7 Hz, 1H), 6.90 (d, J = 7.7 Hz, 1H), 6.69 (s, 1H). **^{13}C{^{1}H} NMR (150 MHz, DMSO-d$_6$):** δ 178.3 (C=O), 161.6 (d, J^{CF} = 243.5 Hz, C), 141.9 (C), 137.7 (d, J^{CF} = 2.8 Hz, C), 133.4 (C), 129.4 (CH), 127.6 (d, J^{CF} = 8.3 Hz, 2CH), 124.8 (CH), 122.2 (CH), 114.9 (d, J^{CF} = 21.4 Hz, 2CH), 110.0 (CH), 76.8 (C). **^{19}F NMR (565 MHz, DMSO-d$_6$):** δ -115.23. **FT-IR (neat, cm^{-1}):** 3433, 3178 1693, 1620, 1602, 1503, 1472, 1229, 1199, 1172, 1121. **HRMS (ESI$^+$):** *m/z* calcd. for $C_{14}H_{10}NO_2FNa$ [M+Na]$^+$ 266.0588, found 266.0591.

The general procedure **C** was followed with **S1r** (300 mg, 1.23 mmol) Purification was performed with flash column chromatography over silica gel (40 g SiO_2, heptane/EtOAc, 100/0 to 70/30, gradient) to afford **1r** as a white solid (150 mg, 0.66 mmol, 54%). **^{1}H NMR (700 MHz, DMSO-d$_6$):** δ 10.53 (brs, 1H), 7.23 (t, J = 7.7 Hz, 1H), 7.20-7.15 (m, 4H), 7.04 (t, J = 9.3 Hz, 1H), 6.96 (td, J = 7.5, 0.7 Hz, 1H), 6.91 (d, J = 7.8 Hz, 1H), 4.78 (s, 1H). **^{13}C{^{1}H} NMR (176 MHz, DMSO-d$_6$):** δ 177.1 (C=O), 161.4 (d, J^{CF} = 243.0 Hz, C), 142.7 (C), 133.8 (d, J^{CF} = 3.1 Hz, C), 130.3 (d, J^{CF} = 8.1 Hz, 2CH), 129.8 (C), 128.2 (CH), 124.8 (CH), 121.8 (CH), 115.5 (d, J^{CF} = 21.4 Hz, 2CH), 109.6 (CH), 50.9 (CH). **^{19}F NMR (659 MHz, DMSO-d$_6$):** δ -155.63. **HRMS (ESI$^+$):** *m/z* calcd. for $C_{14}H_{10}FNONa$ [M+Na]$^+$ 250.0639, found 250.0638. Spectral and physical data are in accordance with literature.[9]

***3-(4-Chlorophenyl)indolin-2-one* 1s**

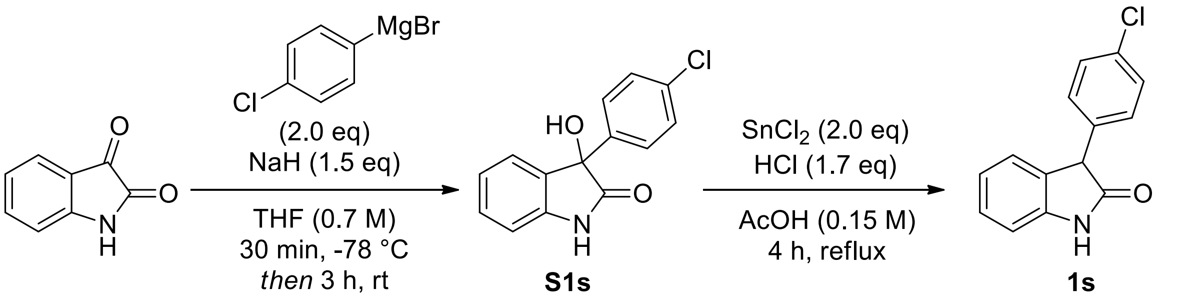

The general procedure **B** was followed with isatin (500 mg, 3.40 mmol) and 4-chlorophenylmagnesium bromide (1.0 M in THF, 6.8 mL, 6.80 mmol) to afford **S1s** as a light yellow solid (200 mg, 0.77 mmol, 23%). **^{1}H NMR (600 MHz, DMSO-d$_6$):** δ 10.45 (s, 1H), 7.32 (d, J = 8.5 Hz, 2H), 7.28-7.24 (m, 3H), 7.10 (d, J = 7.3 Hz, 1H), 6.98 (t, J = 7.5 Hz, 1H), 6.91 (d, J = 7.8 Hz, 1H), 6.74 (s, 1H). **^{13}C{^{1}H} NMR (150 MHz, DMSO-d$_6$):** δ 178.1 (C=O),

142.0 (C), 140.5 (C), 133.2 (C), 132.2 (C), 129.5 (CH), 128.2 (2CH), 127.4 (2CH), 124.8 (CH), 122.2 (CH), 110.0 (CH), 76.9 (C). **FT-IR (neat, cm^{-1}):** 3189, 1714, 1620, 1489, 1471, 1396, 1181, 1102. **HRMS (ESI$^+$):** *m/z* calcd. for $C_{14}H_{10}NO_2ClNa$ [M+Na]$^+$ 282.0298, found 282.0293.

The general procedure **C** was followed with **S1s** (250 mg, 0.96 mmol) Purification was performed with flash column chromatography over silica gel (40 g SiO_2, heptane/EtOAc, 100/0 to 70/30, gradient) to afford **1s** as a yellow solid (163 mg, 0.67 mmol, 70%). **^{1}H NMR (600 MHz, $CDCl_3$):** δ 8.39 (brs, 1H), 7.32 (d, J = 8.5 Hz, 2H), 7.28-7.25 (m, 1H), 7.17 (d, J = 8.5 Hz, 2H), 7.11 (d, J = 7.4 Hz, 1H), 7.05 (td, J = 7.5, 0.6 Hz, 1H), 6.94 (d, J = 7.8 Hz, 1H), 4.61 (s, 1H). **^{13}C{^{1}H} NMR (150 MHz, $CDCl_3$):** δ 177.9 (C=O), 141.6 (C), 136.0 (C), 133.8 (C), 130.0 (2CH), 129.2 (2CH), 129.1 (C), 128.8 (CH), 125.5 (CH), 123.1 (CH), 110.1 (CH), 52.0 (CH). **FT-IR (neat, cm^{-1}):** 3175, 1702 , 1669, 1617, 1488, 1470, 1328, 1218, 1091. **HRMS (ESI$^+$):** *m/z* calcd. for $C_{14}H_{10}NOClNa$ [M+Na]$^+$ 266.0343, found 266.0340.

***7-Chloro-3-(4-methoxyphenyl)indolin-2-one* 3a**

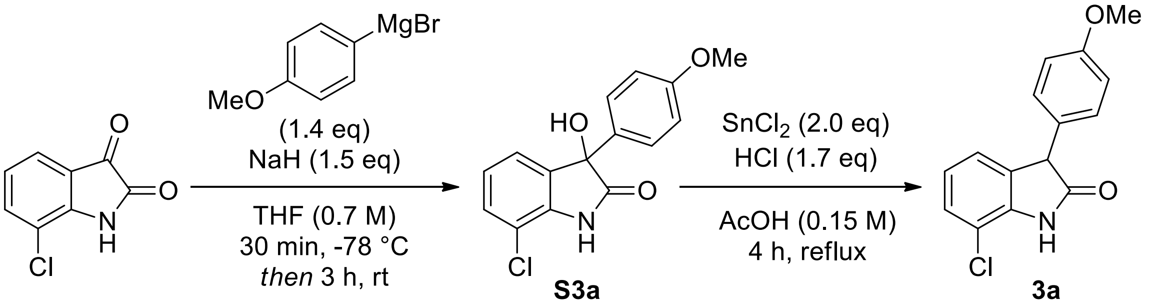

General procedure **B** was followed with 7-chloroisatin (1.0 g, 5.51 mmol) and bromoanisole (1.38 mL, 11.0 mmol, 2.0 equiv.) to afford **S3a** as a yellow solid (1.06 g, 3.67 mmol, 66%). **^{1}H NMR (600 MHz, $CDCl_3$):** δ 7.96 (s, 1H), 7.27 (d, J = 8.9 Hz, 2H), 7.30-7.28 (m, 1H), 7.11 (d, J = 7.4 Hz, 1H), 6.95 (t, J = 7.9 Hz, 1H), 6.79 (d, J = 8.9 Hz, 2H), 3.71 (s, 3H). **^{13}C{^{1}H} NMR (151 MHz, CDCl3) δ** 178.7 (C=O), 160.0 (C), 138.3 (C), 133.5 (C), 131.4 (C), 129.8 (CH), 126.9 (2CH), 124.5 (CH), 123.7 (CH), 115.7 (C), 114.3 (2CH), 78.9 (C), 55.5 (CH_3). **FT-IR (neat, cm^{-1}):** 3301 3136 1720 1617 1508 1472 1454 1249 1184 1166 1133. **HRMS (ESI$^+$):** *m/z* calcd. for $C_{15}H_{12}ClNO_3Na$ [M+Na]$^+$ 312.0398, found 312.0396.

General procedure **C** was followed with **S3a** (250 mg, 0.860 mmol) Purification was performed with flash column chromatography over silica gel (40 g SiO_2, heptane/EtOAc, 100/0 to 70/30, gradient) to afford **3a** as a light yellow solid (140 mg, 0.510 mmol, 59%). **^{1}H NMR (500 MHz, DMSO-d_6):** δ 10.90 (s, 1H), 7.28 (dd, J = 6.7, 2.1 Hz, 1H), 7.06 (d, J = 8.7 Hz, 2H), 6.97 (d, J = 6.7 Hz, 2H), 6.90 (d, J = 8.7 Hz, 2H), 4.82 (s, 1H), 3.73 (s, 3H). **^{13}C{^{1}H} NMR (126 MHz, DMSO-d_6):** δ 177.3 (C=O), 158.5 (C), 140.4 (C), 132.2 (C), 129.4 (2CH), 128.9 (C), 128.0 (CH), 123.4 (CH), 122.9 (CH), 114.2 (2CH), 113.7 (C), 55.1 (CH_3), 51.7 (CH). **FT-IR (neat, cm^{-1}):** 3134, 3065, 1706, 1609, 1512, 1473, 1453, 1253, 1175. **HRMS (ESI$^+$):** *m/z* calcd. for $C_{15}H_{12}NO_2ClNa$ [M+Na]$^+$ 296.0449, found 296.0448.

***3-(4-Methoxyphenyl)-7-(trifluoromethyl)indolin-2-one* 3b**

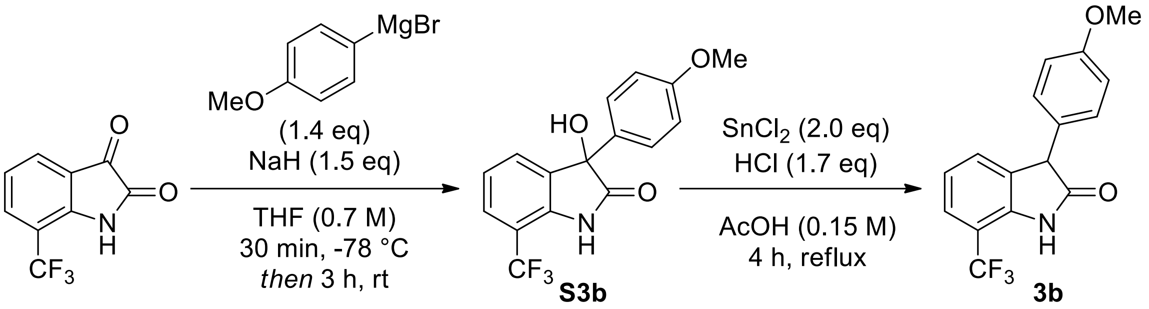

General procedure **B** was followed with 7-(trifluoromethyl)isatin (300 mg, 1.39 mmol) and bromoanisole (0.23 mL, 1.81 mmol, 1.3 equiv.) to afford **S3b** as a yellow solid (456 mg, 1.91 mmol, 61%). **^{1}H NMR (500 MHz, CDCl$_3$):** *δ* 8.13 (s, 1H), 7.48 (d, *J* = 8.0 Hz, 1H), 7.44 (d, *J* = 7.4 Hz, 1H), 7.32 (d, *J* = 8.9 Hz, 2H), 7.15 (t, *J* = 7.8 Hz, 1H), 6.86 (d, *J* = 8.9 Hz, 2H), 3.77 (s, 3H). **^{13}C{^{1}H} NMR (126 MHz, CDCl$_3$):** *δ* 179.1 (C=O), 160.1 (C), 138.0 (q, J^{CF} = 2.5 Hz, C), 133.8 (C), 131.2 (C), 128.9 (CH), 128.6 (2CH), 126.6 (q, J^{CF} = 4.2 Hz, CH), 123.8 (q, J^{CF} = 272.2 Hz, C), 123.5 (CH), 114.3 (2CH), 113.0 (q, J^{CF} = 33.4 Hz, C), 76.9 (C), 55.4 (CH$_3$). **^{19}F NMR (471 MHz, CDCl$_3$):** *δ* -60.59. **FT-IR (neat, cm^{-1}):** 3267, 2924, 1725, 1610, 1509, 1455, 1333, 1309, 1251, 1164. **HRMS (ESI$^+$):** *m/z* calcd. for $C_{16}H_{12}NO_3F_3Na$ [M+Na]$^+$ 346.0662, found 346.0663.

General procedure **C** was followed with **S3b** (250 mg, 0.770 mmol) Purification was performed with flash column chromatography over silica gel (40 g SiO_2, heptane/EtOAc, 100/0 to 70/30, gradient) to afford **3b** as a white solid (201 mg, 0.650 mmol, 85%). **^{1}H NMR (400 MHz, DMSO-d$_6$):** *δ* 10.96 (s, 1H), 7.50 (d, *J* = 8.0 Hz, 1H), 7.29 (d, *J* = 7.4 Hz, 1H), 7.12 (t, *J* = 7.7 Hz, 1H), 7.06 (d, *J* = 8.6 Hz, 2H), 6.91 (d, *J* = 8.6 Hz, 2H), 4.82 (s, 1H), 3.73 (s, 3H). **^{13}C{^{1}H} NMR (100 MHz, DMSO-d$_6$):** *δ* 178.0 (C=O), 158.6 (C), 139.9 (C), 132.4 (C), 129.5 (2CH), 128.7 (CH), 124.5 (q, J^{CF} = 4.5 Hz, CH), 123.7 (q, J^{CF} = 271.5 Hz, C), 121.8 (CH), 114.3 (2CH), 110.6 (q, J^{CF} = 33.0 Hz, C), 55.1 (CH$_3$), 50.1 (CH). **^{19}F NMR (565 MHz, DMSO):** *δ* -59.98. **FT-IR (neat, cm^{-1}):** 3172, 3106, 2843, 1716, 1609, 1514, 1455, 1344, 1317, 1208, 1178. **HRMS (ESI$^+$):** *m/z* calcd. for $C_{16}H_{12}NO_2F_3Na$ [M+Na]$^+$ 330.0713, found 330.0713.

***6-Methoxy-3-(4-methoxyphenyl)indolin-2-one* 3c**

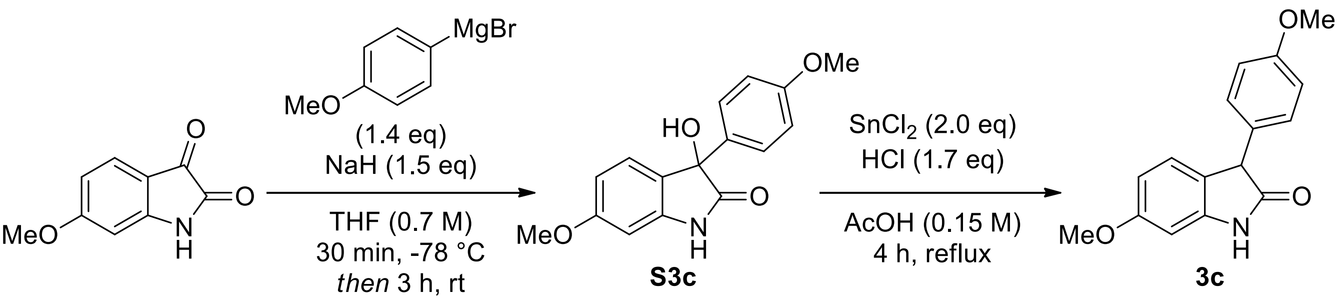

General procedure **B** was followed with 6-methoxyisatin (300 mg, 1.69 mmol) and bromoanisole (0.636 mL, 5.08 mmol, 3.0 equiv.) to afford **S3c** as a yellow solid (260 mg, 0.910 mmol, 54%). **^{1}H NMR (700 MHz, DMSO):** *δ* 10.27 (s, 1H), 7.18 (d, *J* = 8.9 Hz, 2H), 7.00 (d, *J* = 8.2 Hz, 1H), 6.86 (d, *J* = 8.9 Hz, 2H), 6.52 (dd, *J* = 8.2, 2.3 Hz, 1H), 6.44 (d, *J* = 2.3 Hz, 1H), 6.40 (s, 1H), 3.75 (s, 3H), 3.71 (s, 3H). **^{13}C{^{1}H} NMR (176 MHz, DMSO):** *δ*

179.1 (C=O), 160.3 (C), 158.6 (C), 142.2 (C), 133.8 (C), 126.9 (2CH), 125.7 (CH), 125.6 (C), 113.4 (2CH), 106.8 (CH), 96.6 (CH), 76.6 (C), 55.3 (CH_3), 55.1 (CH_3). **FT-IR (neat, cm^{-1}):** 3301, 1723, 1630, 1606, 1507, 1463, 1350, 1298, 1253, 1181 **HRMS (ESI^+):** *m/z* calcd. for $C_{16}H_{15}NO_4Na$ $[M+Na]^+$ 308.0894, found 308.0890.

The general procedure **C** was followed with **S3c** (250 mg, 0.880 mmol) Purification was performed with flash column chromatography over silica gel (40 g SiO_2, heptane/EtOAc, 100/0 to 70/30, gradient) to afford **3c** as a light yellow solid (126 mg, 0.47 mmol, 53%). **^{1}H NMR (500 MHz, DMSO-d$_6$):** δ 10.43 (s, 1H), 7.05 (d, *J* = 8.8 Hz, 2H), 6.93 (d, *J* = 8.5 Hz, 1H), 6.90 (d, *J* = 8.8 Hz, 2H), 6.52 (dd, *J* = 8.2, 2.4 Hz, 1H), 6.49 (d, *J* = 2.3 Hz, 1H), 4.58 (s, 1H), 3.75 (s, 3H), 3.74 (s, 3H). **^{13}C{^{1}H} NMR (126 MHz, DMSO-d$_6$):** δ 178.1 (C=O), 159.6 (C), 158.4 (C), 143.9 (C), 130.0 (C), 129.3 (2CH), 125.5 (CH), 122.2 (C), 114.1 (2CH), 106.6 (CH), 96.4 (CH), 55.3 (CH_3), 55.1 (CH_3), 50.5 (CH). **FT-IR (neat, cm^{-1})** 2974, 2931, 2836, 1712, 1606, 1508, 1485, 1457, 1438, 1297, 1248, 1175. **HRMS (ESI^+):** *m/z* calcd. for $C_{16}H_{15}NO_3Na$ $[M+Na]^+$ 292.0945, found 292.0942.

***5-Methoxy-3-(4-methoxyphenyl)indolin-2-one* 3d**

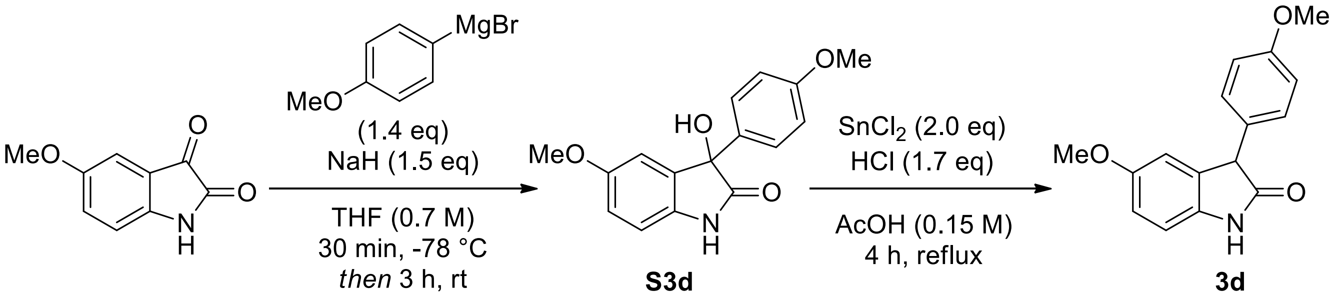

General procedure **B** was followed with 5-methoxylisatin (1.0 g, 5.64 mmol) and bromoanisole (1.41 mL, 11.3 mmol, 2.0 equiv.) to afford **S3d** as a brown solid (735 mg, 2.58 mmol, 46%). **^{1}H NMR (400 MHz, DMSO-d$_6$):** δ 10.15 (s, 1H), 7.18 (d, *J* = 8.8 Hz, 2H), 6.86 (d, *J* = 8.8 Hz, 2H), 6.81 (d, *J* = 2.5 Hz, 2H), 6.70 (d, *J* = 1.4 Hz, 1H), 6.52 (s, 1H), 3.71 (s, 3H), 3.66 (s, 3H). **^{13}C{^{1}H} NMR (100 MHz, DMSO-d$_6$):** δ 178.6 (C=O), 158.7 (C), 155.1 (C), 135.0 (C), 134.9 (C), 133.5 (C), 126.8 (2CH), 113.9 (CH), 113.5 (2CH), 110.4 (CH), 110.3 (CH), 77.3 (C), 55.5 (CH_3), 55.1 (CH_3). **FT-IR (neat, cm^{-1}):** 3261, 1716, 1681, 1607, 1509, 1485, 1464, 1440, 1301, 1256, 1182, 1147. **HRMS (ESI^+):** *m/z* calcd. for $C_{16}H_{15}NO_4Na$ $[M+Na]^+$ 308.0894, found 308.0890.

The general procedure **C** was followed with **S3d** (300 mg, 1.05 mmol) Purification was performed with flash column chromatography over silica gel (40 g SiO_2, heptane/EtOAc, 100/0 to 70/30, gradient) to afford **3d** as a light orange solid (151 mg, 0.561 mmol, 53%). **^{1}H NMR (600 MHz, DMSO-d$_6$):** δ 10.28 (s, 1H), 7.05 (d, *J* = 8.7 Hz, 2H), 6.89 (d, *J* = 8.7 Hz, 2H), 6.82-6.77 (m, 2H), 6.62 (s, 1H), 4.63 (s, 1H), 3.73 (s, 3H), 3.65 (s, 3H). **^{13}C{^{1}H} NMR (150 MHz, DMSO-d$_6$):** δ 177.3 (C=O), 158.4 (C), 154.9 (C), 136.0 (C), 131.6 (C), 129.6 (C), 129.4 (2CH), 114.1 (2CH), 112.8 (CH), 111.5 (CH), 109.8 (CH), 55.4 (CH_3), 55.1 (CH_3), 51.5 (CH). **FT-IR (neat, cm^{-1}):** 3160, 3043, 2839, 1693, 1602, 1513, 1484, 1454, 1441, 1207, 1178. **HRMS (ESI^+):** *m/z* calcd. for $C_{16}H_{15}NO_3Na$ $[M+Na]^+$ 292.0945, found 292.0943.

***5-Bromo-3-(4-methoxyphenyl)indolin-2-one* 3e**

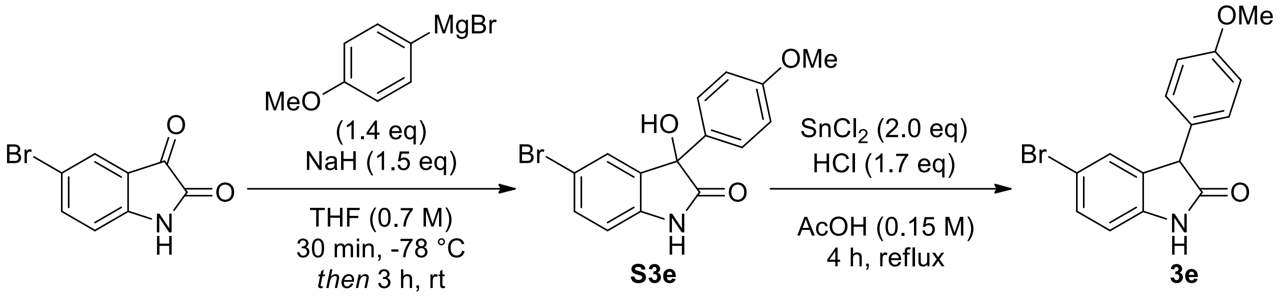

General procedure **B** was followed with 5-bromoisatin (3.0 g, 13.3 mmol) and bromoanisole (2.16 mL, 17.3 mmol, 1.3 equiv.) to afford **S3e** as a light yellow solid (3.6 mg, 10.9 mmol, 82%). **^{1}H NMR (700 MHz, DMSO-d$_6$):** δ 10.50 (s, 1H), 7.43 (dd, *J* = 8.3, 2.1 Hz, 1H), 7.21 (t, *J* = 4.4 Hz, 1H), 7.20-7.17 (m, 2H), 6.91-6.87 (m, 2H), 6.86 (dd, *J* = 8.6, 3.4 Hz, 1H), 6.68 (s, 1H), 3.72 (s, 3H). **^{13}C{^{1}H} NMR (150 MHz, CDCl$_3$):** δ 178.2 (C=O), 158.8 (C), 141.2 (C), 136.2 (C), 132.8 (C), 131.9 (CH), 127.4 (CH), 126.7 (2CH), 113.7 (2CH), 113.6 (C), 112.0 (CH), 76.9 (C), 55.1 (CH$_3$). **FT-IR (neat, cm^{-1}):** 3268, 3178, 1716, 1698, 1672, 1608, 1509, 1473, 1441, 1255, 1170, 1127. **HRMS (ESI$^+$):** *m/z* calcd. for $C_{15}H_{12}BrNO_3Na$ [M+Na]$^+$ 355.9893, found 355.9892.

The general procedure **C** was followed with **S3e** (175 mg, 0.524 mmol) Purification was performed with flash column chromatography over silica gel (40 g SiO_2, heptane/EtOAc, 100/0 to 70/30, gradient) to afford **3e** as a white solid (140 mg, 0.44 mmol, 84%). **^{1}H NMR (600 MHz, CDCl$_3$):** δ 8.43 (s, 1H), 7.39-7.36 (m, 1H), 7.24 (s, 1H), 7.11 (d, *J* = 8.7 Hz, 2H), 6.89 (d, *J* = 8.7 Hz, 2H), 6.81 (d, *J* = 8.3 Hz, 1H), 4.57 (s, 1H), 3.80 (s, 3H). **^{13}C{^{1}H} NMR (150 MHz, CDCl$_3$):** δ 178.2 (C=O), 159.2 (C), 140.6 (C), 132.1 (C), 131.4 (CH), 129.6 (2CH), 128.6 (CH), 127.7 (C), 115.5 (C), 114.7 (2CH), 111.4 (CH), 55.5 (CH$_3$), 52.0 (CH). **FT-IR (neat, cm^{-1}):** 3184, 1710, 1609, 1509, 1473, 1249, 1216, 1177. **HRMS (ESI$^+$):** *m/z* calcd. for $C_{15}H_{12}NO_2BrNa$ [M+Na]$^+$ 339.9944, found 339.9940.

***3-(4-Methoxyphenyl)-5-nitroindolin-2-one* 3f**

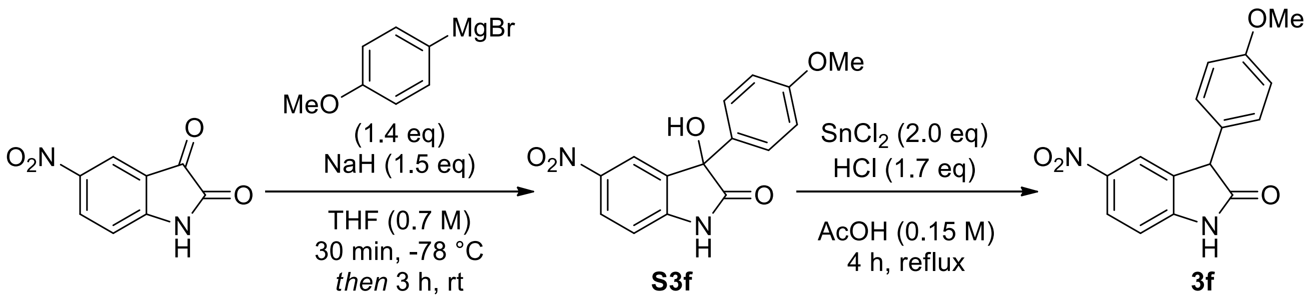

The general procedure **B** was followed with 5-nitroisatin (500 mg, 2.60 mmol) and 4-bromoanisole (0.42 mL, 3.38 mmol, 1.3 equiv.) to afford **S3f** as a white solid (541 mg, 1.80 mmol, 69%). **^{1}H NMR (500 MHz, DMSO-d$_6$):** δ 8.24 (dd, *J* = 8.6, 2.4 Hz, 1H), 7.90 (d, *J* = 2.4 Hz, 1H), 7.23 (d, *J* = 8.9 Hz, 2H), 7.11 (d, *J* = 8.6 Hz, 1H), 6.92-6.88 (m, 3H), 3.72 (s, 3H). **^{13}C{^{1}H} NMR (126 MHz, DMSO-d$_6$):** δ 178.8 (C=O), 159.1 (C), 148.5 (C), 142.5 (C), 134.6 (C), 132.0 (C), 126.8 (2CH), 126.6 (CH), 120.2 (CH), 113.8 (2CH), 110.4 (CH), 76.5

(C), 55.2 (CH_3). **FT-IR (neat, cm^{-1}):** 3285, 1723, 1608, 1521, 1509, 1458, 1344, 1302, 1256, 1176, 1123. **HRMS (ESI$^+$):** *m/z* calcd. for $C_{15}H_{12}N_2O_5Na$ [M+Na]$^+$ 323.0639, found 323.0639.

The general procedure **C** was followed with **S3f** (300 mg, 1.00 mmol) Purification was performed with flash column chromatography over silica gel (40 g SiO_2, heptane/EtOAc, 100/0 to 70/30, gradient) to afford **3f** as a brown solid (120 mg, 0.42 mmol, 42%). **^{1}H NMR (600 MHz, $CDCl_3$):** δ 9.15 (s, 1H), 8.23 (dd, *J* = 8.6, 1.6 Hz, 1H), 8.03 (s, 1H), 7.11 (d, *J* = 8.6 Hz, 2H), 7.03 (d, *J* = 8.6 Hz, 1H), 6.91 (d, *J* = 8.7 Hz, 2H), 4.66 (s, 1H), 3.80 (s, 3H). **^{13}C{^{1}H} NMR (151 MHz, $CDCl_3$) δ** 177.95 (C=O), 158.77 (C), 146.39 (C), 142.90 (C), 129.88 (C), 128.60 (CH), 125.65 (C), 124.73 (CH), 120.38 (CH), 113.93 (CH), 108.88 (CH), 54.50 (CH_3), 50.80 (CH). **FT-IR (neat, cm^{-1}):** 3128, 3080, 2931, 1712, 1621, 1605, 1509, 1456, 1337, 1298, 1183. **HRMS (ESI$^+$):** *m/z* calcd. for $C_{15}H_{12}N_2O_4Na$ [M+Na]$^+$ 307.0690, found 307.0693.

***1,3-Dimethylindolin-2-one* 5a**

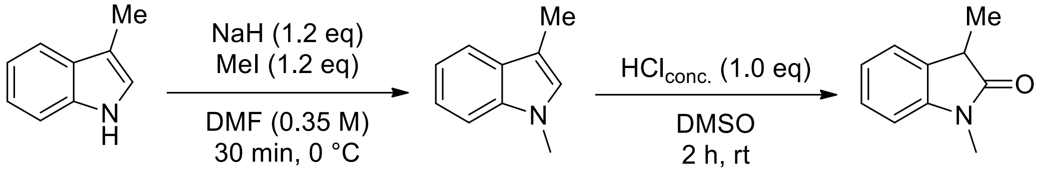

Following a modified reported procedure.[11] To a stirring solution of NaH (60% in mineral oil, 180 mg, 4.57 mmol, 1.2 equiv.) in dry DMF (10 mL), 3-methylindole (500 mg, 3.81 mmol, 1.0 equiv.) was added dropwise at 0 °C. The mixture was allowed to warm to rt and stirred for 30 min. After cooling to 0 °C, MeI (0.29 mL, 4.57 mmol, 1.2 equiv.) was added dropwise. The resulting mixture was stirred at rt for 12 h, then quenched with H_2O and extracted with ethyl acetate. The combined organic phases were dried over $MgSO_4$, filtered, and concentrated under vacuum. The crude product was used directly in the next step without purification.

Following a reported procedure,[12] to a solution of N-methyl-3-methylindole (639 mg, 4.40 mmol, 1.0 equiv.) in DMSO (20 mL) was added slowly concentrated HCl (17.6 mL, 4.40 mmol, 1.0 equiv.) at rt. After stirring for 2 h, the solution was diluted with H_2O (20 mL) and extracted with ethyl acetate. The combined organic layers were washed with sat. $NaHCO_3$ (30 mL), brine (30 mL), dried over anhydrous $MgSO_4$ and concentrated in vacuo. The residue was purified by FC (20 g SiO_2, DCM/ethyl acetate: 100/0 to 95/5, 10 CV) to provide **5a** as an orange oil (366 mg, 2.27 mmol, 52%).

^{1}H NMR (400 MHz, $CDCl_3$): δ 7.29 (dd, *J* = 8.2, 7.3 Hz, 1H), 7.25 (d, *J* = 7.4 Hz, 1H), 7.07 (t, *J* = 7.5 Hz, 1H), 6.84 (d, *J* = 7.8 Hz, 1H), 3.44 (q, *J* = 7.7 Hz, 1H), 3.22 (s, 3H), 1.49 (d, *J* = 7.7 Hz, 3H). Spectral and physical data are in accordance with literature.[13]

***3-Methyl-1-phenylindolin-2-one* 5b**

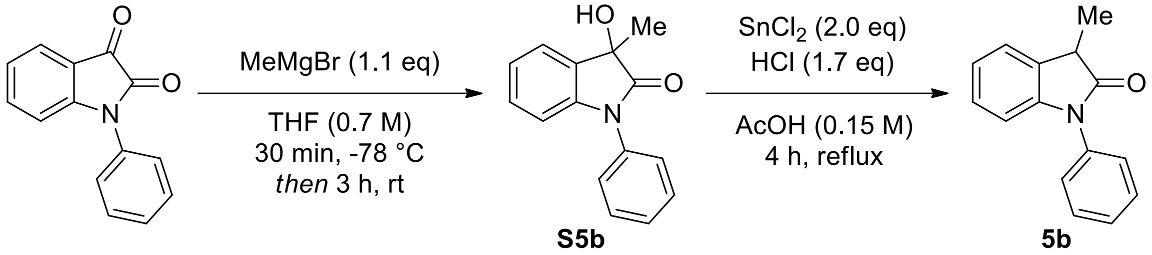

The general procedure **B** was followed with 1-phenylisatin (500 mg, 2.24 mmol) and methylmagnesiumbromide (3 M in Et_2O, 0.82 mL, 1.83 mmol, 1.1 equiv.) to afford **S5b** as a light yellow solid (437 mg, 1.83 mmol, 82%). **^{1}H NMR (500 MHz, DMSO-d$_6$):** δ 7.60-7.55 (m, 2H), 7.48-7.43 (m, 2H), 7.43-7.39 (m, 2H), 7.25 (td, J = 7.7, 1.3 Hz, 1H), 7.11 (td, J = 7.5, 0.8 Hz, 1H), 6.72 (d, J = 7.8 Hz, 1H), 6.15 (s, 1H), 1.51 (s, 3H). **^{13}C{^{1}H} NMR (126 MHz, DMSO-d$_6$):** δ 177.4 (C=O), 142.3 (C), 134.3 (C), 132.9 (C), 129.7 (2CH), 129.0 (CH), 128.0 (CH), 126.6 (2CH), 123.7 (CH), 123.1 (CH), 109.0 (CH), 72.5 (C), 24.7 (CH_3). **HRMS (ESI$^+$):** *m/z* calcd. for $C_{15}H_{13}NO_2Na$ $[M+Na]^+$ 262.0839, found 262.0838. Spectral and physical data are in accordance with literature.[14]

The general procedure **C** was followed with **S5b** (250 mg, 1.04 mmol) Purification was performed with flash column chromatography over silica gel (40 g SiO_2, heptane/EtOAc, 100/0 to 70/30, gradient) to afford **5b** as a colorless oil (200 mg, 0.896 mmol, 86%). **^{1}H NMR (600 MHz, $CDCl_3$):** δ 7.53 (t, J = 7.8 Hz, 2H), 7.44-7.39 (m, 3H), 7.31 (d, J = 7.3 Hz, 1H), 7.20 (t, J = 7.7 Hz, 1H), 7.10 (t, J = 7.5 Hz, 1H), 6.82 (d, J = 7.9 Hz, 1H), 3.63 (d, J = 7.6 Hz, 1H), 1.60 (d, J = 7.6 Hz, 3H). **^{13}C{^{1}H} NMR (150 MHz, $CDCl_3$):** δ 178.1 (C=O), 144.1 (C), 134.7 (C), 130.6 (C), 129.7 (2CH), 128.1 (CH), 127.9 (CH), 126.7 (2CH), 123.9 (CH), 123.0 (CH), 109.4 (CH), 40.9 (CH), 15.8 (CH_3). **HRMS (ESI$^+$):** *m/z* calcd. for $C_{15}H_{13}NONa$ $[M+Na]^+$ 246.0890, found 246.0892. Spectral and physical data are in accordance with literature.[13]

***Tert-butyl 2-oxo-3-phenylindoline-1-carboxylate* 5c**

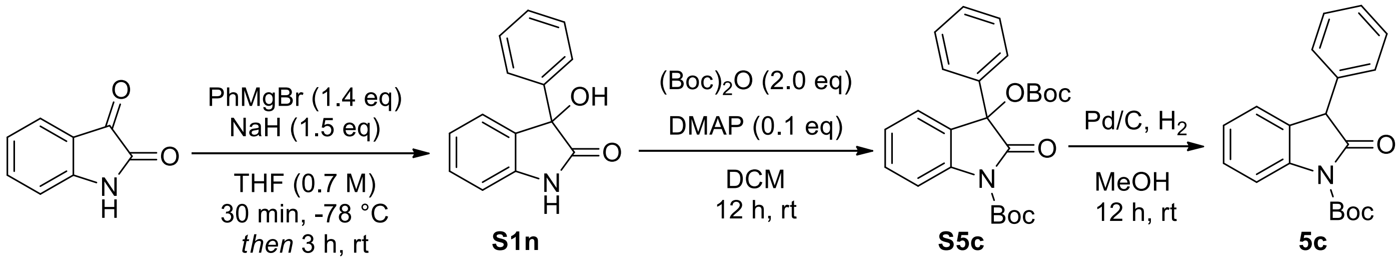

Following reported procedure,[14] **S1n** (500 mg, 2.22 mmol) was dissolved in DCM (22 mL). To this solution were added DMAP (27.1 mg, 0.220 mmol) and $(Boc)_2O$ (969 mg, 4.44 mmol) at rt, and then the mixture was stirred for 12 h. The reaction mixture was diluted with ethyl acetate, and then quenched with saturated aqueous NH_4Cl. The aqueous layer was extracted with ethyl acetate, and the combined organic layers were washed with H_2O and brine and then dried over $MgSO_4$. After the removal of solvent, purification by FC (gradient, heptane/ethyl acetate, 0-10%) gave **S5c** (852 mg, 1.99 mmol, 90%) as a white solid. **^{1}H NMR (700 MHz, $CDCl_3$):** δ 7.99 (d, J = 8.2 Hz, 1H), 7.45 (td, J = 8.2, 1.4 Hz, 1H), 7.35-7.29 (m, 6H), 7.24 (td, J = 7.5, 0.7 Hz, 1H), 1.61 (s, 9H), 1.38 (s, 9H). **^{13}C{^{1}H} NMR (176 MHz, $CDCl_3$):** δ 171.7 (C=O), 151.1 (C=O), 149.2 (C=O), 140.8 (C), 136.1 (C), 130.5 (CH), 129.3 (CH), 128.7 (2CH), 127.5 (C),

126.9 (2CH), 125.1 (CH), 124.2 (CH), 115.6 (CH), 84.7 (C), 84.1 (C), 81.8 (C), 28.2 ($3CH_3$), 27.7 ($3CH_3$). **HRMS (ESI$^+$):** *m/z* calcd. for $C_{24}H_{27}NO_6Na$ $[M+Na]^+$ 448.1731, found 448.1730.

S5c (850 mg, 1.99 mmol) was dissolved in MeOH (9 mL). Pd/C (423 mg, 0.400 mmol, 0.20 equiv.) was added to this solution, and the resulting mixture was stirred under hydrogen atmosphere (balloon) for 12 h at rt. The reaction mixture was filtered through celite to remove Pd/C, and the residue was washed with Et_2O. After the removal of solvent, the crude product was purified by FC (gradient, heptane/ethyl acetate, 0-15%) to give **5c** (300 mg, 0.97 mmol, 56%) as a white solid. **^{1}H NMR (700 MHz, $CDCl_3$):** δ 7.93 (d, J = 8.2 Hz, 1H), 7.38-7.33 (m, 3H), 7.32-7.29 (m, 1H), 7.21-7.19 (m, 2H), 7.18-7.15 (m, 1H), 4.73 (s, 1H), 1.63 (s, 9H). **^{13}C{^{1}H} NMR (175 MHz, $CDCl_3$):** δ 174.0 (C=O), 149.5 (C=O), 140.6 (C), 136.4 (C), 129.0 (2CH), 128.8 (3CH), 128.0 (CH), 127.5 (C), 125.2 (CH), 124.7 (CH), 115.2 (CH), 84.5 (C), 52.7 (CH), 28.2 ($3CH_3$). **HRMS (ESI$^+$):** *m/z* calcd. for $C_{19}H_{19}NO_3Na$ $[M+Na]^+$ 332.1258, found 332.1256. Spectral and physical data are in accordance with literature.[14]

6. Characterization data of 3,3-substituted oxindoles 2a-s, 4a-f, 6a-c and 7a-i

***3-Ethoxy-3-methylindolin-2-one* 2a**

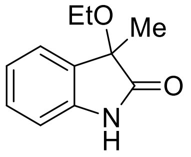

2a

General procedure **A** was followed with 3-methyloxindole (61.3 mg, 0.40 mmol) and ethanol (2.5 mL). Purification was performed with flash column chromatography over silica gel (20 g SiO_2, heptane/EtOAc, 100/0 to 50/50, gradient) to afford **2a** as a light yellow solid (43.3 mg, 0.226 mmol, 57%). **^{1}H NMR (600 MHz, $CDCl_3$):** δ 8.88 (brs, 1H), 7.32 (d, J = 7.3 Hz, 1H), 7.29-7.26 (m, 1H), 7.09 (t, J = 7.5 Hz, 1H), 6.94 (d, J = 7.7 Hz, 1H), 3.28 (tt, J = 14.0, 7.0 Hz, 1H), 3.13 (dq, J = 14.0, 7.0 Hz, 1H), 1.59 (s, 3H), 1.16 (t, J = 7.0 Hz, 3H). **^{13}C{^{1}H} NMR (150 MHz, $CDCl_3$):** δ 179.7 (C=O), 140.6 (C), 129.9 (C), 129.7 (CH), 124.1 (CH), 123.2 (CH), 110.6 (CH), 79.7 (C), 61.3 (CH_2), 24.4 (CH_3), 15.5 (CH_3). **FT-IR (neat, cm^{-1}):** 3229, 2976, 2927, 1714, 1618, 1605, 1470, 1204, 1128. **HRMS (ESI$^+$):** *m/z* calcd. for $C_{11}H_{13}NO_2Na$ $[M+Na]^+$ 214.0838, found 214.0840.

***3-Ethoxyindolin-2-one* 2b**

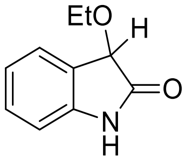

2b

General procedure **A** was followed with oxindole (53.3 mg, 0.40 mmol) and ethanol (2.5 mL). Purification was performed with flash column chromatography over silica gel (20 g SiO_2, heptane/EtOAc, 100/0 to 50/50, gradient) to afford **2b** as a light yellow solid (7.3 mg, 0.042 mmol, 11%). **^{1}H NMR (400 MHz, $CDCl_3$):** δ 7.94 (brs, 1H), 7.38 (d, J = 7.4 Hz, 1H), 7.30-7.24 (m, 1H), 7.06 (t, J = 7.4 Hz, 1H), 6.85 (d, J = 7.8 Hz, 1H), 4.93 (s, 1H), 3.88 (dq, J = 14.1, 7.0 Hz, 1H), 3.70 (dq, J = 14.1, 7.0 Hz, 1H), 1.29 (t, J = 7.0 Hz, 3H). **^{13}C{^{1}H} NMR (100 MHz, $CDCl_3$):** δ 176.8 (C=O), 141.3 (C), 130.0 (CH), 125.9 (C), 125.8 (CH), 123.1 (CH), 110.2 (CH), 76.1 (CH), 64.6 (CH_2), 15.5 (CH_3). **FT-IR (neat, cm^{-1}):** 3132, 3083, 2961, 2926, 1703, 1617, 1470, 1259. **HRMS (ESI$^+$):** *m/z* calcd. for $C_{10}H_{11}NO_2Na$ $[M+Na]^+$ 200.0682, found 200.0678.

3-Ethoxy-3-isopropylindolin-2-one **2c**

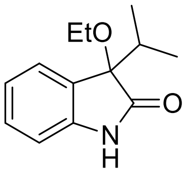

2c

General procedure **A** was followed with **1c** (70.1 mg, 0.40 mmol) and ethanol (2.5 mL). Purification was performed with flash column chromatography over silica gel (20 g SiO_2, heptane/EtOAc, 100/0 to 50/50, gradient) to afford **2c** as a yellow solid (25.0 mg, 0.114 mmol, 29%). **^{1}H NMR (400 MHz, $CDCl_3$):** δ 8.02 (brs, 1H), 7.32-7.24 (m, 2H), 7.06 (t, *J* = 7.5 Hz, 1H), 6.87 (d, *J* = 7.7 Hz, 1H), 3.26 (dq, *J* = 14.0, 7.0 Hz, 1H), 3.10 (dq, *J* = 14.0, 7.0 Hz, 1H), 2.35-2.25 (m, 1H), 1.14 (t, *J* = 7.0 Hz, 3H), 1.04 (d, *J* = 6.8 Hz, 3H), 0.79 (d, *J* = 6.8 Hz, 3H). **^{13}C{^{1}H} NMR (100 MHz, $CDCl_3$):** δ 178.9 (C=O), 141.4 (C), 129.5 (CH), 127.3 (C), 125.4 (CH), 122.7 (CH), 110.0 (CH), 85.8 (C), 61.2 (CH_2), 35.8 (CH), 16.2 (CH_3), 16.2 (CH_3), 15.6 (CH_3). **FT-IR (neat, cm^{-1}):** 3152, 3109, 2966, 2923, 1733, 1687, 1617, 1466, 1213. **HRMS (ESI$^+$):** *m/z* calcd. for $C_{13}H_{17}NO_2Na$ [M+Na]$^+$ 242.1151, found 242.1152.

3-Benzyl-3-ethoxyindolin-2-one **2d**

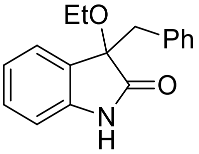

2d

General procedure **A** was followed with **1d** (89.3 mg, 0.40 mmol) and ethanol (2.5 mL). Purification was performed with flash column chromatography over silica gel (20 g SiO_2, heptane/EtOAc, 100/0 to 50/50, gradient) to afford **2d** as a light yellow solid (35.0 mg, 0.131 mmol, 33%). **^{1}H NMR (400 MHz, $CDCl_3$):** δ 8.53 (brs, 1H), 7.21 (td, *J* = 7.5, 1.7 Hz, 1H), 7.13-7.01 (m, 5H), 6.96-6.92 (m, 2H), 6.75 (dd, *J* = 7.6, 3.7 Hz, 1H), 3.22 (d, *J* = 12.9 Hz, 1H), 3.29-3.20 (m, 2H), 3.13 (dd, *J* = 13.8, 3.7 Hz, 1H), 1.17 (t, *J* = 7.0 Hz, 3H). **^{13}C{^{1}H} NMR (100 MHz, $CDCl_3$):** δ 178.8 (C=O), 141.1 (C), 134.1 (C), 130.7 (2CH), 129.8 (CH), 127.7 (2CH), 127.4 (C), 126.8 (CH), 125.3 (CH), 122.7 (CH), 110.3 (CH), 83.7 (C), 61.4 (CH_2), 44.0 (CH_2), 15.5 (CH_3). **HRMS (ESI$^+$):** *m/z* calcd. for $C_{17}H_{17}NO_2Na$ [M+Na]$^+$ 290.1152, found 290.1153. Spectral and physical data are in accordance with literature.[15]

3-Allyl-3-ethoxyindolin-2-one **2e**

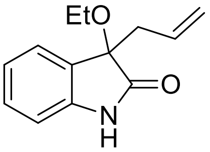

2e

General procedure **A** was followed with **1e** (69.3 mg, 0.40 mmol) and ethanol (2.5 mL). Purification was performed with flash column chromatography over silica gel (20 g SiO_2, heptane/EtOAc, 100/0 to 50/50, gradient) to afford **2e** as a light yellow solid (26.6 mg, 0.122 mmol, 31%). **^{1}H NMR (400 MHz, $CDCl_3$):** δ 8.96 (brs, 1H), 7.32-7.24 (m, 2H), 7.08 (t, J = 7.4 Hz, 1H), 6.92 (d, J = 7.7 Hz, 1H), 5.66-5.45 (m, 1H), 5.05-4.96 (m, 2H), 3.28 (dq, J = 14.0, 7.0 Hz, 1H), 3.15 (dq, J = 14.0, 7.0 Hz, 1H), 2.77 (dd, J = 13.3, 6.4 Hz, 1H), 2.64 (dd, J = 13.3, 8.2 Hz, 1H), 1.15 (t, J = 7.0 Hz, 3H). **^{13}C{^{1}H} NMR (100 MHz, $CDCl_3$):** δ 178.9 (C=O), 141.1 (C), 130.7 (CH), 129.8 (CH), 128.0 (C), 124.9 (CH), 123.0 (CH), 119.8 (CH_2), 110.5 (CH), 82.6 (C), 61.2 (CH_2), 42.3 (CH_2), 15.5 (CH_3). **FT-IR (neat, cm^{-1}):** 3164, 2971, 2903, 1730, 1697, 1618, 1603, 1469, 1231. **HRMS (ESI$^+$):** *m/z* calcd. for $C_{13}H_{15}NO_2Na$ $[M+Na]^+$ 240.0995, found 240.0996.

***3-Cyclopentyl-3-ethoxyindolin-2-one* 2f**

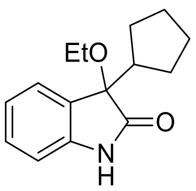

2f

General procedure **A** was followed with **1f** (80.5 mg, 0.40 mmol) and ethanol (2.5 mL). Purification was performed with flash column chromatography over silica gel (20 g SiO_2, heptane/EtOAc, 100/0 to 50/50, gradient) to afford **2f** as a light yellow solid (48.5 mg, 0.198 mmol, 49%). **^{1}H NMR (400 MHz, $CDCl_3$):** δ 8.18 (brs, 1H), 7.32 (d, J = 7.4 Hz, 1H), 7.28-7.22 (m, 1H), 7.05 (td, J = 7.6, 0.9 Hz, 1H), 6.87 (d, J = 7.6 Hz, 1H), 3.26 (dq, J = 14.0, 7.0 Hz, 1H), 3.12 (dq, J = 14.0, 7.0 Hz, 1H), 2.55-2.41 (m, 1H), 1.78-1.70 (m, 1H), 1.62-1.56 (m, 1H), 1.54-1.42 (m, 4H), 1.28-1.22 (m, 1H), 1.14 (t, J = 7.0 Hz, 3H), 0.90-0.82 (m, 1H). **^{13}C{^{1}H} NMR (100 MHz, $CDCl_3$):** δ 179.1 (C=O), 141.2 (C), 129.5 (CH), 128.2 (C), 125.3 (CH), 122.7 (CH), 110.1 (CH), 84.8 (C), 61.4 (CH_2), 47.3 (CH), 26.6 (CH_2), 26.3 (CH_2), 25.4 (CH_2), 25.3 (CH_2), 15.6 (CH_3). **FT-IR (neat, cm^{-1}):** 3149, 3090, 2950, 2874, 1725, 1684, 1618, 1599, 1467, 1212, 1115, 1089. **HRMS (ESI$^+$):** *m/z* calcd. for $C_{15}H_{19}NO_2Na$ $[M+Na]^+$ 268.1308, found 268.1307.

***3-Ethoxy-3-(pent-4-en-1-yl)indolin-2-one* 2g**

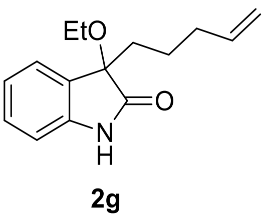

2g

General procedure **A** was followed with **1g** (80.5 mg, 0.40 mmol) and ethanol (2.5 mL). Purification was performed with flash column chromatography over silica gel (20 g SiO_2, heptane/EtOAc, 100/0 to 50/50, gradient) to afford **2g** as a light yellow solid (27.1 mg, 0.110 mmol, 28%). **^{1}H NMR (400 MHz, $CDCl_3$):** δ 7.92 (brs, 1H), 7.33-7.24 (m, 2H), 7.08 (td, J = 7.7, 1.1 Hz, 1H), 6.87 (d, J = 7.7 Hz, 1H), 5.68 (ddt, J = 16.9, 10.2, 6.7 Hz, 1H), 5.02-4.81

(m, 2H), 3.26 (dq, J = 14.0, 7.0 Hz, 1H), 3.12 (dq, J = 14.0, 7.0 Hz, 1H), 2.03-1.90 (m, 4H), 1.40-1.30 (m, 1H), 1.24-1.17 (m, 1H), 1.14 (t, J = 7.0 Hz, 3H). **$^{13}C\{^1H\}$ NMR (100 MHz, $CDCl_3$):** δ 178.6 (C=O), 140.9 (C), 138.2 (CH), 129.7 (CH), 128.7 (C), 124.7 (CH), 123.1 (CH), 115.1 (CH_2), 110.2 (CH), 82.7 (C), 61.1 (CH_2), 37.6 (CH_2), 33.8 (CH_2), 22.2 (CH_2), 15.6 (CH_3). **FT-IR (neat, cm^{-1}):** 3230, 2975, 2923, 1716, 1619, 1470, 1209. HRMS **(ESI^+):** m/z calcd. for $C_{15}H_{19}NO_2Na$ $[M+Na]^+$ 268.1308, found 268.1308.

***3-Ethoxy-3-pentylindolin-2-one* 2h**

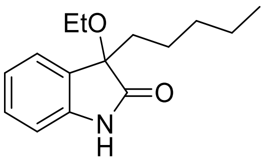

2h

General procedure **A** was followed with **1h** (81.3 mg, 0.40 mmol) and ethanol (2.5 mL) Purification was performed with flash column chromatography over silica gel (20 g SiO_2, heptane/EtOAc, 100/0 to 50/50, gradient) to afford **2h** as a light brown solid (33.1 mg, 0.134 mmol, 34%). **^1H NMR (400 MHz, $CDCl_3$):** δ 8.08 (brs, 1H), 7.30-7.24 (m, 2H), 7.09 (t, J = 7.5 Hz, 1H), 6.91-6.85 (m, 1H), 3.26 (dq, J = 14.0, 7.0 Hz, 1H), 3.12 (dq, J = 14.1, 7.0 Hz, 1H), 1.95 (dd, J = 8.6, 4.4 Hz, 2H), 1.64-1.60 (m, 1H), 1.23-1.18 (m, 4H), 1.14 (t, J = 7.0 Hz, 3H), 1.10-1.01 (m, 1H), 0.80 (t, J = 6.6 Hz, 3H). **$^{13}C\{^1H\}$ NMR (100 MHz, $CDCl_3$):** δ 179.0 (C=O), 141.0 (C), 129.6 (CH), 128.8 (C), 124.6 (CH), 123.1 (CH), 110.2 (CH), 82.9 (C), 61.1 (CH_2), 38.0 (CH_2), 31.9 (CH_2), 22.4 ($2CH_2$), 15.6 (CH_3), 14.1 (CH_3). **FT-IR (neat, cm^{-1}):** 2955, 2926, 2871, 1716, 1618, 1469, 1211, 1188. **HRMS (ESI^+):** m/z calcd. for $C_{15}H_{21}NO_2Na$ $[M+Na]^+$ 270.1465, found 270.1463.

***3-((1,3-dioxolan-2-yl)methyl)-3-ethoxyindolin-2-one* 2i**

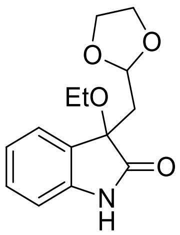

2i

General procedure **A** was followed with **1i** (87.7 mg, 0.40 mmol) and ethanol (2.5 mL) Purification was performed with flash column chromatography over silica gel (20 g SiO_2, heptane/EtOAc, 100/0 to 50/50, gradient) to afford **2i** as a white solid (26.0 mg, 0.10 mmol, 25%). **^1H NMR (400 MHz, $CDCl_3$):** δ 7.51 (brs, 1H), 7.35 (d, J = 7.4 Hz, 1H), 7.27 (td, J = 7.7, 1.1 Hz, 1H), 7.08 (t, J = 7.5 Hz, 1H), 6.85 (d, J = 7.7 Hz, 1H), 4.78 (dd, J = 6.0, 4.0 Hz, 1H), 3.86-3.80 (m, 1H), 3.79-3.74 (m, 1H), 3.73-3.67 (m, 2H), 3.26 (dq, J = 14.1, 7.0 Hz, 1H), 2.74 (dq, J = 14.1, 7.0 Hz, 1H), 2.43-2.40 (m, 2H), 1.13 (t, J = 7.0 Hz, 3H). **$^{13}C\{^1H\}$ NMR (100 MHz, $CDCl_3$):** δ 177.4 (C=O), 141.2 (C), 130.0 (CH), 127.5 (C), 125.4 (CH), 123.0 (CH), 110.1 (CH), 101.0 (CH), 79.7 (C), 64.8 (CH_2), 64.7 (CH_2), 60.3 (CH_2), 41.7 (CH_2), 15.3 (CH_3).

FT-IR (neat, cm^{-1}): 3263, 2974, 2927, 2887, 1725, 1619, 1471, 1193, 1107, 1085, 1048. **HRMS (ESI$^+$):** *m/z* calcd. for $C_{14}H_{17}NO_4Na$ [M+Na]$^+$ 286.1050, found 286.1049.

***2-(3-Ethoxy-2-oxoindolin-3-yl)acetonitrile* 2j**

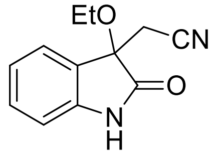

2j

General procedure **A** was followed with **1j** (68.9 mg, 0.40 mmol) and ethanol (2.5 mL) Purification was performed with flash column chromatography over silica gel (20 g SiO_2, heptane/EtOAc, 100/0 to 50/50, gradient) to afford **2j** as a light brown solid (20.5 mg, 0.095 mmol, 24%). **^{1}H NMR (400 MHz, $CDCl_3$):** δ 8.60 (brs, 1H), 7.56 (d, J = 7.5 Hz, 1H), 7.37 (td, J = 7.7, 1.1 Hz, 1H), 7.17 (td, J = 7.6, 0.7 Hz, 1H), 6.96 (dd, J = 7.7, 2.2 Hz, 1H), 3.34.3.23 (m, 1H), 3.23-3.15 (m, 1H), 3.06 (d, J = 16.4 Hz, 1H), 2.74 (d, J = 16.4 Hz, 1H), 1.17 (t, J = 7.0 Hz, 3H). **^{13}C{^{1}H} NMR (100 MHz, $CDCl_3$):** δ 175.8 (C=O), 140.7 (C), 131.2 (CH), 125.3 (C), 125.2 (CH), 123.9 (CH), 115.5 (C), 111.1 (CH), 78.1 (C), 62.0 (CH_2), 27.0 (CH_2), 15.3 (CH_3). **FT-IR (neat, cm^{-1})**: 3230, 2971, 2932, 1738, 1698, 1618, 1469, 1227, 1115, 1045. **HRMS (ESI$^+$):** *m/z* calcd. for $C_{12}H_{12}N_2O_2Na$ [M+Na]$^+$ 239.0791, found 239.0787.

***Ethyl 2-(3-ethoxy-2-oxoindolin-3-yl)acetate* 2k**

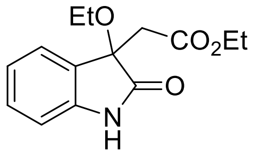

2k

General procedure **A** was followed with **1k** (87.7 mg, 0.40 mmol) and ethanol (2.5 mL) Purification was performed with flash column chromatography over silica gel (20 g SiO_2, heptane/EtOAc, 100/0 to 50/50, gradient) to afford **2k** as a light yellow solid (36.0 mg, 0.137 mmol, 34%). **^{1}H NMR (400 MHz, $CDCl_3$):** δ 8.80 (brs, 1H), 7.33 (d, J = 7.4 Hz, 1H), 7.27 (td, J = 7.7, 1.1 Hz, 1H), 7.06 (td, J = 7.7, 0.7 Hz, 1H), 6.90 (d, J = 7.8, Hz, 1H), 3.98-3.83 (m, 2H), 3.29 (dd, J = 8.4, 7.0 Hz, 1H), 3.20 (d, J = 15.4 Hz, 1H), 3.14-3.03 (m, 2H), 1.11 (t, J = 7.0 Hz, 3H), 1.01 (t, J = 7.1 Hz, 3H). **^{13}C{^{1}H} NMR (100 MHz, $CDCl_3$):** δ 177.6 (C=O), 168.8 (C=O), 142.1 (C), 130.3 (CH), 126.9 (C), 124.8 (CH), 123.0 (CH), 110.5 (CH), 79.2 (C), 60.8 (CH_2), 60.4 (CH_2), 42.1 (CH_2), 15.4 (CH_3), 13.9 (CH_3). **FT-IR (neat, cm^{-1}):** 3328, 3169, 2976, 2928, 1736, 1714, 1618, 1470, 1371, 1342, 1188. **HRMS (ESI$^+$):** *m/z* calcd. for $C_{14}H_{17}NO_4Na$ [M+Na]$^+$ 286.1050, found 286.1044.

***3-Ethoxy-3-(3-methoxyphenyl)indolin-2-one* 2l**

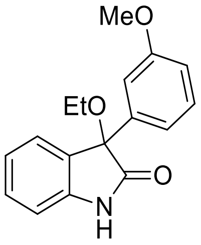

2l

General procedure **A** was followed with **1l** (95.7 mg, 0.40 mmol) and ethanol (2.5 mL) Purification was performed with flash column chromatography over silica gel (20 g SiO_2, heptane/EtOAc, 100/0 to 50/50, gradient) to afford **2l** as a light yellow solid (28.0 mg, 0.099 mmol, 25%). **^{1}H NMR (400 MHz, $CDCl_3$):** δ 8.76 (brs, 1H), 7.30 (t, *J* = 7.7 Hz, 1H), 7.25-7.18 (m, 2H), 7.11-7.05 (m, 2H), 6.96-6.90 (m, 2H), 6.83 (dd, *J* = 8.2, 2.0 Hz, 1H), 3.78 (s, 3H), 3.50 (dq, *J* = 14.1, 7.0 Hz, 1H), 3.34 (dq, *J* = 14.1, 7.0 Hz, 1H), 1.26 (t, *J* = 7.0 Hz, 3H). **^{13}C{^{1}H} NMR (100 MHz, $CDCl_3$):** δ 178.1 (C=O), 159.8 (C), 141.6 (C), 140.5 (C), 130.1 (CH), 129.5 (CH), 129.3 (C), 126.0 (CH), 123.4 (CH), 118.6 (CH), 113.9 (CH), 112.3 (CH), 110.7 (CH), 83.9 (C), 61.3 (CH_2), 55.4 (CH_3), 15.6 (CH_3). **FT-IR (neat, cm^{-1}):**.3150, 3091, 2950, 2874, 1725, 1684, 1618, 1467, 1212, 1152. **HRMS (ESI$^+$):** *m/z* calcd. for $C_{17}H_{17}NO_3Na$ $[M+Na]^+$ 306.1101, found 306.1097.

***3-Ethoxy-3-(4-methoxyphenyl)indolin-2-one* 2m**

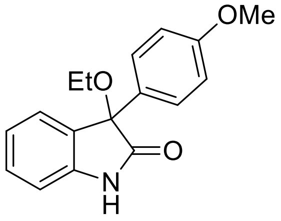

2m

General procedure **A** was followed with **1m** (95.7 mg, 0.40 mmol) and ethanol (2.5 mL) Purification was performed with flash column chromatography over silica gel (20 g SiO_2, heptane/EtOAc, 100/0 to 50/50, gradient) to afford **2m** as a light yellow solid (78.7 mg, 0.278 mmol, 69%). **^{1}H NMR (400 MHz, $CDCl_3$):** δ 8.88 (brs, 1H), 7.34 (d, *J* = 8.9 Hz, 2H), 7.30 (t, *J* = 7.8 Hz, 1H), 7.25 (d, *J* = 8.3 Hz, 1H), 7.10 (t, *J* = 7.5 Hz, 1H), 6.94 (d, *J* = 7.8 Hz, 1H), 6.85 (d, *J* = 8.9 Hz, 2H), 3.77 (s, 3H), 3.48 (dq, *J* = 14.0, 7.0 Hz, 1H), 3.31 (dq, *J* = 14.0, 7.0 Hz, 1H), 1.25 (t, *J* = 7.0 Hz, 3H). **^{13}C{^{1}H} NMR (100 MHz, $CDCl_3$):** δ 178.4 (C=O), 159.8 (C), 141.6 (C), 131.0 (C), 130.0 (CH), 129.2 (C), 127.8 (2CH), 126.0 (CH), 123.3 (CH), 114.0 (2CH), 110.7 (CH), 83.6 (C), 61.2 (CH_2), 55.4 (CH_3), 15.6 (CH_3). **FT-IR (neat, cm^{-1}):** 3170, 2975, 2878, 1721, 1690, 1618, 1608, 1507, 1471, 1259, 1217, 1182. **HRMS (ESI$^+$):** *m/z* calcd. for $C_{17}H_{17}NO_3Na$ $[M+Na]^+$ 306.1101, found 306.1091.

***3-Ethoxy-3-phenylindolin-2-one* 2n**

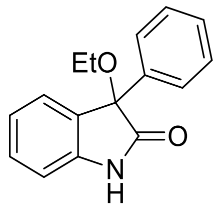

2n

General procedure **A** was followed with **1n** (83.7 mg, 0.40 mmol) and ethanol (2.5 mL) Purification was performed with flash column chromatography over silica gel (20 g SiO_2, heptane/EtOAc, 100/0 to 50/50, gradient) to afford **2n** as a light yellow solid (51.0 mg, 0.201 mmol, 50%). **^{1}H NMR (600 MHz, $CDCl_3$):** δ 9.16 (brs, 1H), 7.43-7.40 (m, 2H), 7.33-7.27 (m, 4H), 7.23 (d, J = 7.4 Hz, 1H), 7.09 (dd, J = 11.0, 3.9 Hz, 1H), 6.96 (d, J = 7.8 Hz, 1H), 3.50 (dq, J = 14.0, 7.0 Hz, 1H), 3.34 (dq, J = 14.0, 7.0 Hz, 1H), 1.26 (t, J = 7.0 Hz, 3H). **^{13}C{^{1}H} NMR (150 MHz, $CDCl_3$):** δ 178.5 (C=O), 141.7 (C), 139.0 (C), 130.1 (CH), 129.3 (C), 128.6 (2CH), 128.5 (CH), 126.3 (2CH), 126.0 (CH), 123.4 (CH), 110.8 (CH), 84.0 (C), 61.2 (CH_2), 15.5 (CH_3). **HRMS (ESI$^+$):** *m/z* calcd. for $C_{16}H_{15}NO_2Na$ $[M+Na]^+$ 276.0995, found 276.0993. Spectral and physical data are in accordance with literature.[16]

***3-Ethoxy-3-(p-tolyl)indolin-2-one* 2o**

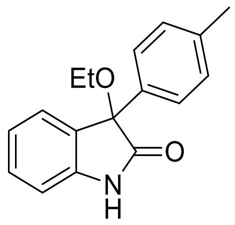

2o

General procedure **A** was followed with **1o** (89.3 mg, 0.40 mmol) and ethanol (2.5 mL) Purification was performed with flash column chromatography over silica gel (20 g SiO_2, heptane/EtOAc, 100/0 to 50/50, gradient) to afford **2o** as a light yellow solid (43.7 mg, 0.163 mmol, 41%). **^{1}H NMR (400 MHz, $CDCl_3$):** δ 9.24 (brs, 1H), 7.33-7.27 (m, 3H), 7.23 (d, J = 7.4 Hz, 1H), 7.13 (d, J = 8.1 Hz, 2H), 7.08 (t, J = 7.4 Hz, 1H), 6.94 (d, J = 7.8 Hz, 1H), 3.49 (dq, J = 14.1, 7.0 Hz, 1H), 3.33 (dq, J = 14.1, 7.0 Hz, 1H), 2.31 (s, 3H), 1.26 (t, J = 7.0 Hz, 3H). **^{13}C{^{1}H} NMR (100 MHz, $CDCl_3$):** δ 178.7 (C=O), 141.7 (C), 138.3 (C), 136.0 (C), 130.0 (CH), 129.4 (C), 129.3 (2CH), 126.3 (2CH), 125.9 (CH), 129.3 (CH), 110.8 (CH), 83.9 (C), 61.2 (CH_2), 21.3 (CH_3), 15.6 (CH_3). **FT-IR (neat, cm^{-1}):** 3231, 2975, 1714, 1616, 1469, 1207, 1175. **HRMS (ESI$^+$):** *m/z* calcd. for $C_{17}H_{17}NO_2Na$ $[M+Na]^+$ 290.1152, found 290.1152.

***3-(4-(Tert-butyl)phenyl)-3-ethoxyindolin-2-one* 2p**

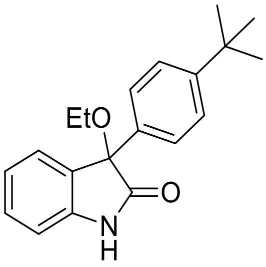

2p

General procedure **A** was followed with **1p** (106.0 mg, 0.40 mmol) and ethanol (2.5 mL) Purification was performed with flash column chromatography over silica gel (20 g SiO_2, heptane/EtOAc, 100/0 to 50/50, gradient) to afford **2p** as a light yellow solid (46.7 mg, 0.151 mmol, 38%). **^{1}H NMR (400 MHz, $CDCl_3$):** δ 9.10 (brs, 1H), 7.37-7.32 (m, 4H), 7.31-7.27 (m, 1H), 7.27-7.24 (m, 1H), 7.09 (t, J = 7.5 Hz, 1H), 6.95 (d, J = 7.8 Hz, 1H), 3.50 (dq, J = 14.1, 7.0 Hz, 1H), 3.34 (dq, J = 14.1, 7.0 Hz, 1H), 1.28 (s, 9H), 1.25 (t, J = 7.0 Hz, 3H). **^{13}C{^{1}H} NMR (100 MHz, $CDCl_3$):** δ 178.6 (C=O), 151.4 (C), 141.7 (C), 135.9 (C), 130.0 (CH), 129.3 (C), 126.1 (2CH), 126.1 (CH), 125.3 (2CH), 123.3 (CH), 110.8 (CH), 83.9 (C), 61.2 (CH_2), 34.6 (C), 31.4 ($3CH_3$), 15.6 (CH_3). **FT-IR (neat, cm^{-1}):** 2960, 1719, 1618, 1469, 1218, 1193. **HRMS (ESI$^+$):** *m/z* calcd. for $C_{20}H_{23}NO_2Na$ [M+Na]$^+$ 332.1621, found 332.1622.

***3-([1,1'-Biphenyl]-4-yl)-3-ethoxyindolin-2-one* 2q**

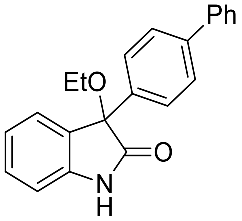

2q

General procedure **A** was followed with **1q** (114.0 mg, 0.40 mmol), ethanol (2.5 mL) in THF as solvent (2.5 mL) Purification was performed with flash column chromatography over silica gel (20 g SiO_2, heptane/EtOAc, 100/0 to 50/50, gradient) to afford **2q** as a light yellow solid (60.0 mg, 0.182 mmol, 46%). **^{1}H NMR (400 MHz, $CDCl_3$) δ** 7.99 (s, 1H), 7.54 (m, 4H), 7.48 (d, J = 8.4 Hz, 2H), 7.41 (t, J = 7.6 Hz, 2H), 7.36-7.28 (m, 3H), 7.13 (td, J = 7.5, 0.8 Hz, 1H), 6.96 (d, J = 7.8 Hz, 1H), 3.53 (dq, J = 14.0, 7.0 Hz, 1H), 3.37 (dq, J = 14.0, 7.0 Hz, 1H), 1.28 (t, J = 7.0 Hz, 3H). **^{13}C{^{1}H} NMR (100 MHz, $CDCl_3$) δ** 177.4 (C=O), 141.5 (C), 141.4 (C), 140.9 (C), 138.0 (C), 130.2 (CH), 129.2 (C), 128.9 (2CH), 127.5 (CH), 127.4 (2CH), 127.3 (2CH), 126.9 (2CH), 126.2 (CH), 123.5 (CH), 110.5 (CH), 83.7 (C), 61.3 (CH_2), 15.6 (CH_3). **FT-IR (neat, cm^{-1}):** 3229, 2923, 2852, 1717, 1617, 1469, 1177, 1105. **HRMS (ESI$^+$):** *m/z* calcd. for $C_{22}H_{19}NO_2Na$ [M+Na]$^+$ 352.1308, found 352.1289.

***3-Ethoxy-3-(4-fluorophenyl)indolin-2-one* 2r**

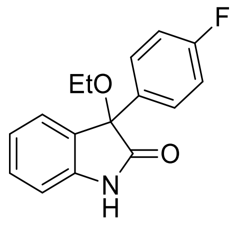

2r

General procedure **A** was followed with **1r** (90.9 mg, 0.40 mmol) and ethanol (2.5 mL) Purification was performed with flash column chromatography over silica gel (20 g SiO_2, heptane/EtOAc, 100/0 to 50/50, gradient) to afford **2r** as a light yellow solid (77 mg, 0.284 mmol, 71%). **^{1}H NMR (400 MHz, $CDCl_3$):** δ 8.71 (s, 1H), 7.43-7.36 (m, 2H), 7.32 (t, J = 7.7 Hz, 1H), 7.22 (d, J = 7.4 Hz, 1H), 7.11 (t, J = 7.5 Hz, 1H), 7.00 (t, J = 8.6 Hz, 2H), 6.95 (d, J = 7.9 Hz, 1H), 3.47 (dq, J = 14.0, 7.0 Hz, 1H), 3.31 (dq, J = 14.0, 7.0 Hz, 1H), 1.25 (t, J = 6.9 Hz, 3H). **^{13}C{^{1}H} NMR (100 MHz, $CDCl_3$):** δ 178.0 (C=O), 162.9 (d, J^{CF} = 247.1 Hz, C), 141.6 (C), 134.8 (d, J^{CF} = 247.1 Hz, C), 130.3 (CH), 128.9 (C), 128.4 (CH), 128.3 (CH), 126.0 (CH), 123.6 (CH), 115.4 (d, J^{CF} = 21.4 Hz, 2CH), 110.8 (CH), 83.5 (C), 61.3 (CH_2), 15.5 (CH_3). **^{19}F NMR (376 MHz, $CDCl_3$):** δ -113.83. **FT-IR (neat, cm^{-1}):** 3375, 3282, 2975, 2930, 2882, 1720, 1652, 1560, 1437, 1245, 1221. **HRMS (ESI$^+$):** *m/z* calcd. for $C_{16}H_{14}FNO_2Na$ $[M+Na]^+$ 294.0901, found 294.0902.

***3-(4-Chlorophenyl)-3-ethoxyindolin-2-one* 2s**

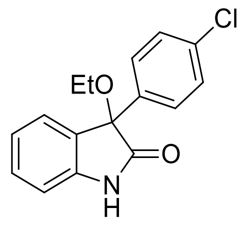

2s

General procedure **A** was followed with **1s** (97.5 mg, 0.40 mmol) and ethanol (2.5 mL). Purification was performed with flash column chromatography over silica gel (20 g SiO_2, heptane/EtOAc, 100/0 to 50/50, gradient) to afford **2s** as a light yellow solid (59.8 mg, 0.208 mmol, 52%). **^{1}H NMR (600 MHz, $CDCl_3$):** δ 8.36 (s, 1H), 7.35-7.31 (m, 2H), 7.29-7.22 (m, 2H), 7.19 (s, 1H), 7.12-7.05 (m, 1H), 6.94 (d, J = 7.8 Hz, 1H), 3.53-3.39 (m, 1H), 3.34-3.25 (m, 1H), 1.24 (t, J = 7.0 Hz, 3H). **^{13}C{^{1}H} NMR (150 MHz, $CDCl_3$)** δ 177.4 (C=O), 141.5 (C), 137.6 (C), 134.5 (C), 130.4 (CH), 128.8 (C), 128.7 (2CH), 127.9 (2CH), 126.0 (CH), 123.6 (CH), 110.7 (CH), 83.4 (C), 61.4 (CH_2), 15.5 (CH_3). **FT-IR (neat, cm^{-1}):** 3208, 1715, 1617, 1487, 1469, 1210, 1175. **HRMS (ESI$^+$):** *m/z* calcd. for $C_{16}H_{14}ClNO_2Na$ $[M+Na]^+$ 310.0606, found 310.0605.

7-Chloro-3-ethoxy-3-(4-methoxyphenyl)indolin-2-one **4a**

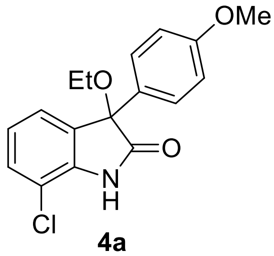

4a

General procedure **A** was followed with **3a** (109 mg, 0.40 mmol) and ethanol (2.5 mL). Purification was performed with flash column chromatography over silica gel (20 g SiO_2, heptane/EtOAc, 100/0 to 50/50, gradient) to afford **4a** as a colourless oil (32.0 mg, 0.101 mmol, 25%). **^{1}H NMR (600 MHz, $CDCl_3$):** *δ* 7.70 (s, 1H), 7.33 (t, *J* = 8.5 Hz, 3H), 7.19 (d, *J* = 7.4 Hz, 1H), 7.07 (t, *J* = 7.8 Hz, 1H), 6.85 (d, *J* = 8.8 Hz, 2H), 3.78 (s, 3H), 3.48 (dq, *J* = 14.1, 6.9 Hz, 1H), 3.32 (dq, *J* = 14.1, 6.9 Hz, 1H), 1.25 (t, *J* = 6.9 Hz, 3H). **^{13}C{^{1}H} NMR (150 MHz, $CDCl_3$):** *δ* 176.3 (C=O), 160.1 (C), 139.0 (C), 130.7 (C), 130.3 (C), 129.90 (CH), 127.8 (2CH), 124.4 (CH), 124.2 (CH), 115.7 (C), 114.1 (2CH), 84.2 (C), 61.5 (CH_2), 55.4 (CH_3), 15.5 (CH_3). **FT-IR (neat, cm^{-1}):** 3188, 2976, 2925, 1719, 1615, 1608, 1509, 1453, 1252, 1171. **HRMS (ESI$^+$):** *m/z* calcd. for $C_{17}H_{16}ClNO_3Na$ $[M+Na]^+$ 340.0711, found 340.0713.

3-Ethoxy-3-(4-methoxyphenyl)-7-(trifluoromethyl)indolin-2-one **4b**

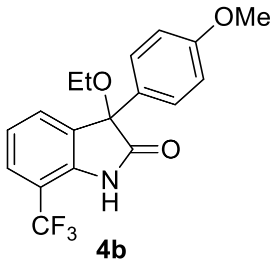

4b

General procedure **A** was followed with **3b** (123 mg, 0.40 mmol) and ethanol (2.5 mL). Purification was performed with flash column chromatography over silica gel (20 g SiO_2, heptane/EtOAc, 100/0 to 50/50, gradient) to afford **4b** as a white solid (46.1 mg, 0.311 mmol, 33%). **^{1}H NMR (600 MHz, $CDCl_3$):** *δ* 7.67 (brs, 1H), 7.54 (d, *J* = 8.0 Hz, 1H), 7.46 (d, *J* = 7.4 Hz, 1H), 7.32 (d, *J* = 8.9 Hz, 2H), 7.22 (t, *J* = 7.7 Hz, 1H), 6.86 (d, *J* = 8.9 Hz, 2H), 3.78 (s, 3H), 3.49 (dq, *J* = 14.1, 7.0 Hz, 1H), 3.31 (dq, *J* = 14.1, 7.0 Hz, 1H), 1.25 (t, *J* = 6.9 Hz, 3H). **^{13}C{^{1}H} NMR (150 MHz, $CDCl_3$):** *δ* 176.7 (C=O), 160.1 (C), 138.8 (C), 131.0 (C), 130.0 (C), 129.5 (CH), 127.8 (2CH), 126.8 (q, J^{CF} = 4.0 Hz, CH), 123.9 (q, J^{CF} = 270.0 Hz, C), 123.1 (CH), 114.1 (2CH), 112.7 (q, J^{CF} = 33.3 Hz, C), 82.2 (C), 61.5 (CH_2), 55.4 (CH_3), 15.5 (CH_3). **^{19}F NMR (565 MHz, $CDCl_3$):** *δ* -60.53. **FT-IR (neat, cm^{-1}):** 3229, 3124, 2975, 1734, 1611, 1509, 1452, 1342, 1255. **HRMS (ESI$^+$):** *m/z* calcd. for $C_{18}H_{16}F_3NO_3Na$ $[M+Na]^+$ 374.0975, found 374.0976.

***3-Ethoxy-6-methoxy-3-(4-methoxyphenyl)indolin-2-one* 4c**

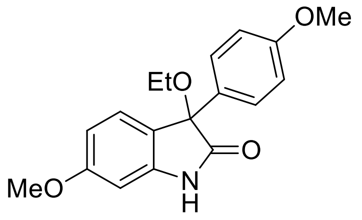

4c

General procedure **A** was followed with **3c** (108 mg, 0.40 mmol) and ethanol (2.5 mL). Purification was performed with flash column chromatography over silica gel (20 g SiO_2, heptane/EtOAc, 100/0 to 50/50, gradient) to afford **4c** as a yellow solid (73.0 mg, 0.233 mmol, 58%). **^{1}H NMR (400 MHz, $CDCl_3$):** δ 9.10 (brs, 1H), 7.34 (d, J = 8.9 Hz, 2H), 7.13 (d, J = 8.2 Hz, 1H), 6.84 (d, J = 8.9 Hz, 2H), 6.61 (dd, J = 8.2, 2.2 Hz, 1H), 6.54 (d, J = 2.2 Hz, 1H), 3.82 (s, 3H), 3.77 (s, 3H), 3.45 (dq, J = 14.1, 7.0 Hz, 1H), 3.32 ((dq, J = 14.1, 7.0 Hz, 1H), 1.24 (t, J = 7.0 Hz, 3H). **^{13}C{^{1}H} NMR (100 MHz, $CDCl_3$):** δ 179.2 (C=O), 161.5 (C), 159.8 (C), 143.0 (C), 131.3 (C), 127.9 (2CH), 126.9 (CH), 120.7 (C), 113.9 (2CH), 108.4 (CH), 97.7 (CH), 83,4 (C), 61.0 (CH_2), 55.7 (CH_3), 55.4 (CH_3), 15.6 (CH_3). **FT-IR (neat, cm^{-1}):** 3217, 2959, 2932, 2836, 1717, 1622, 1601, 1506, 1459, 1303, 1249, 1171. **HRMS (ESI$^+$):** *m/z* calcd. for $C_{18}H_{19}NO_4Na$ [M+Na]$^+$ 336.1207, found 336.1204.

***3-Ethoxy-5-methoxy-3-(4-methoxyphenyl)indolin-2-one* 4d**

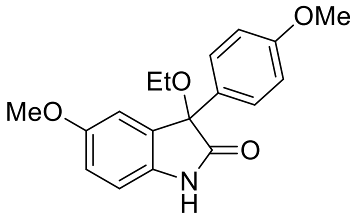

4d

General procedure **A** was followed with **3d** (108 mg, 0.40 mmol) and ethanol (2.5 mL). Purification was performed with flash column chromatography over silica gel (20 g SiO_2, heptane/EtOAc, 100/0 to 50/50, gradient) to afford **4d** as a brown oil (60.0 mg, 0.19 mmol, 48%). **^{1}H NMR (600 MHz, $CDCl_3$):** δ 8.53 (s, 1H), 7.34 (d, J = 8.8 Hz, 2H), 6.86-6.25 (m, 5H), 3.77 (s, 3H), 3.77 (s, 3H), 3.48 (dq, J = 14.0, 7.0 Hz, 1H), 3.32 (dq, J = 14.0, 7.0 Hz, 1H), 1.25 (t, J = 7.0 Hz, 3H). **^{13}C{^{1}H} NMR (150 MHz, $CDCl_3$):** δ 178.2 (C=O), 159.8 (C), 156.5 (C), 134.8 (C), 131.0 (C), 130.6 (C), 127.8 (2CH), 115.0 (CH), 114.0 (2CH), 112.4 (CH), 111.1 (CH), 84.0 (C), 61.3 (CH_2), 55.9 (CH_3), 55.4 (CH_3), 15.6 (CH_3). **FT-IR (neat, cm^{-1}):** 3238, 2975, 2932, 2836, 1713, 1607, 1509, 1485, 1438, 1297, 1249, 1275. **HRMS (ESI$^+$):** *m/z* calcd. for $C_{18}H_{19}NO_4Na$ [M+Na]$^+$ 336.1207, found 336.1204.

***5-Bromo-3-ethoxy-3-(4-methoxyphenyl)indolin-2-one* 4e**

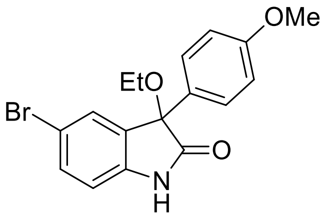

4e

General procedure **A** was followed with **3e** (127 mg, 0.40 mmol) and ethanol (2.5 mL). Purification was performed with flash column chromatography over silica gel (20 g SiO_2, heptane/EtOAc, 100/0 to 50/50, gradient) to afford **4e** as a light yellow solid (114 mg, 0.319 mmol, 79%). **^{1}H NMR (600 MHz, $CDCl_3$):** δ 8.92 (brs, 1H), 7.42 (dd, J = 8.3, 1.9 Hz, 1H), 7.35 (d, J = 1.8 Hz, 1H), 7.31 (d, J = 8.9 Hz, 2H), 6.86 (d, J = 8.9 Hz, 2H), 6.83 (d, J = 8.3 Hz, 1H), 3.78 (s, 3H), 3.47 (dq, J = 14.0, 7.0 Hz, 1H), 3.32 (dq, J = 14.0, 7.0 Hz, 1H), 1.26 (t, J = 6.9 Hz, 3H). **^{13}C{^{1}H} NMR (150 MHz, $CDCl_3$):** δ 178.0 (C=O), 160.0 (C), 140.5 (C), 133.0 (CH), 131.6 (C), 130.3 (C), 129.0 (CH), 127.6 (2CH), 116.1 (C), 114.1 (2CH), 112.3 (CH), 83.6 (C), 61.4 (CH_2), 55.4 (CH_3), 15.5 (CH_3). **FT-IR (neat, cm^{-1}):** 3215, 2974, 2929, 2836, 1713, 1609, 1508, 1468, 1438, 1248, 1171. **HRMS (ESI$^+$):** *m/z* calcd. for $C_{17}H_{16}BrNO_3Na$ $[M+Na]^+$ 384.0206, found 384.0203.

***3-Ethoxy-3-(4-methoxyphenyl)-5-nitroindolin-2-one* 4f**

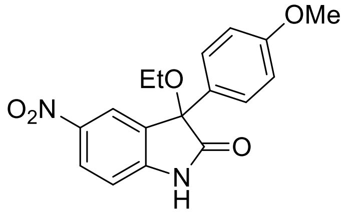

4f

General procedure **A** was followed with **3f** (114 mg, 0.40 mmol) and ethanol (2.5 mL). Purification was performed with flash column chromatography over silica gel (20 g SiO_2, heptane/EtOAc, 100/0 to 50/50, gradient) to afford **4f** as a white solid (49.8 mg, 0.152 mmol, 38%). **^{1}H NMR (500 MHz, $CDCl_3$):** δ 8.55 (s, 1H), 8.29 (dd, J = 8.6, 2.3 Hz, 1H), 8.17 (d, J = 2.3 Hz, 1H), 7.32 (d, J = 8.9 Hz, 2H), 7.05 (d, J = 8.6 Hz, 1H), 6.88 (d, J = 8.9 Hz, 2H), 3.79 (s, 3H), 3.50 (dq, J = 13.9, 6.9 Hz, 1H), 3.36 (dq, J = 13.9, 7.0 Hz, 1H), 1.28 (d, J = 6.9 Hz, 3H). **^{13}C{^{1}H} NMR (126 MHz, $CDCl_3$):** δ 177.7 (C=O), 160.3 (C), 146.8 (C), 144.2 (C), 130.6 (C), 129.2 (C), 127.6 (2CH), 127.0 (CH), 122.0 (CH), 114.4 (2CH), 110.4 (CH), 83.0 (C), 61.7 (CH_2), 55.5 (CH_3), 15.5 (CH_3). **FT-IR (neat, cm^{-1}):** 3231, 2929, 1733, 1622, 1606, 1525, 1510, 1338, 1253, 1177. **HRMS (ESI$^+$):** *m/z* calcd. for $C_{17}H_{16}N_2O_5Na$ $[M+Na]^+$ 351.0952, found 351.0950.

***3-Ethoxy-1,3-dimethylindolin-2-one* 6a**

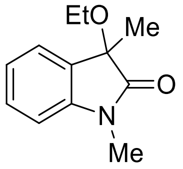

6a

General procedure **A** was followed with **5a** (64.5 mg, 0.40 mmol) and ethanol (2.5 mL). Purification was performed with flash column chromatography over silica gel (20 g SiO_2, heptane/EtOAc, 100/0 to 50/50, gradient) to afford **6a** as a light yellow oil (50.7 mg, 0.247 mmol, 62%). **^{1}H NMR (400 MHz, $CDCl_3$):** δ 7.32 (dd, J = 10.2, 4.2 Hz, 2H), 7.10 (t, J = 7.6 Hz, 1H), 6.83 (d, J = 8.2 Hz, 1H), 3.20 (s, 3H), 3.15 (dd, J = 14.7, 7.6 Hz, 1H), 3.03 (dd, J = 14.7, 7.6 Hz, 1H), 1.54 (s, 3H), 1.12 (t, J = 7.0 Hz, 3H). **^{13}C{^{1}H} NMR (100 MHz, $CDCl_3$):** δ 177.0 (C=O), 143.3 (C), 129.6 (CH), 129.5 (C), 123.7 (CH), 123.2 (CH), 108.4 (CH), 79.1 (C), 61.0 (CH_2), 26.2 (CH_3), 24.3 (CH_3), 15.5 (CH_3). **HRMS (ESI$^+$):** *m/z* calcd. for $C_{12}H_{15}NO_2Na$ $[M+Na]^+$ 228.0995, found 228.0993. Spectral and physical data are in accordance with literature.[17]

***3-Ethoxy-3-methyl-1-phenylindolin-2-one* 6b**

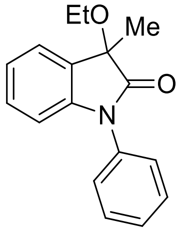

6b

General procedure **A** was followed with **5b** (89.3 mg, 0.40 mmol) and ethanol (2.5 mL). Purification was performed with flash column chromatography over silica gel (20 g SiO_2, heptane/EtOAc, 100/0 to 50/50, gradient) to afford **6b** as a white solid (79.6 mg, 0.298 mmol, 74%). **^{1}H NMR (500 MHz, $CDCl_3$):** δ 7.52 (dd, J = 10.6, 5.1 Hz, 2H), 7.42 (dd, J = 10.7, 4.6 Hz, 4H), 7.28-7.24 (m, 1H), 7.15 (td, J = 7.5, 0.7 Hz, 1H), 6.84 (d, J = 7.9 Hz, 1H), 3.35 (dq, J = 13.9, 7.0 Hz, 1H), 3.21 (dq, J = 14.1, 7.0 Hz, 1H), 1.68 (s, 3H), 1.19 (t, J = 7.0 Hz, 3H). **^{13}C{^{1}H} NMR (126 MHz, $CDCl_3$):** δ 176.4 (C=O), 143.4 (C), 134.3 (C), 129.8 (2CH), 129.6 (CH), 129.4 (C), 128.3 (CH), 126.6 (2CH), 124.1 (CH), 123.7 (CH), 109.8 (CH), 79.2 (C), 61.2 (CH_2), 24.6 (CH_3), 15.6 (CH_3). **FT-IR (neat, cm^{-1}):** 3055, 2976, 2923, 2877, 1720, 1607, 1595, 1496, 1455, 1370, 1202, 1182. **HRMS (ESI$^+$):** *m/z* calcd. for $C_{17}H_{17}NO_2Na$ $[M+Na]^+$ 290.1152, found 290.1151.

***Tert-butyl 3-ethoxy-2-oxo-3-phenylindoline-1-carboxylate* 6c**

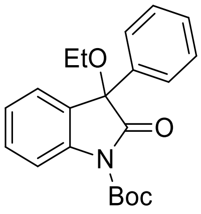

6c

General procedure **A** was followed with **5c** (124 mg, 0.40 mmol) and ethanol (2.5 mL). Purification was performed with flash column chromatography over silica gel (20 g SiO_2, heptane/EtOAc, 100/0 to 50/50, gradient) to afford **6c** as a white solid (55.6 mg, 0.157 mmol, 40%). **^{1}H NMR (700 MHz, $CDCl_3$):** δ 7.98 (d, J = 8.2 Hz, 1H), 7.44 (td, J = 7.5, 1.5 Hz, 1H), 7.36 (dd, J = 8.0, 1.5 Hz, 2H), 7.33-7.28 (m, 4H), 7.25 (td, J = 7.5, 0.6 Hz, 1H), 3.44 (dq, J = 14.0, 7.0 Hz, 1H), 3.30 (dq, J = 14.0, 7.0 Hz, 1H), 1.62 (s, 9H), 1.25 (t, J = 7.0 Hz, 3H). **^{13}C{^{1}H} NMR (176 MHz, $CDCl_3$):** δ 173.8 (C=O), 149.3 (C=O), 140.8 (C), 138.9 (C), 130.4 (CH), 128.7 (CH), 128.6 (2CH), 127.6 (C), 126.7 (2CH), 125.7 (CH), 125.2 (CH), 115.5 (CH), 84.8 (C), 83.4 (C), 61.4 (CH_2), 28.2 ($3CH_3$), 15.5 (CH_3). **FT-IR (neat, cm^{-1}):** 2923, 2878, 2853, 1770, 1726, 1464, 1339, 1285, 1246, 1160**.** **HRMS (ESI$^+$):** *m/z* calcd. for $C_{21}H_{23}NO_4Na$ $[M+Na]^+$ 376.1520, found 376.1520.

***3-Methoxy-3-(4-methoxyphenyl)indolin-2-one* 7a**

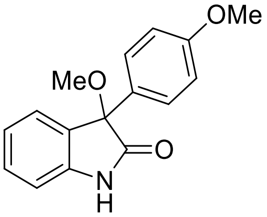

7a

General procedure **A** was followed with **1m** (95.7 mg, 0.40 mmol) and methanol (2.5 mL). Purification was performed with flash column chromatography over silica gel (20 g SiO_2, heptane/EtOAc, 100/0 to 50/50, gradient) to afford **7a** as a light yellow solid (68.1 mg, 0.253 mmol, 63%). **^{1}H NMR (400 MHz, $CDCl_3$):** δ 9.29 (brs, 1H), 7.36-7.28 (m, 3H), 7.24 (d, J = 7.3 Hz, 1H), 7.11 (dd, J = 7.8, 7.3 Hz, 1H), 6.95 (d, J = 7.8 Hz, 1H), 6.85 (d, J = 8.9 Hz, 2H), 3.77 (s, 3H), 3.27 (s, 3H). **^{13}C{^{1}H} NMR (100 MHz, $CDCl_3$):** δ 178.5 (C=O), 159.8 (C), 141.9 (C), 130.6 (C), 130.2 (CH), 128.4 (C), 127.8 (2CH), 126.1 (CH), 123.3 (CH), 114.0 (2CH), 111.0 (CH), 84.2 (C), 55.4 (CH_3), 53.3 (CH_3). **FT-IR (neat, cm^{-1}):** 3177, 2927, 2818, 1720, 1690, 1618, 1605, 1508, 1471, 1255, 1219. **HRMS (ESI$^+$):** *m/z* calcd. For $C_{16}H_{15}NO_3Na$ $[M+Na]^+$ 292.0945, found 292.0935.

***3-(Allyloxy)-3-(4-methoxyphenyl)indolin-2-one* 7b**

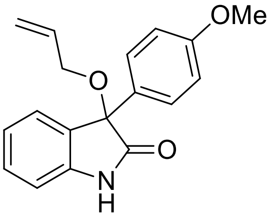

7b

General procedure **A** was followed with **1m** (95.7 mg, 0.40 mmol) and allyl alcohol (2.5 mL). Purification was performed with flash column chromatography over silica gel (20 g SiO_2, heptane/EtOAc, 100/0 to 50/50, gradient) to afford **7b** as a light yellow solid (40.0 mg, 0.135 mmol, 34%). **^{1}H NMR (400 MHz, $CDCl_3$):** δ 8.60 (brs, 1H), 7.35 (d, J = 8.9 Hz, 2H), 7.34.-7.29 (m, 1H), 7.27 (d, J = 6.4 Hz, 1H), 7.11 (dd, J = 7.8, 7.3 Hz, 1H), 6.94 (d, J = 7.8 Hz, 1H), 6.85 (d, J = 8.9 Hz, 2H), 6.00-5.89 (m, 1H), 5.32-5.25 (m, 1H), 5.16-5.12 (m, 1H), 3.97 (dd, J = 11.8, 5.5 Hz, 1H), 3.83 (dd, J = 11.8, 5.5 Hz, 1H), 3.78 (s, 3H). **^{13}C{^{1}H} NMR (100 MHz, $CDCl_3$):** δ 178.1 (C=O), 159.9 (C), 141.6 (C), 134.3 (CH), 130.7 (C), 130.2 (CH), 128.9 (C), 127.9 (2CH), 126.2 (CH), 123.4 (CH), 117.2 (CH_2), 114.2 (2CH), 110.8 (CH), 83.5 (C), 66.8 (CH_2), 55.4 (CH_3). **FT-IR (neat, cm^{-1}):** 3177, 2916, 1720, 1688, 1617, 1605, 1509, 1470, 1254, 1215, 1171, 1109. **HRMS (ESI$^+$):** *m/z* calcd. for $C_{18}H_{17}NO_3Na$ $[M+Na]^+$ 318.1101, found 318.1112.

***3-(Benzyloxy)-3-(4-methoxyphenyl)indolin-2-one* 7c**

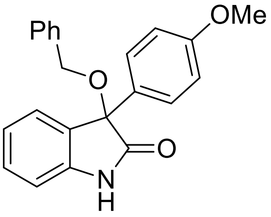

7c

General procedure **A** was followed with **1m** (95.7 mg, 0.40 mmol) and benzyl alcohol (2.5 mL). Purification was performed with flash column chromatography over silica gel (20 g SiO_2, heptane/EtOAc, 100/0 to 50/50, gradient) to afford **7c** as a light yellow solid (90.1 mg, 0.261 mmol, 65%). **^{1}H NMR (400 MHz, $CDCl_3$):** δ 9.22 (brs, 1H), 7.43-7.36 (m, 4H), 7.35-7.28 (m, 4H), 7.27 (d, J = 7.5 Hz, 1H), 7.12 (dd, J = 7.7, 7.5 Hz, 1H), 6.98 (d, J = 7.7 Hz, 1H), 6.86 (d, J = 8.8 Hz, 2H), 4.51 (d, J = 10.6 Hz, 1H), 4.33 (d, J = 10.6 Hz, 1H), 3.77 (s, 3H). **^{13}C{^{1}H} NMR (100 MHz, $CDCl_3$):** δ 178.3 (C=O), 159.9 (C), 141.8 (C), 137.9 (C), 130.8 (C), 130.3 (CH), 128.8 (C), 128.4 (2CH), 128.0 (2CH), 127.9 (2CH), 127.8 (CH), 126.1 (CH), 123.4 (CH), 114.0 (2CH), 111.0 (CH), 83.4 (C), 67.7 (CH_2), 55.4 (CH_3). **FT-IR (neat, cm^{-1}):** 3195, 3087, 3060, 2928, 2836, 1709, 1615, 1605, 1508, 1469, 1244, 1212, 1170, 1106, 1087, 1026. **HRMS (ESI$^+$):** *m/z* calcd. for $C_{22}H_{19}NO_3Na$ $[M+Na]^+$ 368.1258, found 368.1265.

***3-(Hexyloxy)-3-(4-methoxyphenyl)indolin-2-one* 7d**

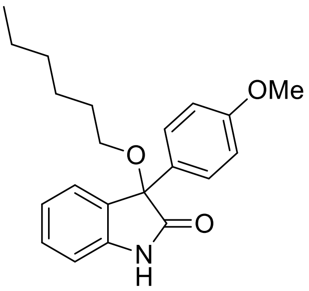

7d

General procedure **A** was followed with **1m** (95.7 mg, 0.40 mmol) and 1-hexanol (1.0 mL, 20.0 equiv.) in MeCN (5.0 mL). Purification was performed with flash column chromatography over silica gel (20 g SiO_2, heptane/EtOAc, 100/0 to 50/50, gradient) to afford **7d** as a light brown solid (71.0 mg, 0.209 mmol, 52%). **^{1}H NMR (600 MHz, $CDCl_3$):** δ 8.74 (brs, 1H), 7.33 (d, J = 8.9 Hz, 2H), 7.30 (td, J = 7.7, 1.2 Hz, 1H), 7.23 (d, J = 7.2 Hz, 1H), 7.10 (td, J = 7.5, 0.8 Hz, 1H), 6.94 (d, J = 7.8 Hz, 1H), 6.84 (d, J = 8.9 Hz, 4H), 3.77 (s, 3H), 3.21 (dt, J = 8.2, 6.8 Hz, 2H), 3.21 (dt, J = 8.2, 6.8 Hz, 1H), 1.62 (dt, J = 13.7, 6.8 Hz, 2H), 1.39-1.33 (m, 2H), 1.30-1.22 (m, 4H), 0.86 (t, J = 7.1 Hz, 3H). **^{13}C{^{1}H} NMR (150 MHz, $CDCl_3$):** δ 178.3 (C=O), 159.8 (C), 141.6 (C), 131.2 (C), 130.0 (CH), 129.3 (C), 127.8 (2CH), 126.1 (CH), 123.3 (CH), 113.9 (2CH), 110.7 (CH), 83.5 (C), 66.6 (CH_2), 55.4 (CH_3), 31.7 (CH_2), 30.0 (CH_2), 25.9 (CH_2), 22.7 (CH_2), 14.2 (CH_3). **FT-IR (neat, cm^{-1}):** 3171, 2930, 2867, 1721, 1689, 1619, 1606, 1509, 1467, 1217. **HRMS (ESI$^+$):** *m/z* calcd. for $C_{21}H_{25}NO_3Na$ [M+Na]$^+$ 362.1727, found 362.1727.

***3-(But-2-yn-1-yloxy)-3-(4-methoxyphenyl)indolin-2-one* 7e**

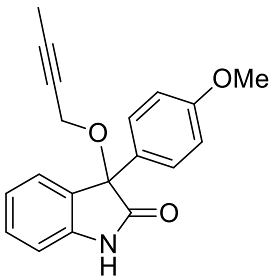

7e

General procedure **A** was followed with **1m** (95.7 mg, 0.40 mmol) and 2-butyn-1-ol (0.60 mL, 20.0 equiv.) in MeCN (5.0 mL). Purification was performed with flash column chromatography over silica gel (20 g SiO_2, heptane/EtOAc, 100/0 to 50/50, gradient) to afford **7e** as a light yellow solid (65.0 mg, 0.211 mmol, 53%). **^{1}H NMR (700 MHz, $CDCl_3$):** δ 9.08 (brs, 1H), 7.34 (d, J = 8.9 Hz, 2H), 7.31 (td, J = 7.7, 1.1 Hz, 1H), 7.28 (d, J = 7.3 Hz, 1H), 7.09 (dd, J = 11.0, 4.0 Hz, 1H), 6.96 (d, J = 7.7 Hz, 1H), 6.83 (d, J = 8.9 Hz, 2H), 4.10 (dq, J = 14.2, 2.3 Hz, 1H), 4.01 (dq, J = 14.2, 2.3 Hz, 1H), 3.76 (s, 3H), 1.76 (s, 3H). **^{13}C{^{1}H} NMR (176 MHz, $CDCl_3$):** δ 177.9 (C=O), 159.9 (C), 141.7 (C), 130.4 (CH), 130.3 (C), 128.1 (C), 128.0 (2CH), 126.4 (CH), 123.3 (CH), 114.0 (2CH), 110.9 (CH), 83.4 (C), 83.2 (C), 74.9 (C), 55.4 (CH_3), 54.6 (CH_2), 3.8 (CH_3). **FT-IR (neat, cm^{-1}):** 3208, 2961, 2921, 1720, 1681, 1619, 1508, 1466, 1215. **HRMS (ESI$^+$):** *m/z* calcd. for $C_{19}H_{17}NO_3Na$ [M+Na]$^+$ 330.1101, found 330.1103.

***3-(4-Methoxyphenyl)-3-(2-methylbutoxy)indolin-2-one* 7f**

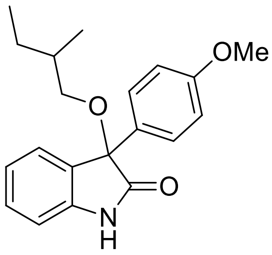

7f, *dr*: 1/1

General procedure **A** was followed with **1m** (95.7 mg, 0.40 mmol) and DL-2-methyl-1-butanol (2.5 mL). Purification was performed with flash column chromatography over silica gel (20 g SiO_2, heptane/EtOAc, 100/0 to 50/50, gradient) to afford **7f** as a light yellow solid (87.0 mg, 0.267 mmol, 67%, *dr*: 1/1). **^{1}H NMR (400 MHz, $CDCl_3$, mixture of diastereoisomers):** δ 9.00 (brs, 1H), 7.34 (d, J = 8.7 Hz, 2H), 7.30 (t, J = 7.8 Hz, 1H), 7.21 (d, J = 7.3 Hz, 1H), 7.09 (dd, J = 7.8, 7.3 Hz, 1H), 6.94 (d, J = 7.8 Hz, 1H), 6.84 (d, J = 8.9 Hz, 2H), 3.77 (s, 3H), 3.25 (ddd, J = 21.0, 8.0, 6.0 Hz, 1H), 3.02 (ddd, J = 21.0, 8.0, 6.5 Hz, 1H), 1.75-1.66 (m, 1H), 1.56-1.43 (m, 1H), 1.21-1.09 (m, 1H), 0.92 (dd, 6.7, 2.8 Hz, 3H), 0.88-0.82 (m, 3H). **^{13}C{^{1}H} NMR (100 MHz, $CDCl_3$, mixture of diastereoisomers):** δ 178.6 (C=O), 159.7 (C), 141.7 (C), 131.4 (C), 130.0 (CH), 129.3 (C), 129.3 (2CH), 127.7 (CH), 123.3 (CH), 113.9 (2CH), 110.7 (CH), 83.4 (C), 70.3 (CH_2, d1), 70.2 (CH_2, d2), 55.4 (CH_3), 35.4 (CH, d1), 35.3 (CH, d2), 26.2 (CH_2, d1), 26.2 (CH_2, d2), 16.8 (CH_3, d1), 16.6 (CH_3, d2), 11.4 (CH_3, d1), 11.3 (CH_3, d2). **FT-IR (neat, cm^{-1}):** 3208, 2960, 2908, 2873, 1722, 1687, 1618, 1607, 1508, 1467, 1254, 1215, 1171. **HRMS (ESI$^+$):** *m/z* calcd. for $C_{20}H_{23}NO_3Na$ [M+Na]$^+$ 348.1571, found 348.1580.

***3-(4-Methoxyphenyl)-3-(2-((trimethylsilyl)oxy)ethoxy)indolin-2-one* 7g**

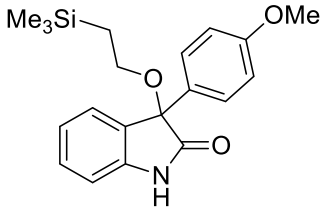

7g

General procedure **A** was followed with **1m** (95.7 mg, 0.40 mmol) and 2-(trimethylsilyl)ethanol (1.19 mL, 20.0 equiv.) in MeCN (5.0 mL). Purification was performed with flash column chromatography over silica gel (20 g SiO_2, heptane/EtOAc, 100/0 to 50/50, gradient) to afford **7g** as a light yellow solid (115.0 mg, 0.323 mmol, 81%). **^{1}H NMR (400 MHz, $CDCl_3$):** δ 8.38 (brs, 1H), 7.35-7.28 (m, 3H), 7.24 (d, J = 7.2 Hz, 1H), 7.12 (dd, J = 7.8, 7.2 Hz, 1H), 6.93 (d, J = 7.8 Hz, 1H), 6.84 (d, J = 8.9 Hz, 2H), 3.77 (s, 3H), 3.51 (td, J = 8.8, 6.4 Hz, 1H), 3.34 (td, J = 8.8, 6.9 Hz, 1H), 1.04-0.93 (m, 2H), -0.03 (s, 9H). **^{13}C{^{1}H} NMR (100 MHz, $CDCl_3$):** δ 178.2 (C=O), 159.8 (C), 141.5 (C), 131.2 (C), 130.0 (CH), 129.3 (C), 127.8 (2CH), 126.1 (CH), 123.3 (CH), 113.9 (2CH), 110.6 (CH), 83.5 (C), 62.9 (CH_2), 55.4 (CH_3), 18.7 (CH_2), -1.1 ($3CH_3$). **FT-IR (neat, cm^{-1}):** 3084, 2955, 2894, 1714, 1614, 1604, 1508, 1469, 1275, 1252, 1174. **HRMS (ESI$^+$):** *m/z* calcd. for $C_{20}H_{25}NO_3SiNa$ [M+Na]$^+$ 378.1496, found 378.1508.

***3-(3-Chloropropoxy)-3-(4-methoxyphenyl)indolin-2-one* 7h**

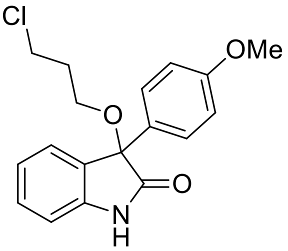

7h

General procedure **A** was followed with **1m** (95.7 mg, 0.40 mmol) and 3-chloro-1-propanol (2.5 mL). Purification was performed with flash column chromatography over silica gel (20 g SiO_2, heptane/EtOAc, 100/0 to 50/50, gradient) to afford **7h** as a light yellow solid (78.1 mg, 0.235 mmol, 59%). **^{1}H NMR (400 MHz, $CDCl_3$):** δ 8.76 (brs, 1H), 7.34-7.30 (m, 3H), 7.25 (d, *J* = 7.7 Hz, 1H), 7.11 (dd, *J* = 7.8, 7.7 Hz, 1H), 6.95 (d, *J* = 7.8 Hz, 1H), 6.85 (d, *J* = 8.9 Hz, 2H), 3.77 (s, 3H), 3.71 (t, *J* = 6.5 Hz, 2H), 3.54-3.49 (m, 1H), 3.44-3.38 (m, 1H), 2.14-2.02 (m, 2H). **^{13}C{^{1}H} NMR (100 MHz, $CDCl_3$):** δ 178.3 (C=O), 159.9 (C), 141.6 (C), 130.7 (C), 130.2 (CH), 128.7 (C), 127.8 (2CH), 126.1 (CH), 123.4 (CH), 114.0 (2CH), 110.9 (CH), 83.6 (C), 62.0 (CH_2), 55.4 (CH_3), 41.9 (CH_2), 33.0 (CH_2). **FT-IR (neat, cm^{-1}):** 3171, 2957, 1727, 1684, 1617, 1508, 1468, 1250, 1214, 1170. **HRMS (ESI$^+$):** *m/z* calcd. for $C_{18}H_{18}NClO_3Na$ $[M+Na]^+$ 354.0867, found 354.0867.

***3-(4-Methoxyphenyl)-3-phenethoxyindolin-2-one* 7i**

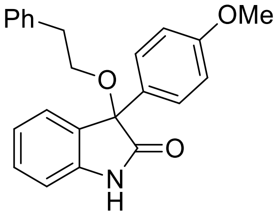

7i

General procedure **A** was followed with **1m** (95.7 mg, 0.40 mmol) and 2-phenylethyl alcohol (0.50 mL, 10.0 equiv.) in MeCN (5.0 mL). Purification was performed with flash column chromatography over silica gel (20 g SiO_2, heptane/EtOAc, 100/0 to 50/50, gradient) to afford **7i** as a brown solid (51.0 mg, 0.142 mmol, 36%). **^{1}H NMR (500 MHz, $CDCl_3$):** δ 8.69 (brs, 1H), 7.32-7.26 (m, 3H), 7.24 (d, *J* = 7.5 Hz, 2H), 7.21-7.15 (m, 3H), 7.05 (qd, *J* = 7.4, 0.9 Hz, 2H), 6.91 (d, *J* = 7.8 Hz, 1H), 6.83 (d, *J* = 8.9 Hz, 2H), 3.77 (s, 3H), 3.66 (td, *J* = 7.9, 6.2 Hz, 1H), 3.43 (dd, *J* = 15.8, 7.5 Hz, 1H), 3.00-2.91 (m, 2H). **^{13}C{^{1}H} NMR (126 MHz, $CDCl_3$):** δ 178.1 (C=O), 159.8 (C), 141.5 (C), 138.7 (C), 130.9 (C), 130.1 (CH), 129.2 (2CH), 129.0 (C), 128.4 (2CH), 127.8 (2CH), 126.3 (CH), 126.1 (CH), 123.4 (CH), 113.9 (2CH), 110.6 (CH), 83.6 (C), 66.5 (CH_2), 55.4 (CH_3), 36.6 (CH_2). **FT-IR (neat, cm^{-1}):** 3291, 2928, 1724, 1687, 1687, 1619, 1506, 1452. **HRMS (ESI$^+$):** *m/z* calcd. for $C_{23}H_{21}NO_3Na$ $[M+Na]^+$ 383.1414, found 383.1408.

7. Procedures and Characterization data of 3,3-substituted oxindoles 8 and 9 (Scheme 3)

***3-(4-Methoxyphenyl)-3-(2-oxo-2-phenylethyl)indolin-2-one* 8**

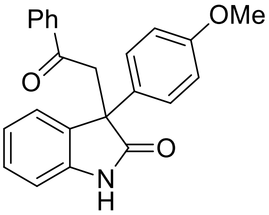

8

With no precautions to exclude air or moisture, the ElectraSyn vial (10 mL) was charged with **1m** (95.7 mg, 0.40 mmol), Et_4NOTs (121 mg, 0.40 mmol, 1.0 equiv.), 1-phenyl-1-trimethylsilyloxyethylene (820 µL, 10.00 equiv.) and MeCN (5.0 mL). The ElectraSyn vial cap equipped with anode (graphite) and cathode (graphite) were inserted into the mixture. The reaction mixture was electrolyzed at a constant current of 10 mA for 3 h. The ElectraSyn vial cap was removed, and electrodes were rinsed with DCM (2.0 mL), which was combined with the crude mixture. Then, the crude mixture was concentrated under reduced pressure and purified by FC over silica gel (heptane/ethyl acetate, 100/0 to 50/50, gradient) to furnish the desired product **8** as a light yellow solid (88.2 mg, 0.247 mmol, 62%).

^{1}H NMR (400 MHz, $CDCl_3$): δ 8.21 (brs, 1H), 7.91-7.84 (m, 2H), 7.53 (t, J = 7.4 Hz, 1H), 7.43-7.33 (m, 4H), 7.24 (dd, J = 13.0, 5.4 Hz, 2H), 7.01 (t, J = 7.5 Hz, 1H), 6.96 (d, J = 7.6 Hz, 1H), 6.85 (d, J = 8.9 Hz, 2H), 4.15 (d, J = 18.0 Hz, 1H), 4.08 (d, J = 18.0 Hz, 1H), 3.77 (s, 3H). **^{13}C{^{1}H} NMR (100 MHz, $CDCl_3$):** δ 196.0 (C=O), 180.9 (C=O), 159.2 (C), 141.9 (C), 136.5 (C), 133.5 (CH), 132.5 (C), 131.4 (C), 128.7 (2CH), 128.4 (CH), 128.2 (2CH), 128.0 (2CH), 124.5 (CH), 122.3 (CH), 114.2 (2CH), 111.3 (CH), 55.4 (CH_3), 53.0 (C), 47.0 (CH_2). **FT-IR (neat, cm^{-1}):** 3132, 3058, 3007, 2963, 2838, 1708, 1671, 1616, 1506, 1472, 1218, 1183. **HRMS (ESI$^+$):** m/z calcd. for $C_{23}H_{19}NO_3Na$ $[M+Na]^+$ 380.1258, found 380.1253.

***3-Azido-3-(4-methoxyphenyl)indolin-2-one* 9**

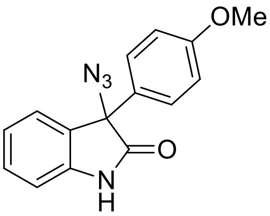

9

With no precautions to exclude air or moisture, the ElectraSyn vial (10 mL) was charged with **1m** (95.7 mg, 0.40 mmol), Et_4NOTs (121 mg, 0.40 mmol, 1.0 equiv.), azidotrimethylsilane (0.53 mL, 10.0 equiv.) and MeCN (5.0 mL). The ElectraSyn vial cap equipped with anode (graphite) and cathode (graphite) were inserted into the mixture. The reaction mixture was electrolyzed at a constant current of 10 mA for 3 h. The ElectraSyn vial cap was removed, and

electrodes were rinsed with DCM (2.0 mL), which was combined with the crude mixture. Then, the crude mixture was concentrated under reduced pressure and purified by FC over silica gel (heptane/ethyl acetate, 100/0 to 50/50, gradient) to furnish the desired product **9** as a light yellow oil (25.1 mg, 0.089 mmol, 22%).

^{1}H NMR (400 MHz, $CDCl_3$): δ 8.66 (brs, 1H), 7.37 (d, J = 8.8 Hz, 2H), 7.35-7.31 (m, 1H), 7.29 (d, J = 7.4 Hz, 1H), 7.13 (dd, J = 7.8, 7.4 Hz, 1H), 6.98 (d, J = 7.8 Hz, 1H), 6.90 (d, J = 8.8 Hz, 2H), 3.79 (s, 3H). **^{13}C{^{1}H} NMR (100 MHz, $CDCl_3$):** δ 176.1 (C=O), 160.2 (C), 140.7 (C), 130.5 (CH), 129.0 (C), 128.2 (2CH), 128.1 (C), 125.6 (CH), 123.7 (CH), 114.5 (2CH), 111.0 (CH), 70.0 (C), 55.5 (CH_3). **FT-IR (neat, cm^{-1}):** 3234, 2956, 2932, 2837, 2096, 1716, 1508, 1470, 1249, 1177. **HRMS (ESI^{+}):** *m/z* calcd. for $C_{15}H_{12}N_4O_2Na$ $[M+Na]^+$ 303.0853, found 303.0867.

8. Additional Experiments with Different Type of Alkenes

All these reactions were carried out on 0.40 mmol scale following the general procedure A and are **preliminary unoptimized results.** The crude reaction mixture was analyzed by ^{1}H NMR and purified by FC (10 g SiO_2, heptane/ethyl acetate: 100/0 to 70/30, 35 CV).

Ketene Silyl Acetal

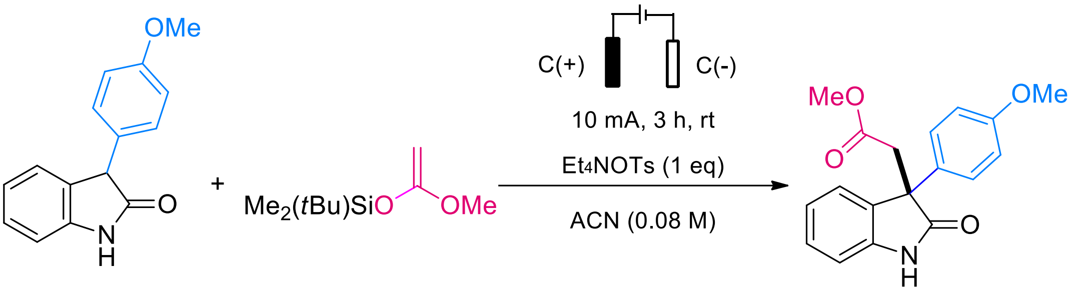

1m (10 eq) **17%**

Allylsilane

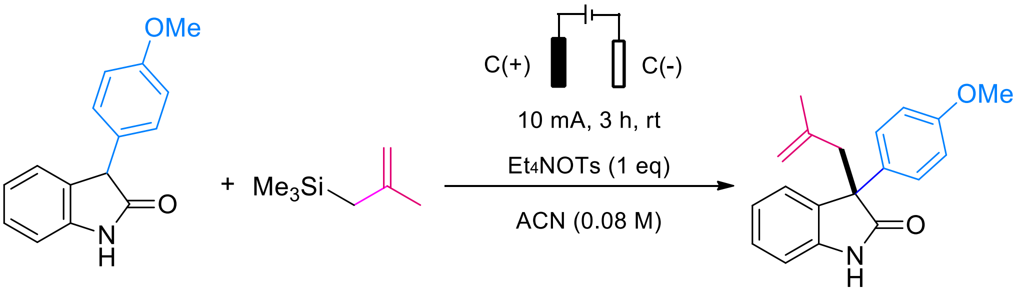

1m (10 eq) **37% (52% brsm)**

Alkenyl Silane

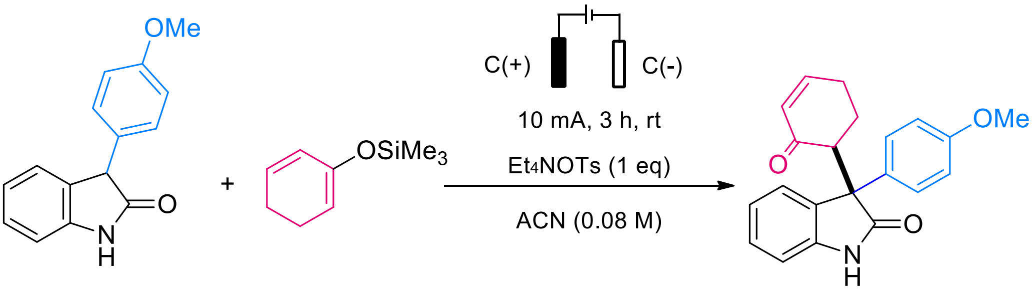

1m (5 eq)

traces of product
mainly SM recovered

9. BHT trapping experiment (Scheme 4)

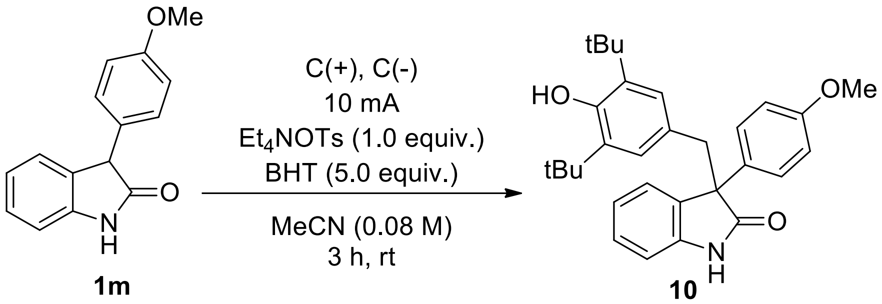

With no precautions to exclude air or moisture, the ElectraSyn vial (10 mL) was charged with 3-(4-methoxyphenyl)indolin-2-one **1m** (95.7 mg, 0.40 mmol, 1.0 equiv.), Et_4NOTs (121 mg, 0.40 mmol, 1.0 equiv.), 2,6-di-tert-butyl-4-methylphenol (441 mg, 2.0 mmol, 5.0 equiv.) and MeCN (5.0 mL). The ElectraSyn vial cap equipped with anode (graphite) and cathode (graphite) were inserted into the mixture. The reaction mixture was electrolyzed at a constant current of 10 mA for 3 h. The ElectraSyn vial cap was removed, and electrodes were rinsed with DCM (2.0 mL), which was combined with the crude mixture. Then, the crude mixture was concentrated under reduced pressure and purified by FC over silica gel (heptane/ethyl acetate, 100/0 to 50/50, gradient) to furnish the desired product **10** as a white solid (98.9 mg, 0.216 mmol, 54%).

^{1}H NMR (400 MHz, $CDCl_3$): δ 8.54 (s, 1H), 7.33 (d, *J* = 8.8 Hz, 2H), 7.07 (td, *J* = 7.7, 1.3 Hz, 1H), 7.03-6.92 (m, 2H), 6.80 (d, *J* = 8.8 Hz, 2H), 6.65 (d, *J* = 7.7 Hz, 1H), 6.56 (s, 2H), 4.86 (s, 1H), 3.72 (s, 3H), 3.45 (d, *J* = 12.9 Hz, 1H), 3.30 (d, *J* = 12.9 Hz, 1H), 1.13 (s, 18H). **^{13}C{^{1}H} NMR (100 MHz, $CDCl_3$):** δ 180.5 (C=O), 159.0 (C), 152.5 (C), 141.1 (C), 134.9 (2C), 132.5 (C), 131.8 (C), 128.7 (2CH), 128.0 (CH), 127.1 (2CH), 126.2 (CH), 126.1 (C), 122.1 (CH), 114.0 (2CH), 109.8 (CH), 58.4 (C), 55.4 (CH_3), 44.2 (CH_2), 34.2 (2C), 30.3 (6CH_3). **HRMS (ESI$^+$):** *m/z* calcd. for $C_{30}H_{35}NO_3Na$ $[M+Na]^+$ 480.2510, found 480.2513.

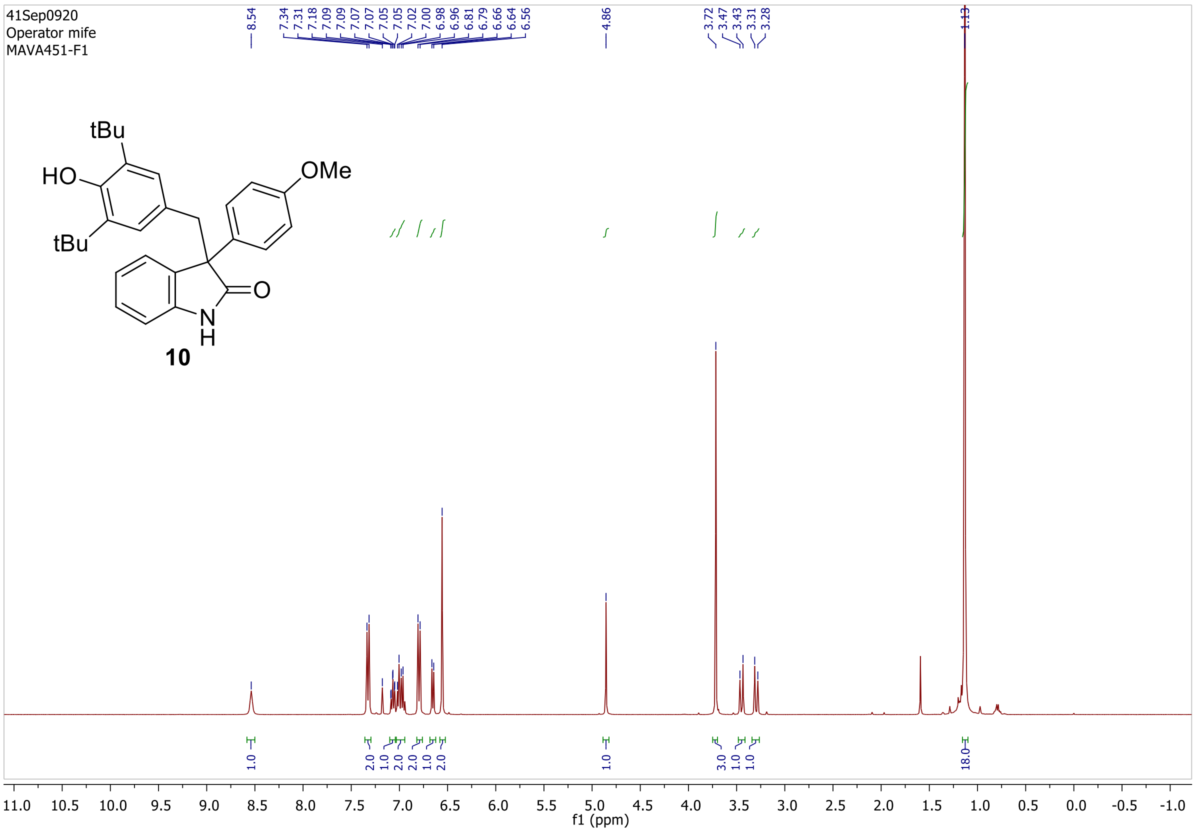

^{1}H NMR in $CDCl_3$ at 400 MHz

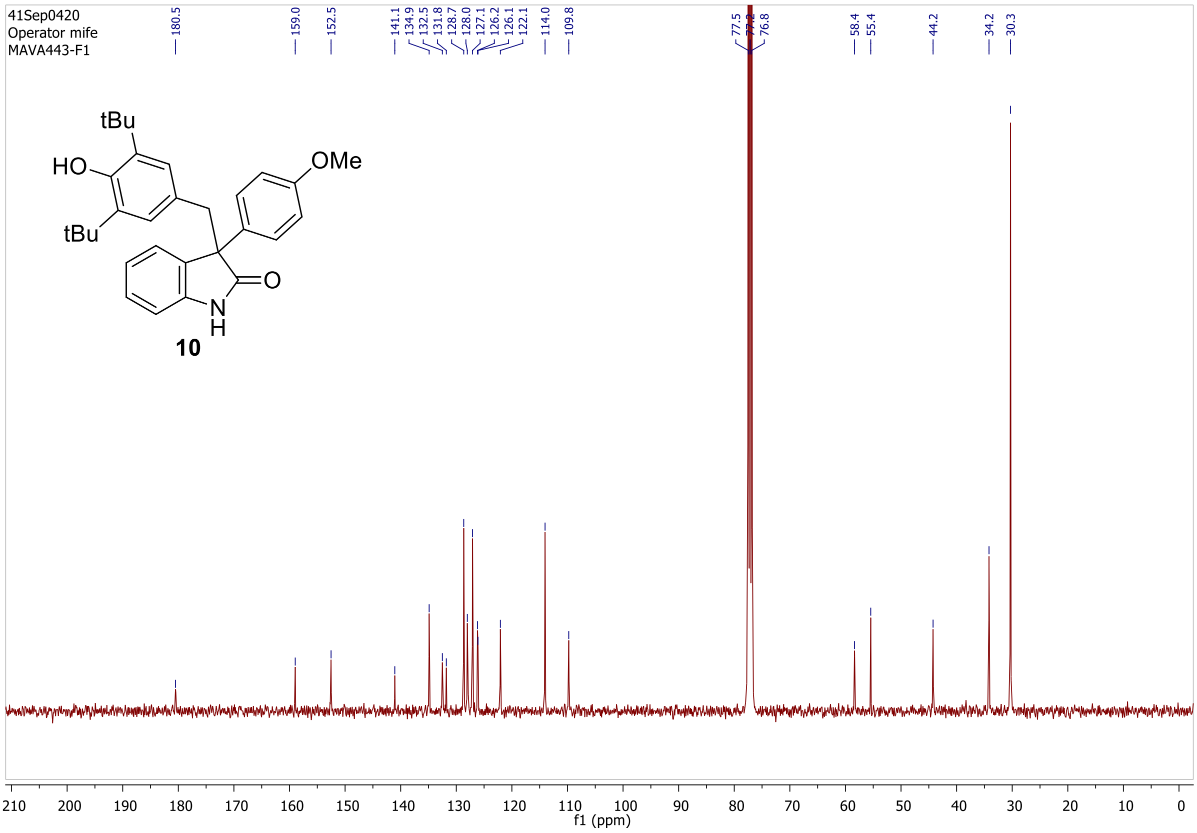

$^{13}C\{^{1}H\}$ NMR in $CDCl_3$ at 100 MHz

10. NMR Spectra

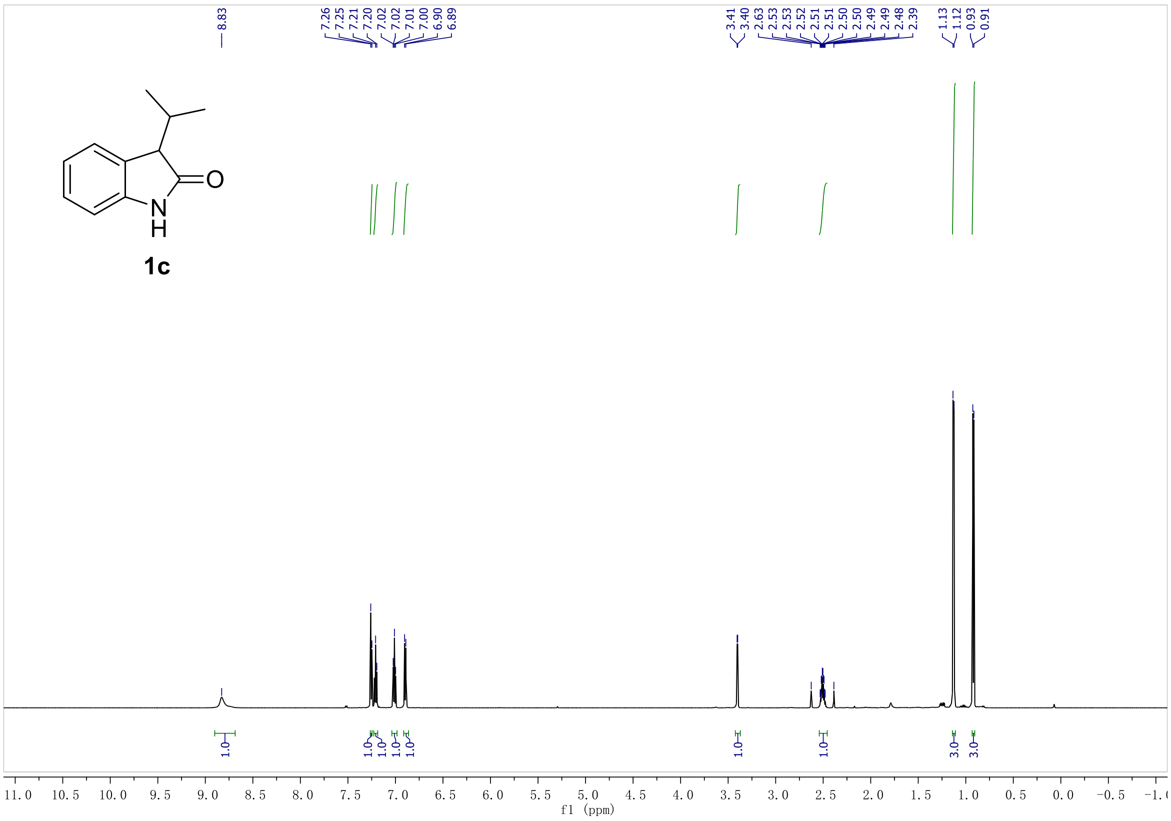

^{1}H NMR in $CDCl_3$ at 600 MHz

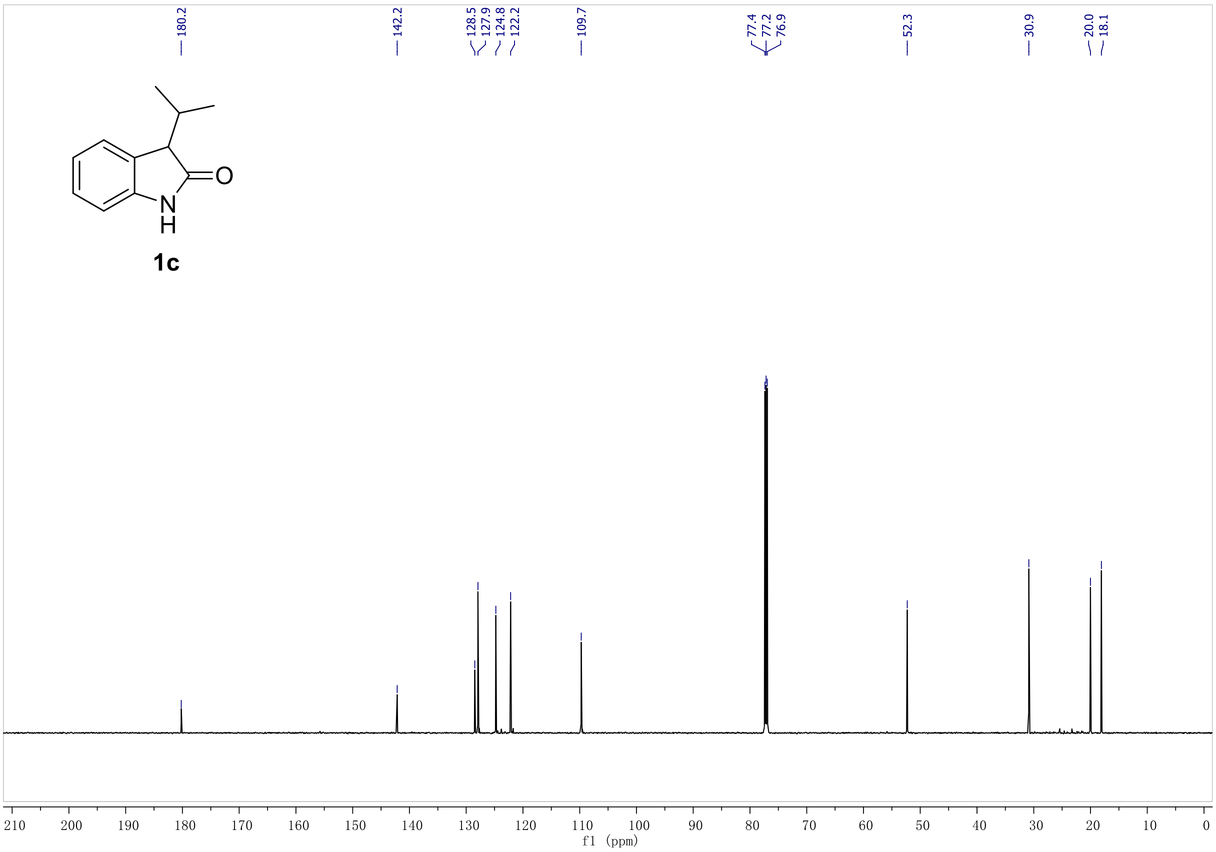

^{13}C{^{1}H} NMR in $CDCl_3$ at 150 MHz

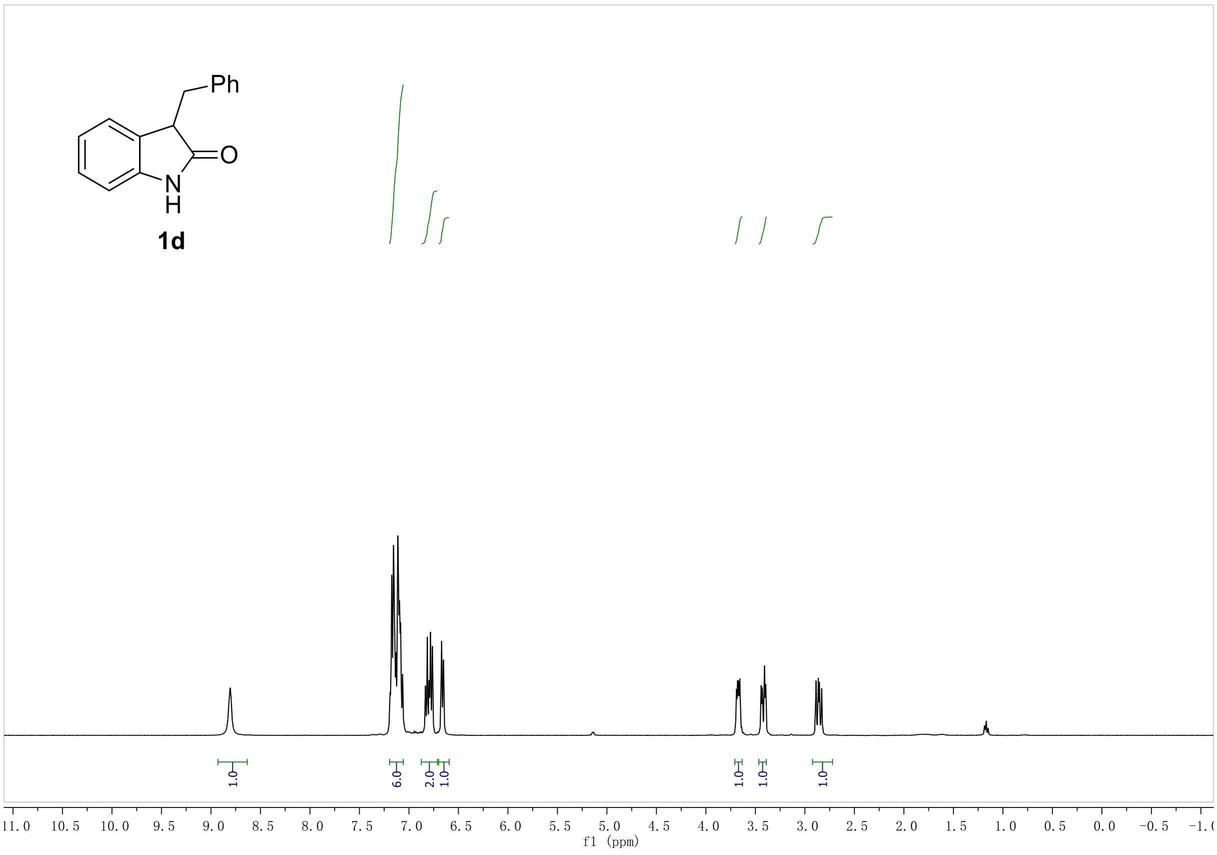

^{1}H NMR in $CDCl_3$ at 400 MHz

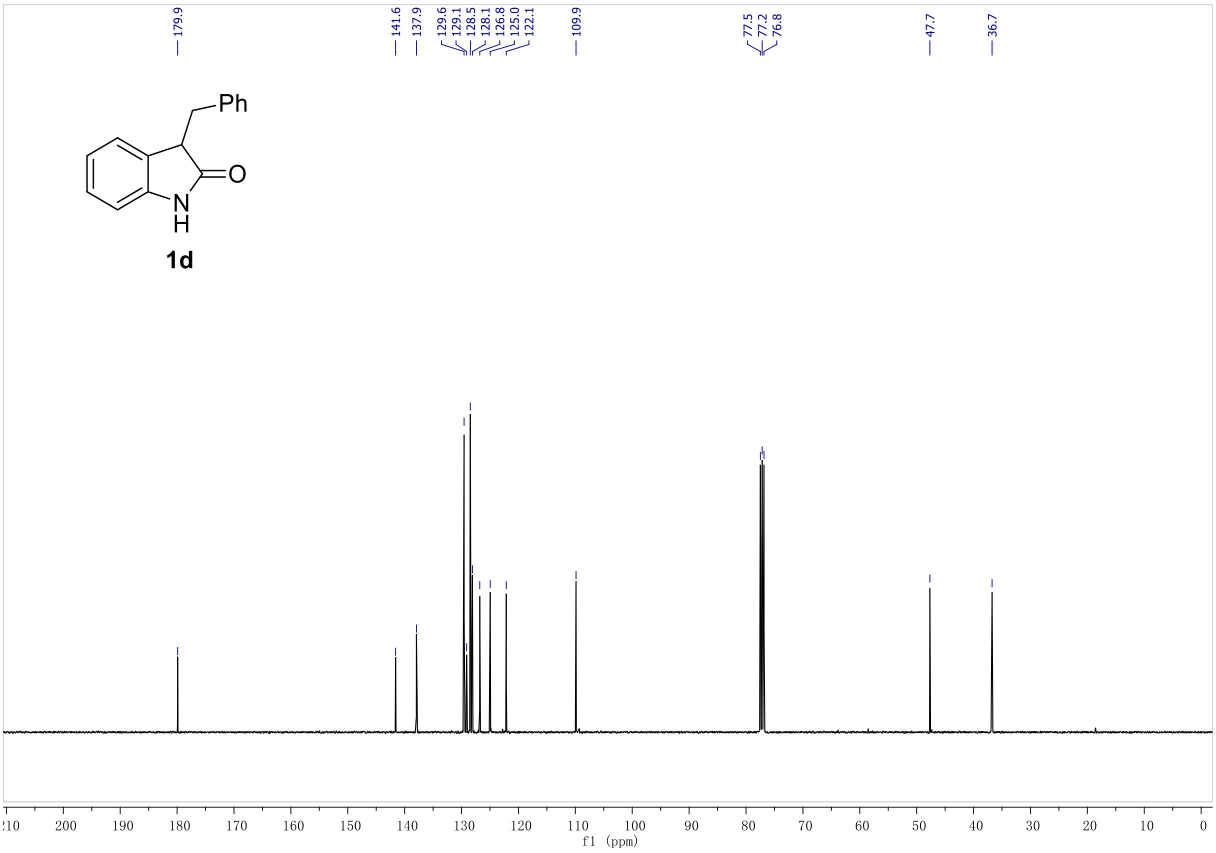

^{13}C{^{1}H} NMR in $CDCl_3$ at 100 MHz

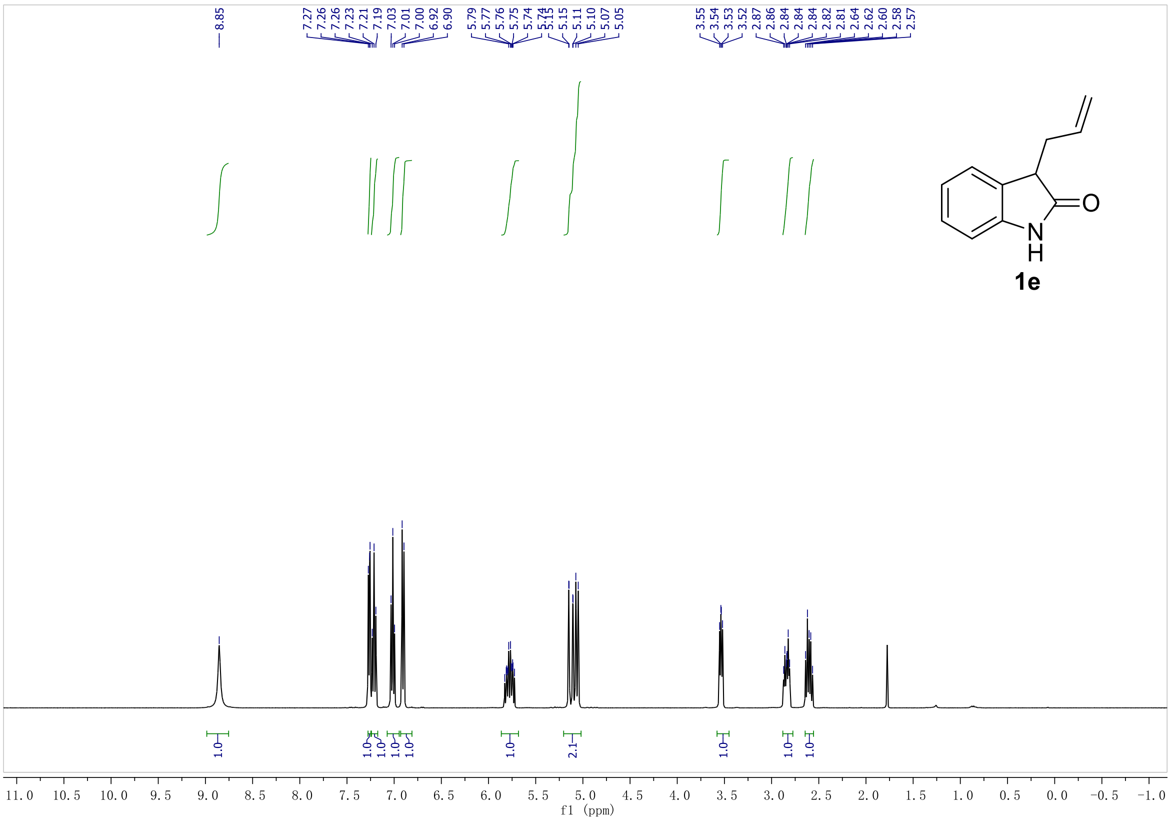

^{1}H NMR in $CDCl_3$ at 400 MHz

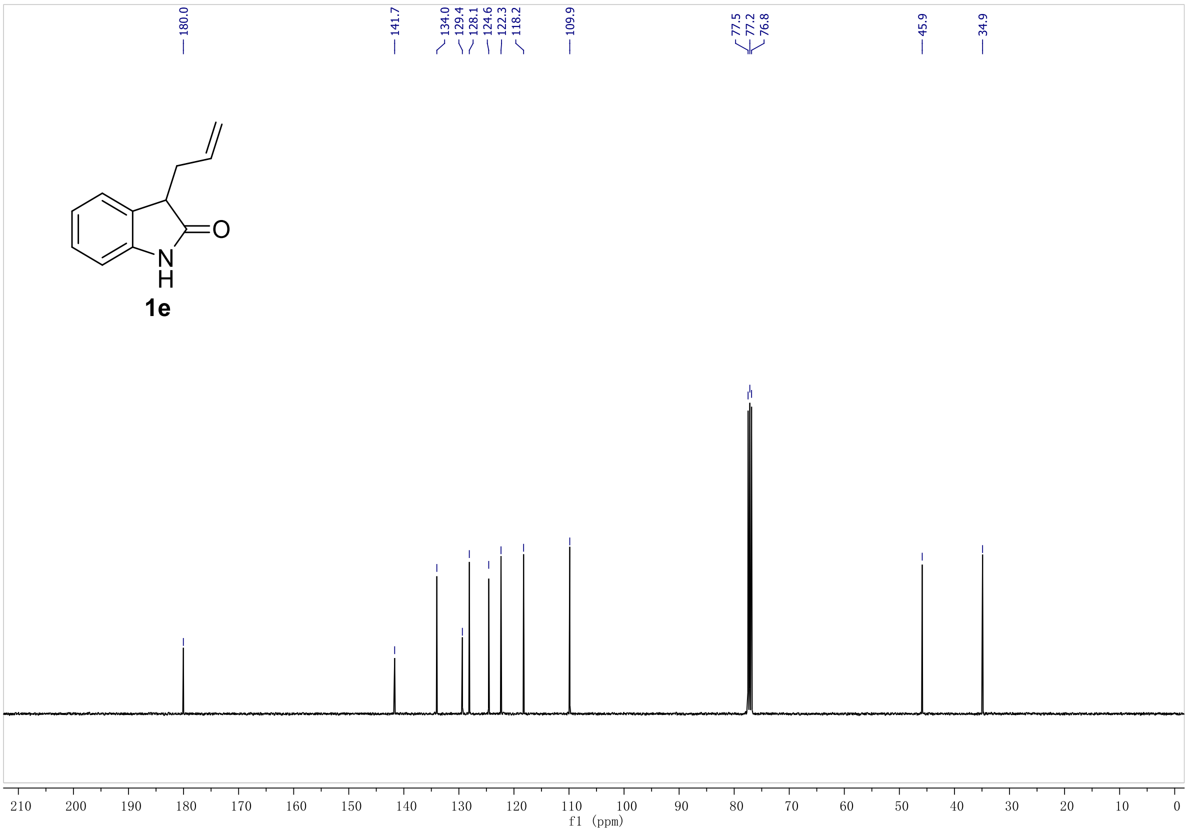

$^{13}C\{^{1}H\}$ NMR in $CDCl_3$ at 100 MHz

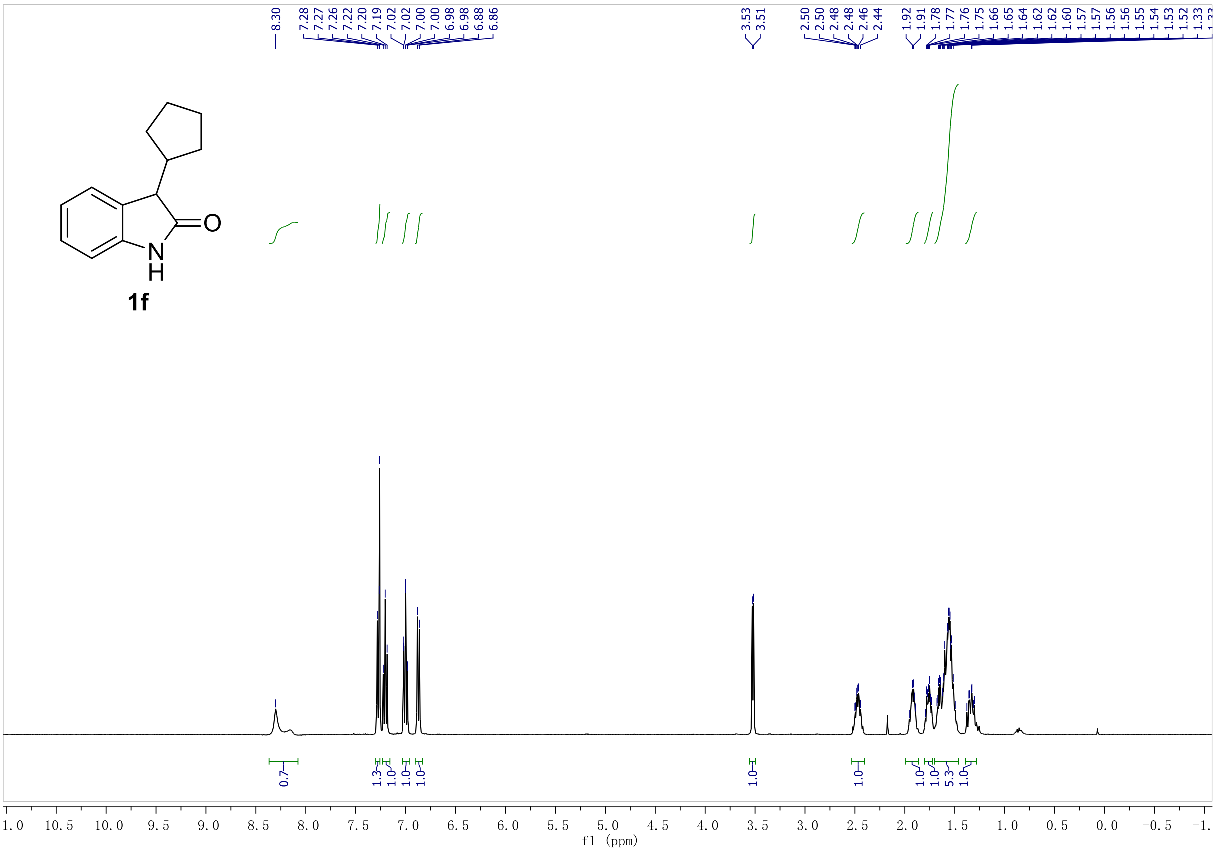

^{1}H NMR in $CDCl_3$ at 400 MHz

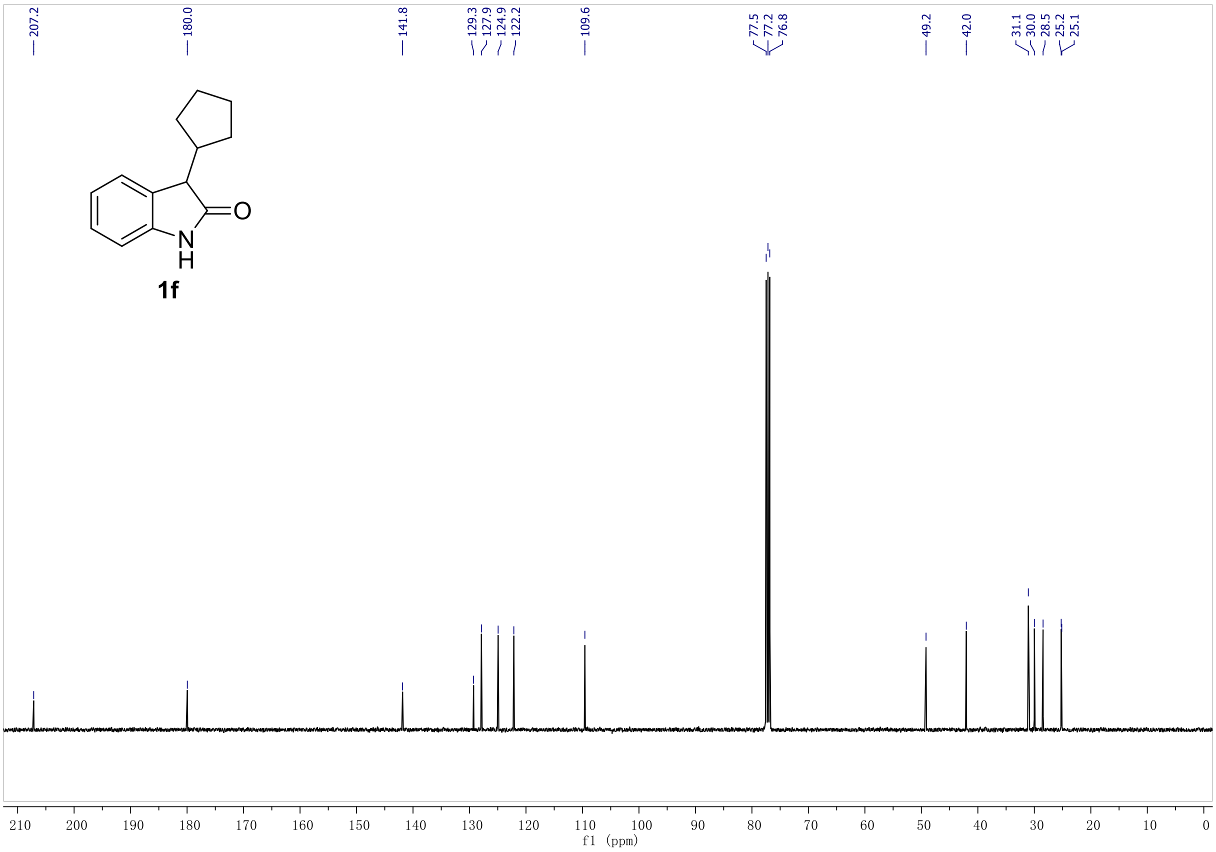

$^{13}C\{^{1}H\}$ NMR in $CDCl_3$ at 100 MHz

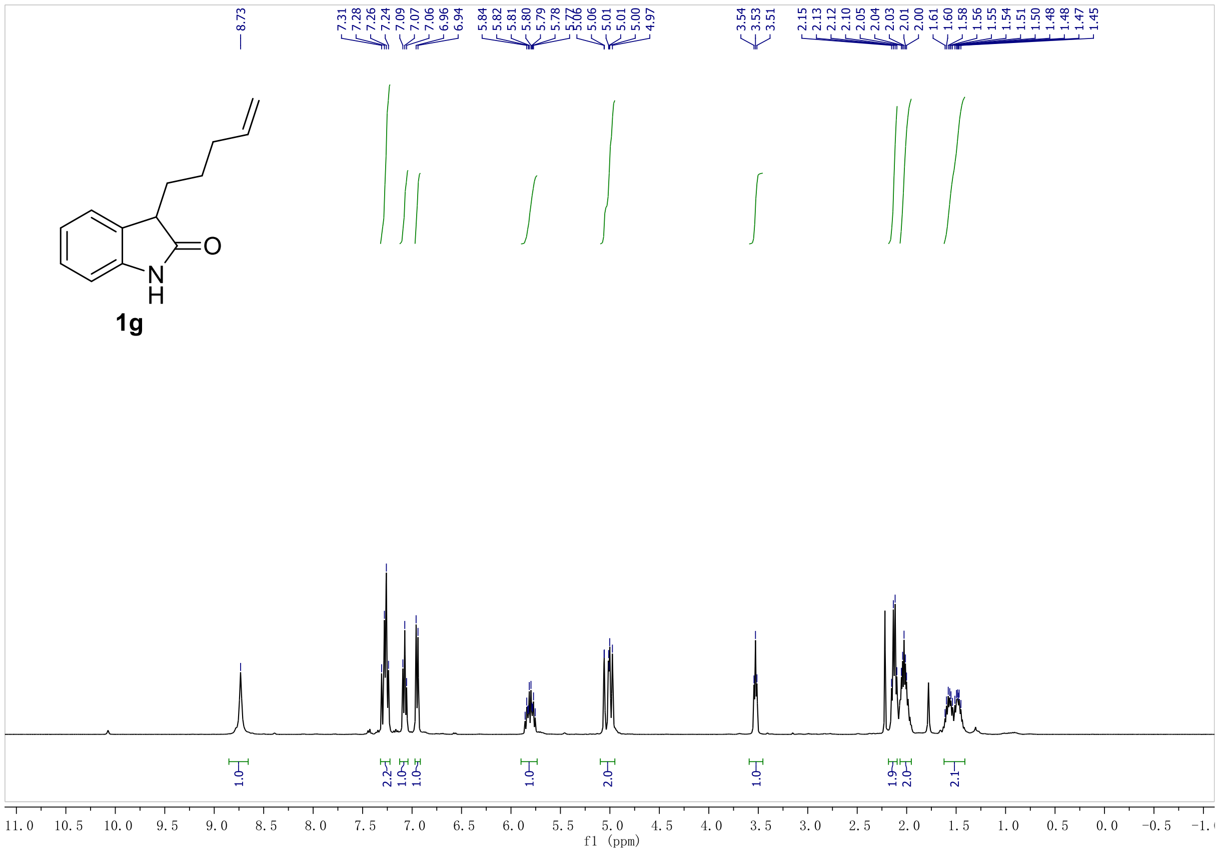

^{1}H NMR in $CDCl_3$ at 400 MHz

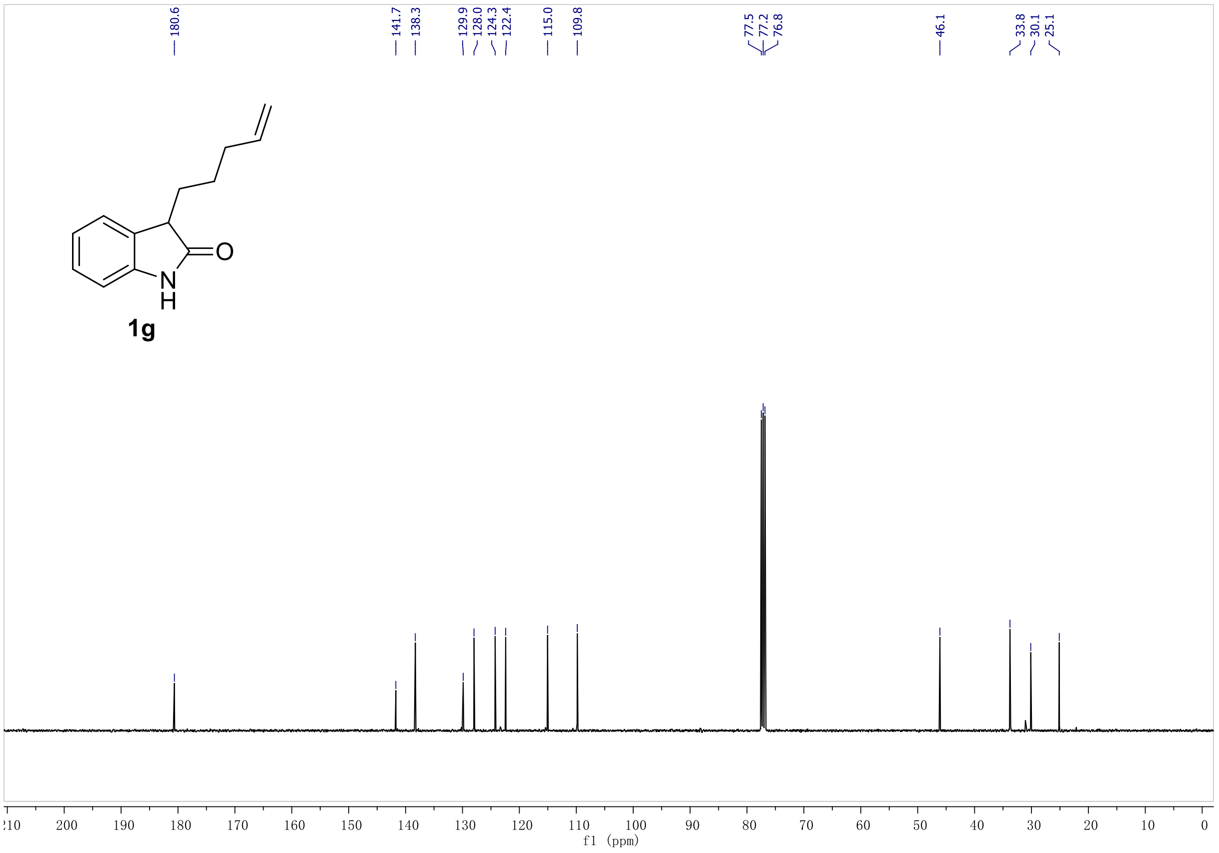

$^{13}C\{^{1}H\}$ NMR in $CDCl_3$ at 100 MHz

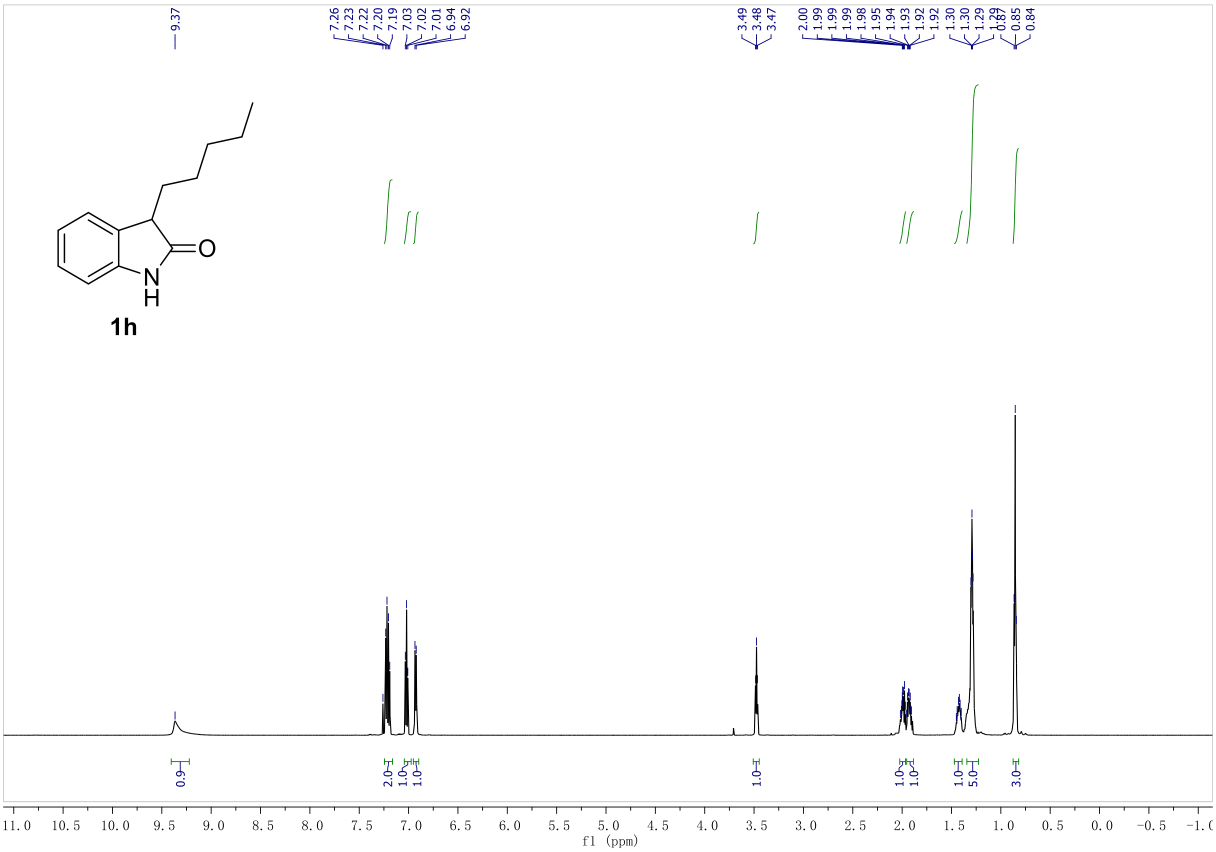

^{1}H NMR in $CDCl_3$ at 600 MHz

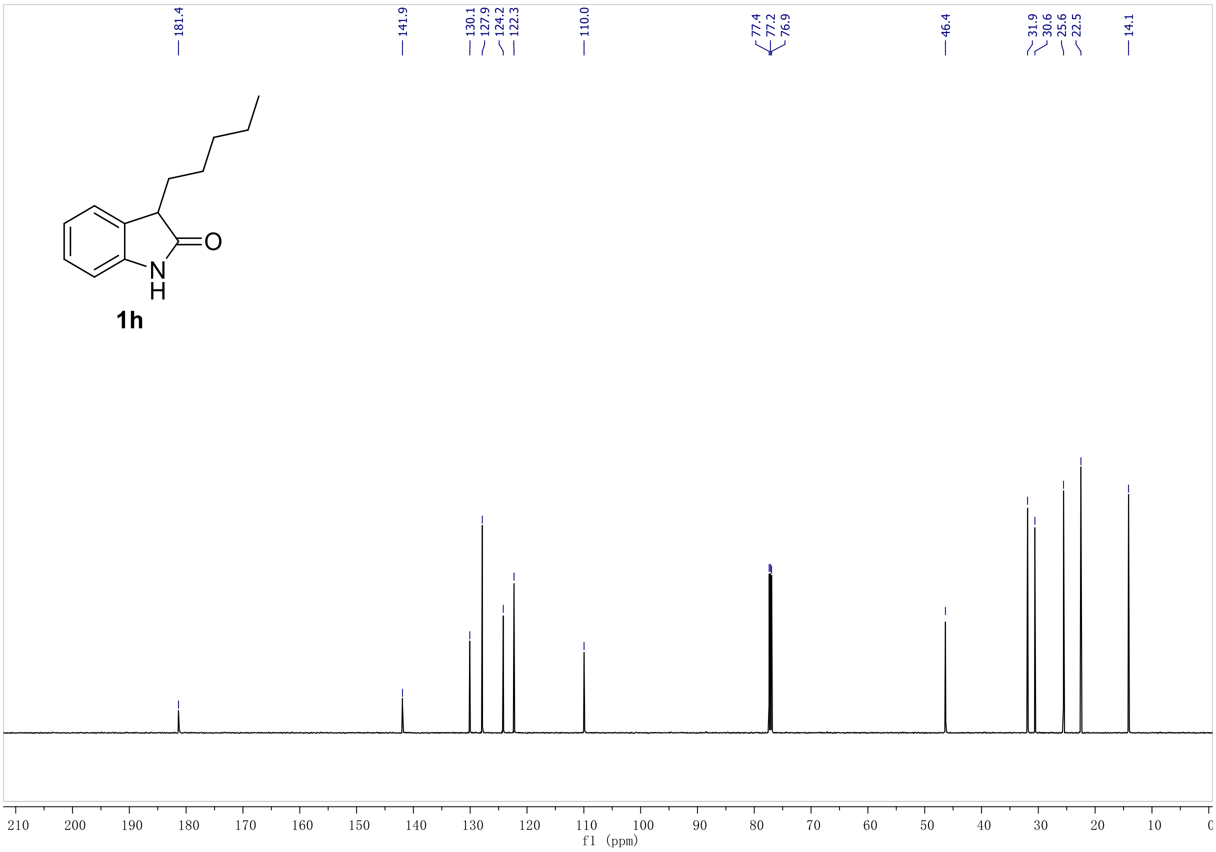

$^{13}C\{^{1}H\}$ NMR in $CDCl_3$ at 150 MHz

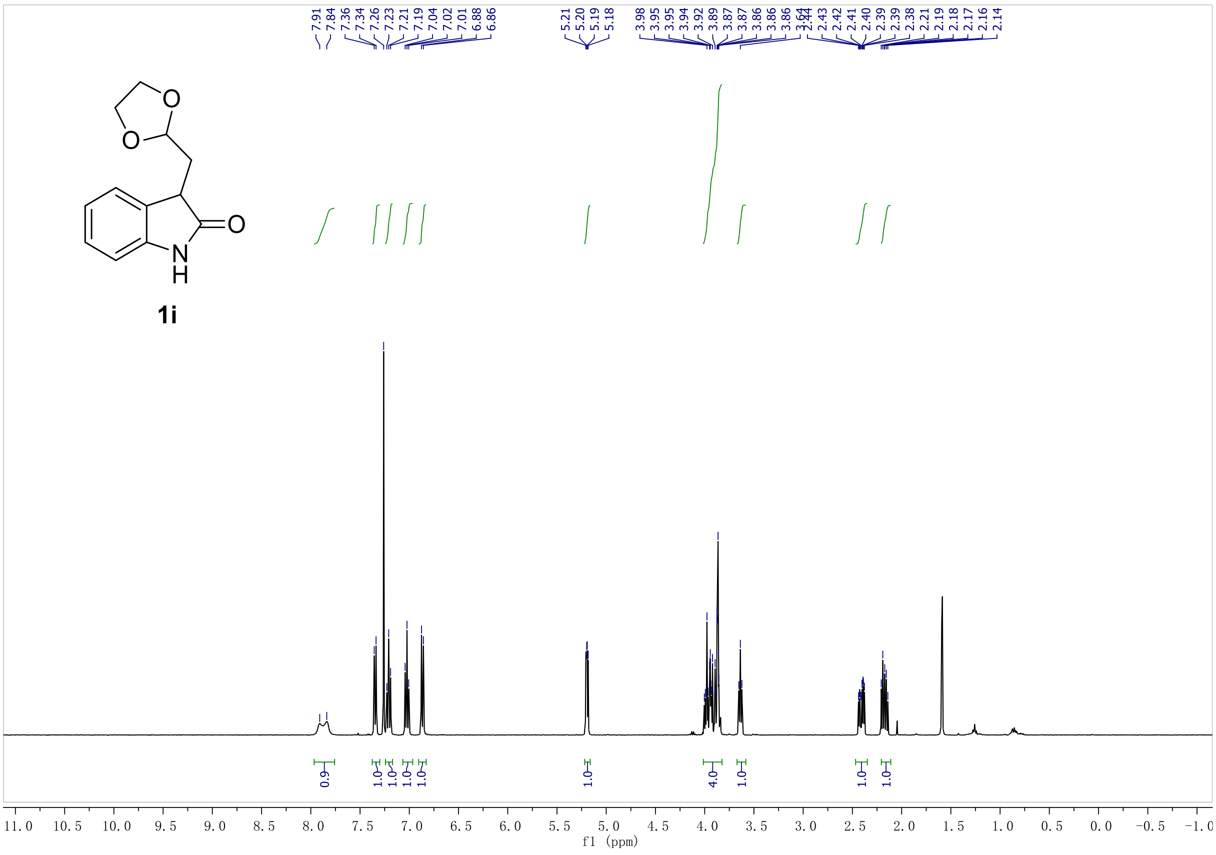

^{1}H NMR in $CDCl_3$ at 400 MHz

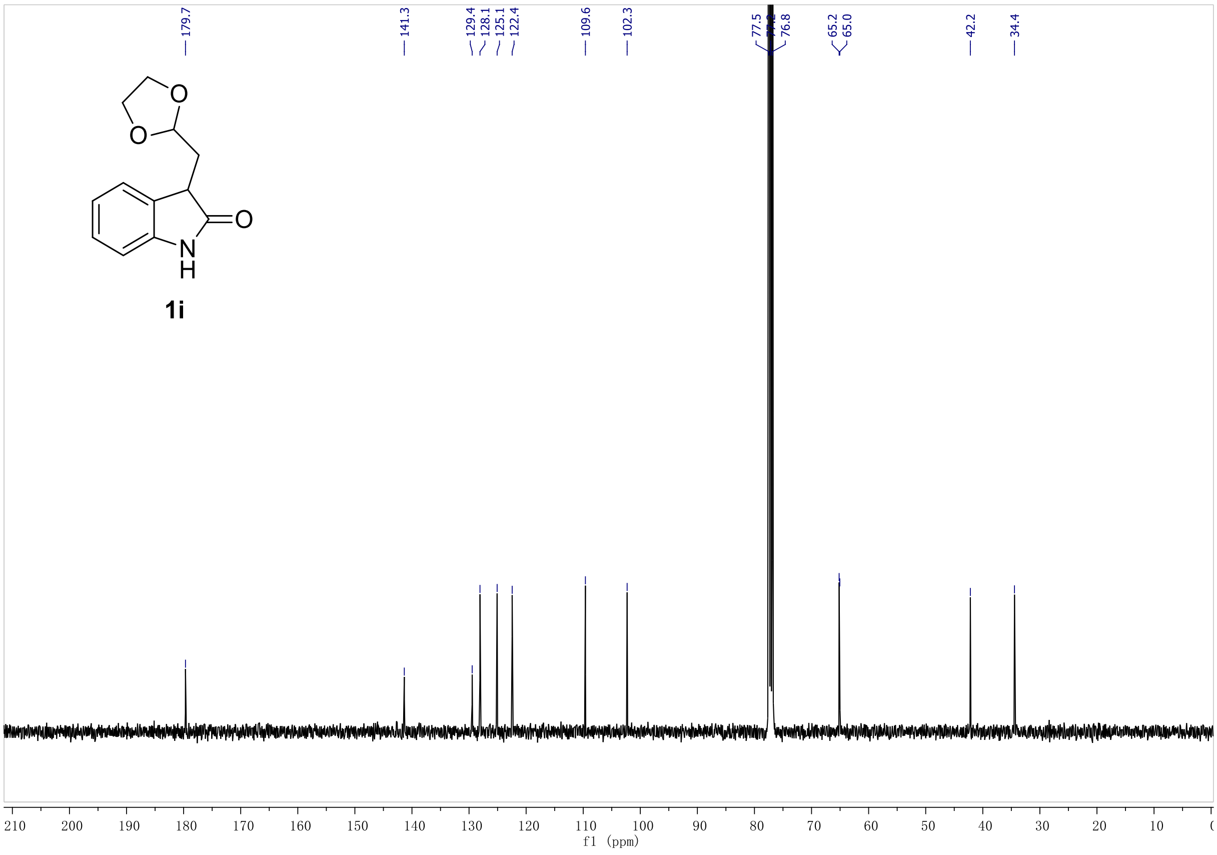

$^{13}C\{^{1}H\}$ NMR in $CDCl_3$ at 100 MHz

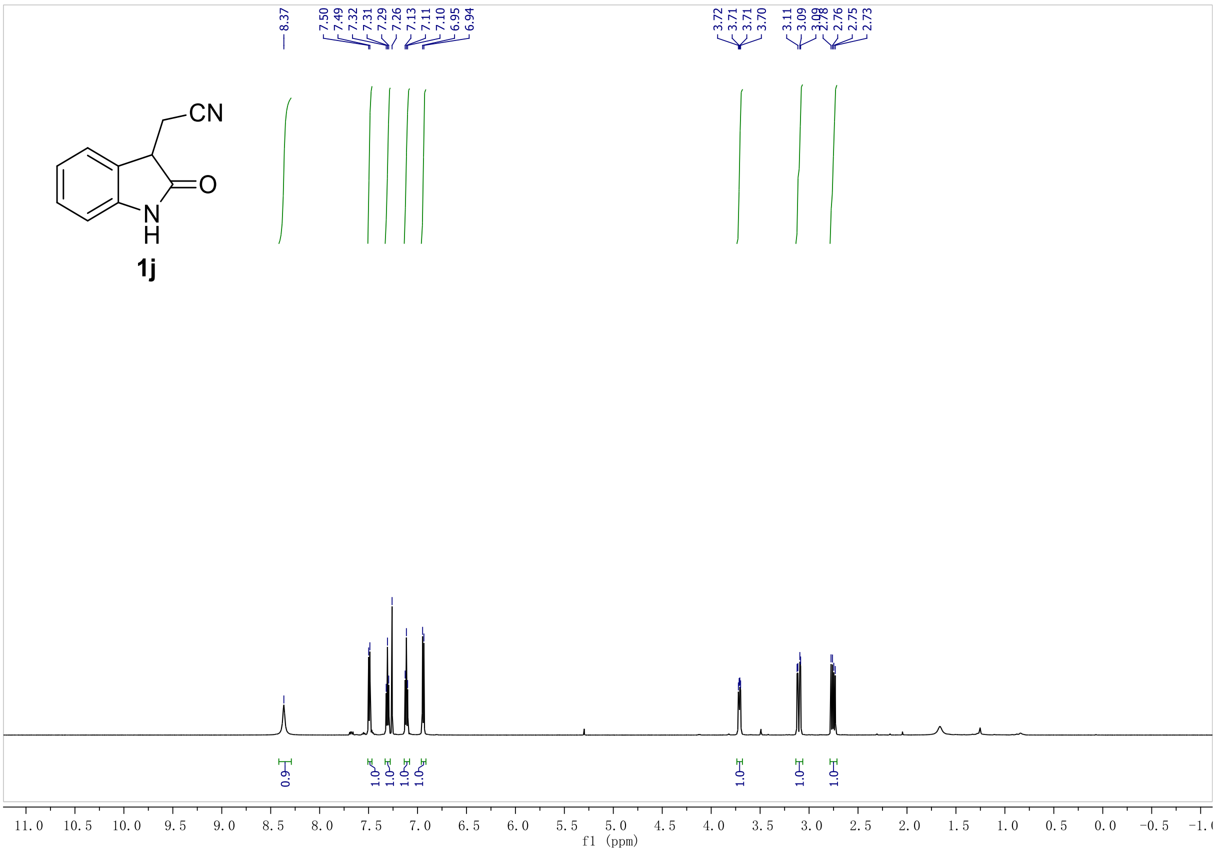

^{1}H NMR in $CDCl_3$ at 600 MHz

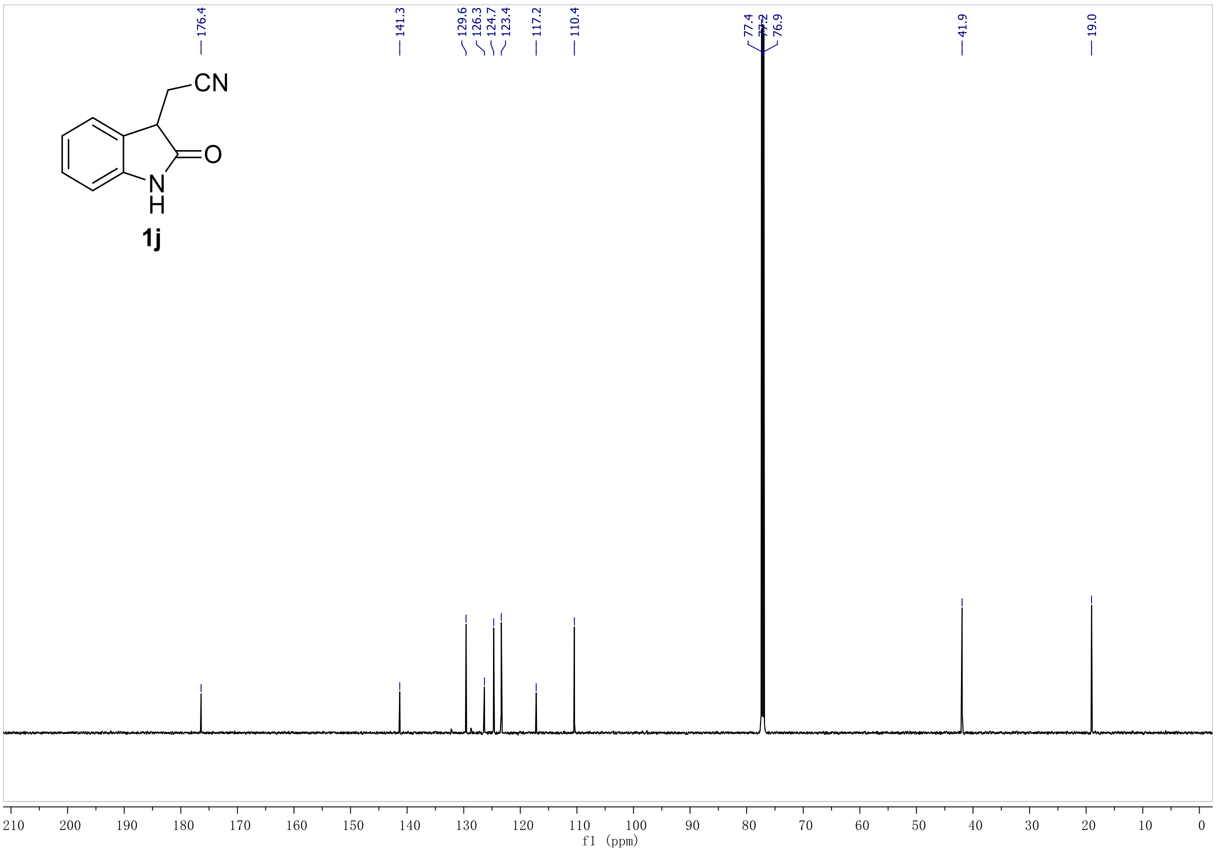

$^{13}C\{^{1}H\}$ NMR in $CDCl_3$ at 150 MHz

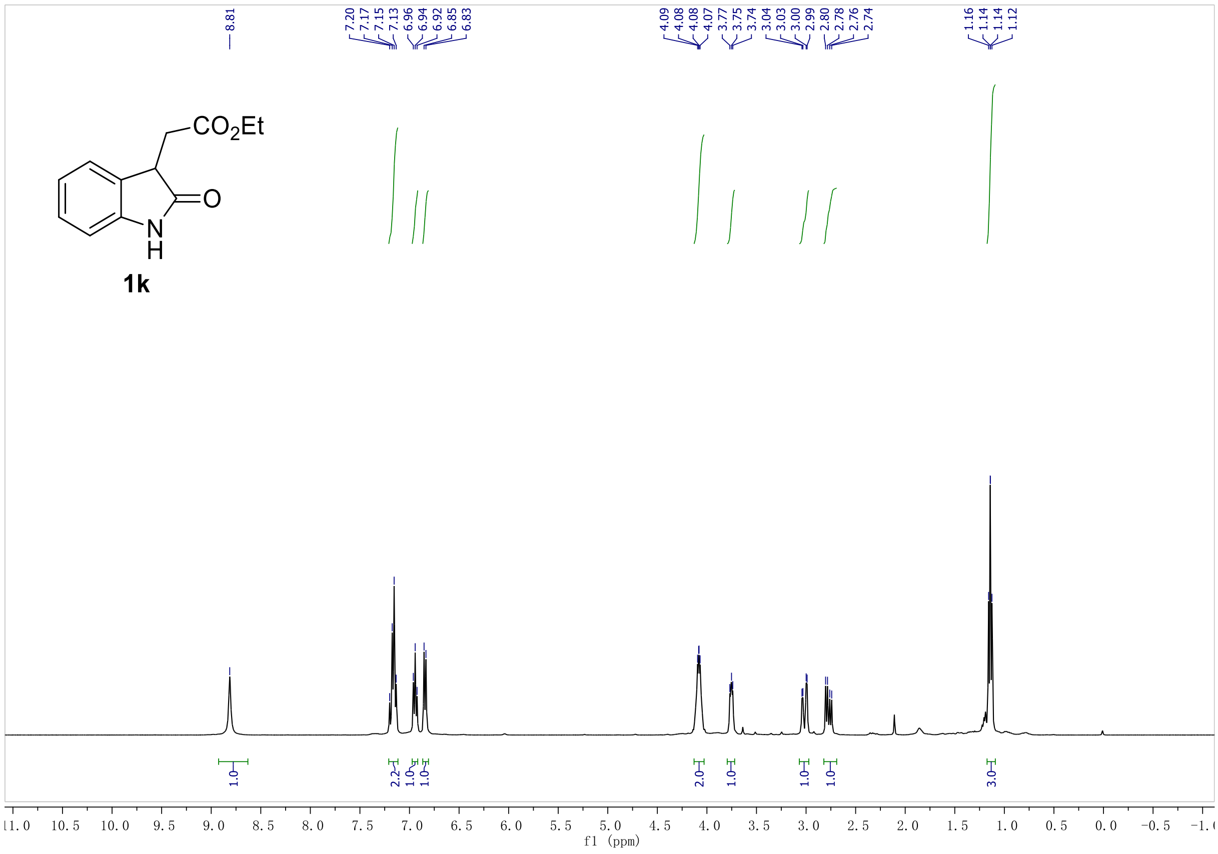

^{1}H NMR in $CDCl_3$ at 400 MHz

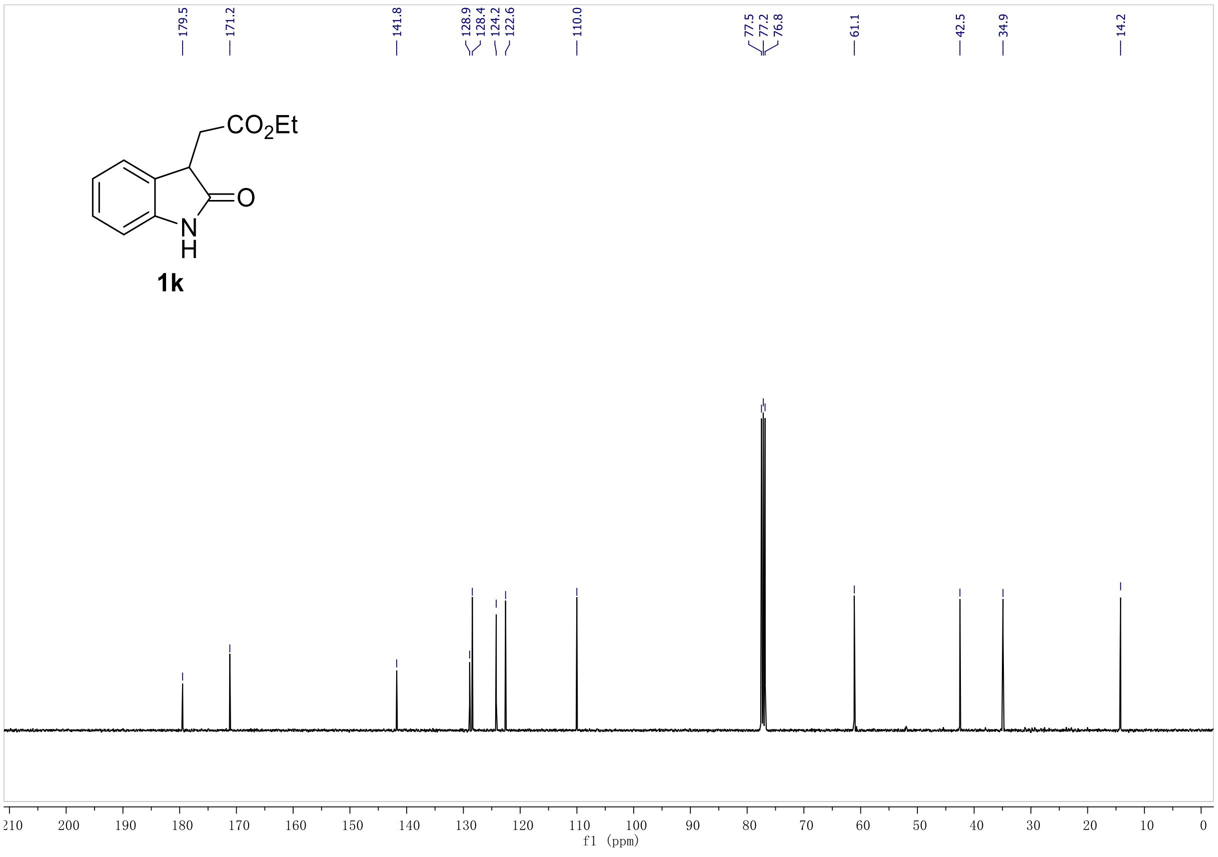

$^{13}C\{^{1}H\}$ NMR in $CDCl_3$ at 100 MHz

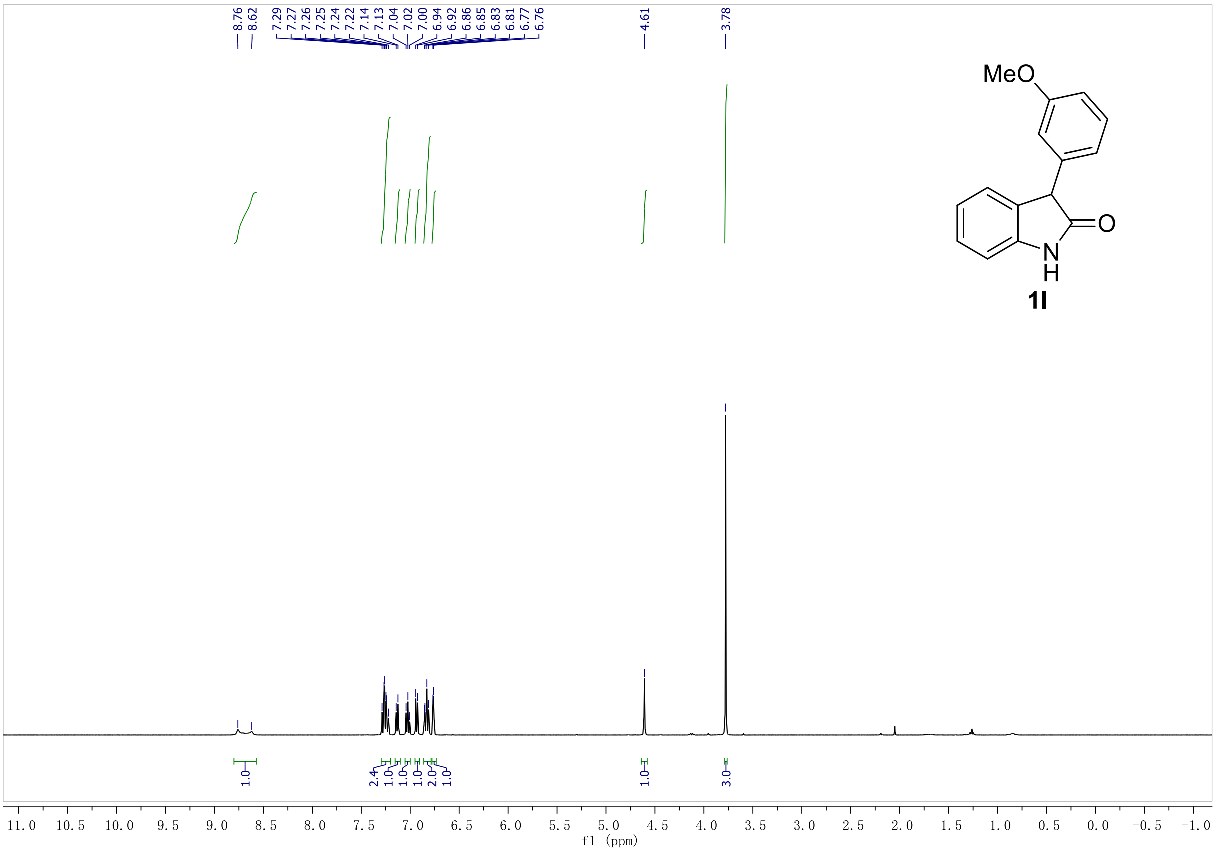

^{1}H NMR in $CDCl_3$ at 400 MHz

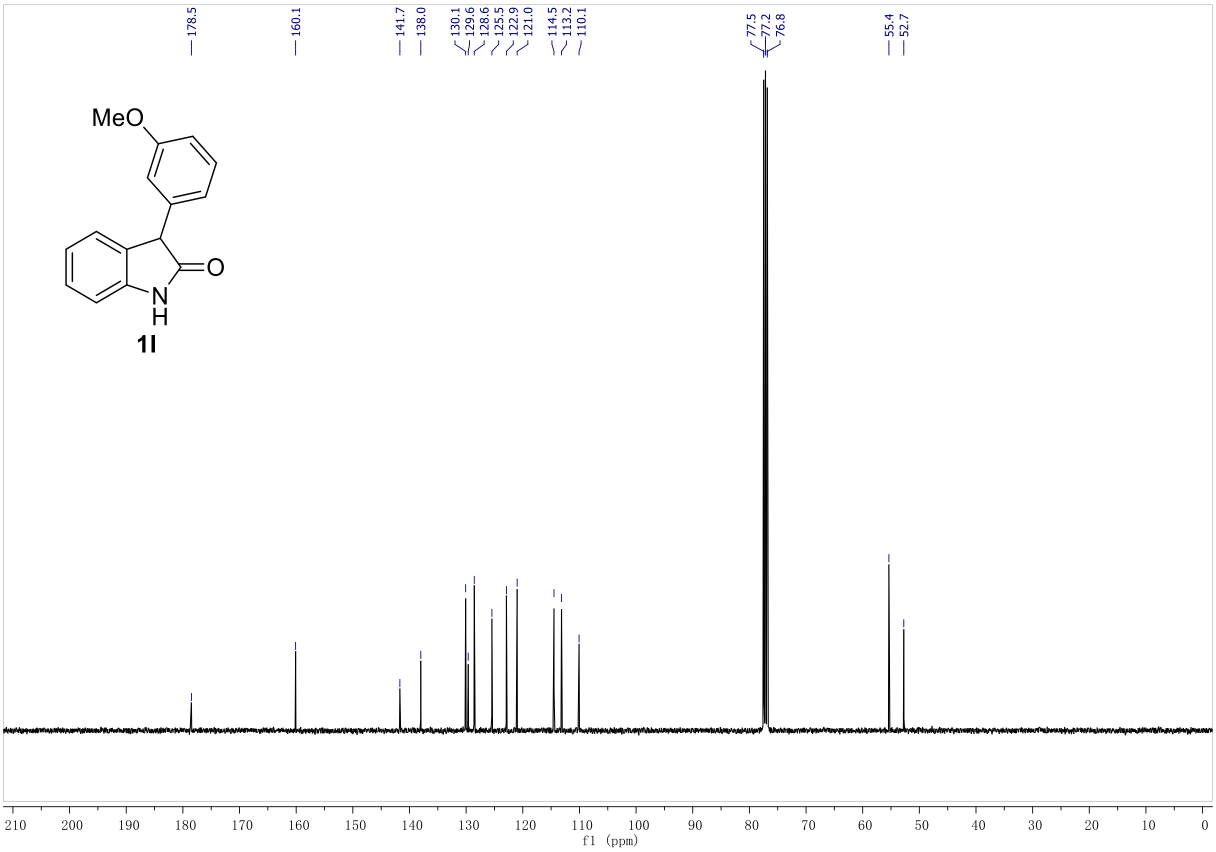

$^{13}C\{^{1}H\}$ NMR in $CDCl_3$ at 100 MHz

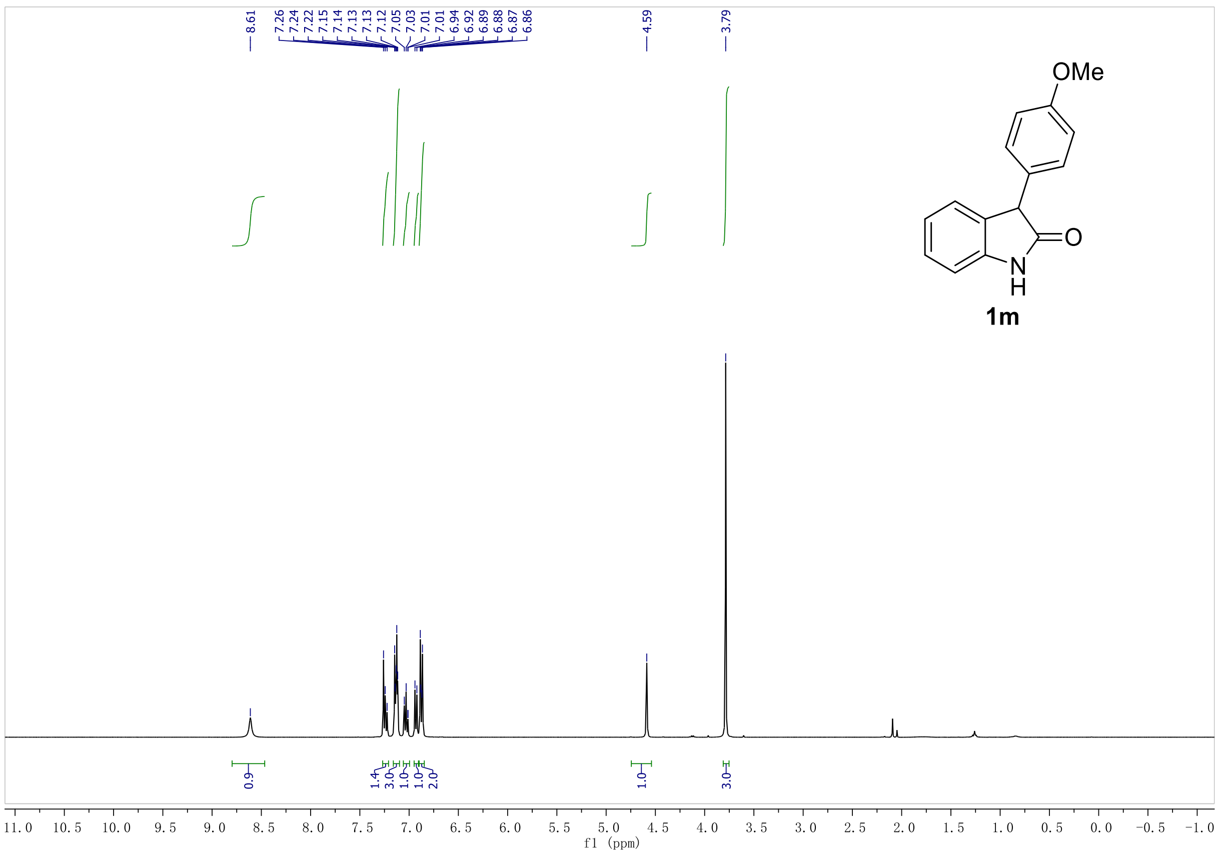

^{1}H NMR in $CDCl_3$ at 400 MHz

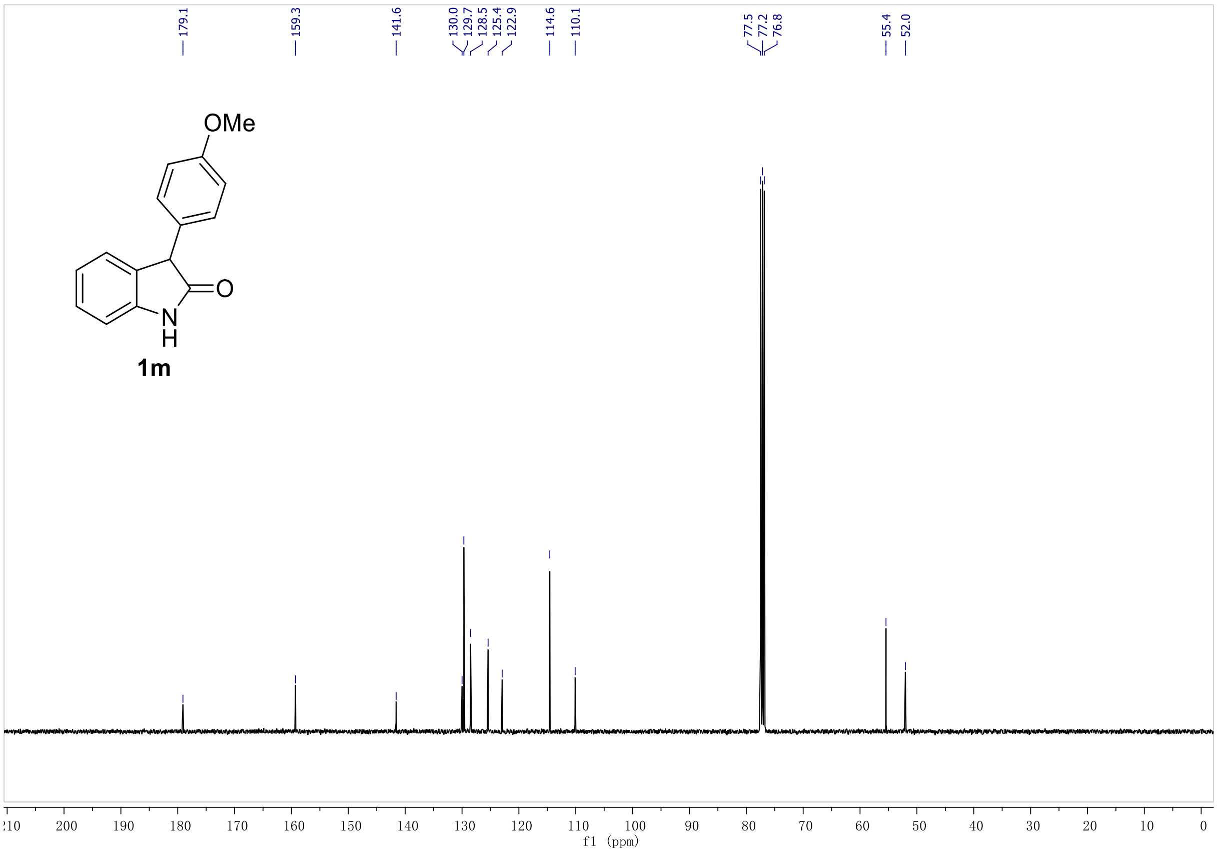

$^{13}C\{^{1}H\}$ NMR in $CDCl_3$ at 100 MHz

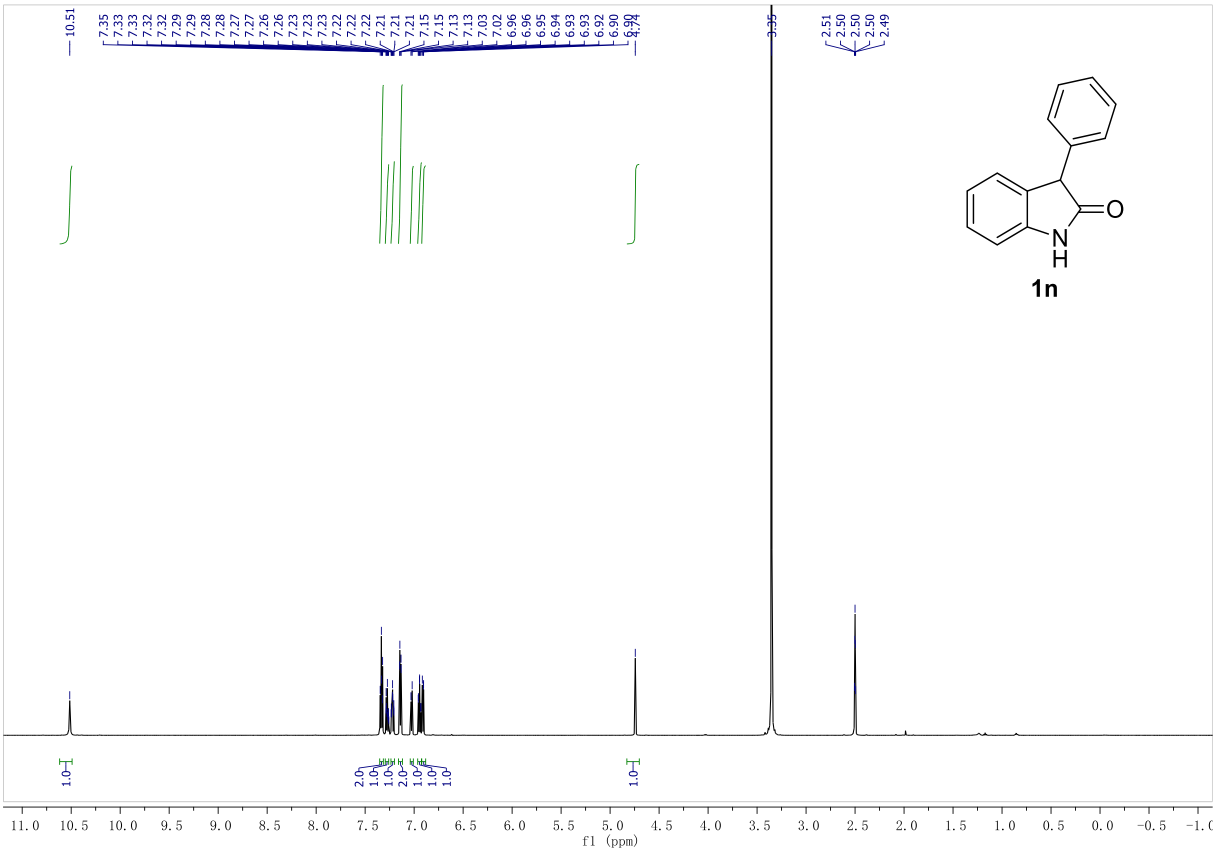

^{1}H NMR in DMSO-d_6 at 600 MHz

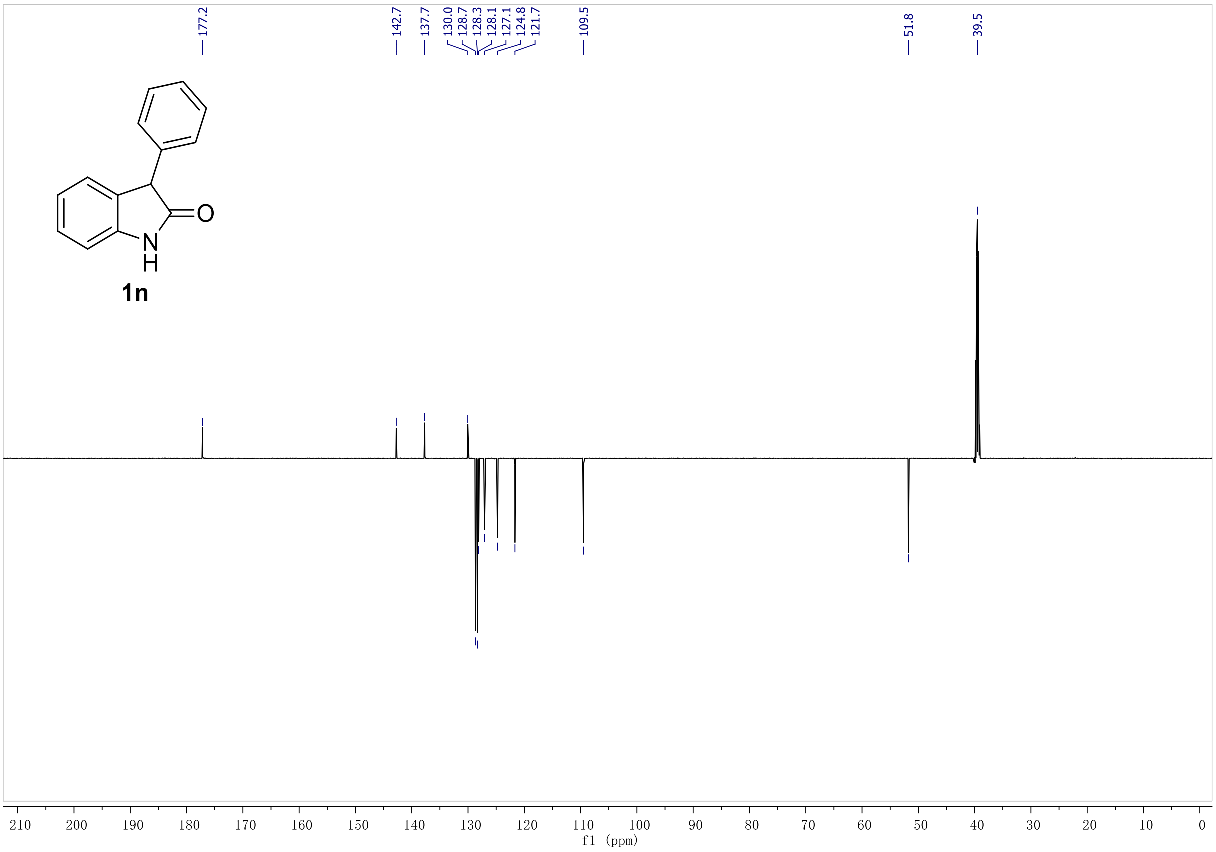

$^{13}C\{^{1}H\}$ NMR in DMSO-d_6 at 150 MHz

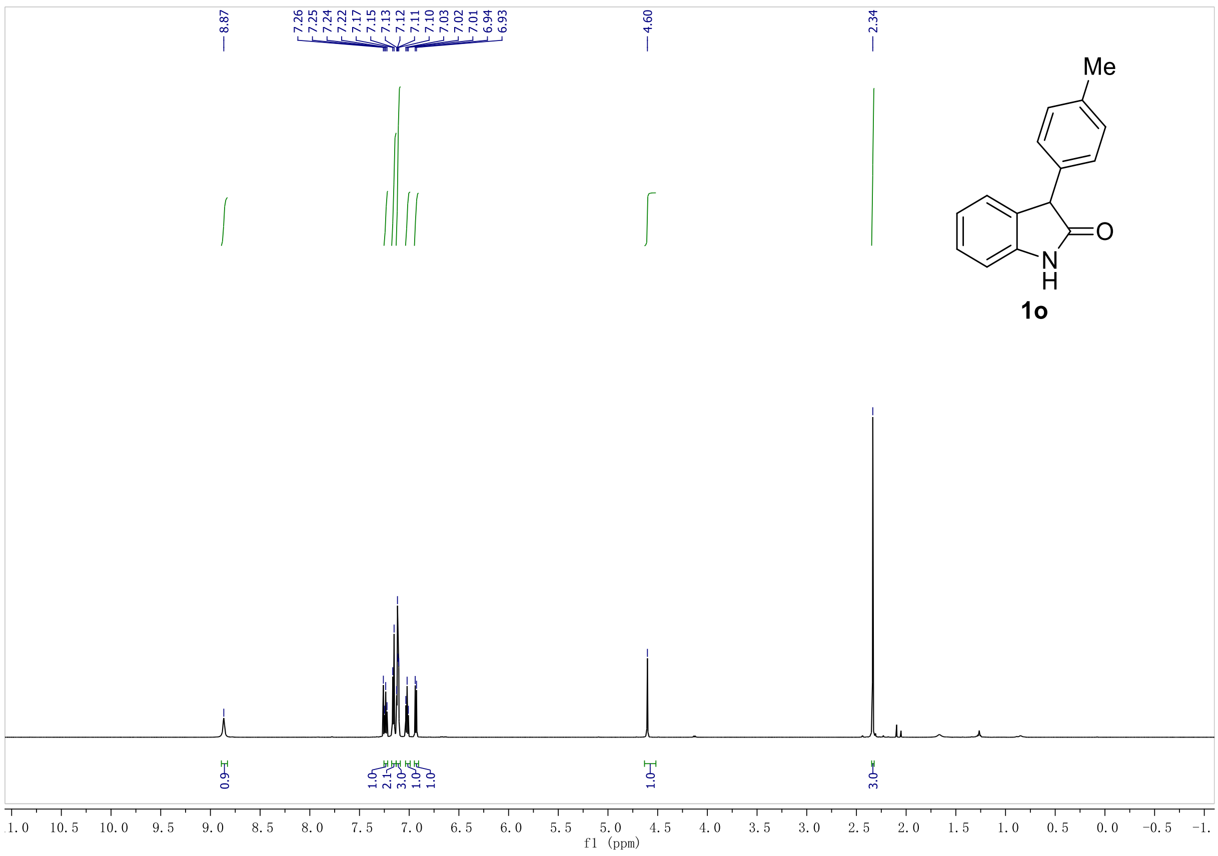

^{1}H NMR in $CDCl_3$ at 600 MHz

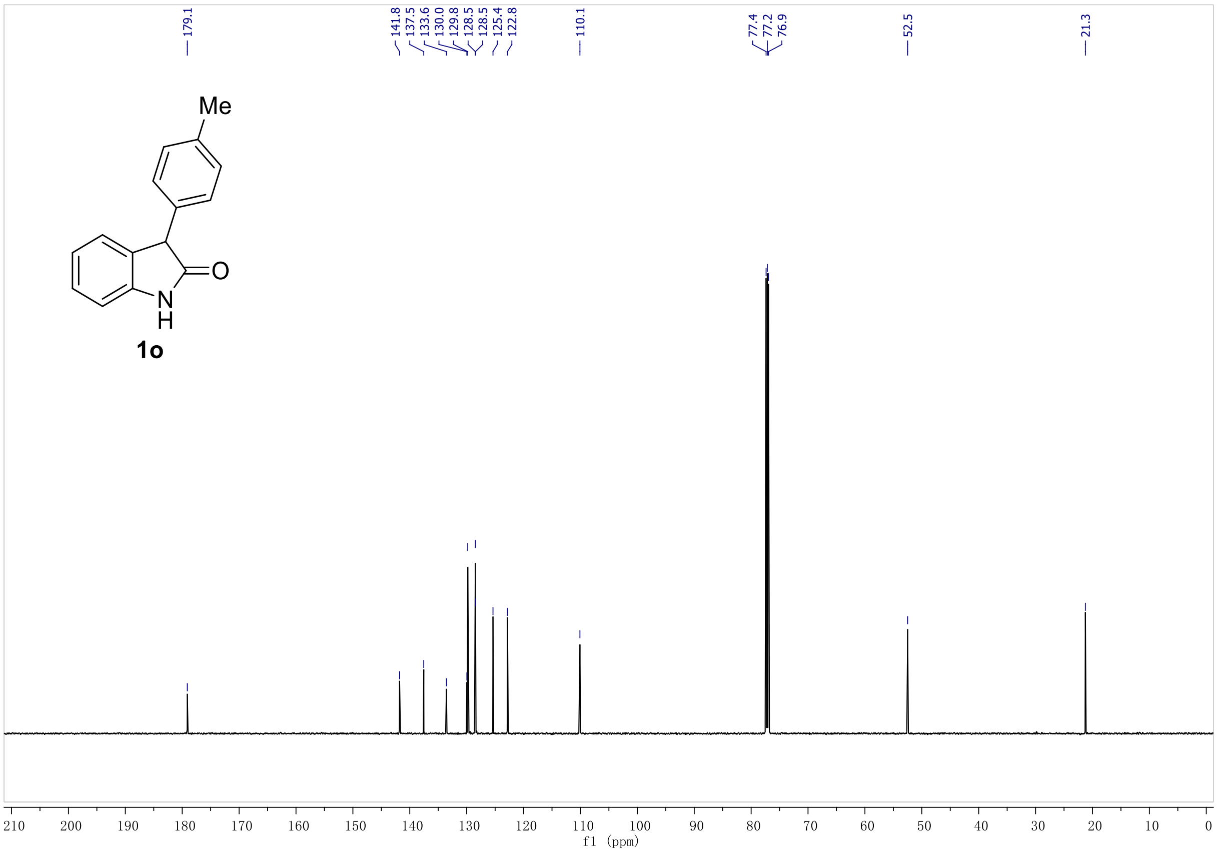

$^{13}C\{^{1}H\}$ NMR in $CDCl_3$ at 150 MHz

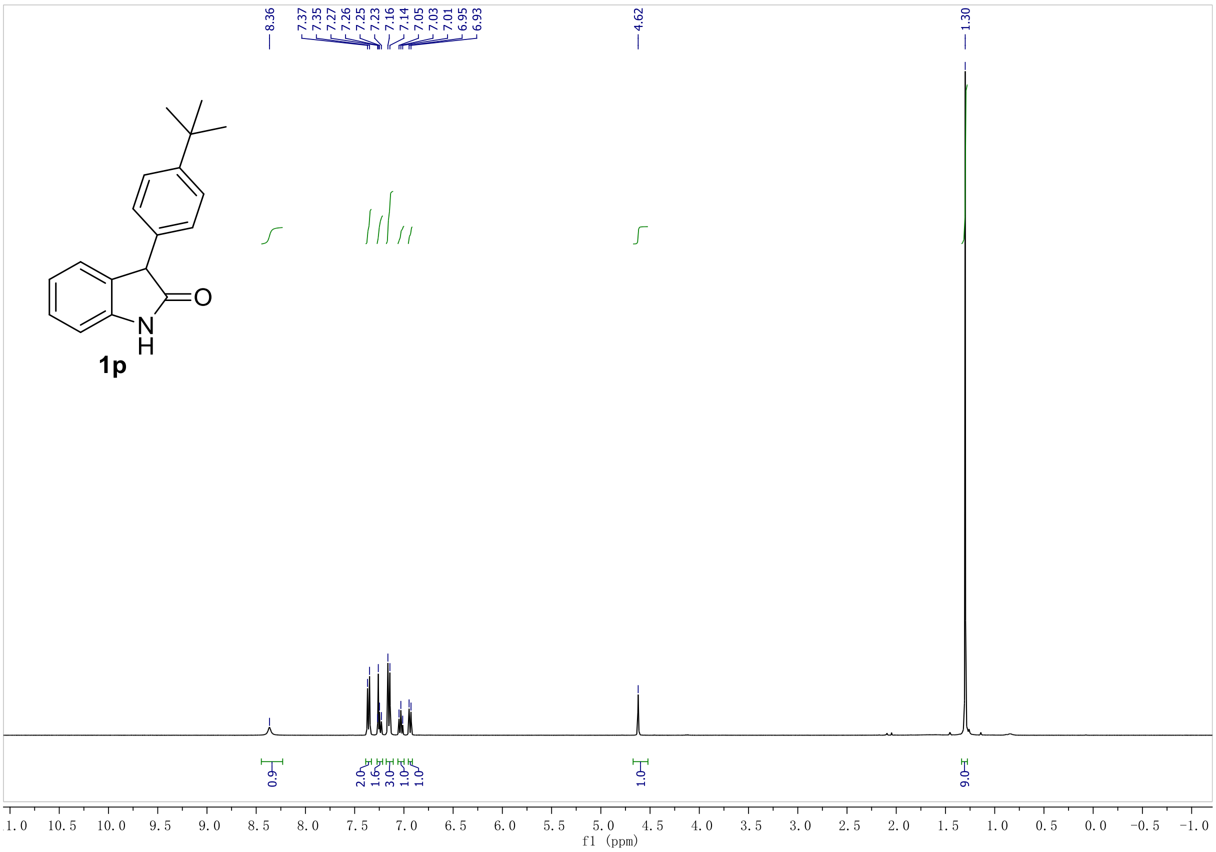

^{1}H NMR in $CDCl_3$ at 600 MHz

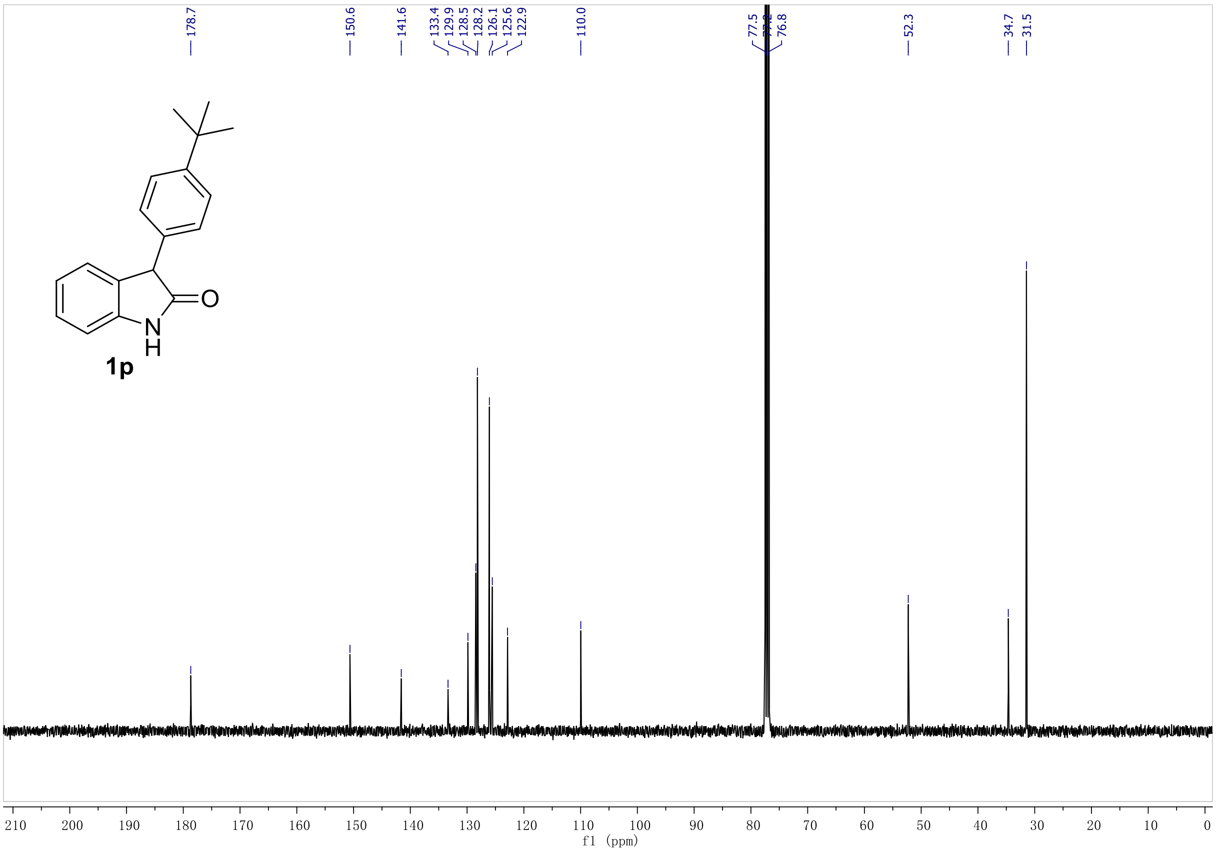

$^{13}C\{^1H\}$ NMR in $CDCl_3$ at 100 MHz

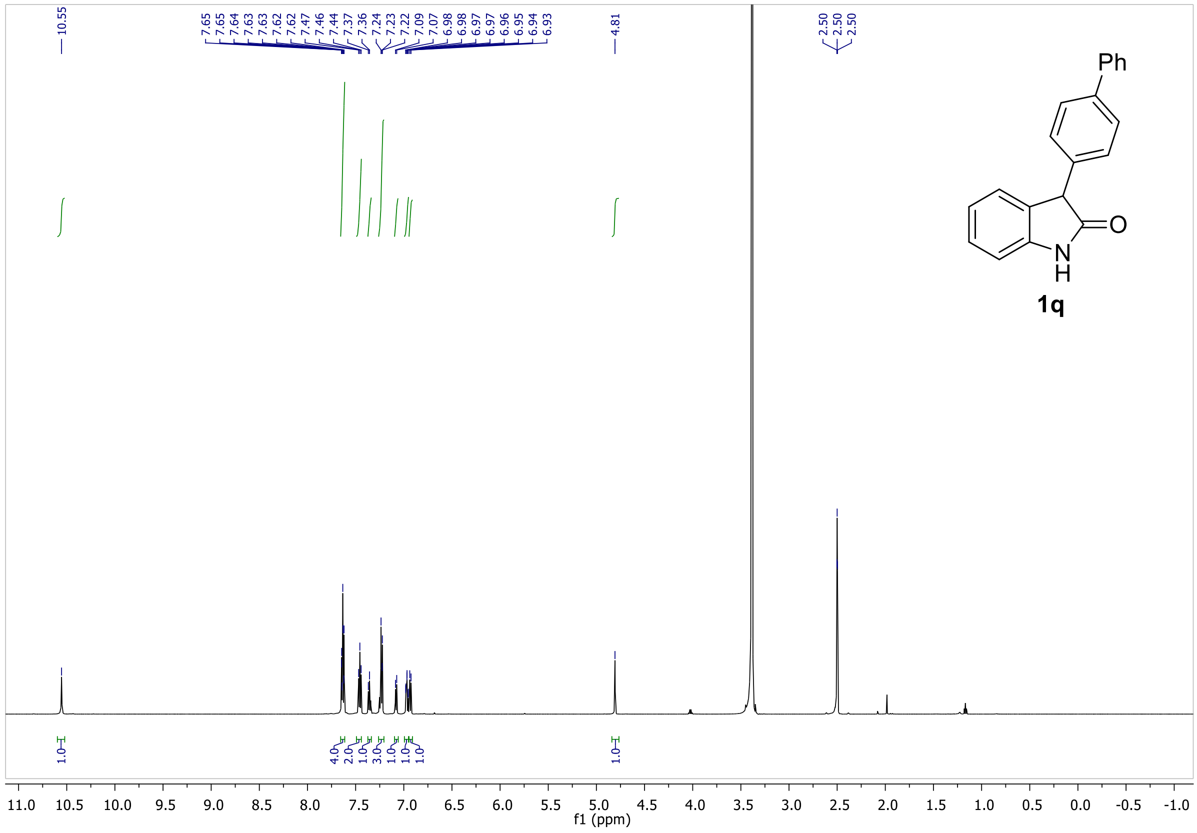

^{1}H NMR in DMSO-d_6 at 600 MHz

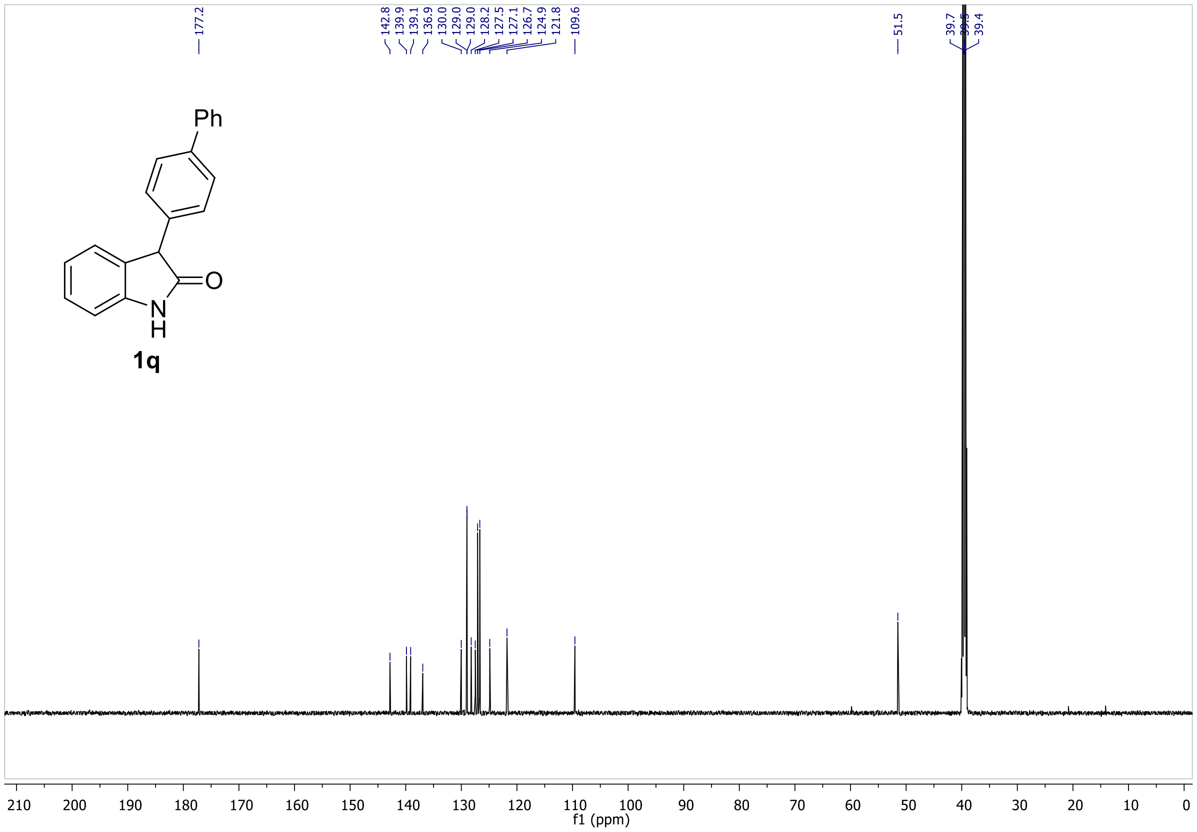

$^{13}C\{^{1}H\}$ NMR in DMSO-d_6 at 150 MHz

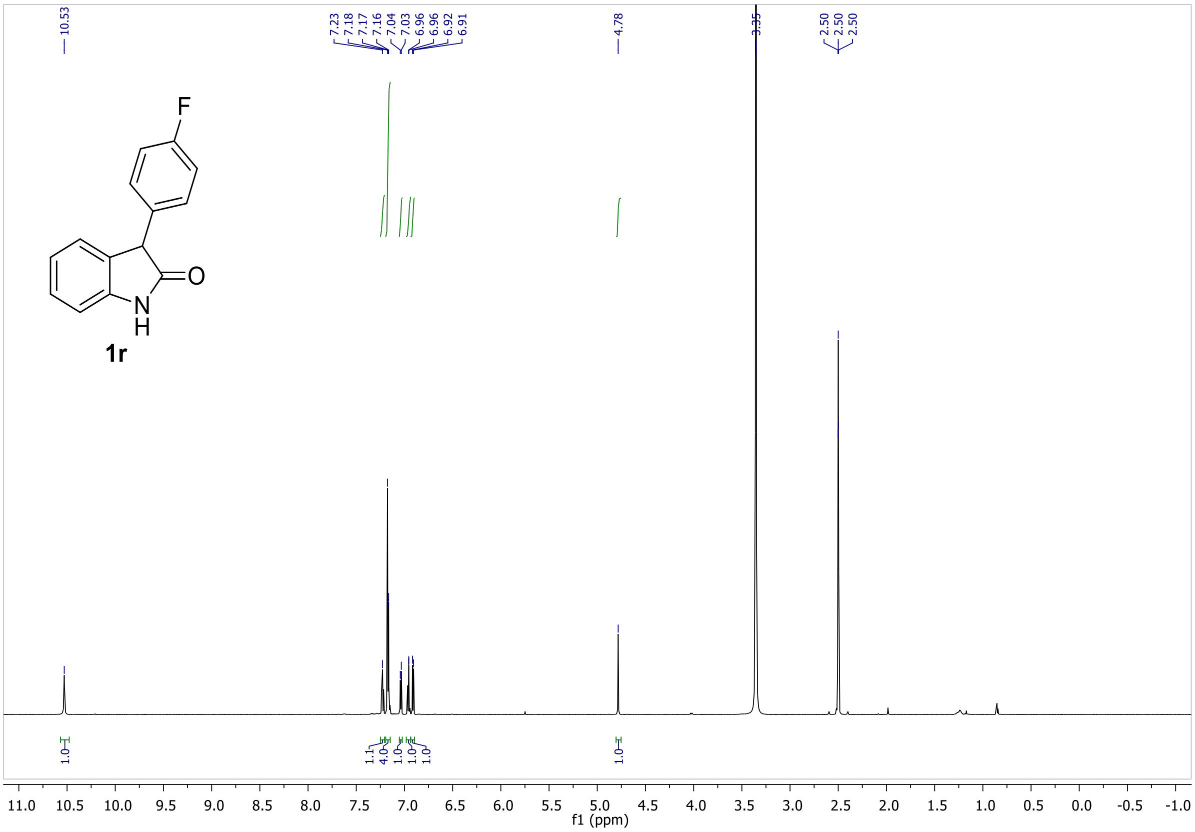

^{1}H NMR in DMSO-d_6 at 700 MHz

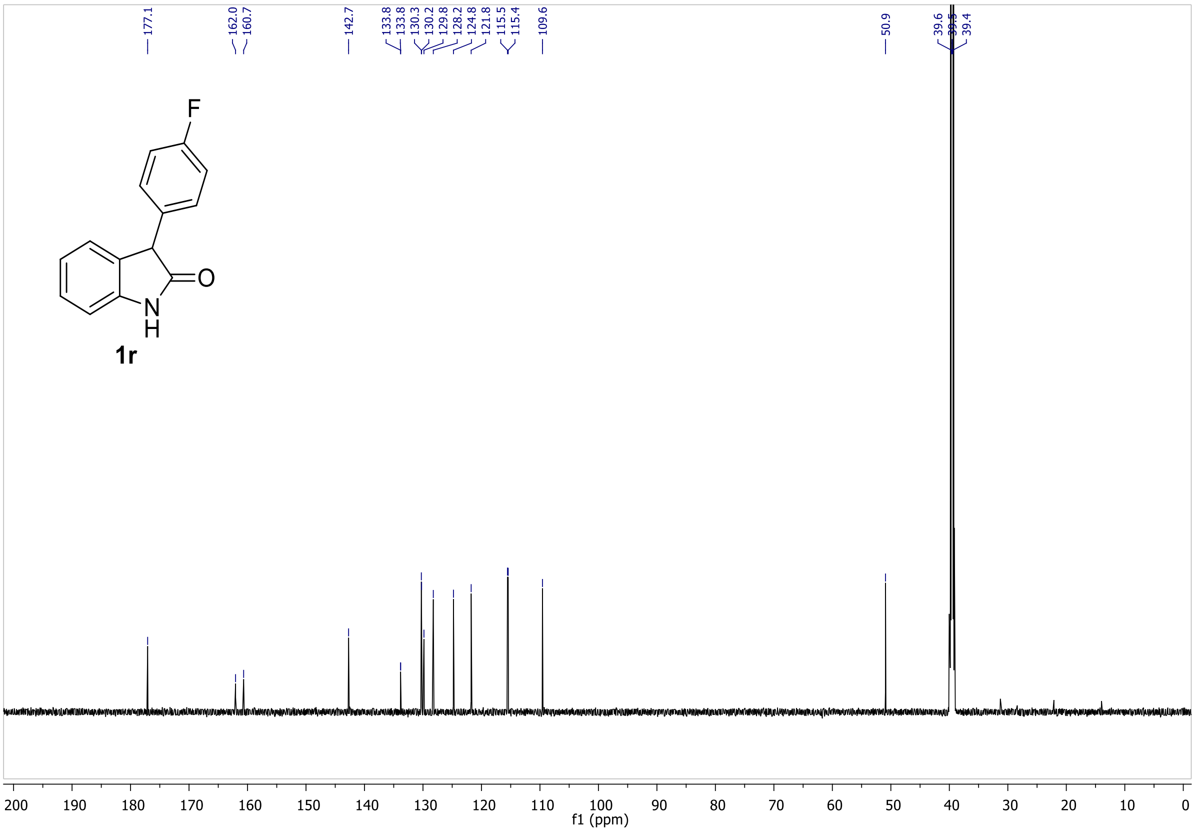

$^{13}C\{^{1}H\}$ NMR in DMSO-d_6 at 176 MHz

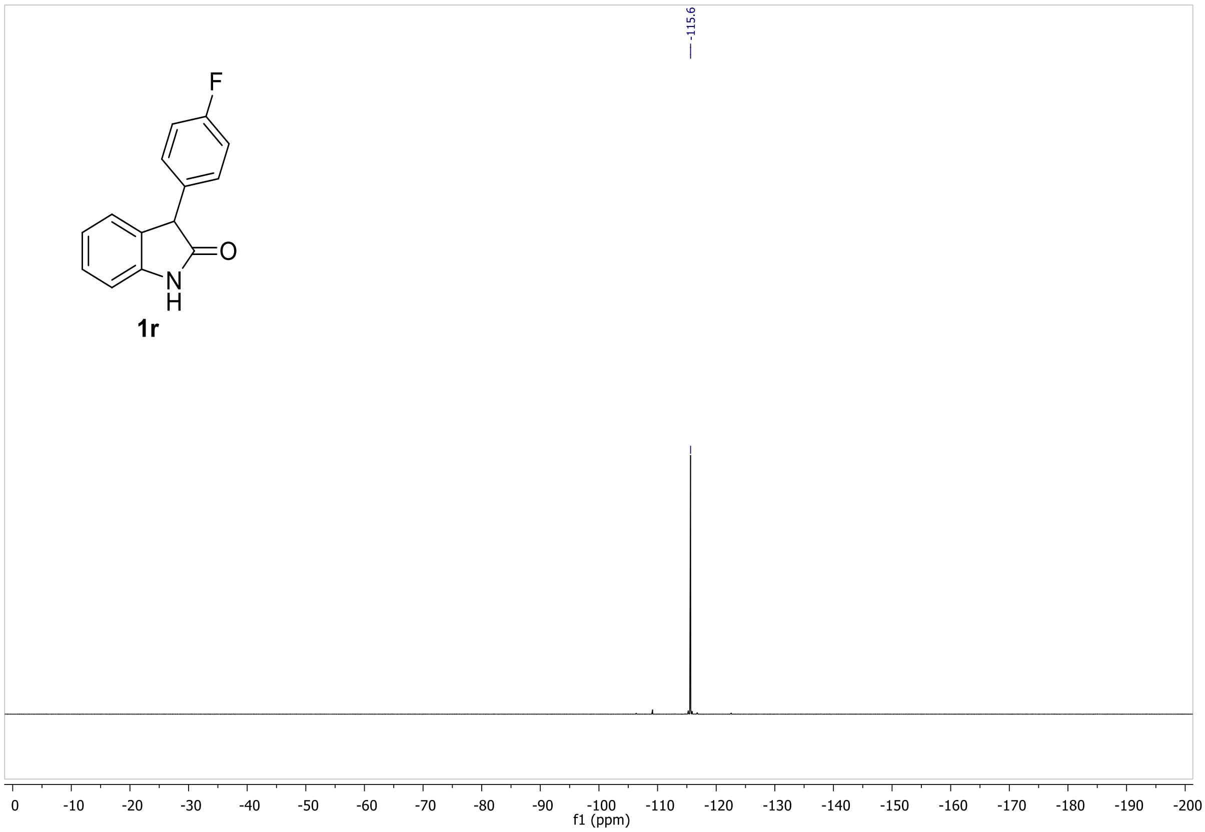

^{19}F NMR in DMSO-d_6 at 659 MHz

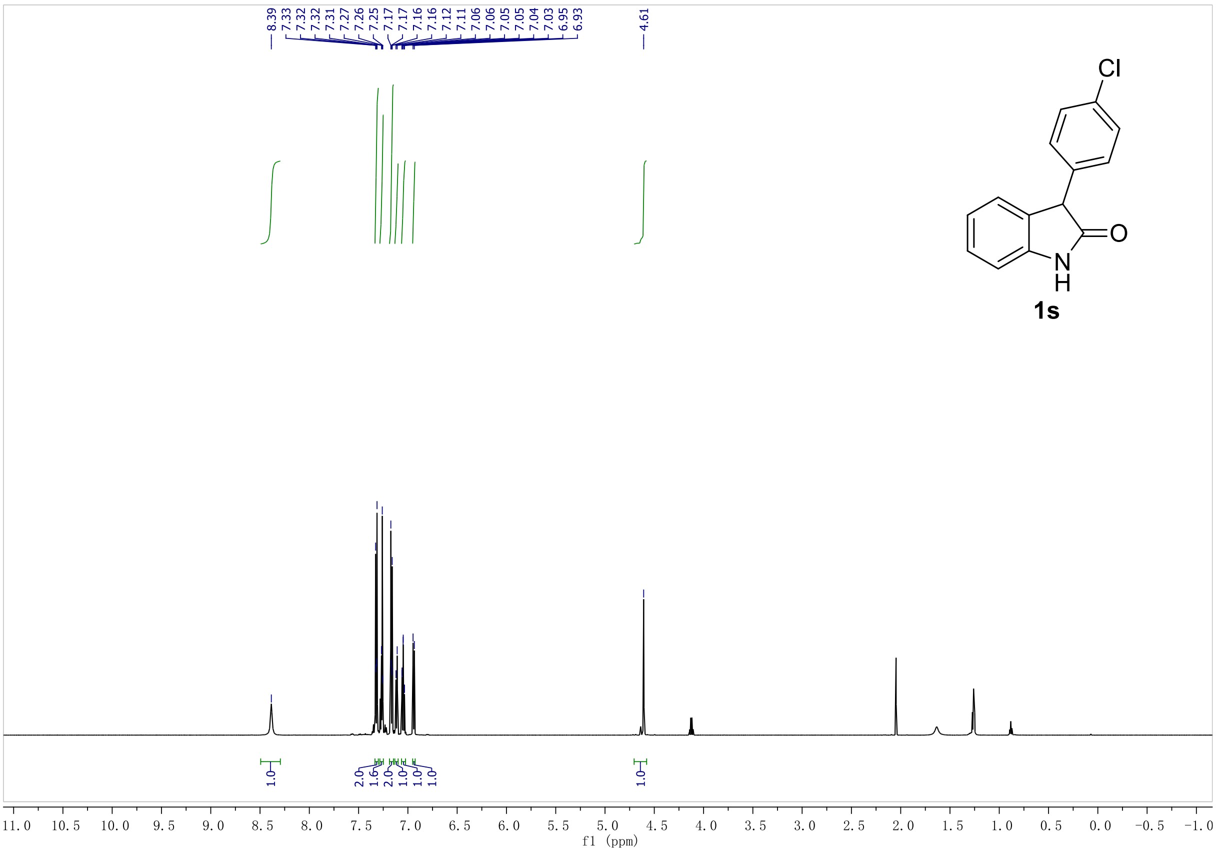

^{1}H NMR in $CDCl_3$ at 600 MHz

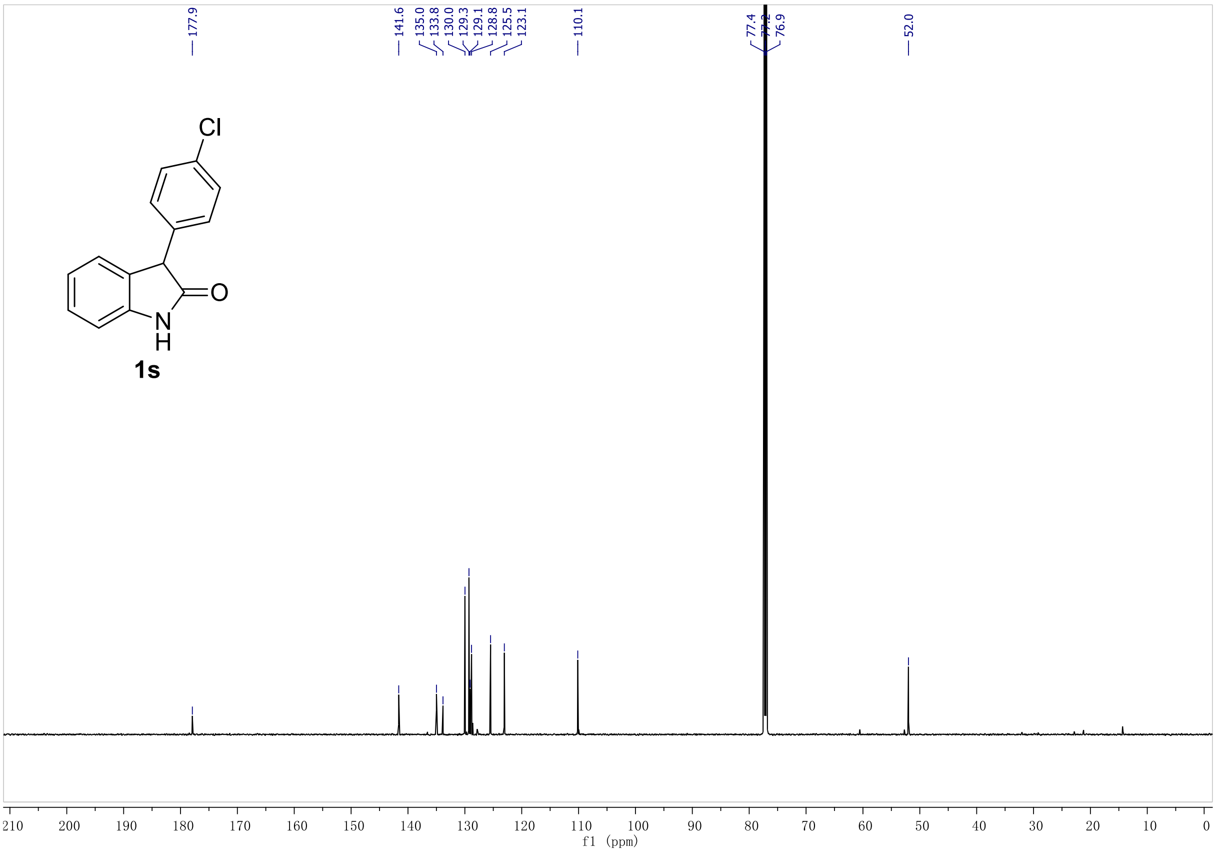

$^{13}C\{^{1}H\}$ NMR in $CDCl_3$ at 150 MHz

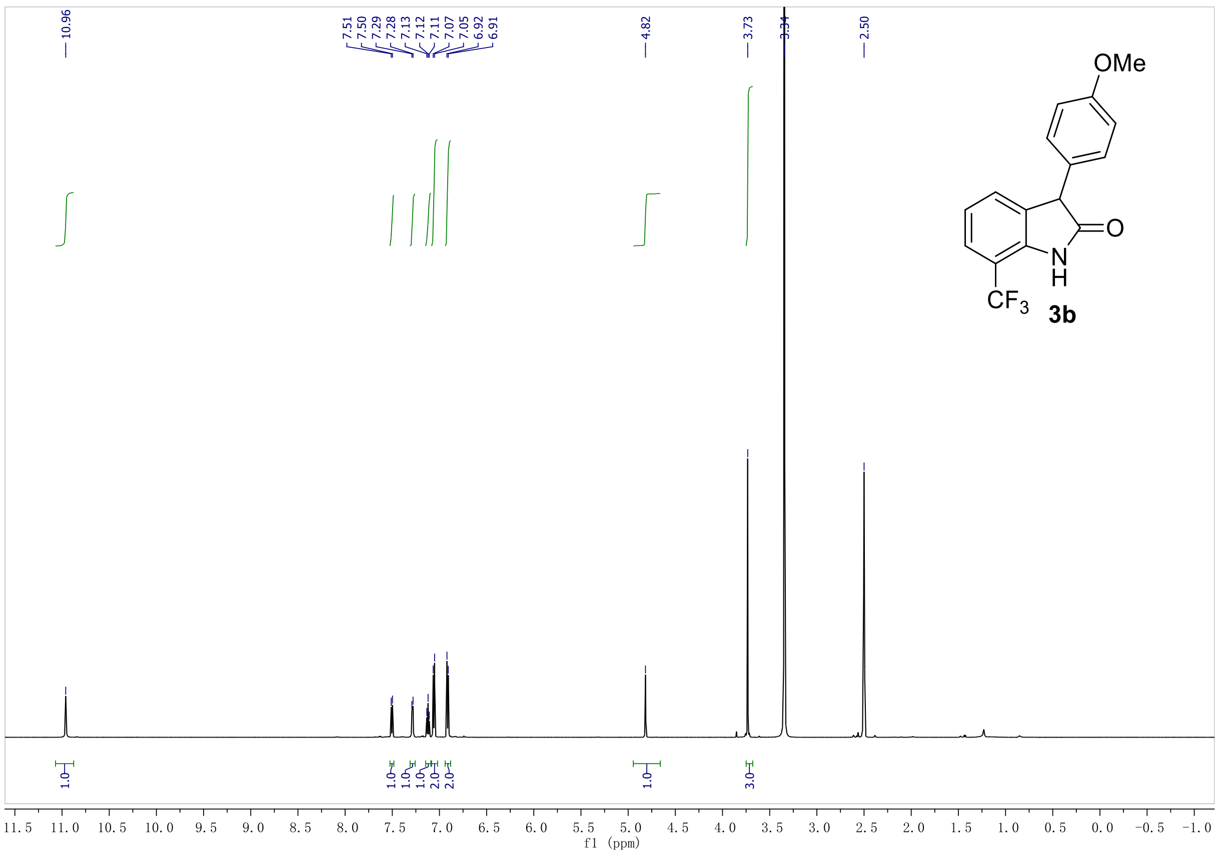

^{1}H NMR in DMSO-d_6 at 600 MHz

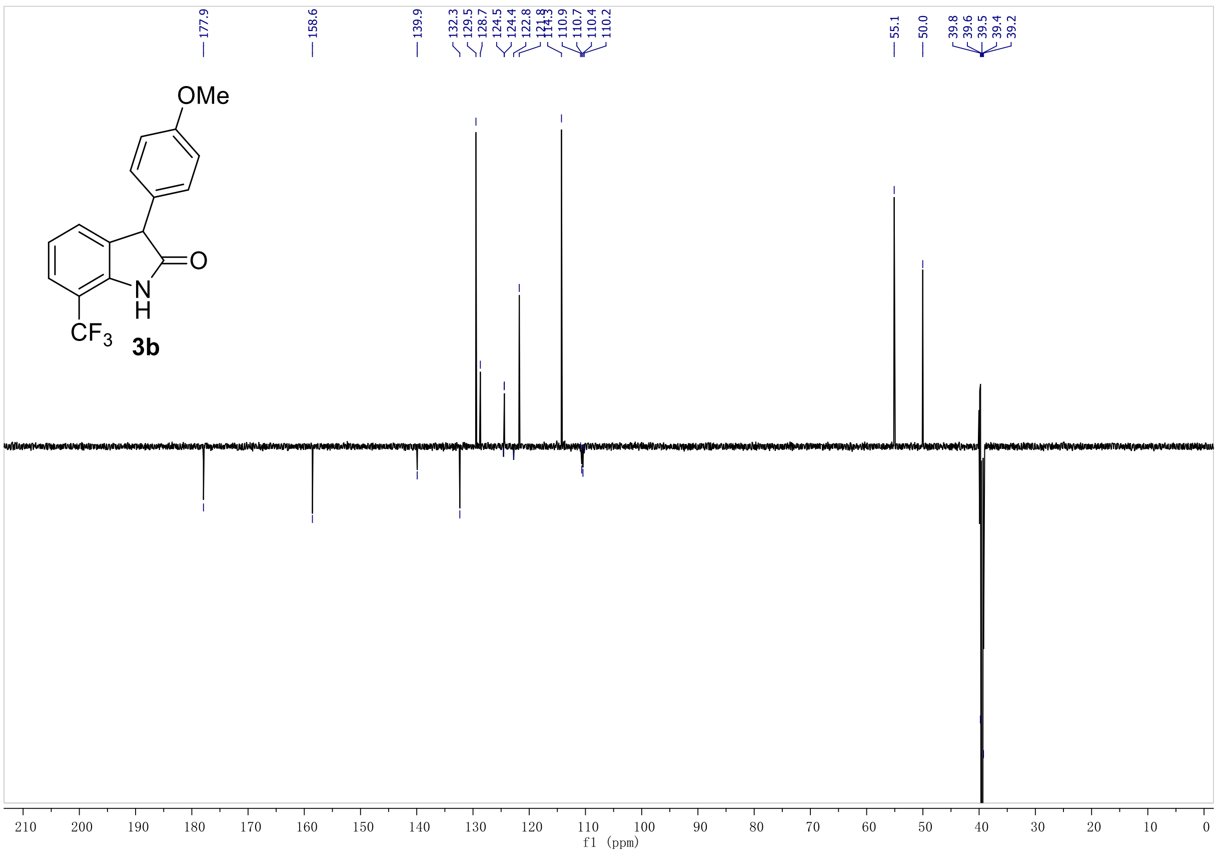

^{13}C{^{1}H} NMR in DMSO-d_6 at 150 MHz

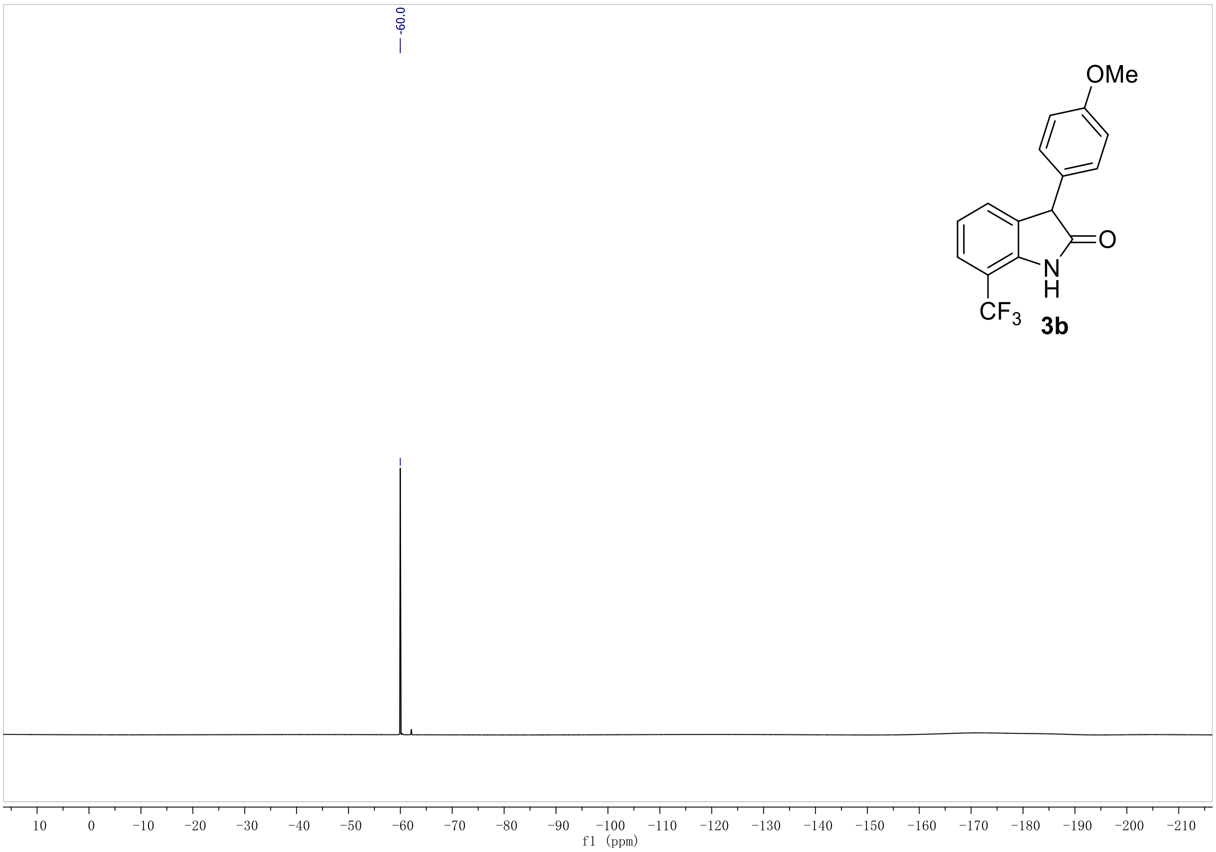

^{19}F NMR in DMSO-d_6 at 565 MHz

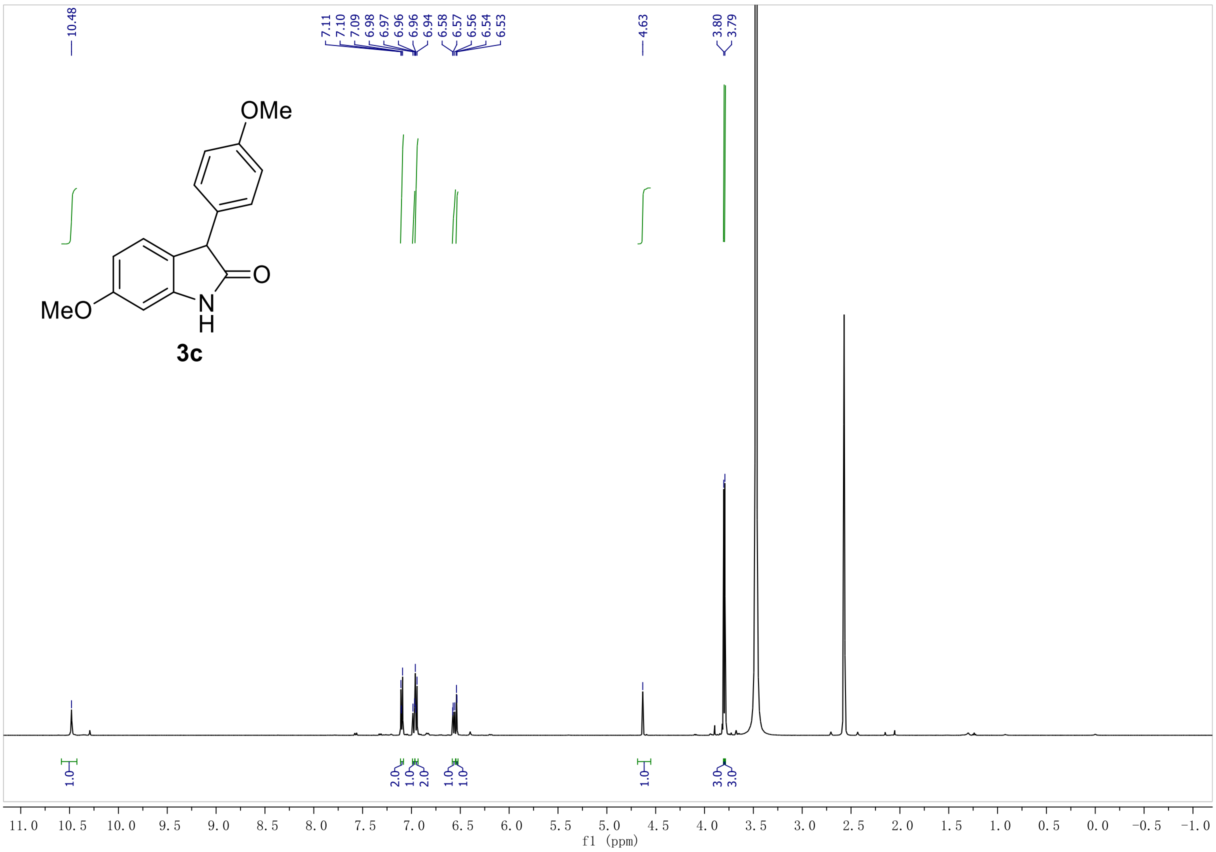

^{1}H NMR in DMSO-d_6 at 500 MHz

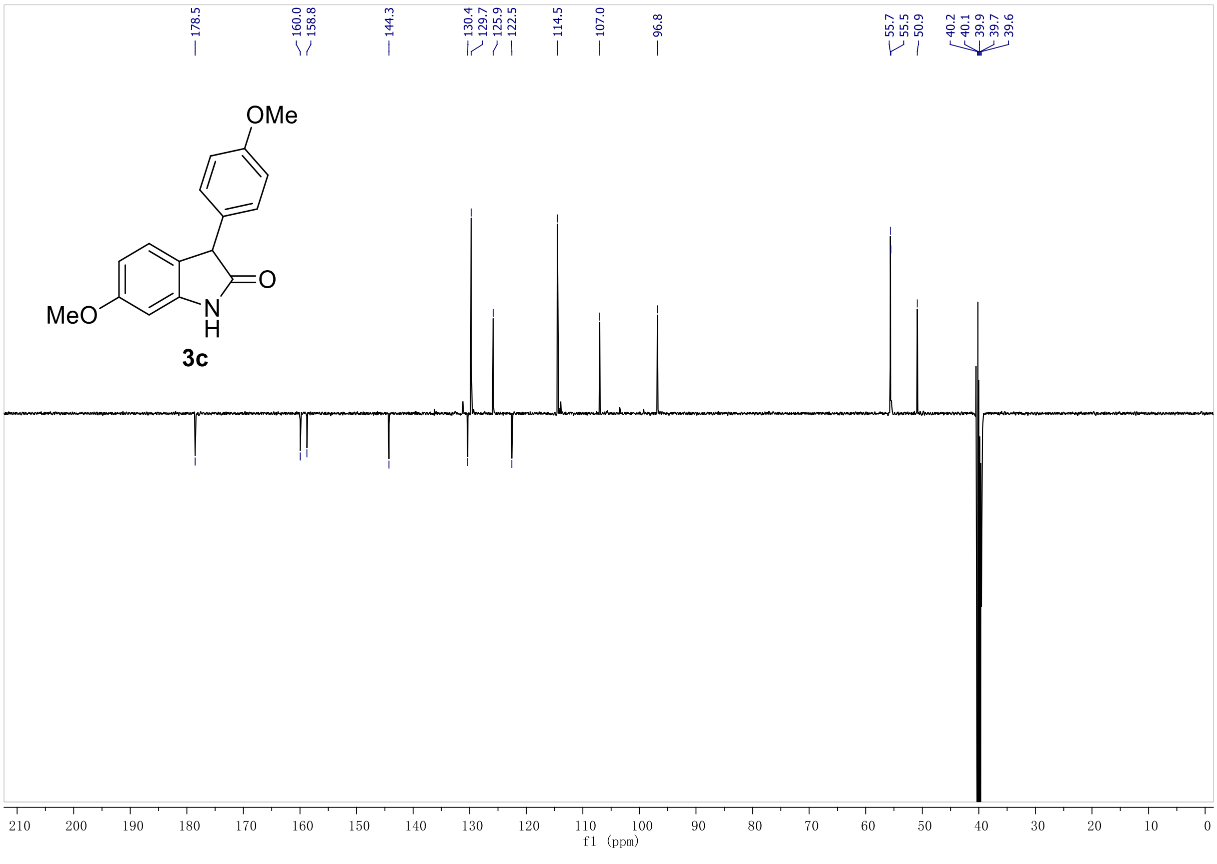

^{13}C{^{1}H} NMR in DMSO-d_6 at 126 MHz

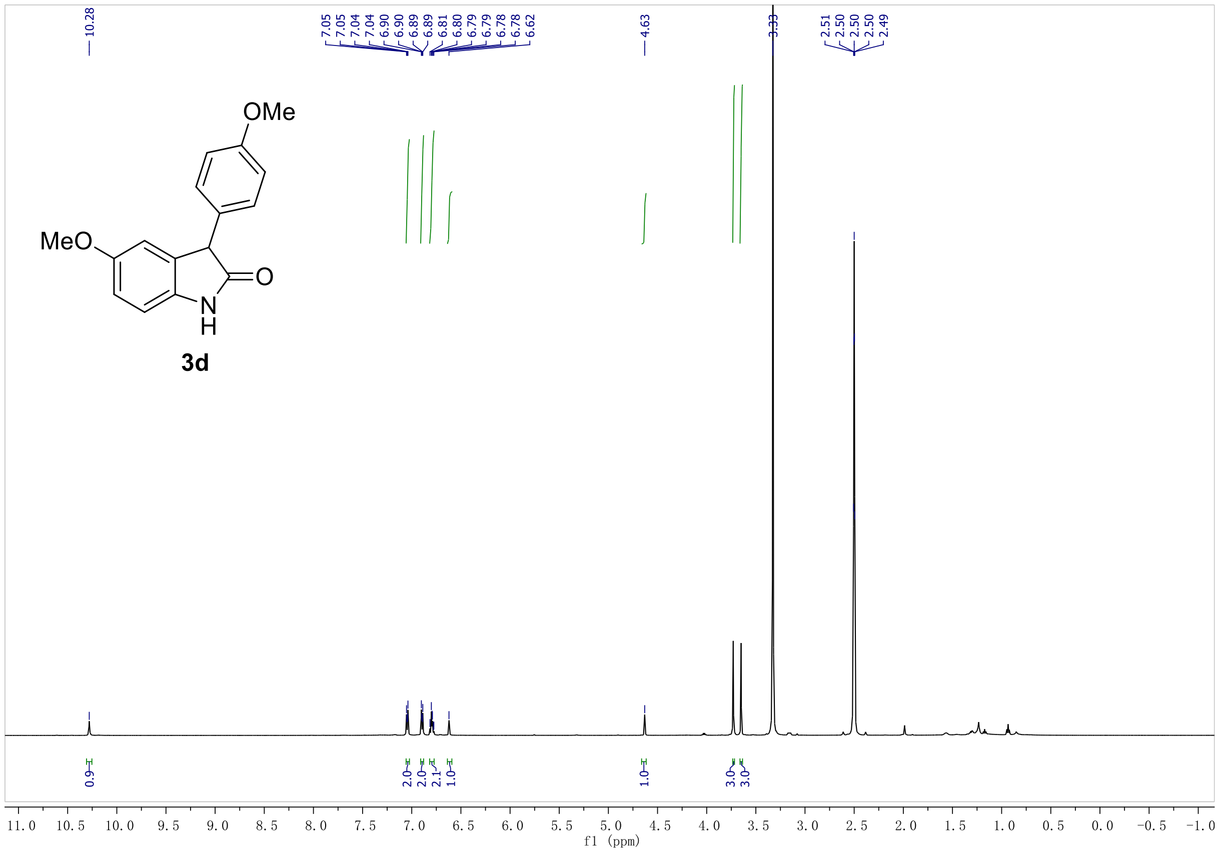

^{1}H NMR in DMSO-d_6 at 600 MHz

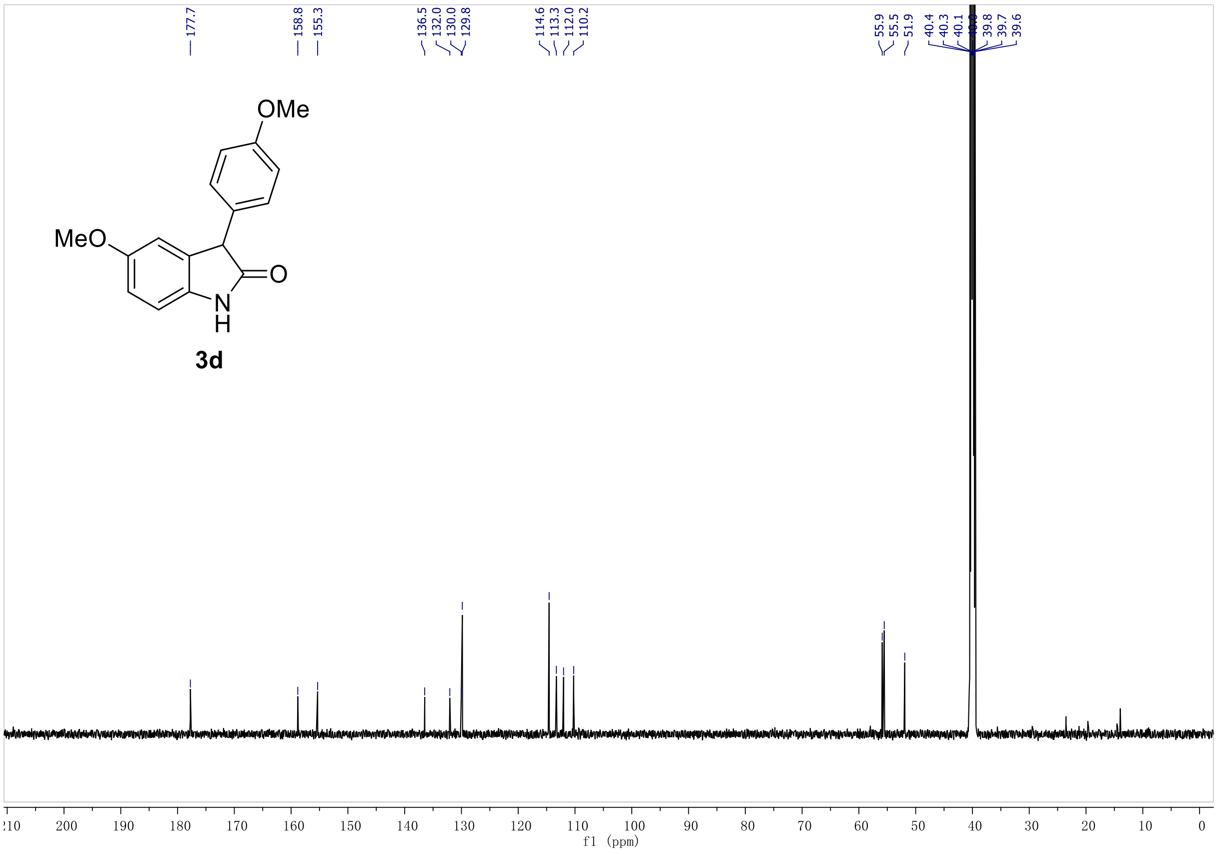

^{13}C{^{1}H} NMR in DMSO-d_6 at 150 MHz

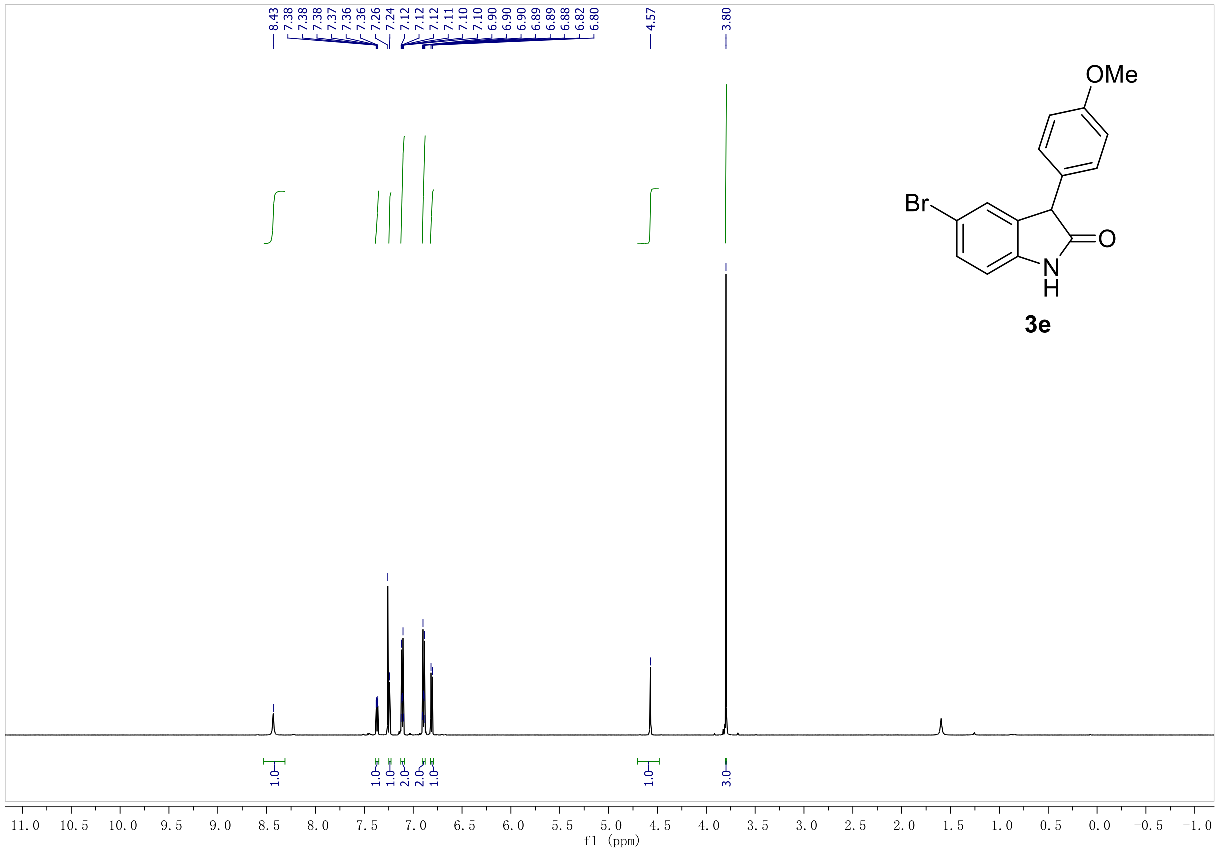

^{1}H NMR in $CDCl_3$ at 600 MHz

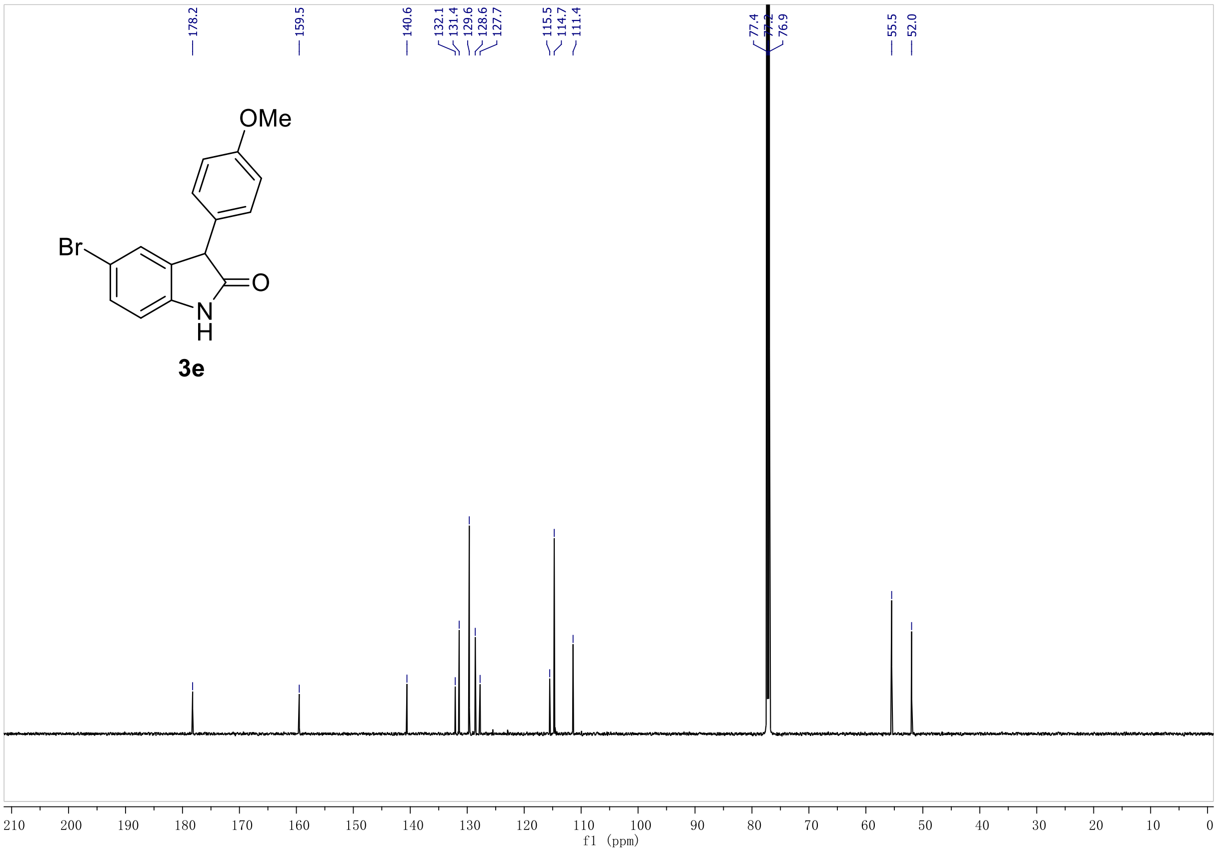

^{13}C{^{1}H} NMR in $CDCl_3$ at 150 MHz

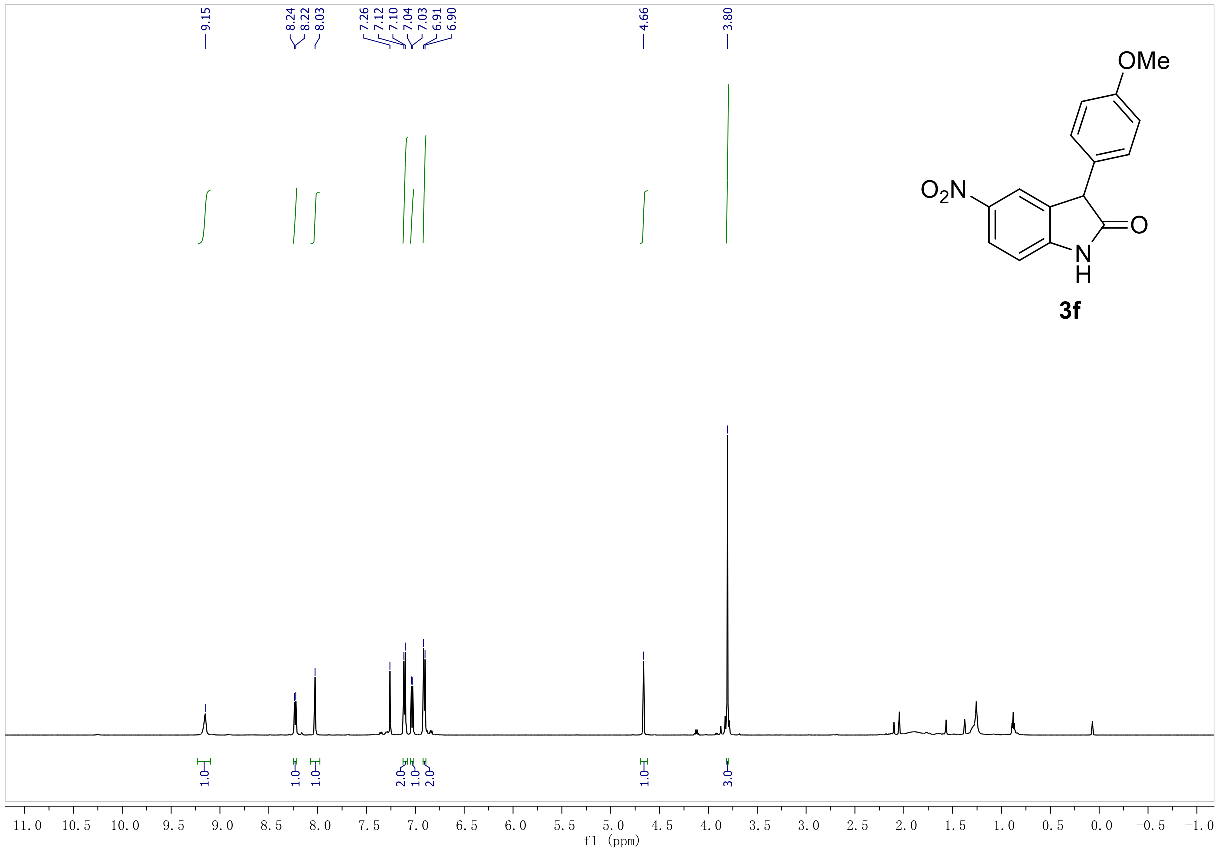

^{1}H NMR in $CDCl_3$ at 600 MHz

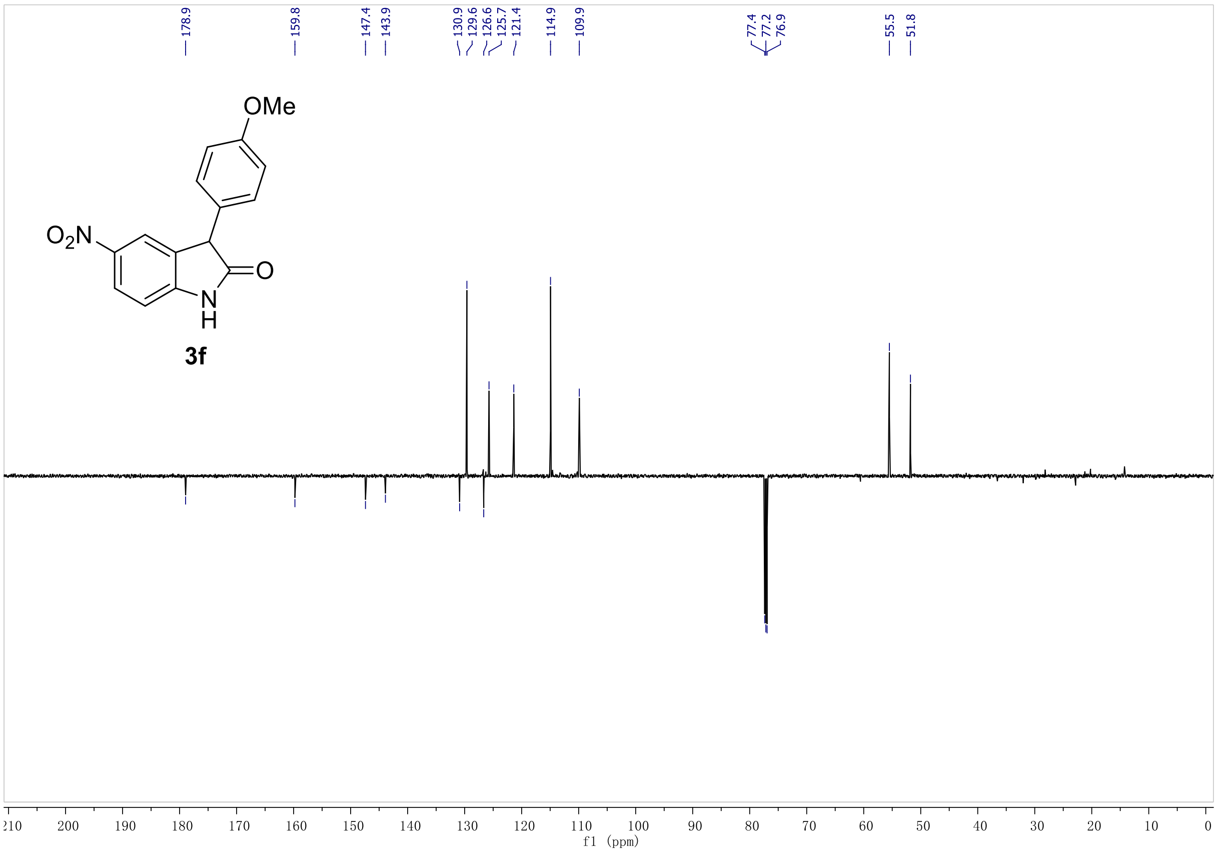

$^{13}C\{^{1}H\}$ NMR in $CDCl_3$ at 150 MHz

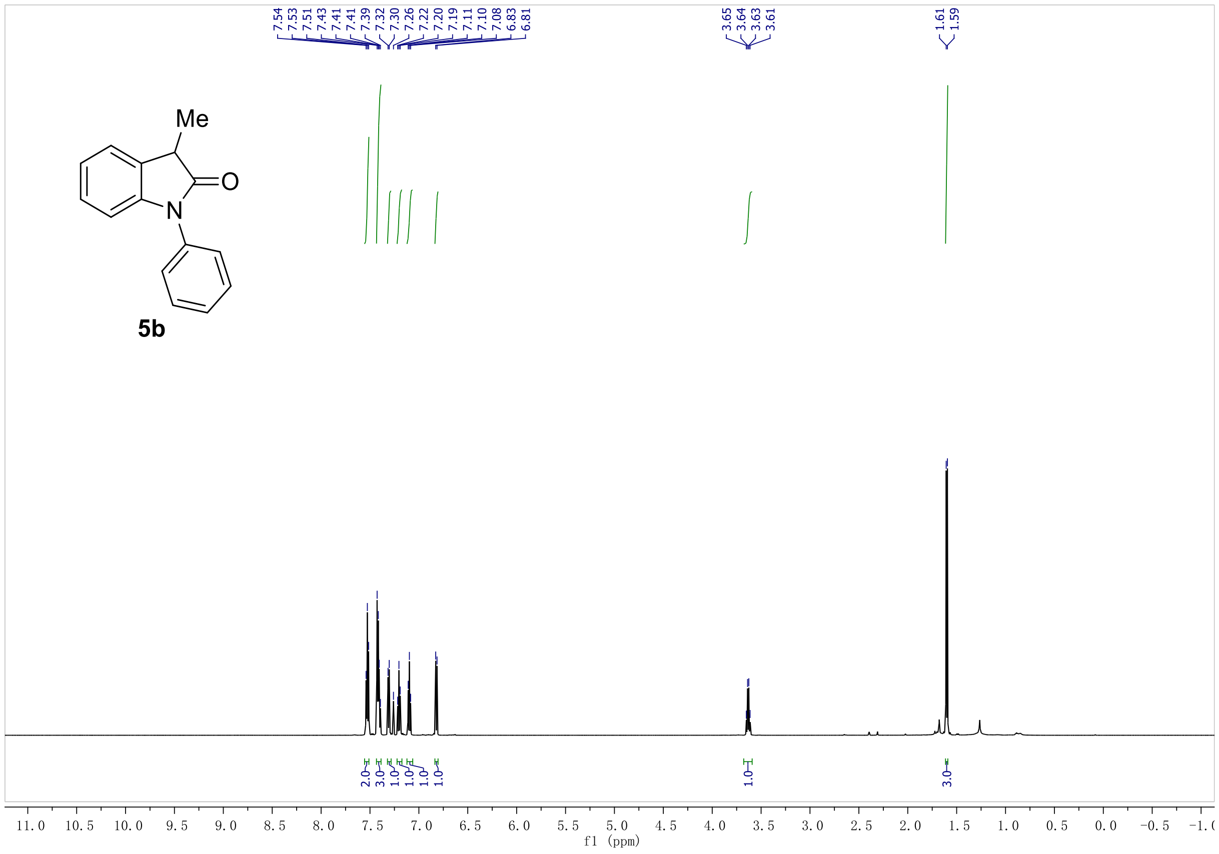

^{1}H NMR in $CDCl_3$ at 600 MHz

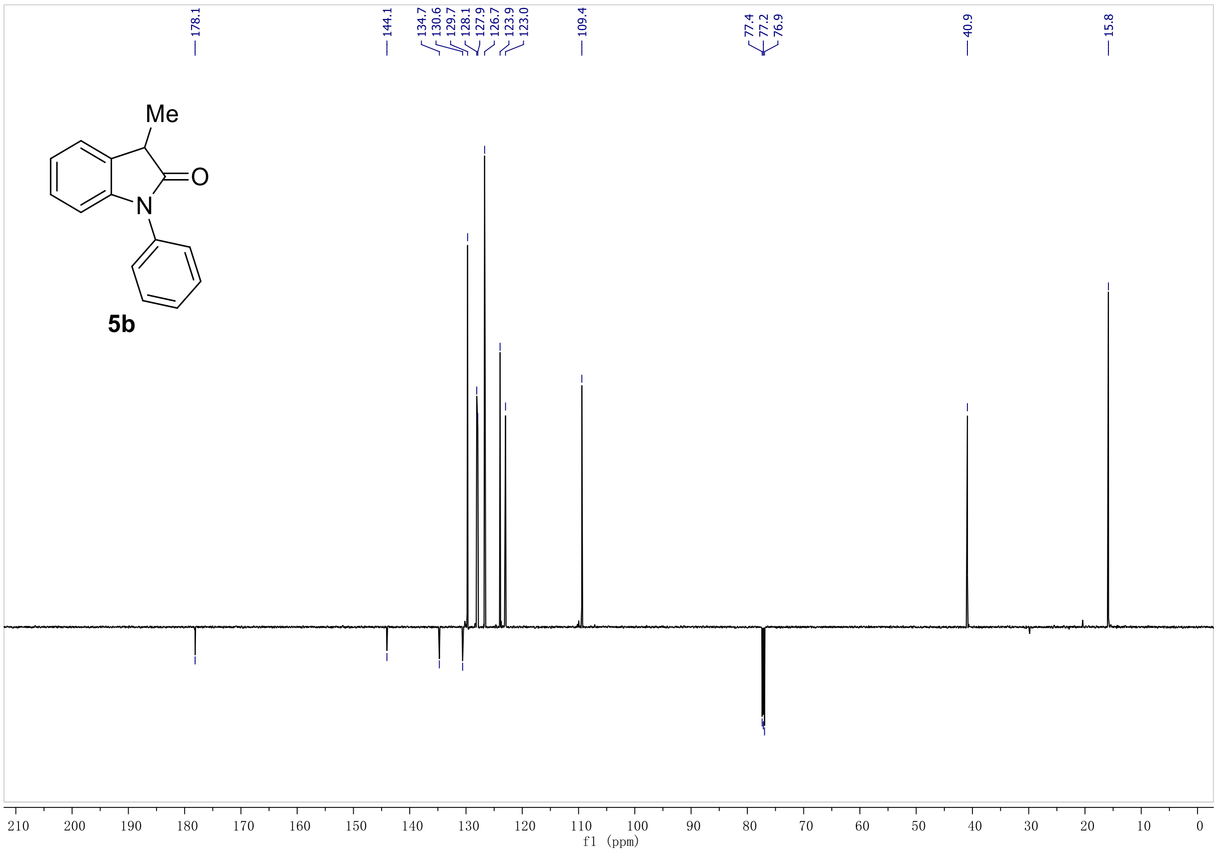

^{13}C{^{1}H} NMR in $CDCl_3$ at 150 MHz

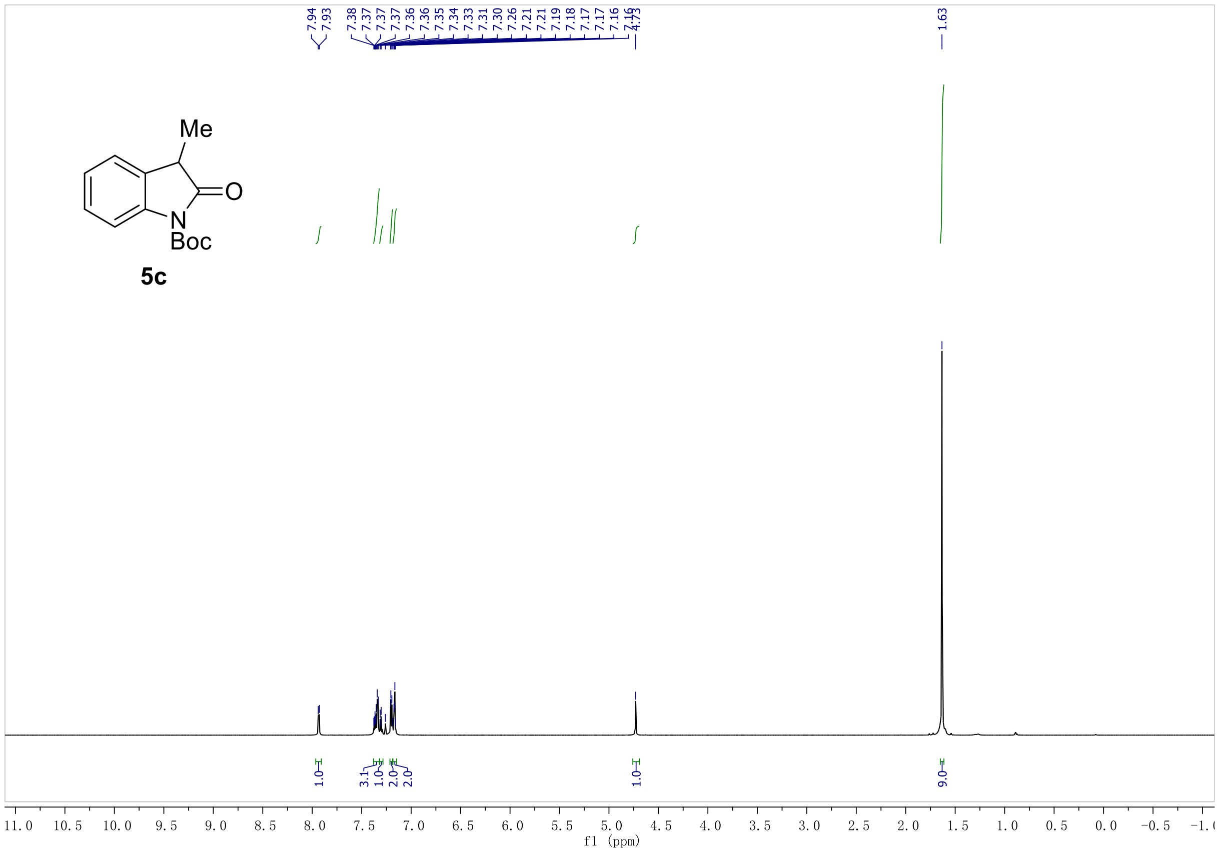

^{1}H NMR in $CDCl_3$ at 700 MHz

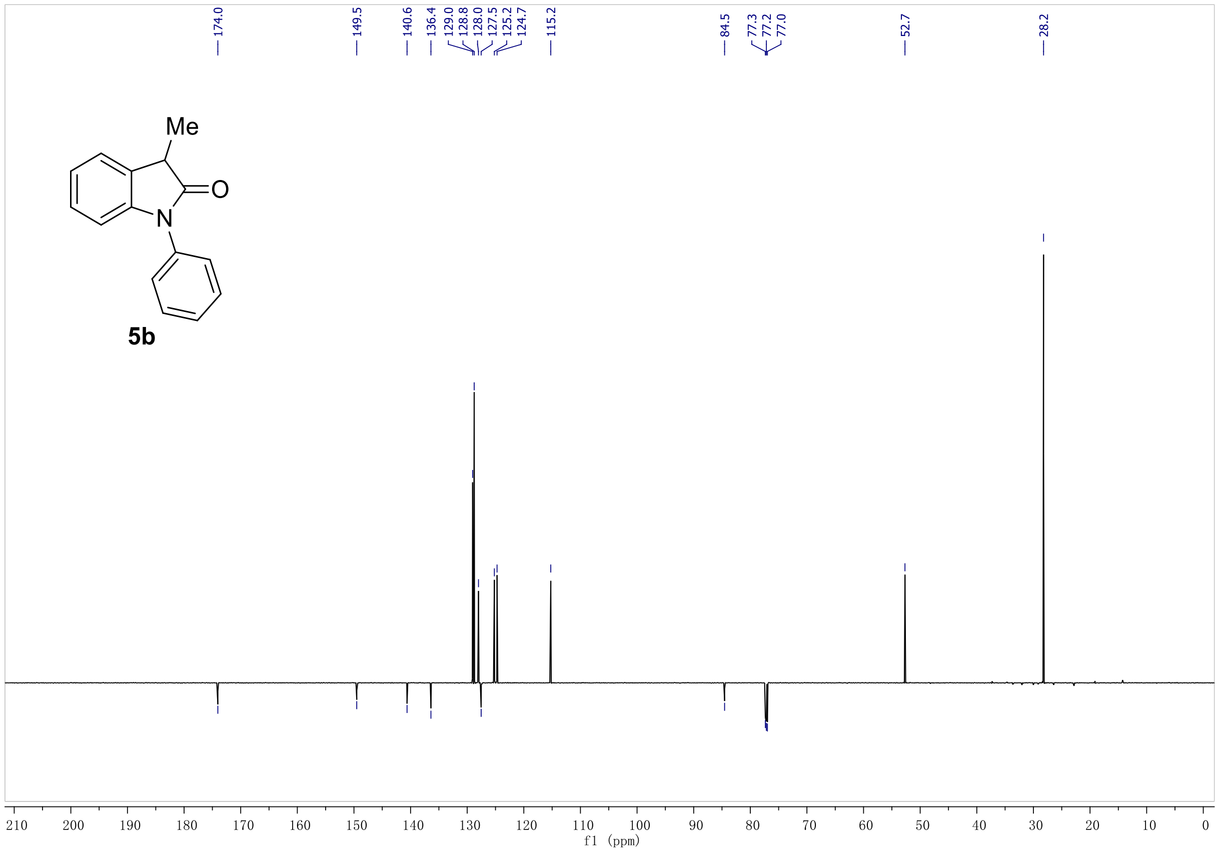

^{13}C{^{1}H} NMR in $CDCl_3$ at 176 MHz

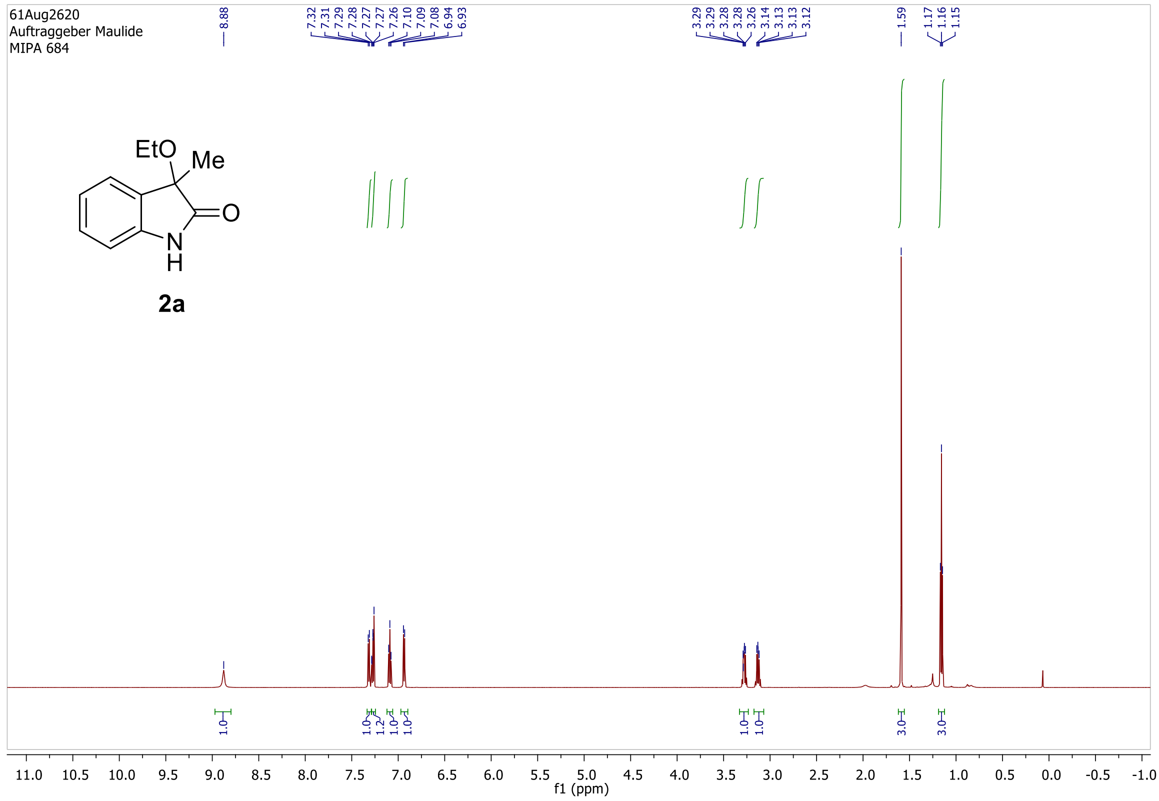

^{1}H NMR in $CDCl_3$ at 600 MHz

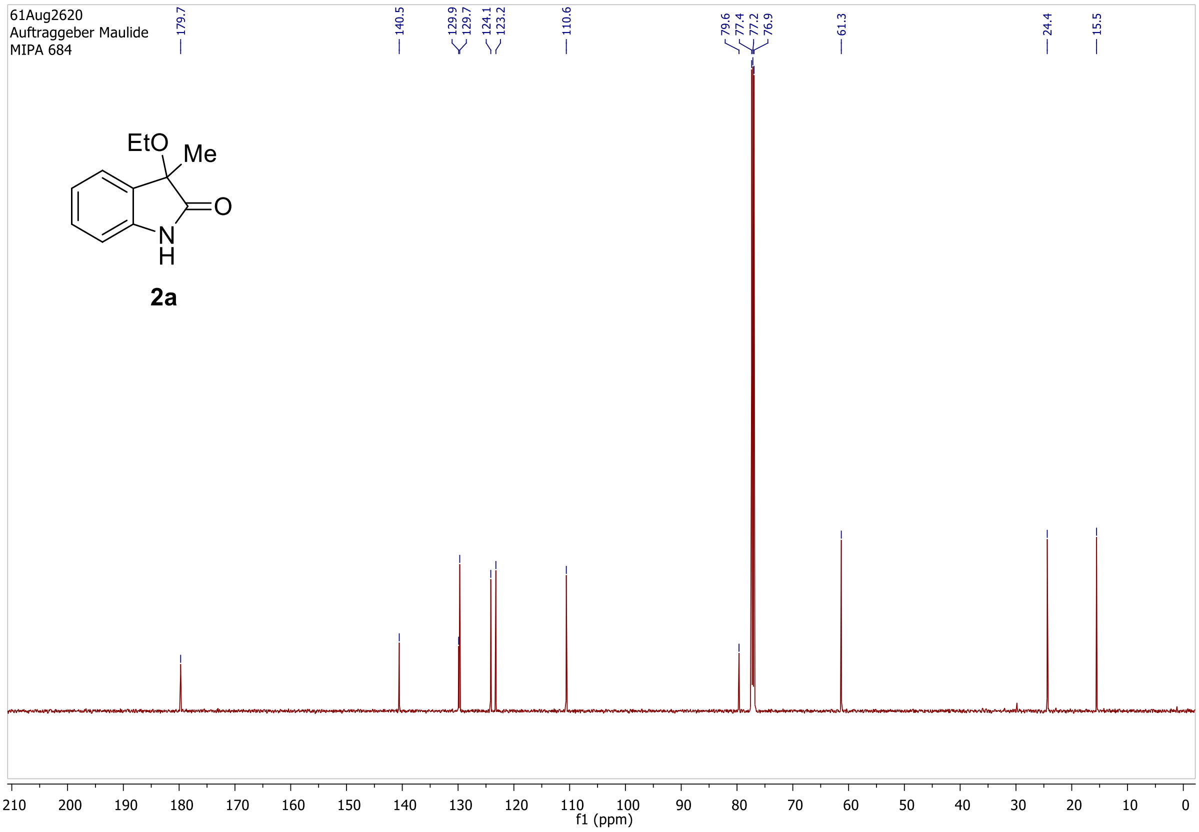

$^{13}C\{^1H\}$ NMR in $CDCl_3$ at 150 MHz

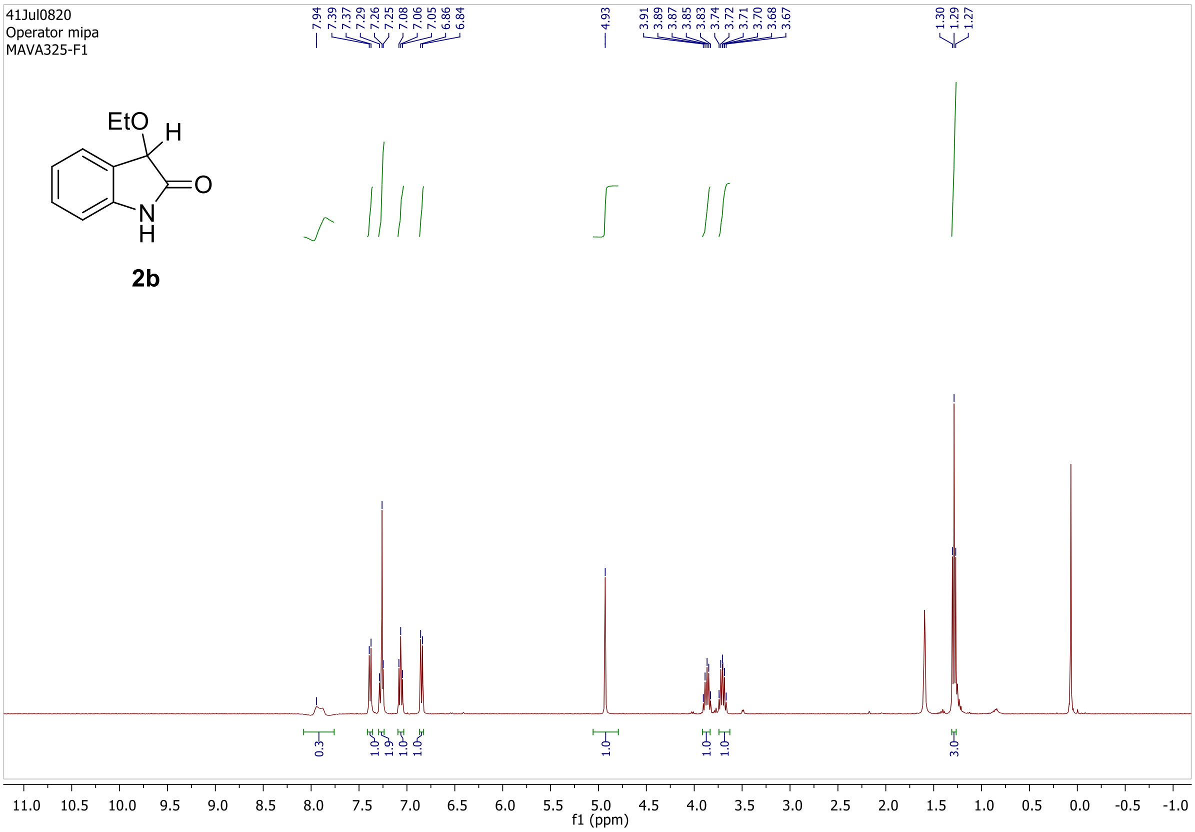

^{1}H NMR in $CDCl_3$ at 400 MHz

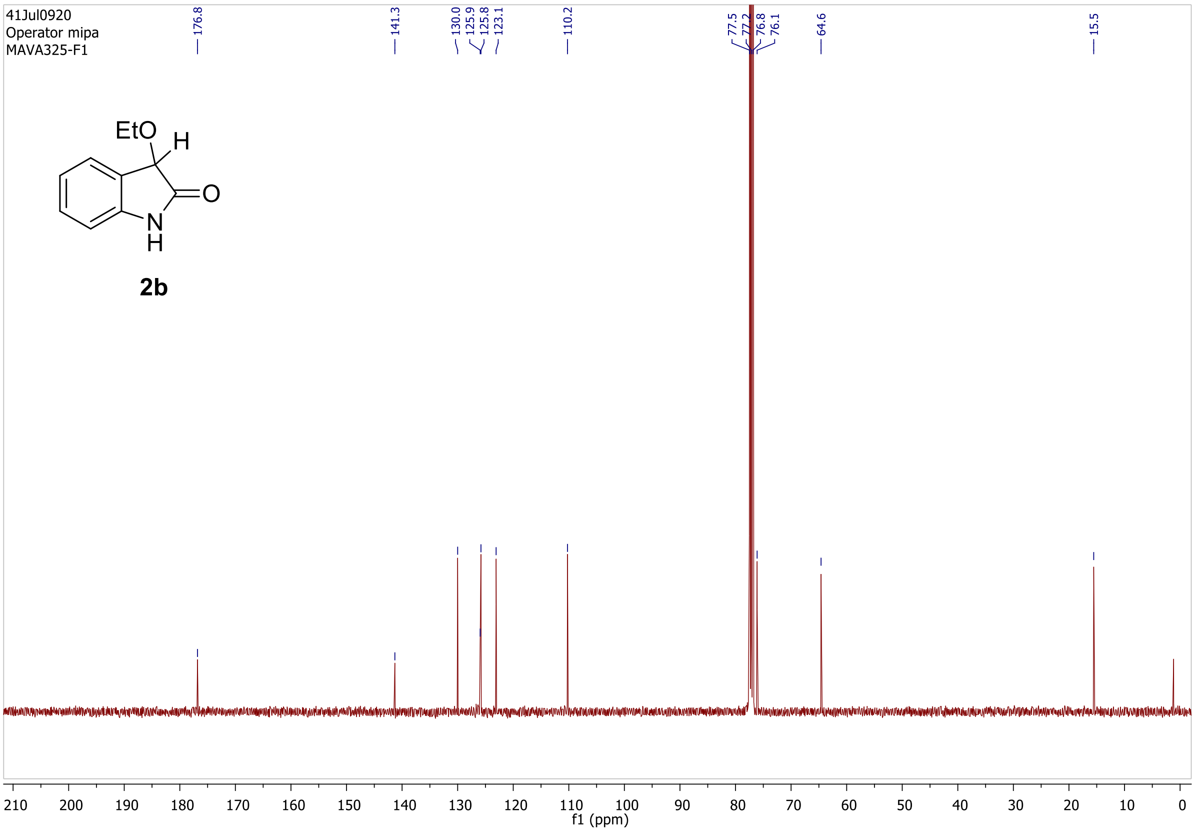

$^{13}C\{^{1}H\}$ NMR in $CDCl_3$ at 100 MHz

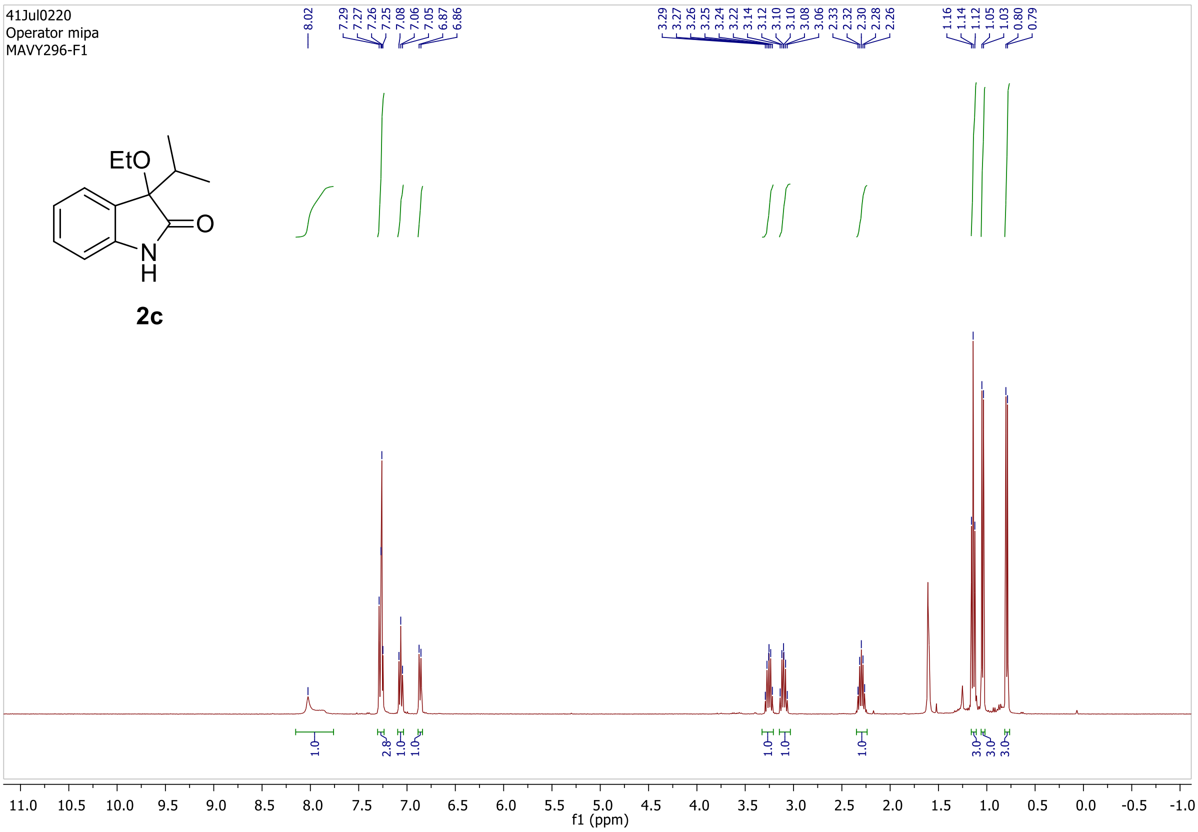

^{1}H NMR in $CDCl_3$ at 400 MHz

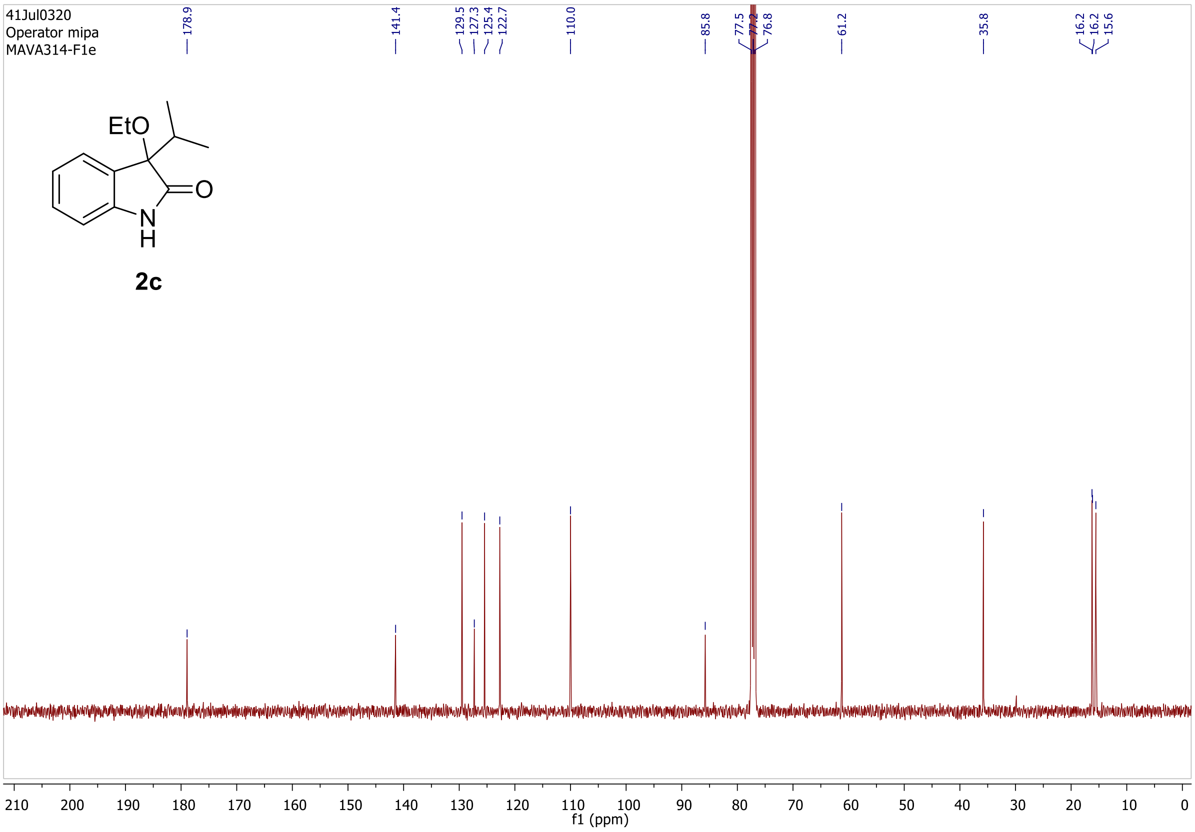

$^{13}C\{^{1}H\}$ NMR in $CDCl_3$ at 100 MHz

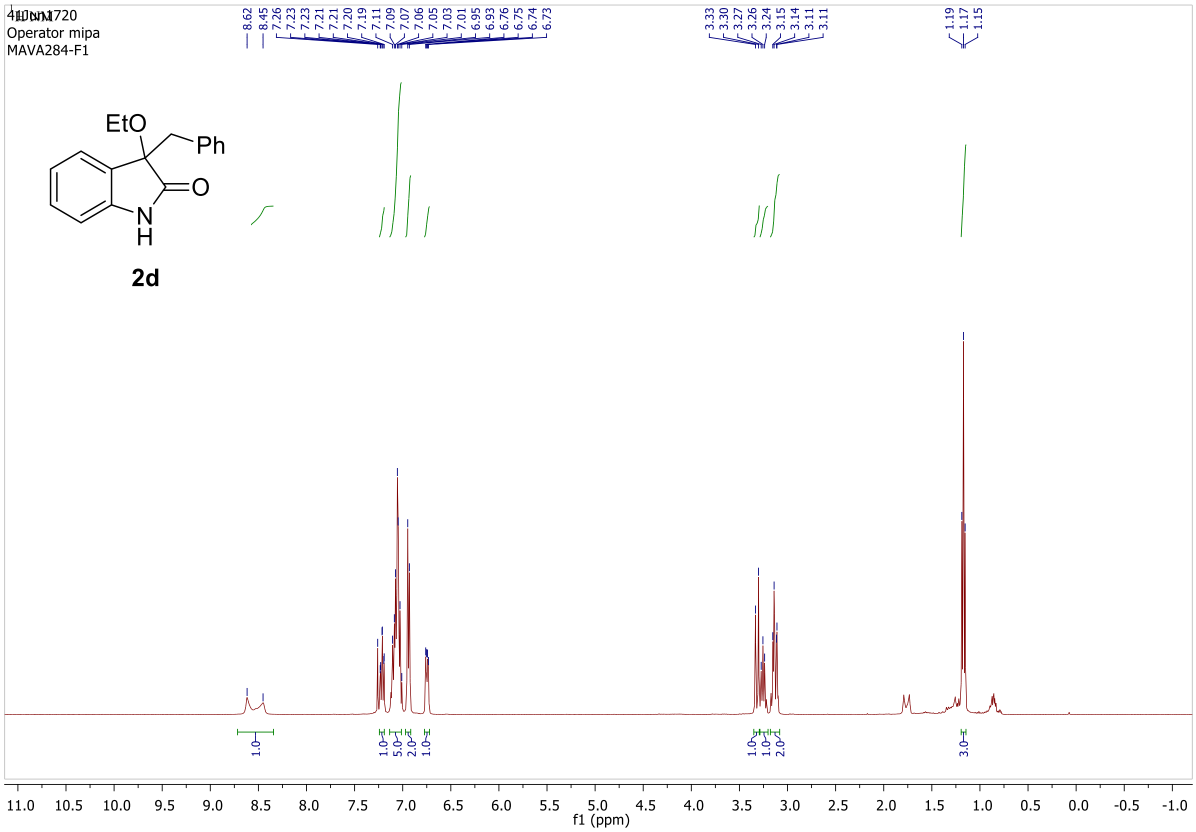

^{1}H NMR in $CDCl_3$ at 400 MHz

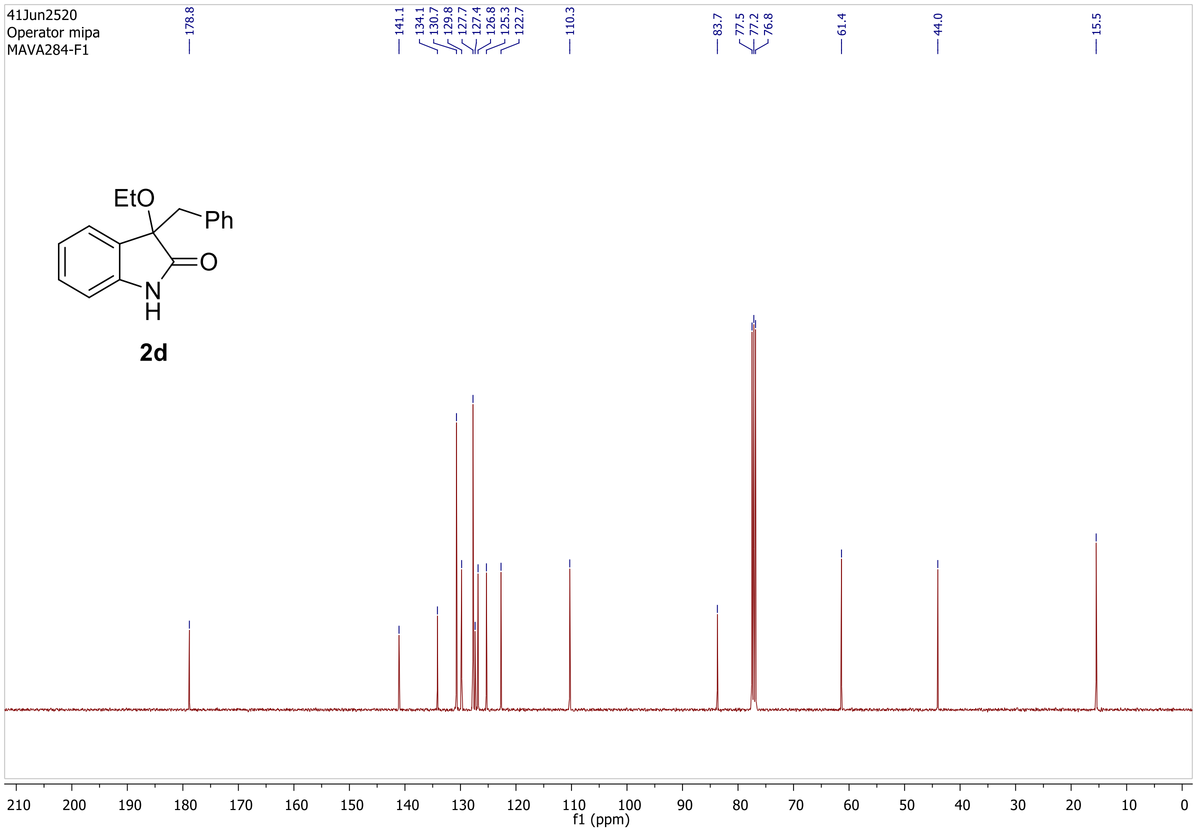

^{13}C{^{1}H} NMR in $CDCl_3$ at 100 MHz

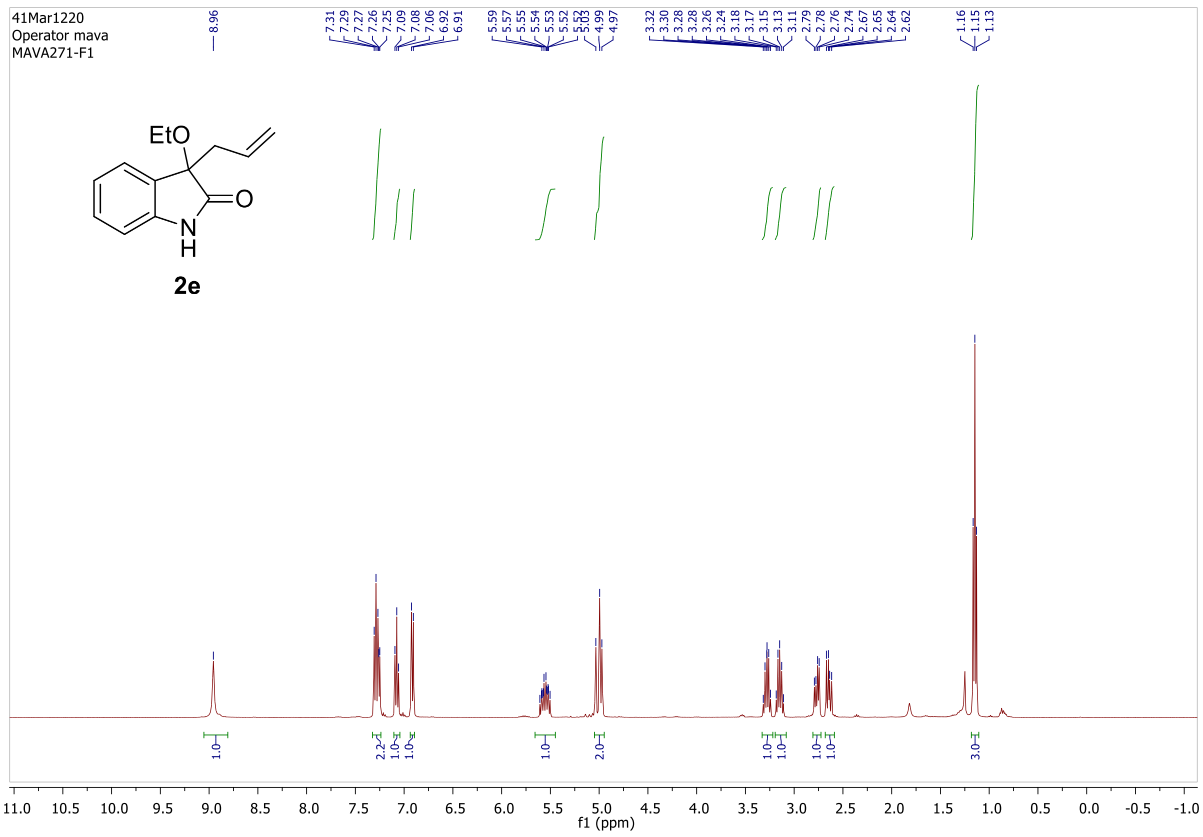

^{1}H NMR in $CDCl_3$ at 400 MHz

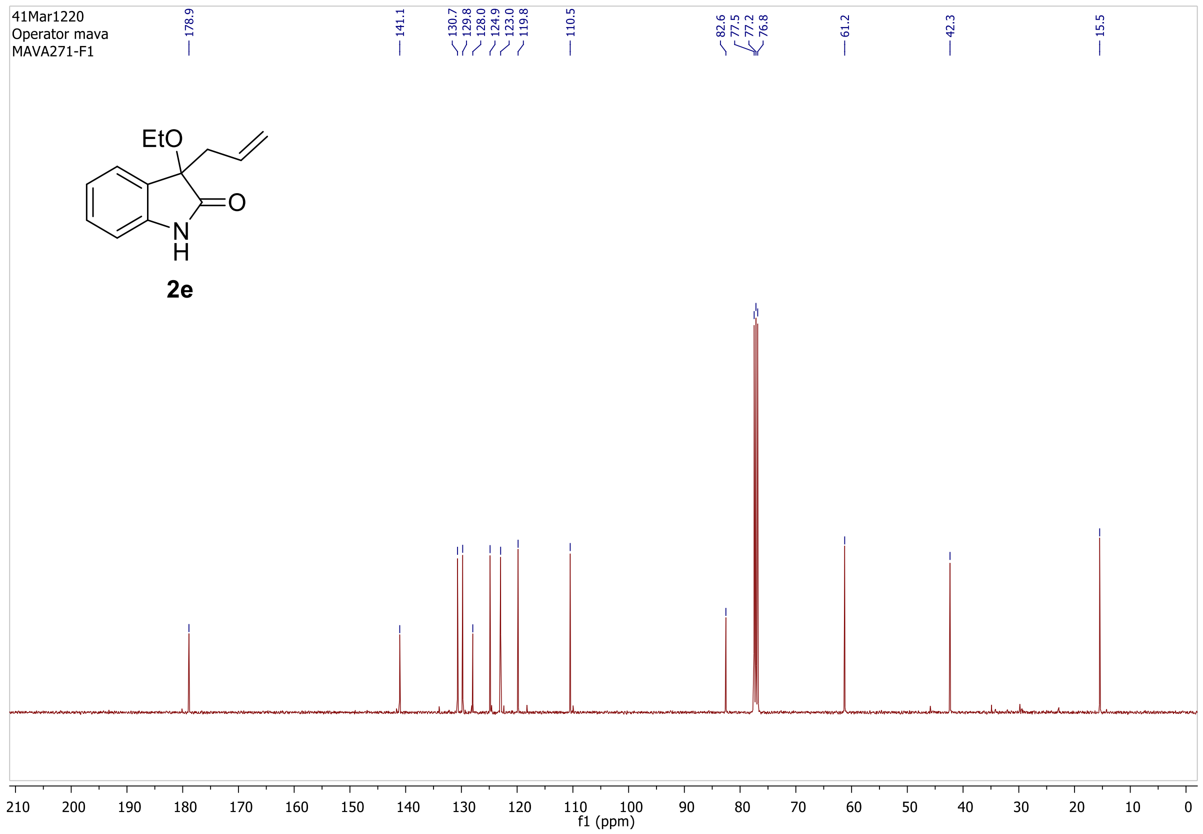

$^{13}C\{^1H\}$ NMR in $CDCl_3$ at 100 MHz

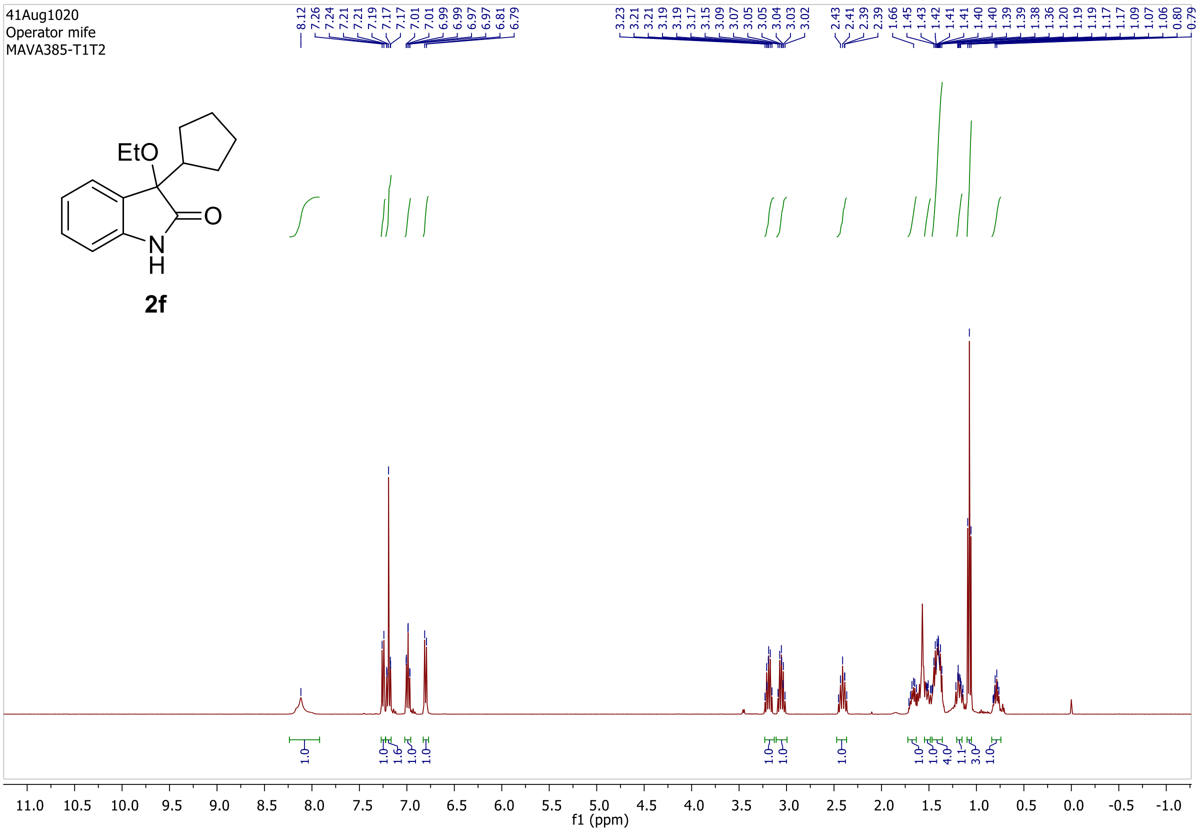

^{1}H NMR in $CDCl_3$ at 400 MHz

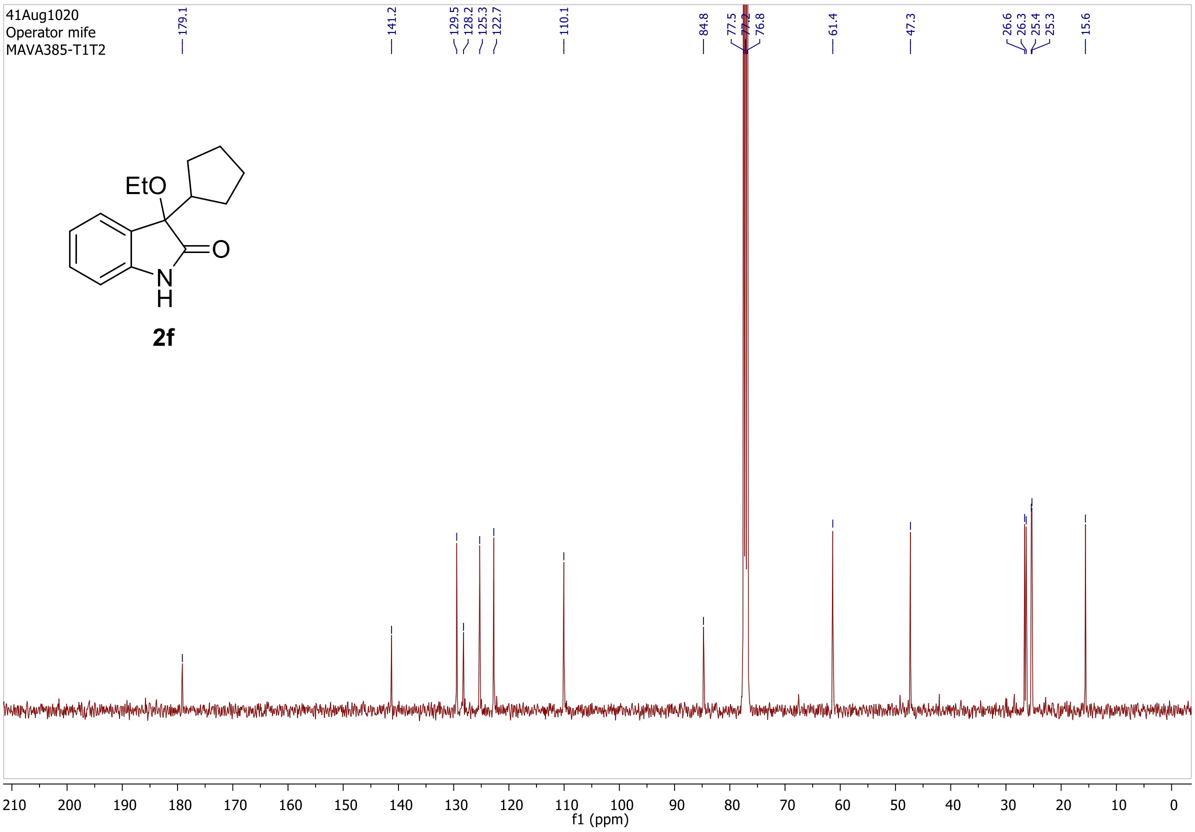

$^{13}C\{^{1}H\}$ NMR in $CDCl_3$ at 100 MHz

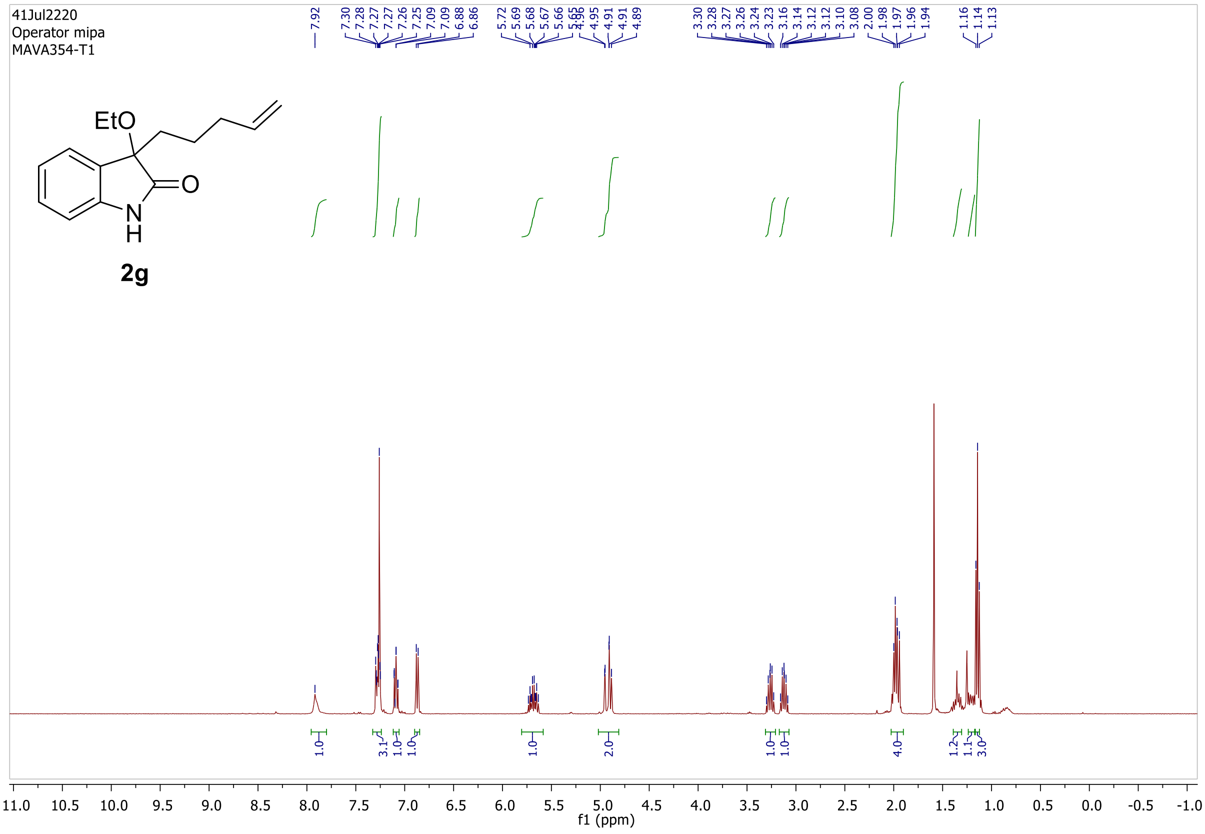

^{1}H NMR in $CDCl_3$ at 400 MHz

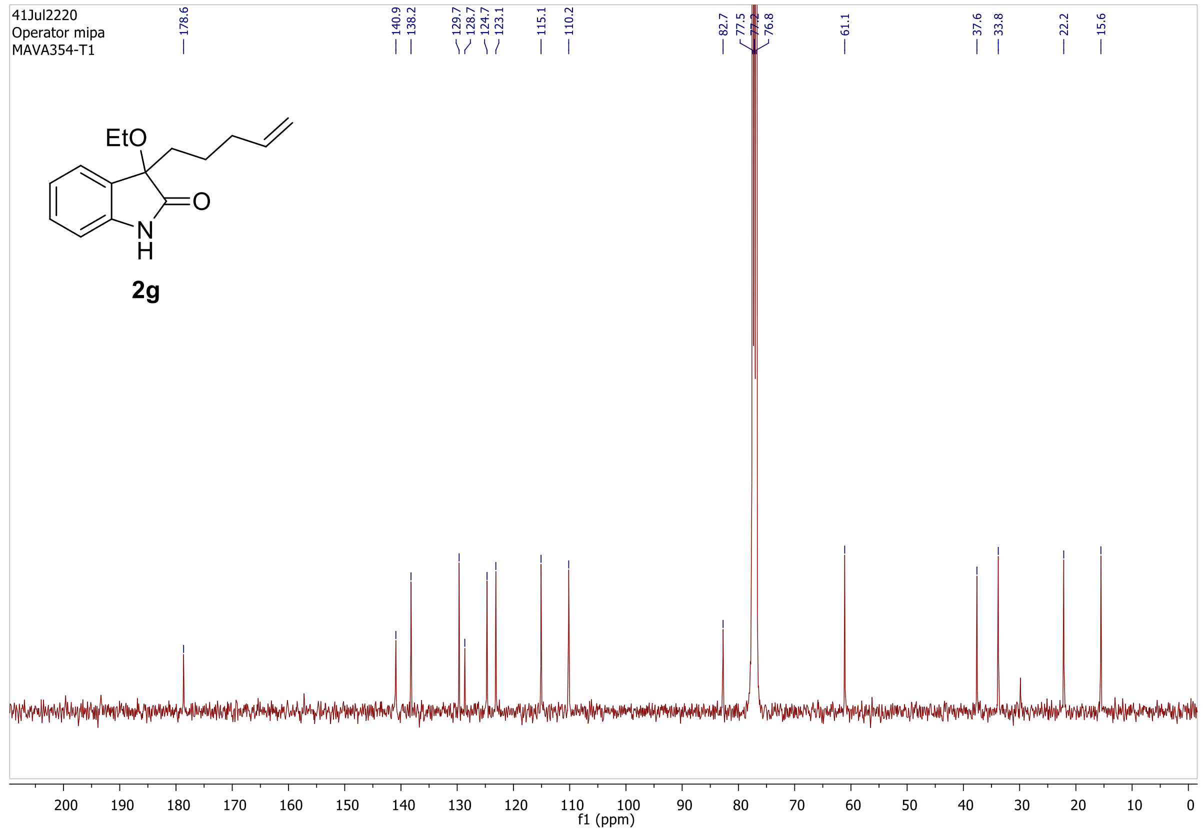

$^{13}C\{^{1}H\}$ NMR in $CDCl_3$ at 100 MHz

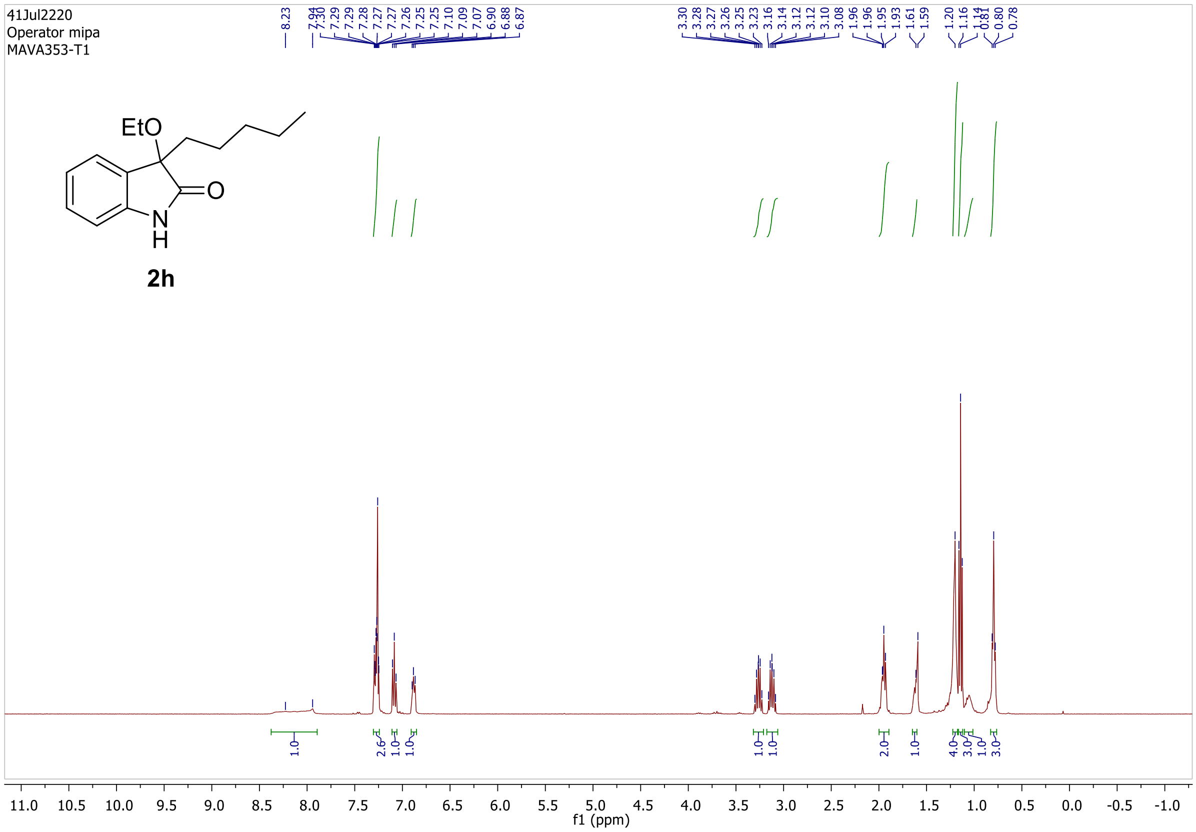

^1H NMR in $CDCl_3$ at 400 MHz

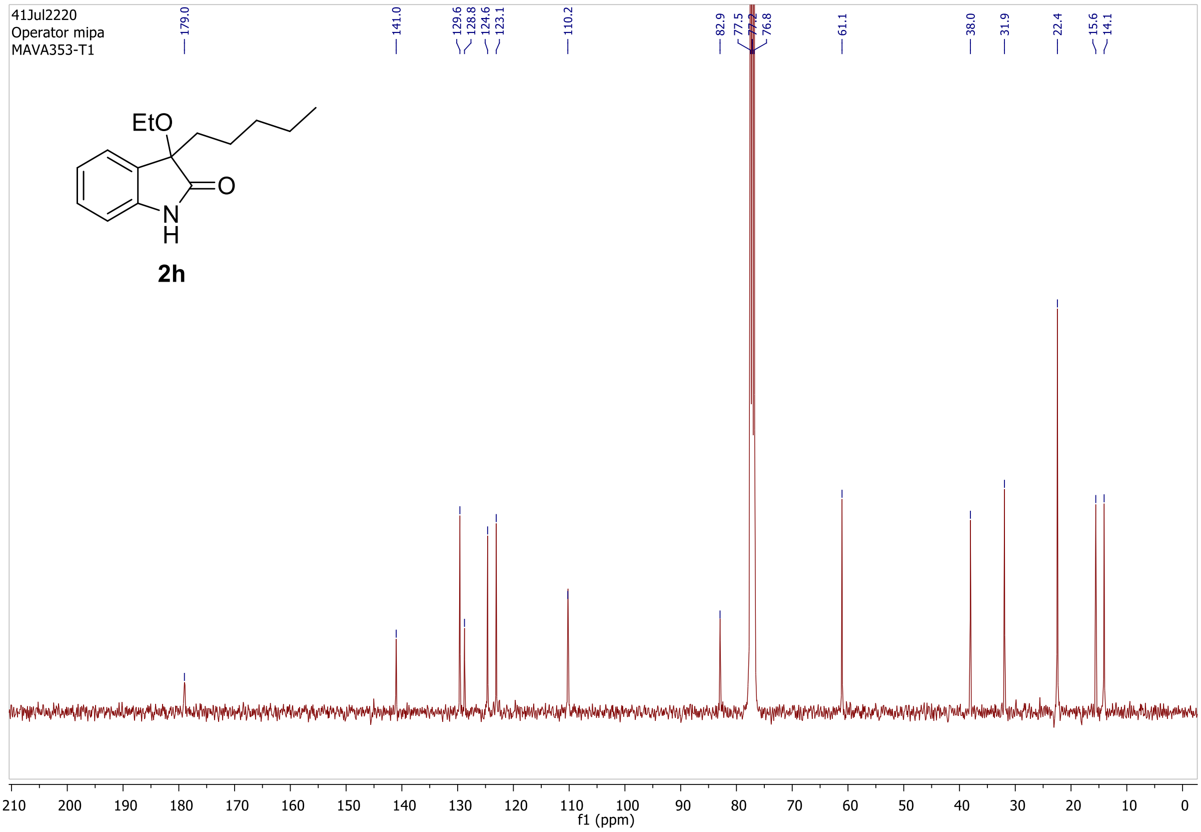

$^{13}C\{^1H\}$ NMR in $CDCl_3$ at 100 MHz

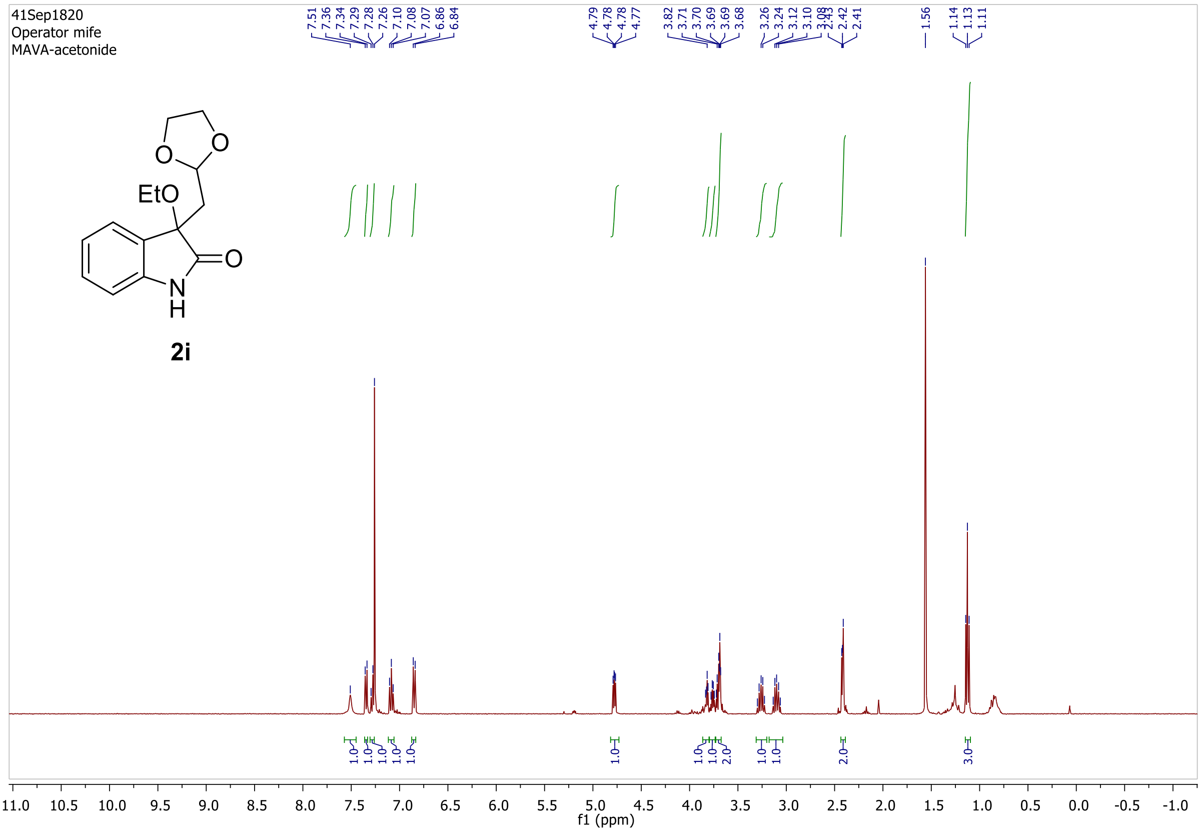

^{1}H NMR in $CDCl_3$ at 400 MHz

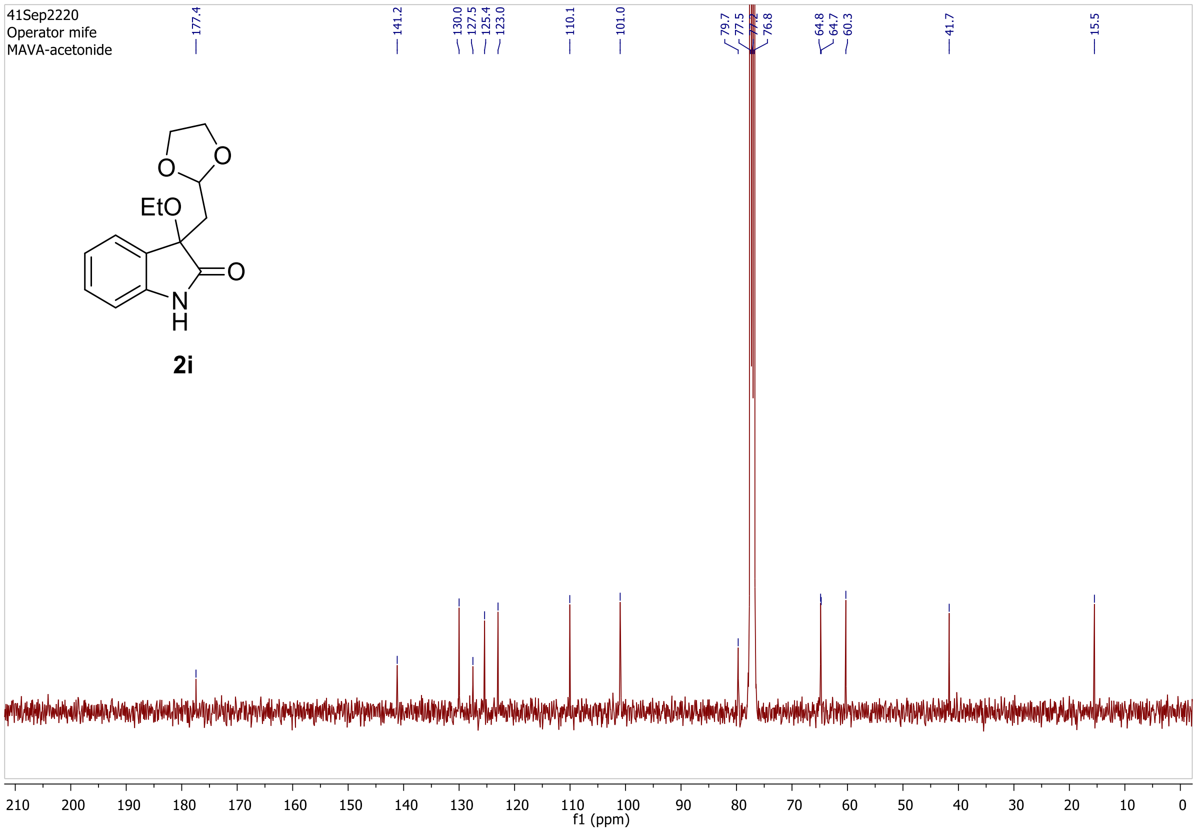

$^{13}C\{^{1}H\}$ NMR in $CDCl_3$ at 100 MHz

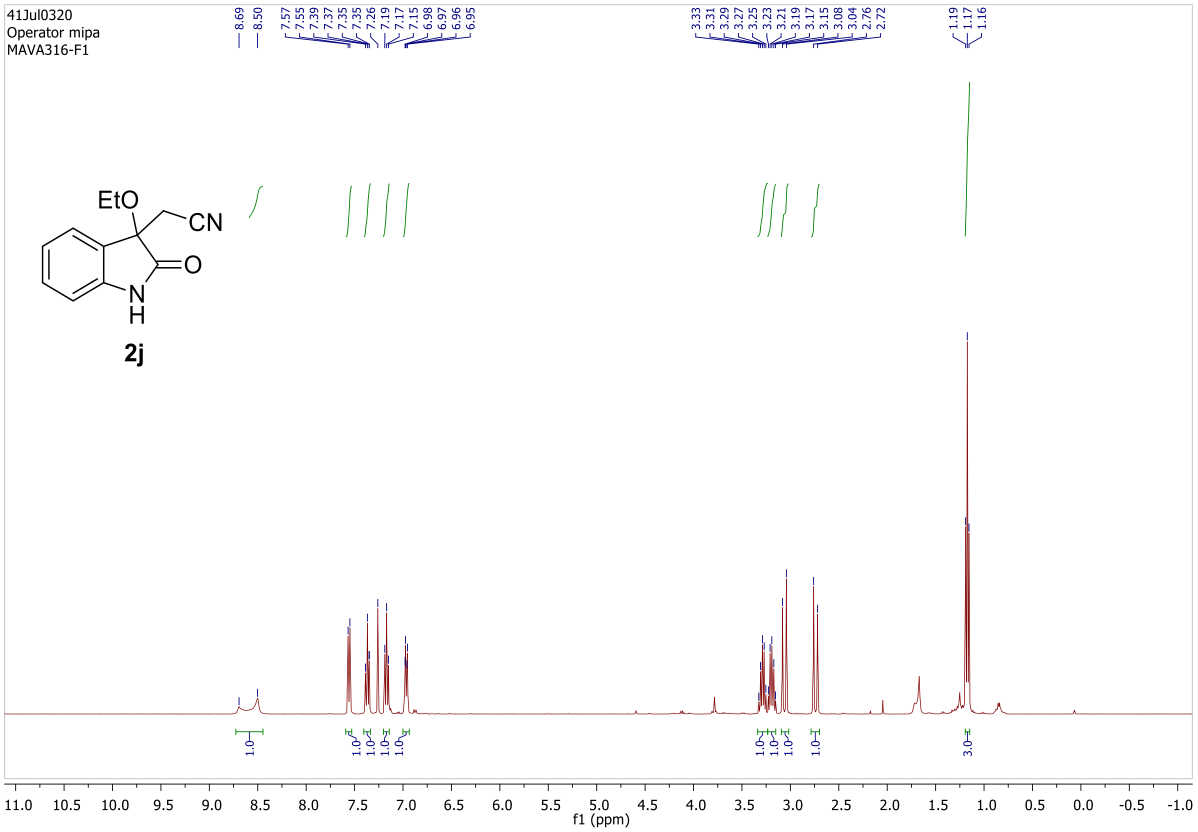

^{1}H NMR in $CDCl_3$ at 400 MHz

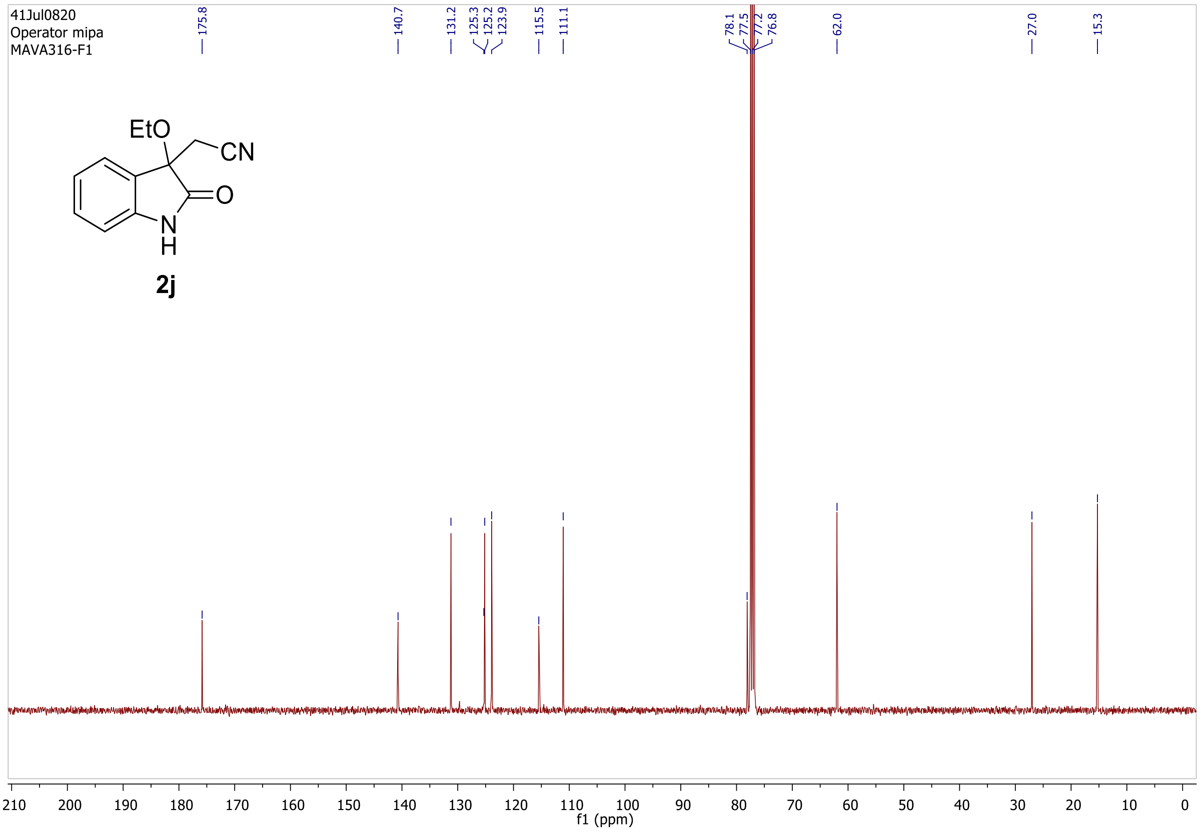

^{13}C{^{1}H} NMR in $CDCl_3$ at 100 MHz

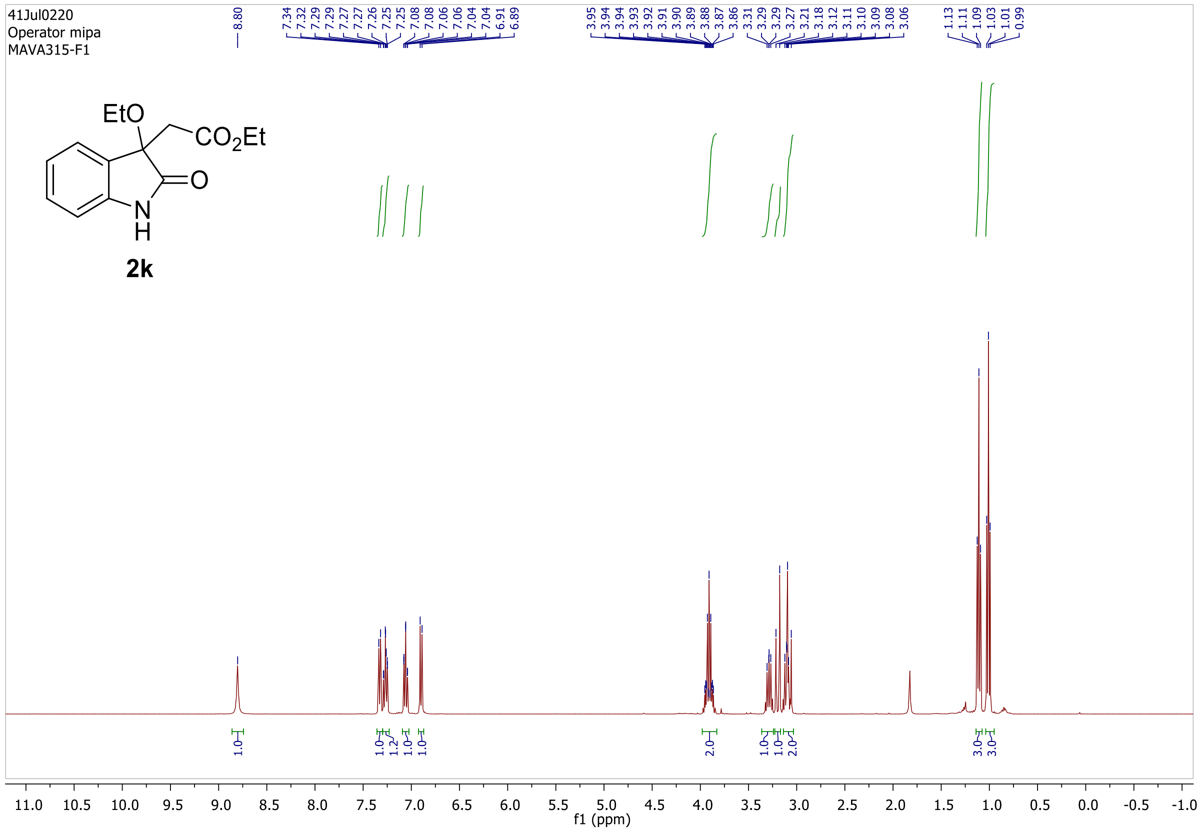

^{1}H NMR in $CDCl_3$ at 400 MHz

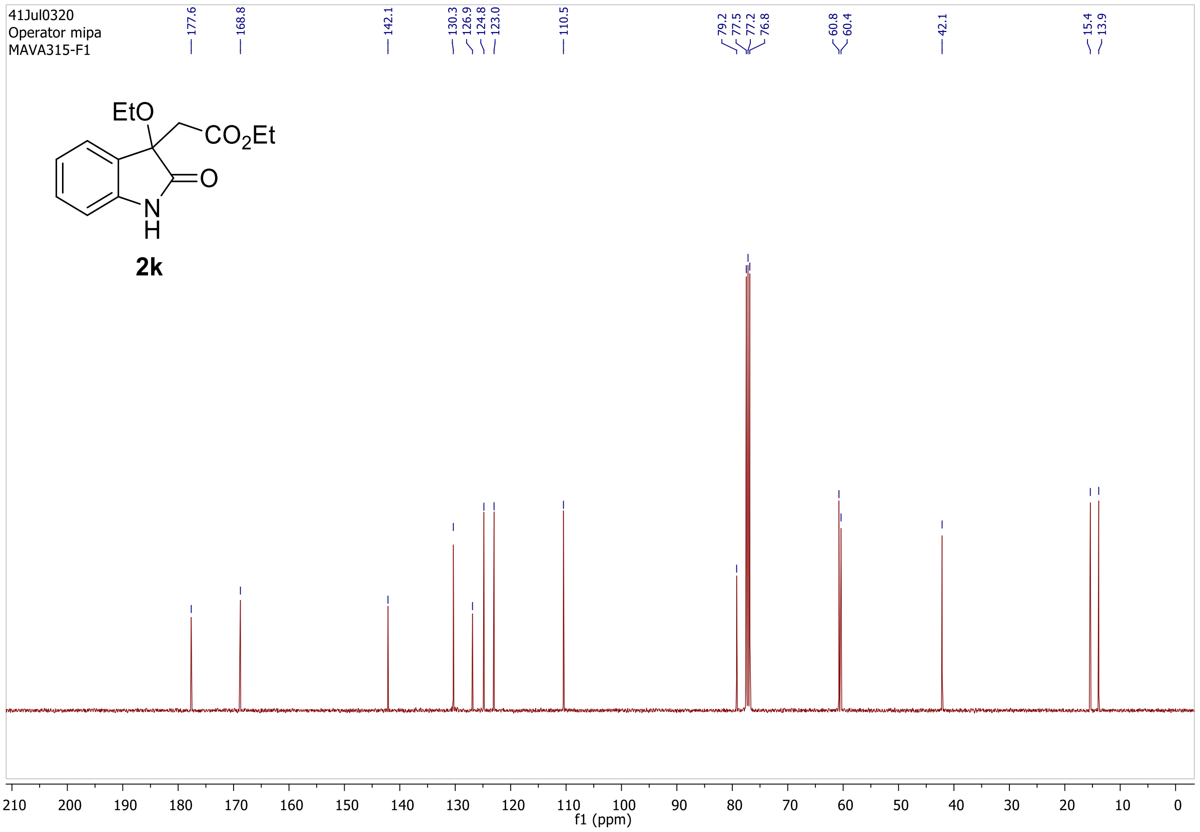

^{13}C{^{1}H} NMR in $CDCl_3$ at 100 MHz

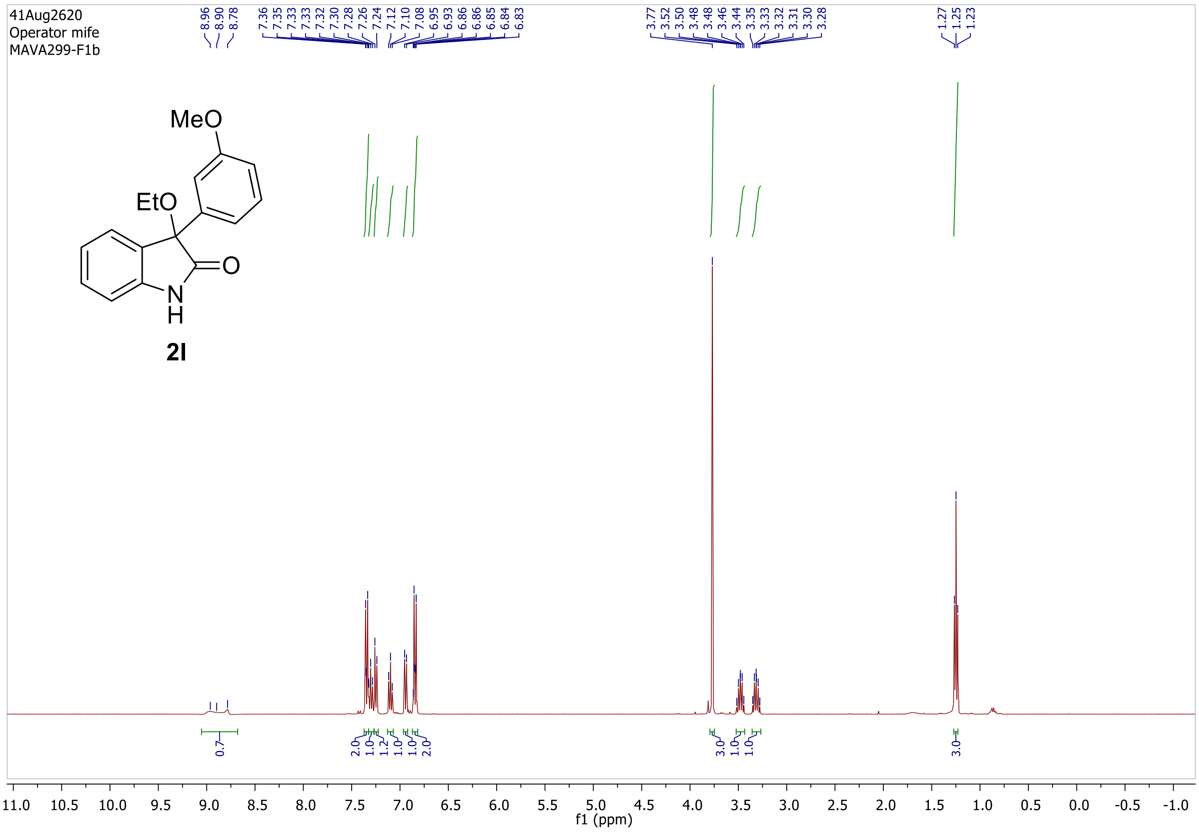

^{1}H NMR in $CDCl_3$ at 400 MHz

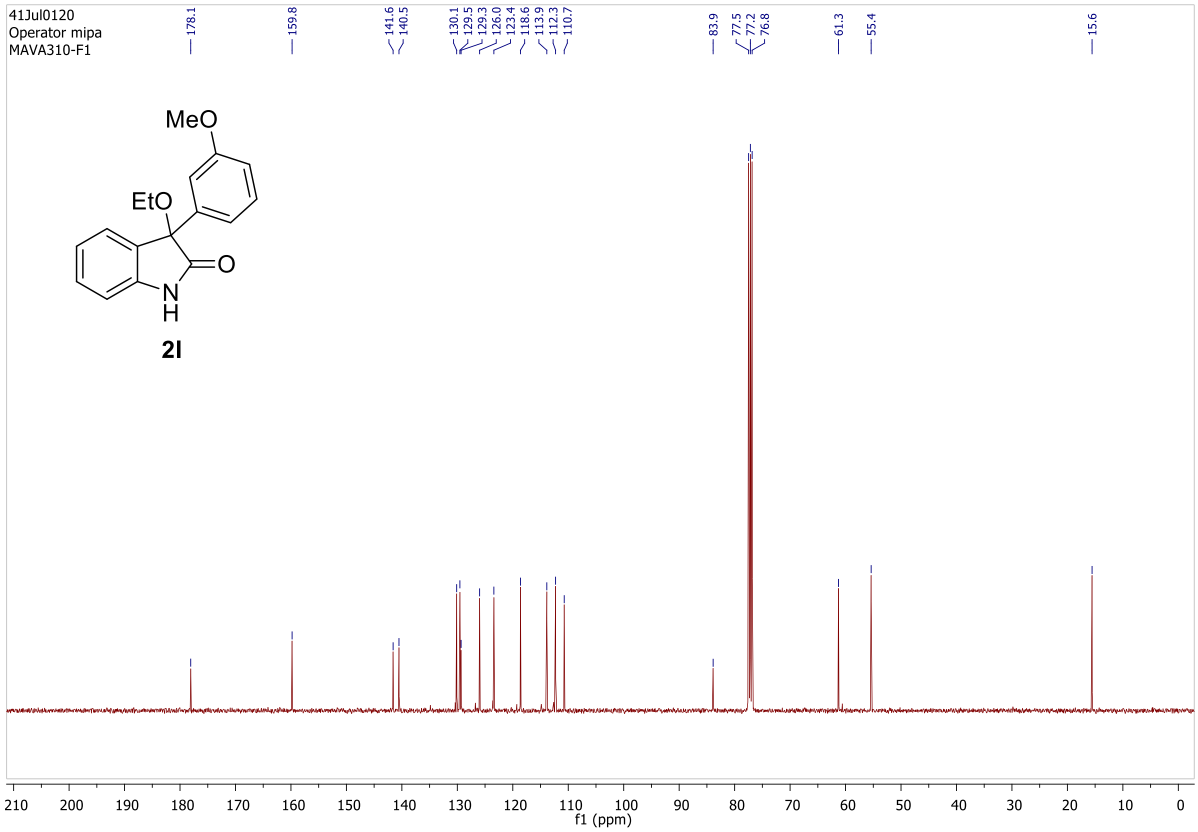

$^{13}C\{^{1}H\}$ NMR in $CDCl_3$ at 100 MHz

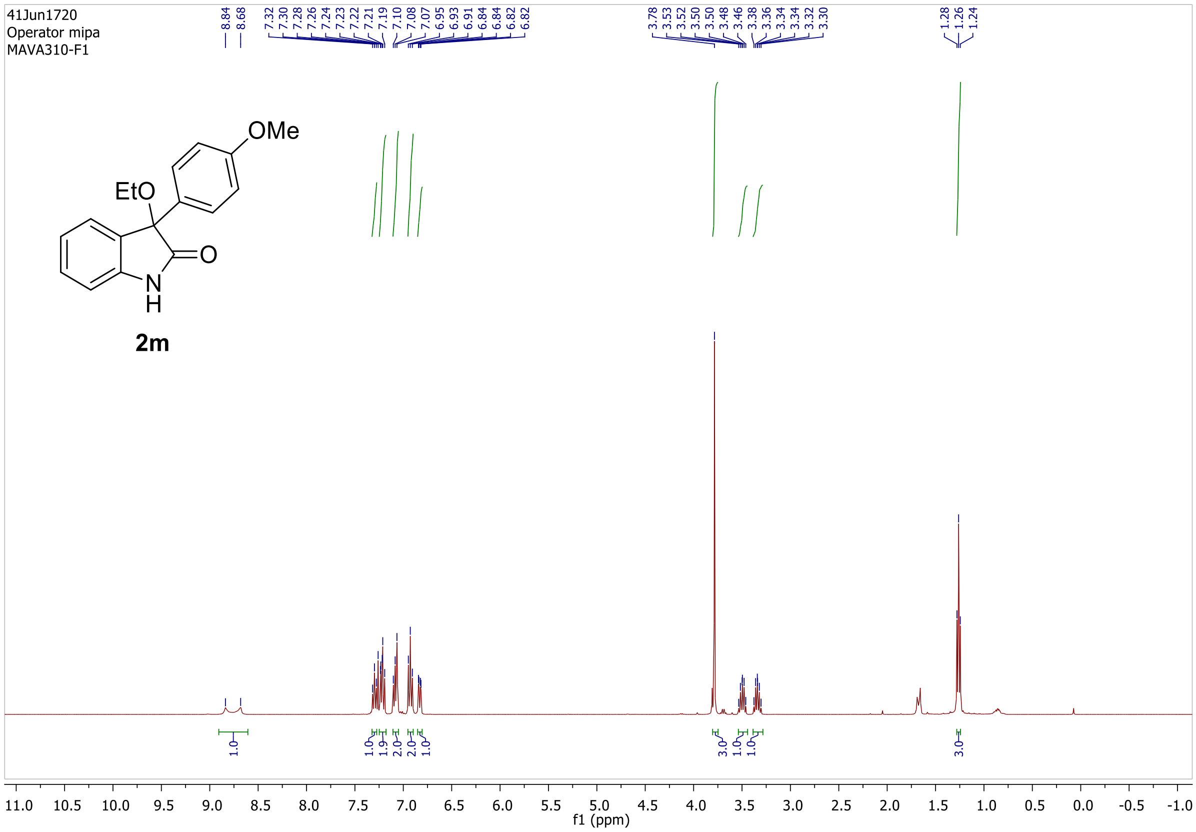

^{1}H NMR in $CDCl_3$ at 400 MHz

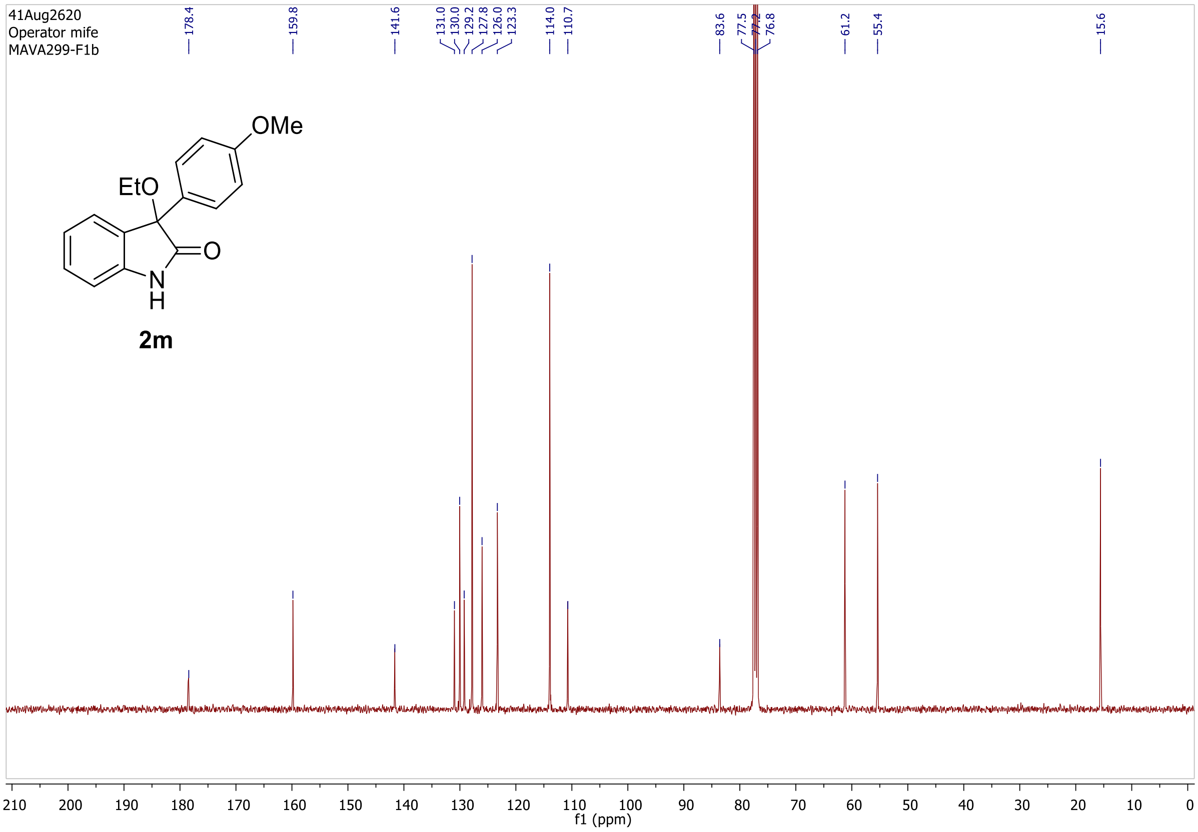

$^{13}C\{^{1}H\}$ NMR in $CDCl_3$ at 100 MHz

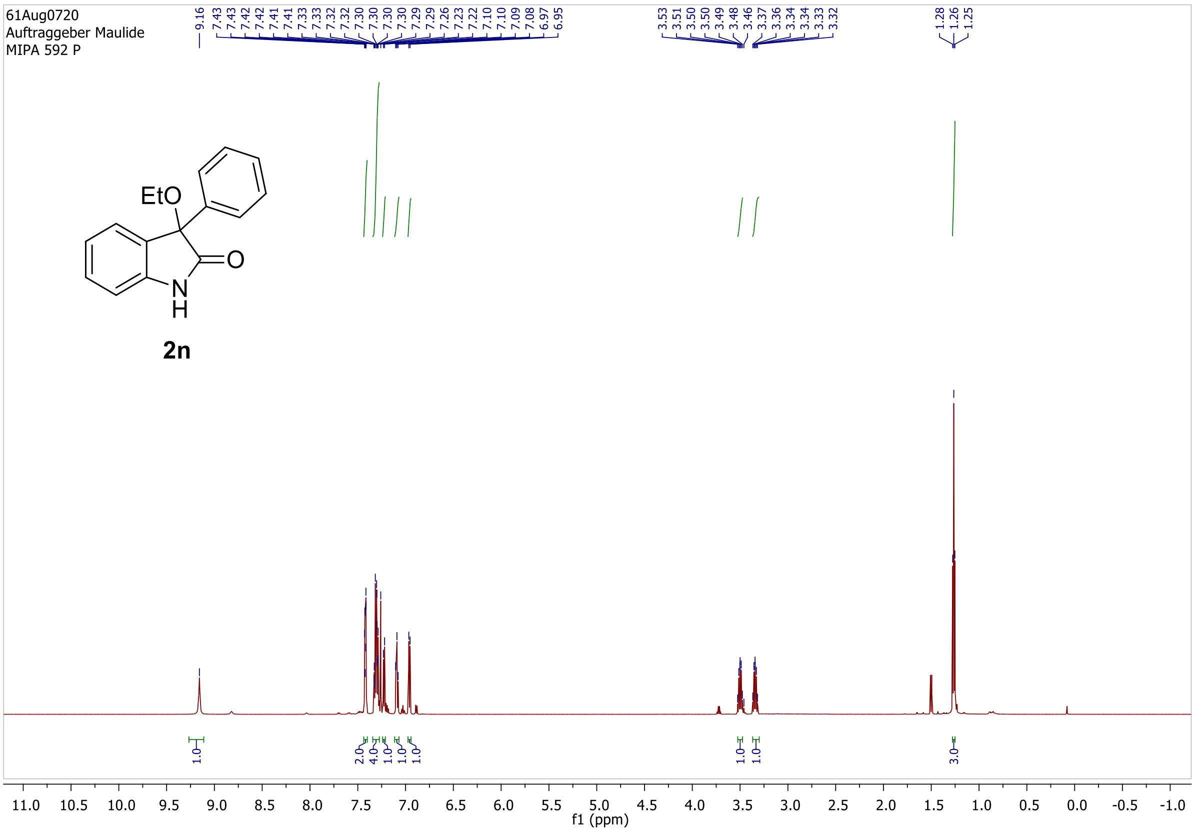

^{1}H NMR in $CDCl_3$ at 600 MHz

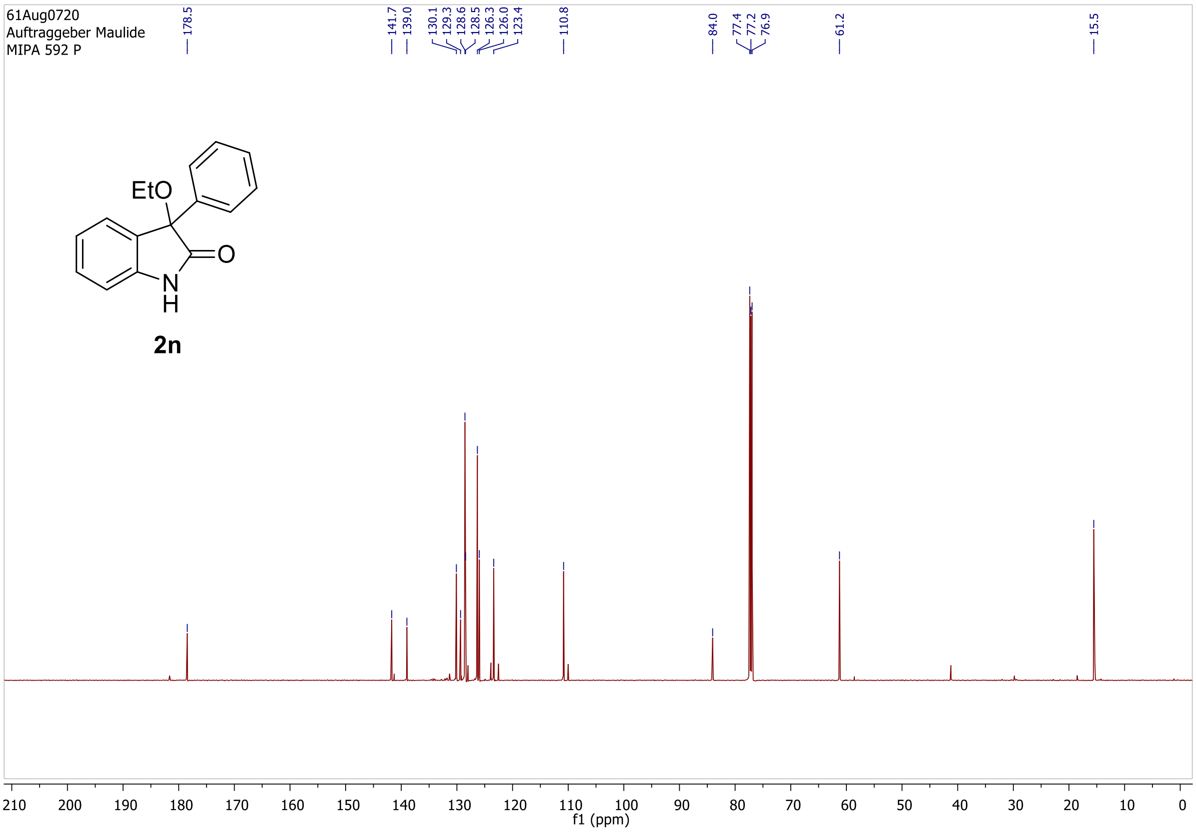

$^{13}C\{^{1}H\}$ NMR in $CDCl_3$ at 150 MHz

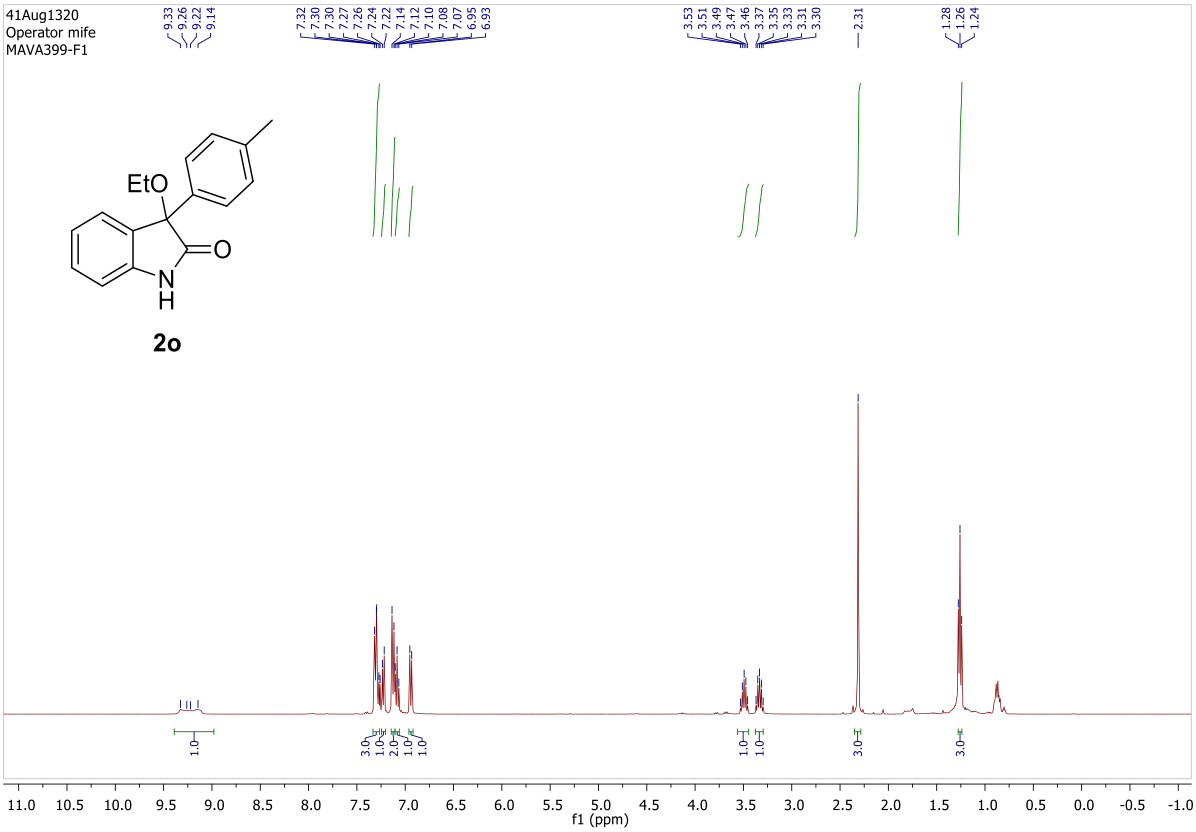

^{1}H NMR in $CDCl_3$ at 400 MHz

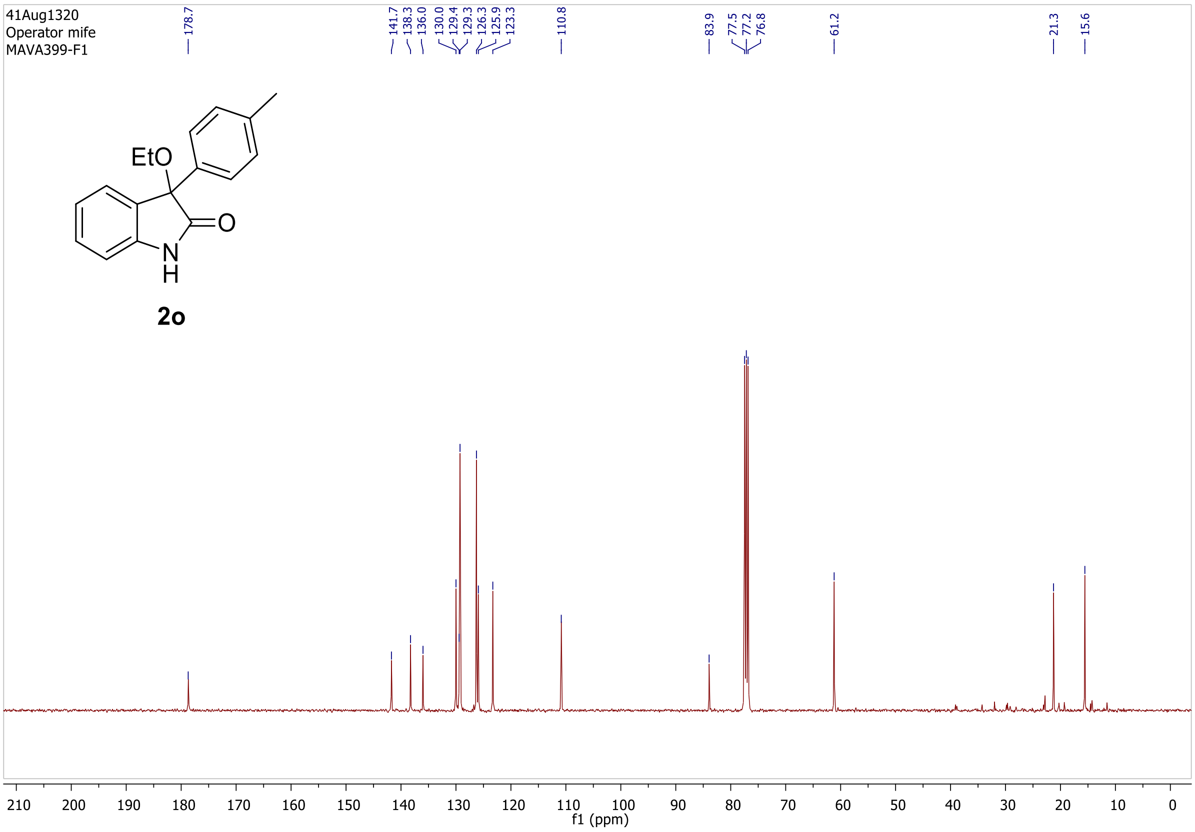

$^{13}C\{^{1}H\}$ NMR in $CDCl_3$ at 100 MH

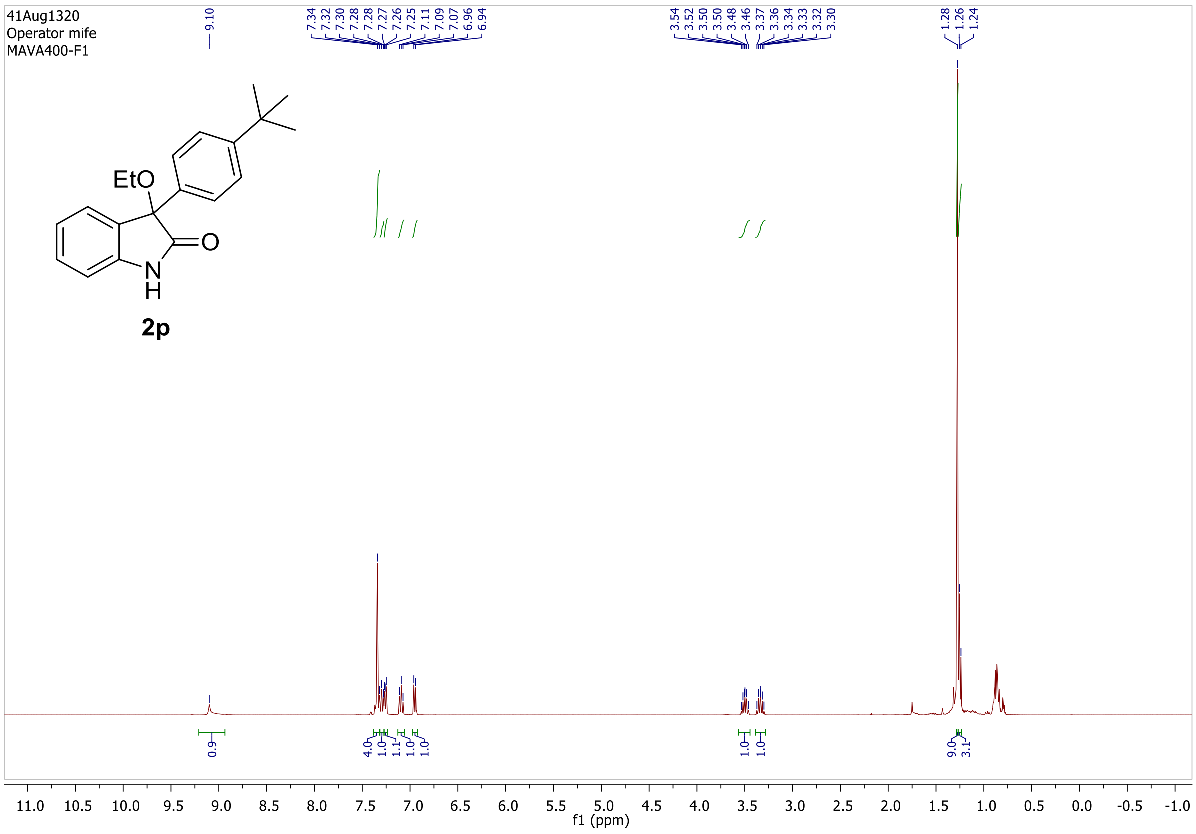

^{1}H NMR in $CDCl_3$ at 400 MHz

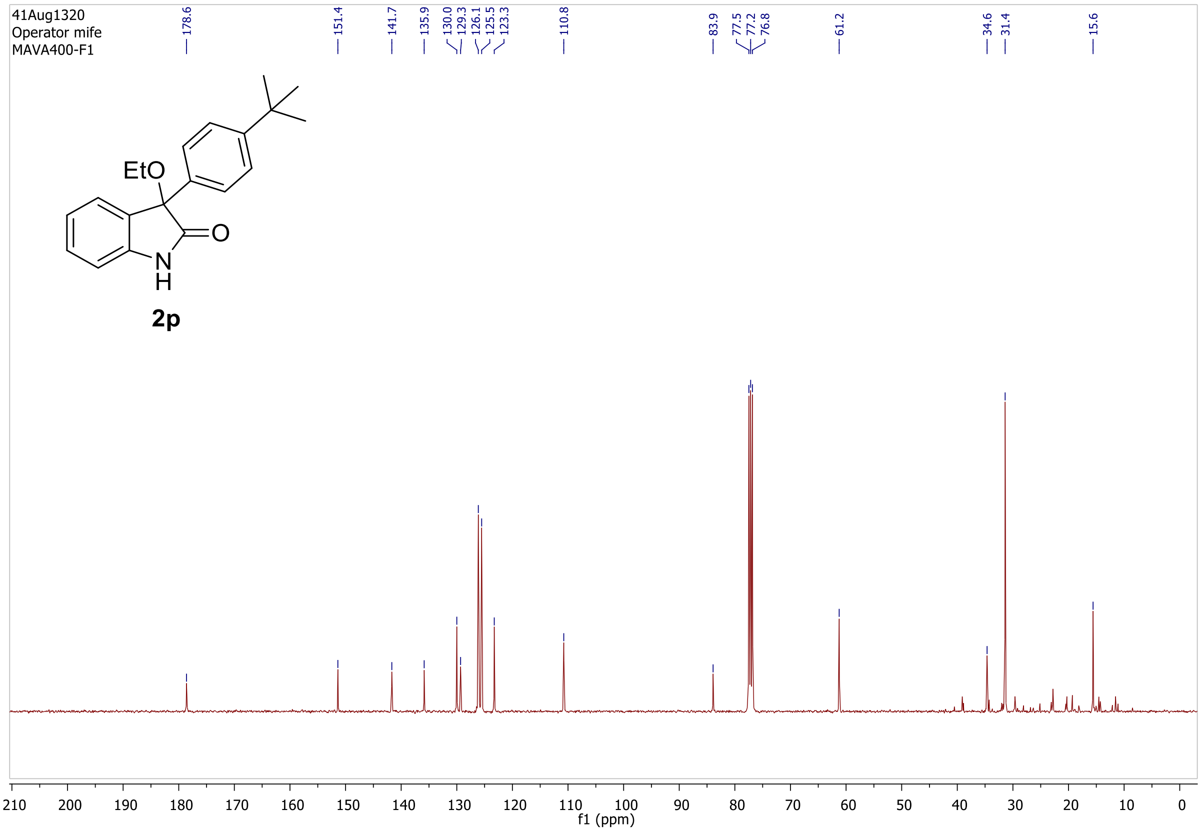

$^{13}C\{^{1}H\}$ NMR in $CDCl_3$ at 100 MHz

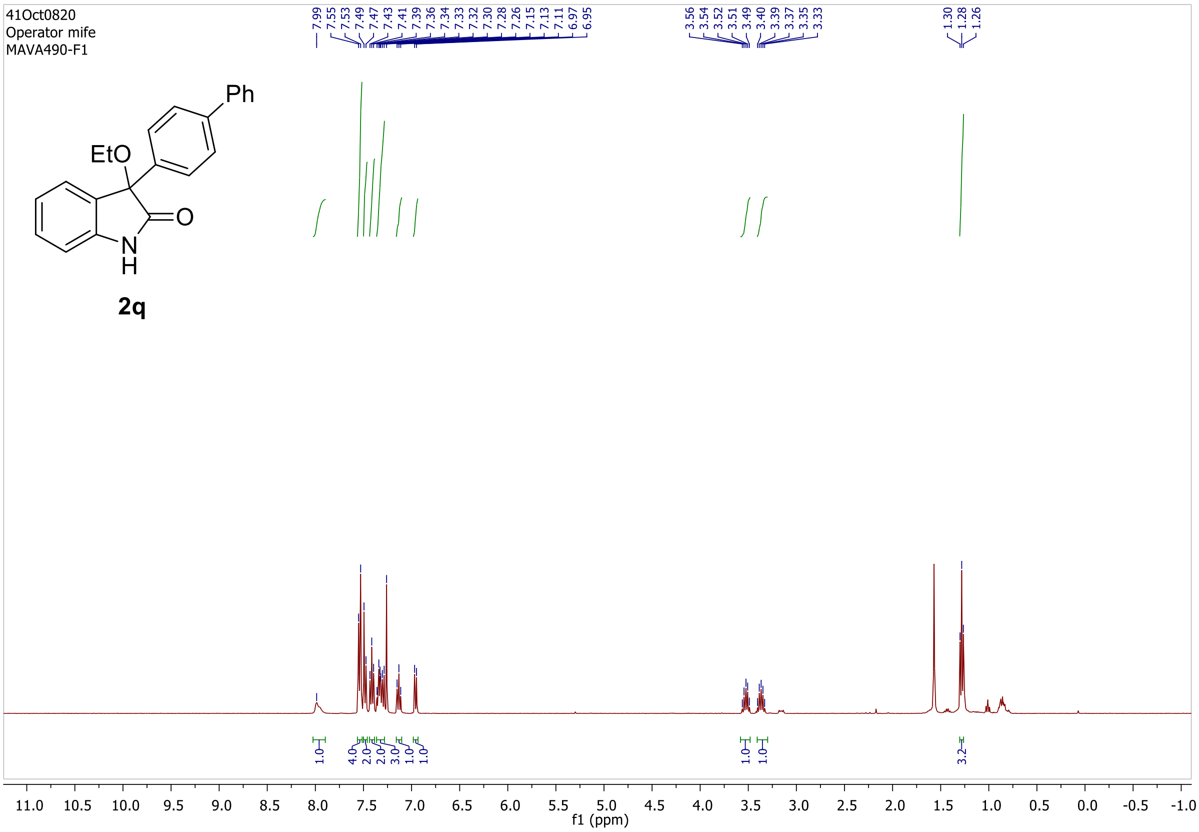

^{1}H NMR in $CDCl_3$ at 400 MHz

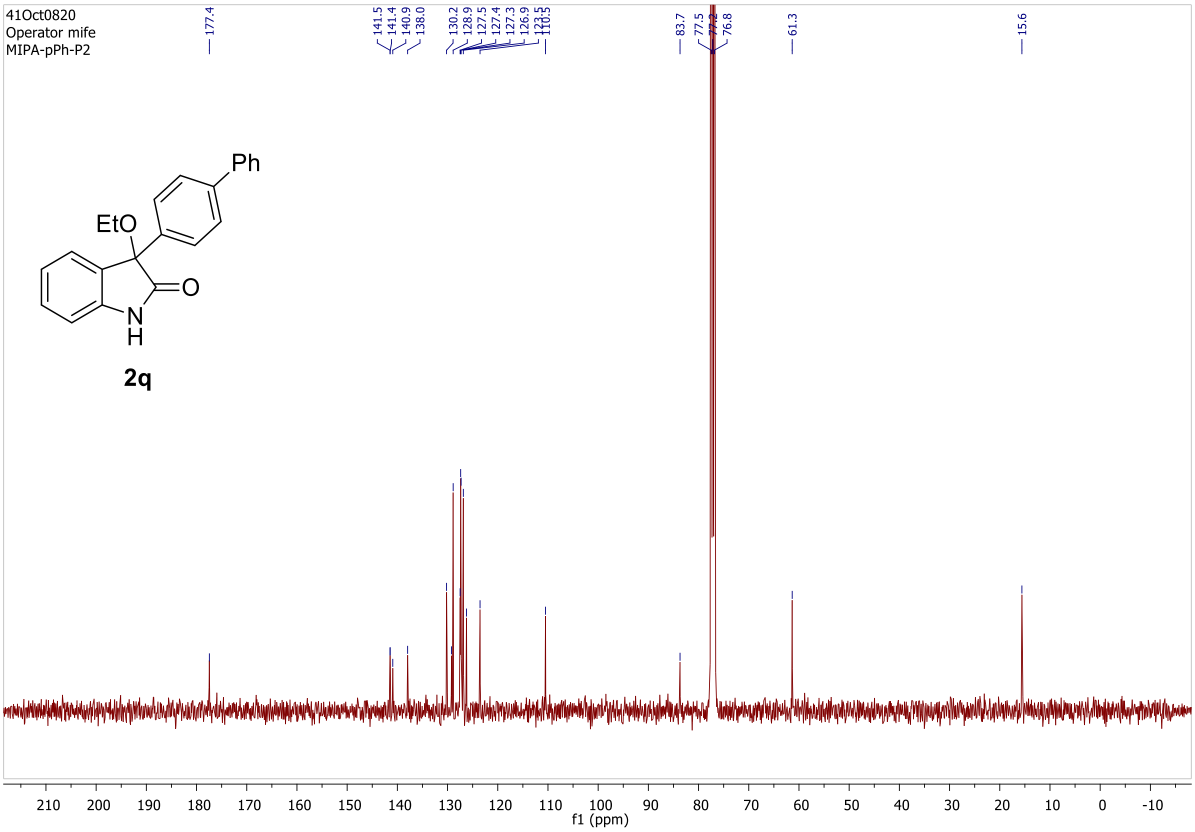

$^{13}C\{^1H\}$ NMR in $CDCl_3$ at 100 MHz

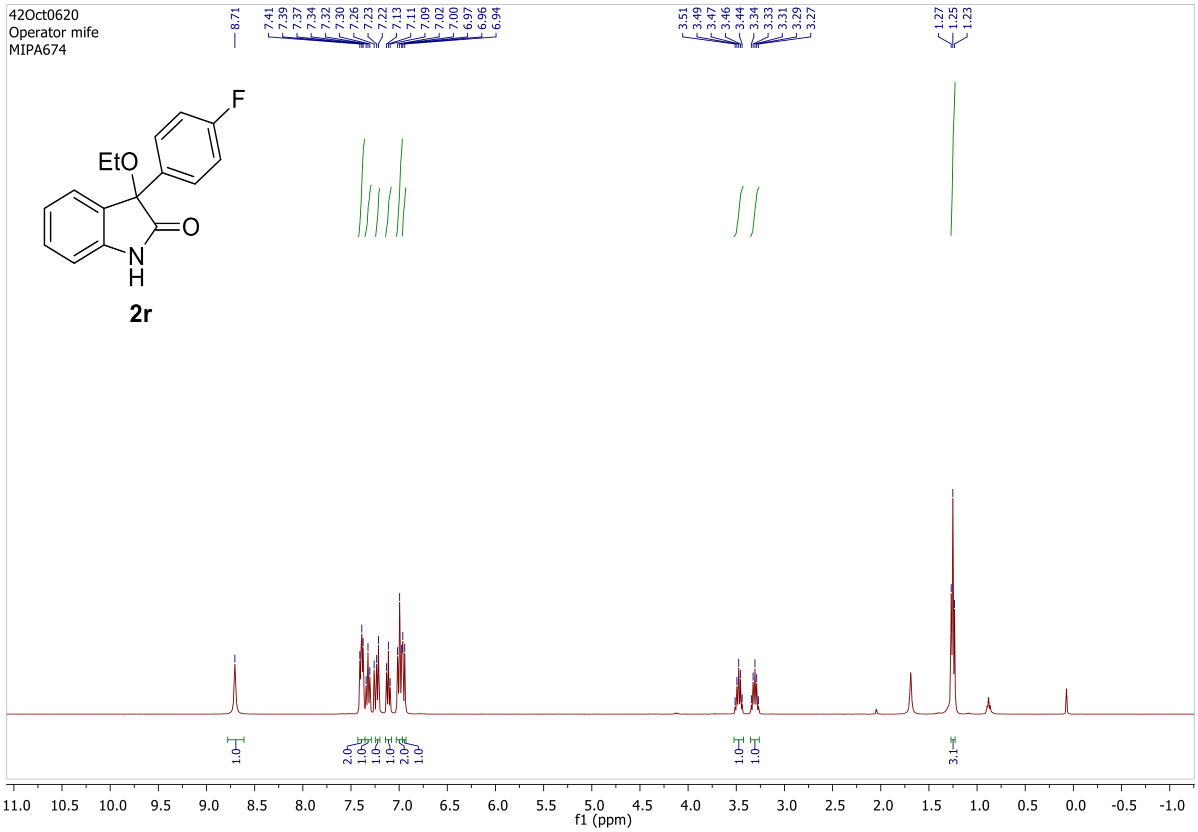

^{1}H NMR in $CDCl_3$ at 600 MHz

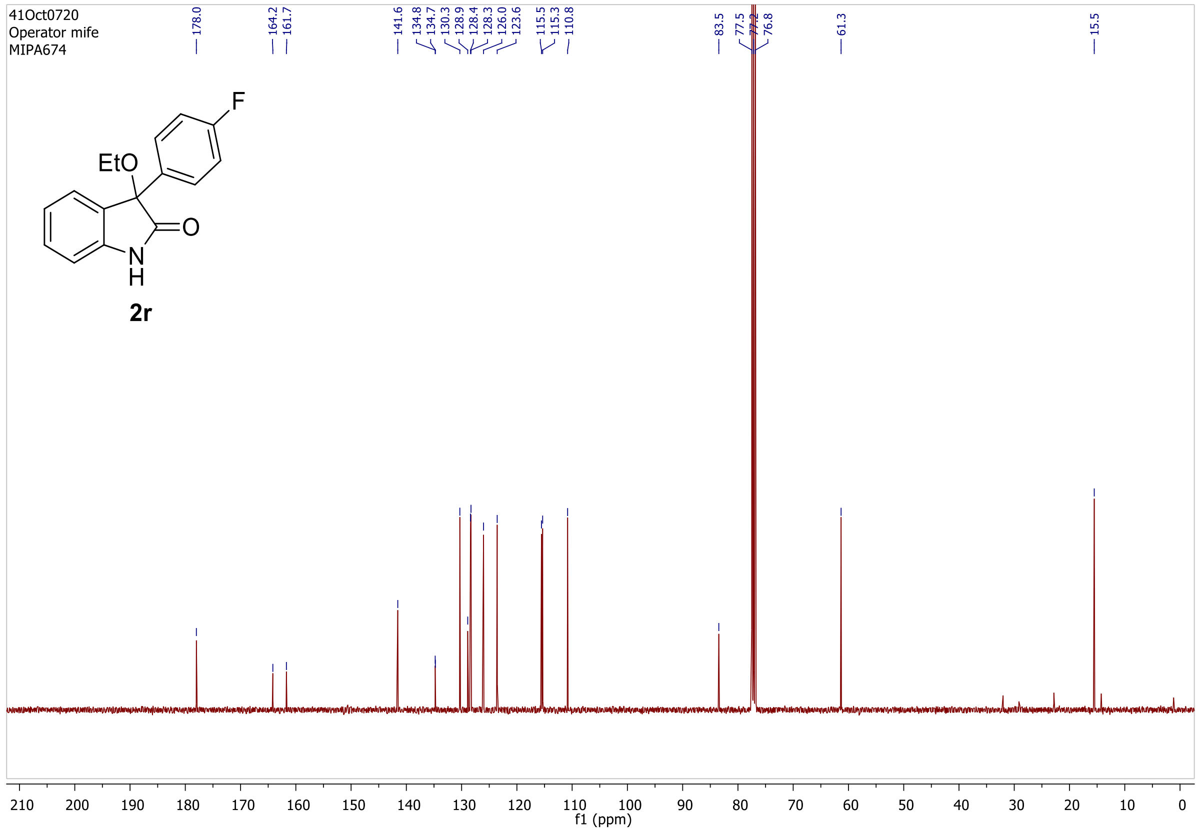

$^{13}C\{^{1}H\}$ NMR in $CDCl_3$ at 150 MHz

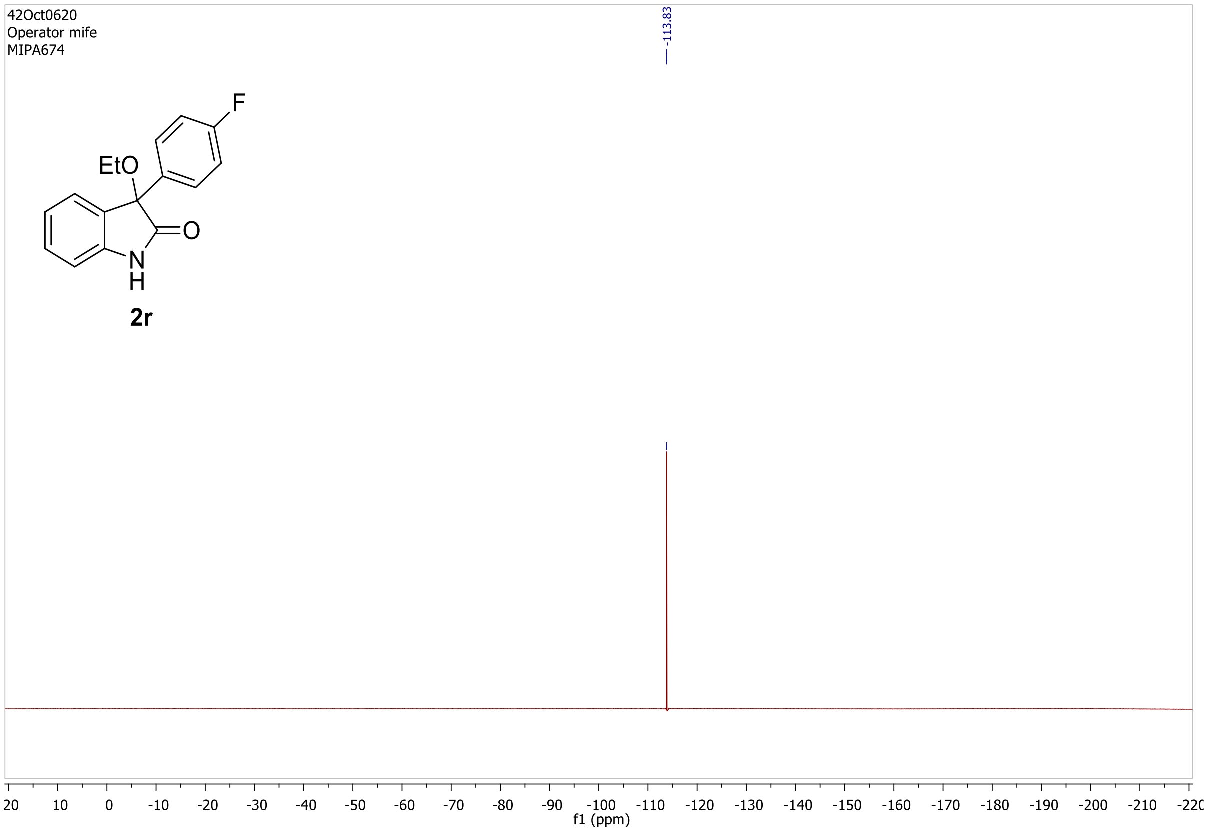

^{19}F NMR in $CDCl_3$ at 376 MHz

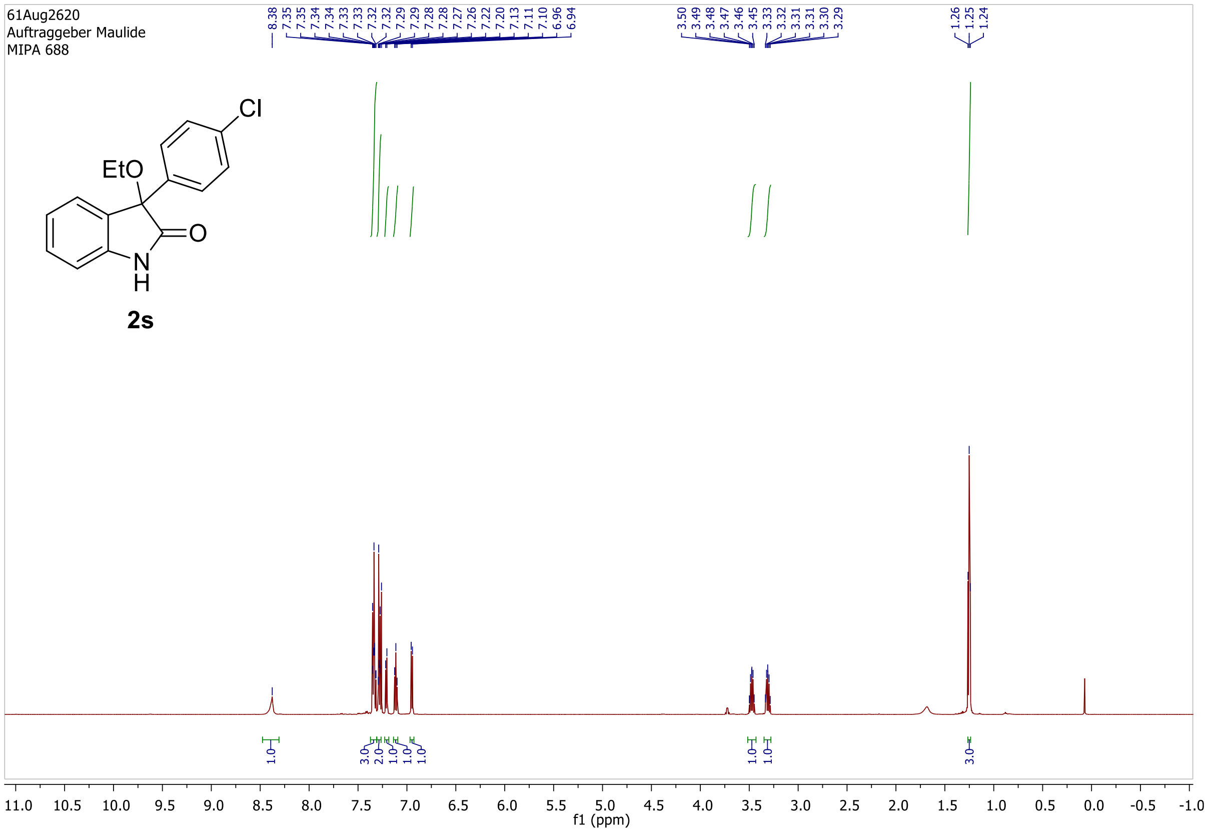

^{1}H NMR in $CDCl_3$ at 600 MHz

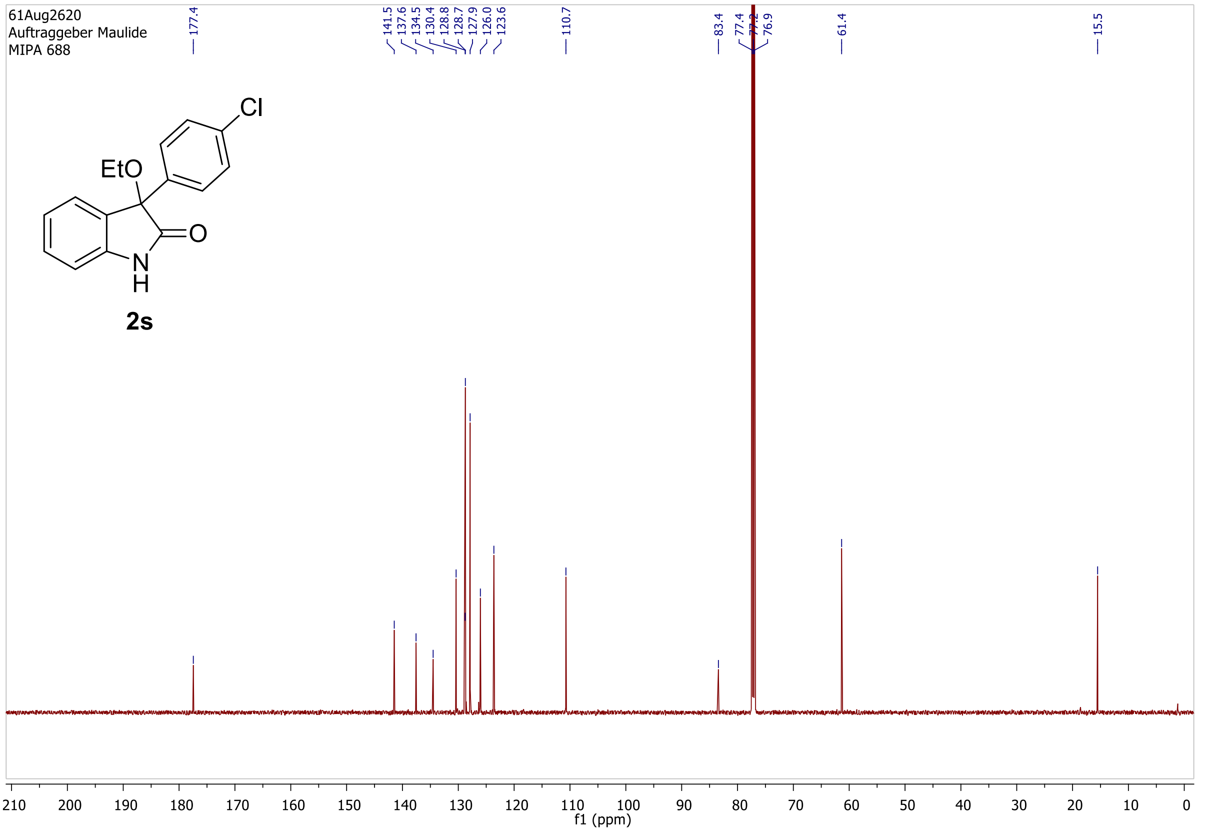

^{13}C{^{1}H} NMR in $CDCl_3$ at 150 MHz

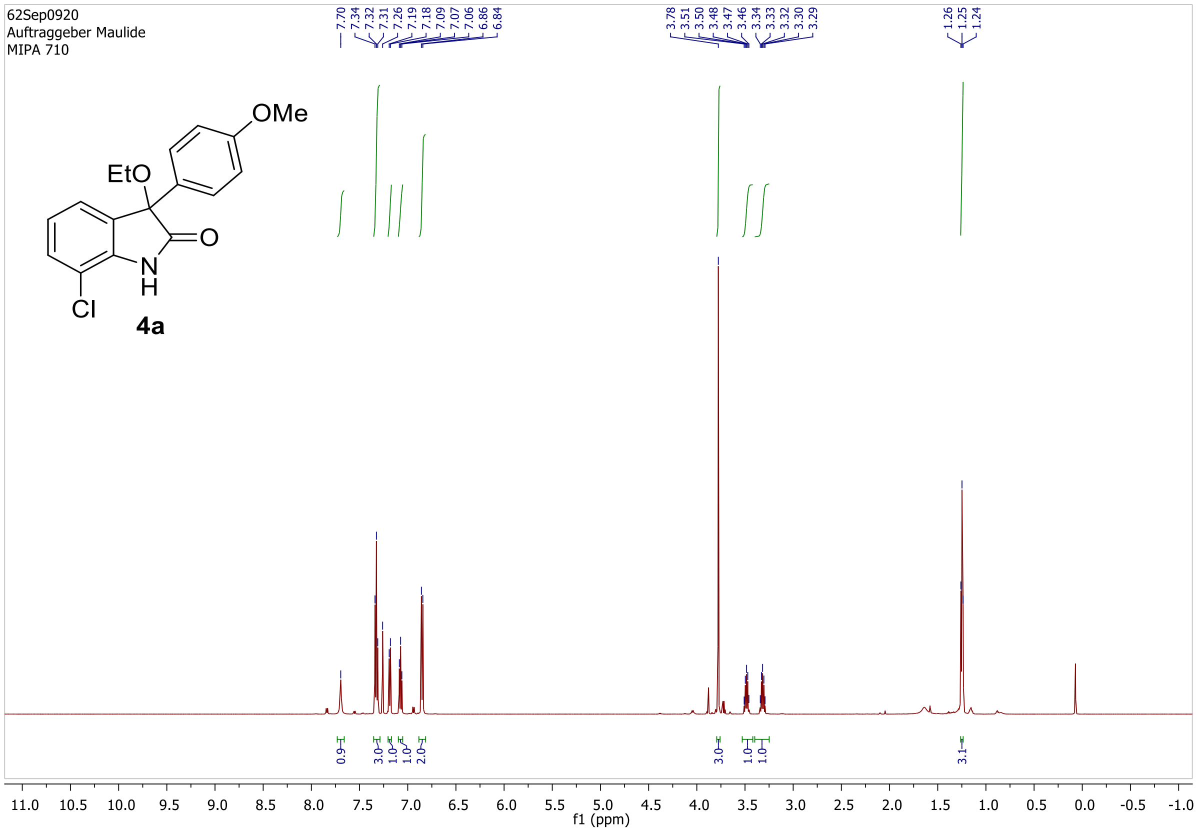

^{1}H NMR in $CDCl_3$ at 600 MHz

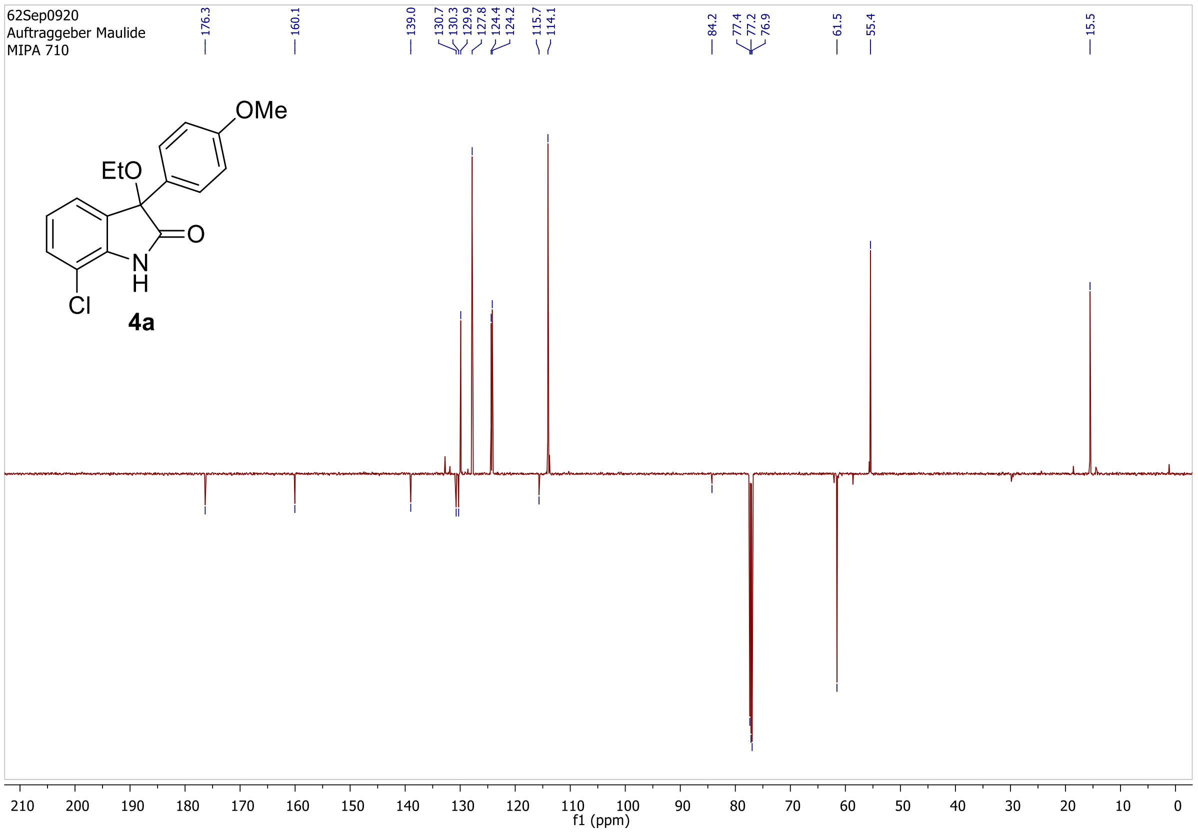

$^{13}C\{^{1}H\}$ NMR in $CDCl_3$ at 150 MHz

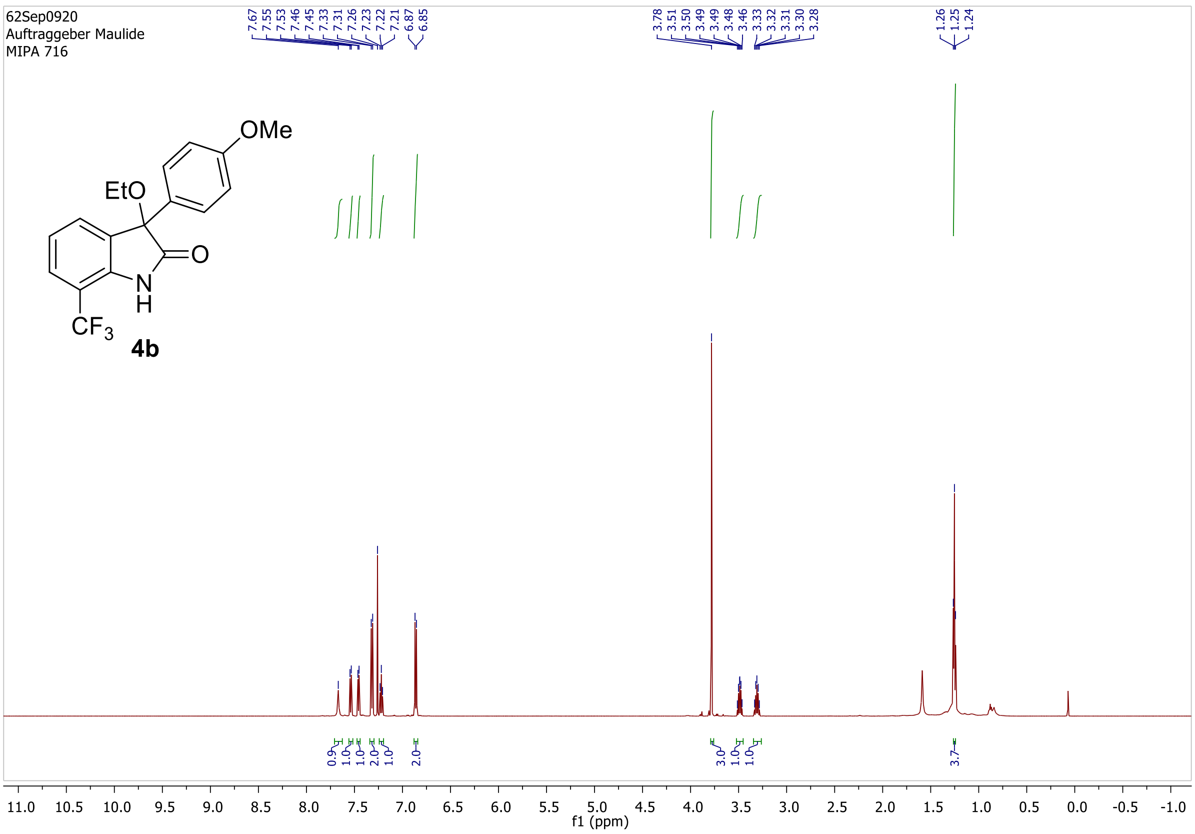

^{1}H NMR in $CDCl_3$ at 600 MHz

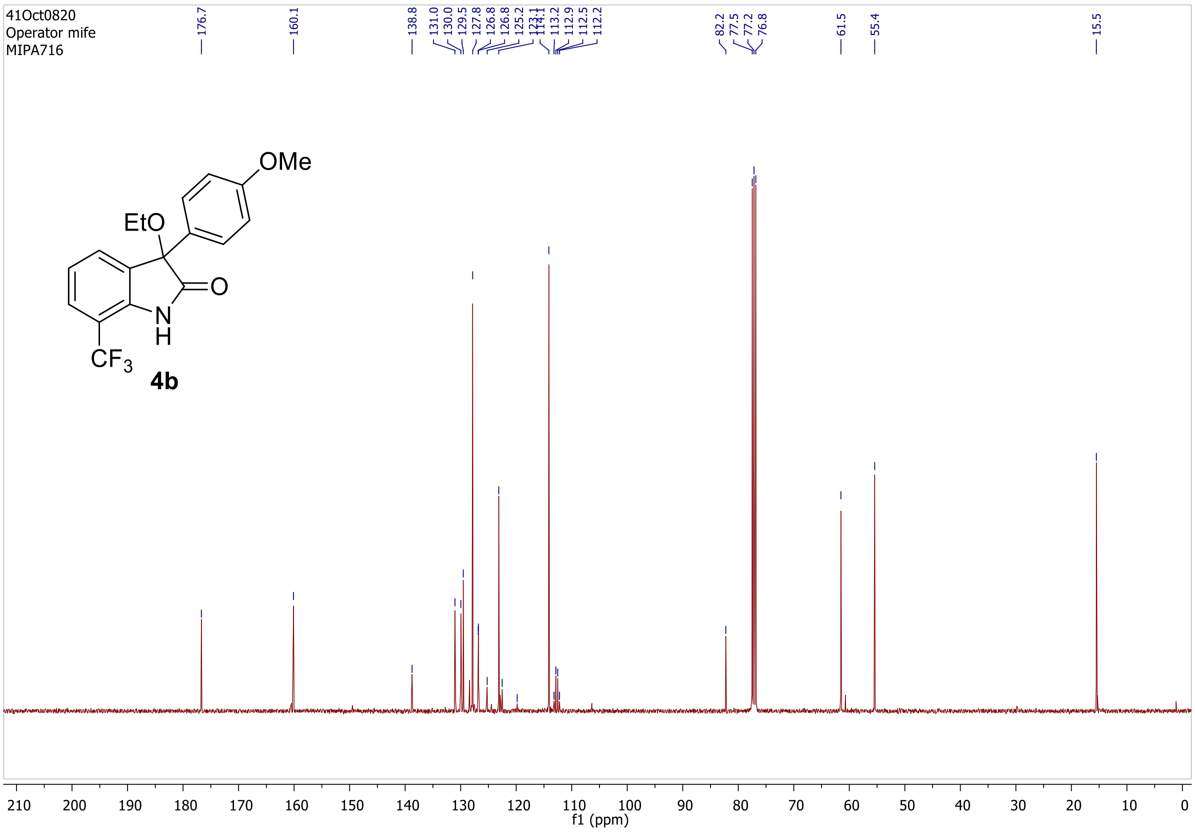

$^{13}C\{^{1}H\}$ NMR in $CDCl_3$ at 150 MHz

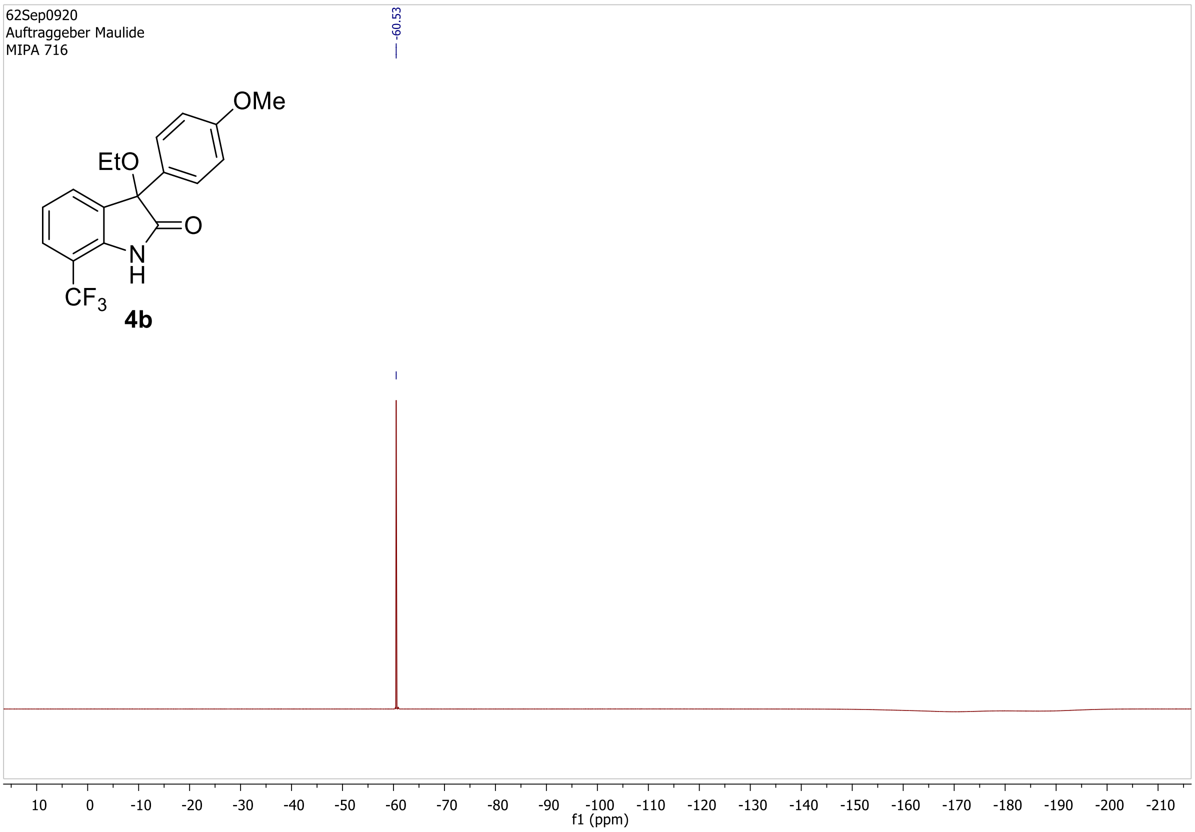

^{19}F NMR in $CDCl_3$ at 565 MHz

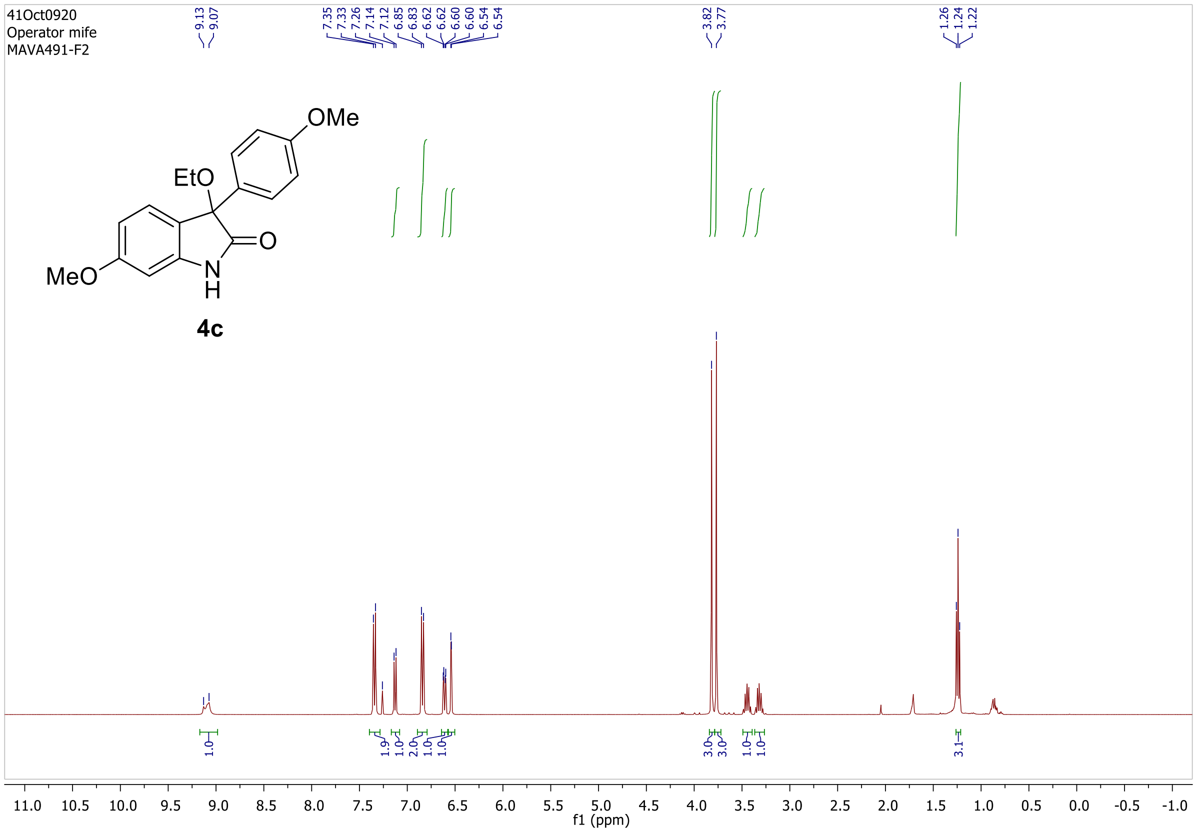

^{1}H NMR in $CDCl_3$ at 400 MHz

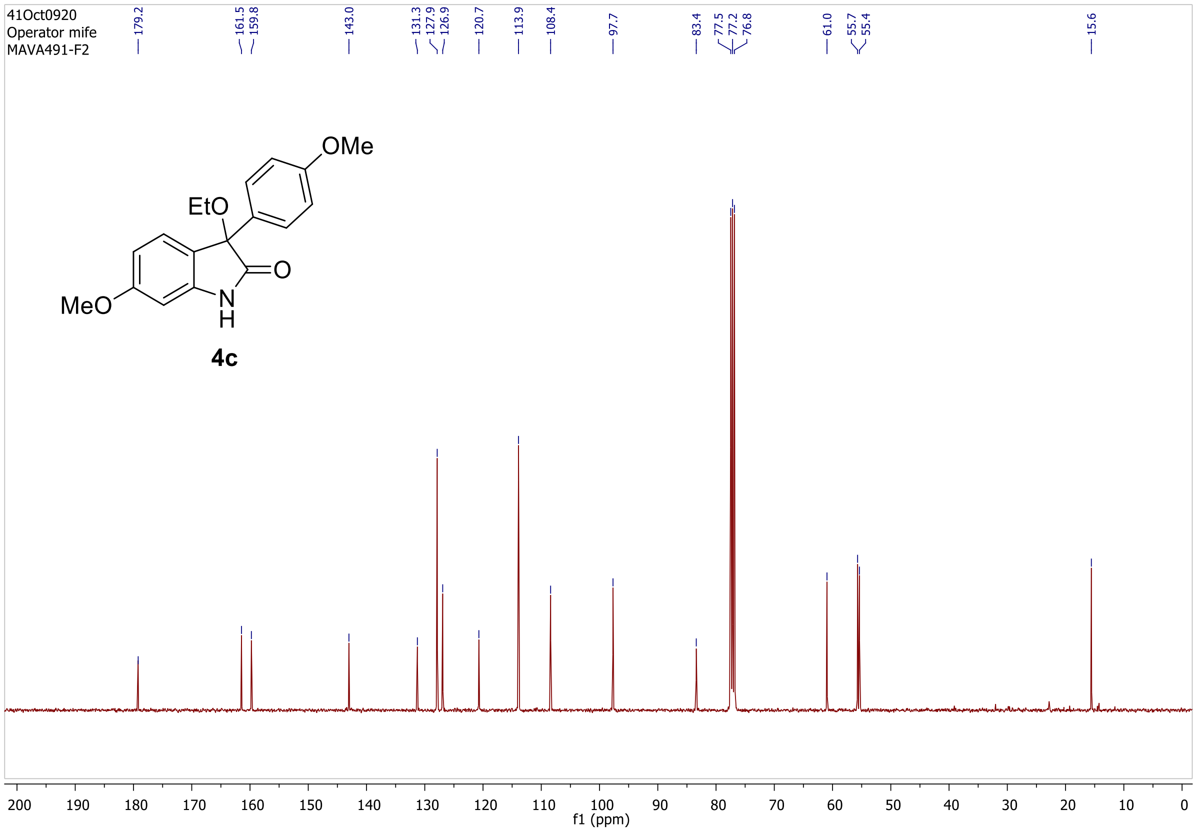

$^{13}C\{^{1}H\}$ NMR in $CDCl_3$ at 100 MHz

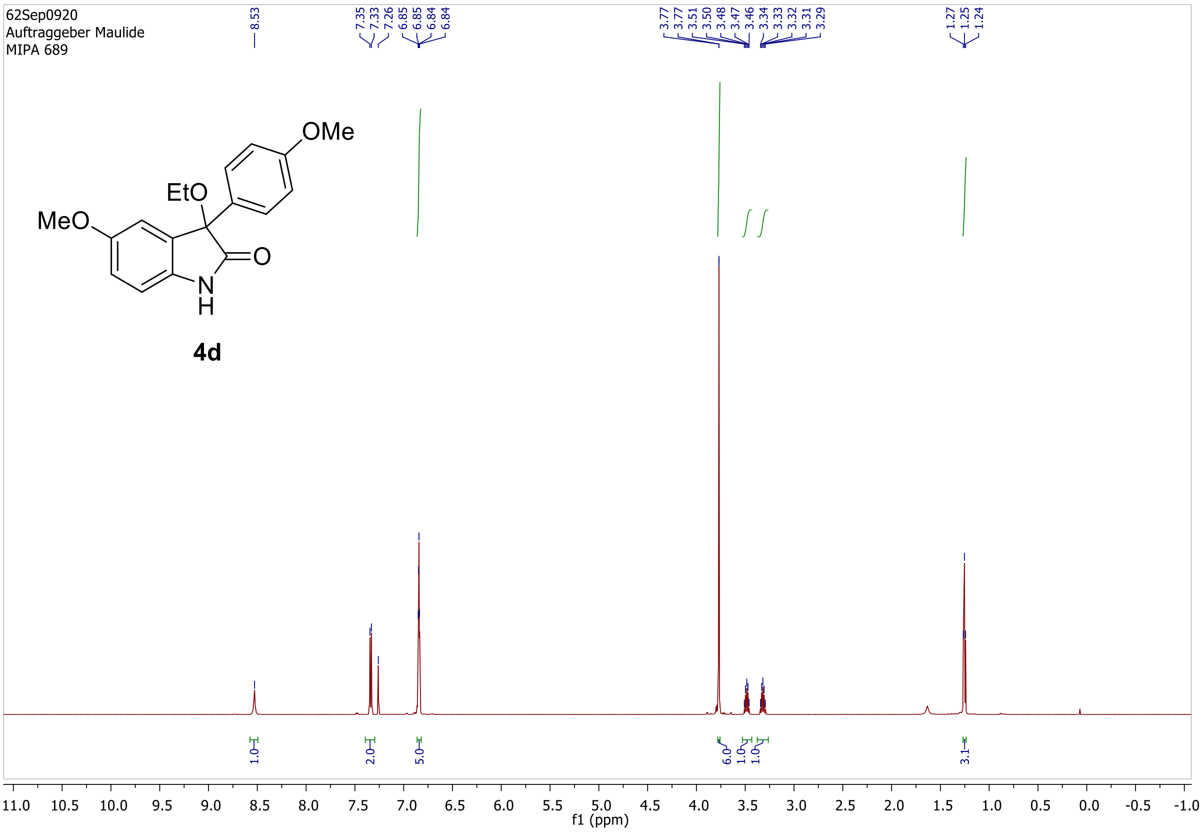

^{1}H NMR in $CDCl_3$ at 600 MHz

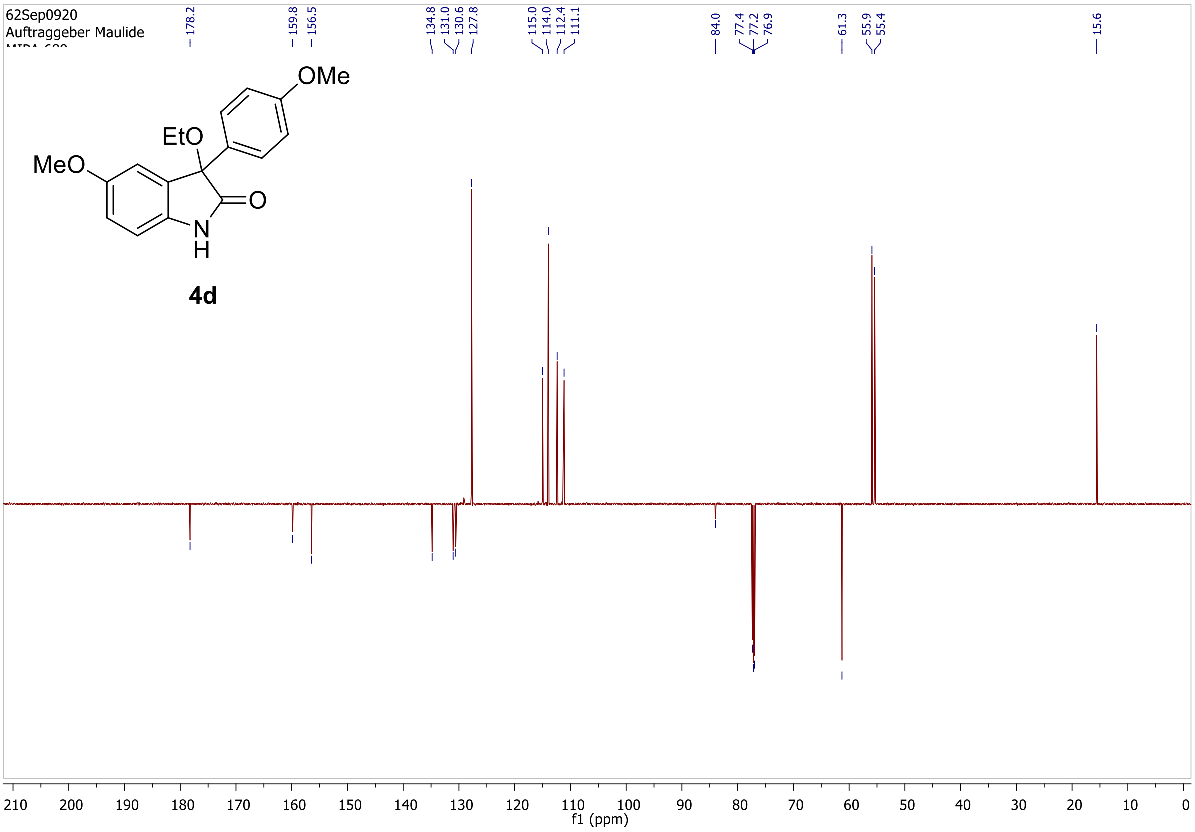

$^{13}C\{^{1}H\}$ NMR in $CDCl_3$ at 150 MHz

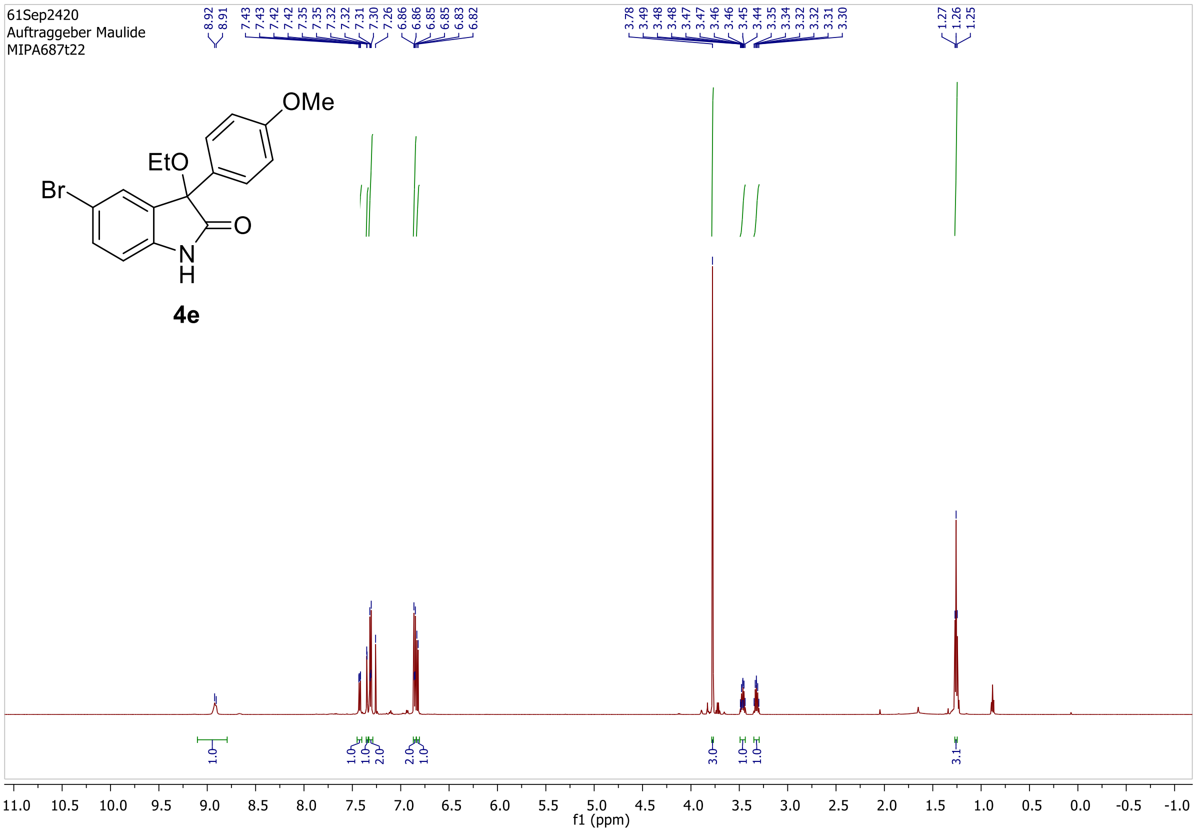

^{1}H NMR in $CDCl_3$ at 600 MHz

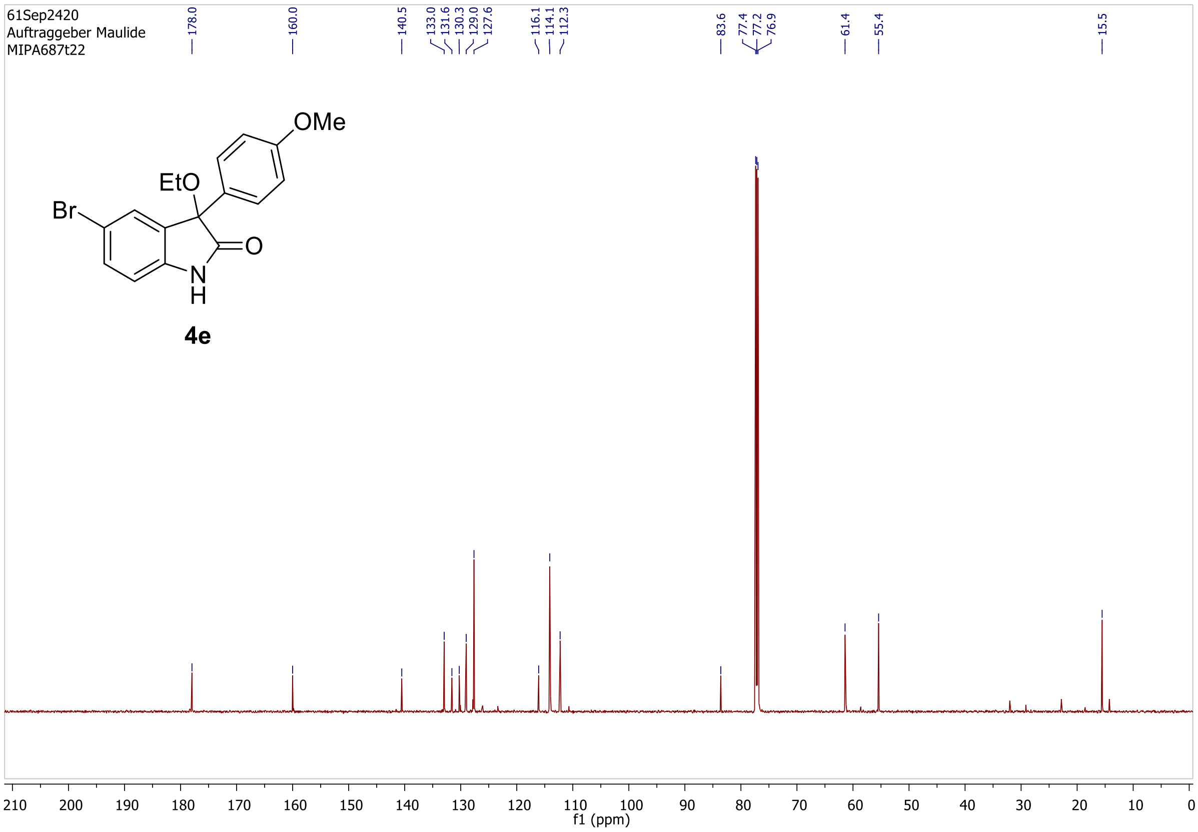

$^{13}C\{^{1}H\}$ NMR in $CDCl_3$ at 150 MHz

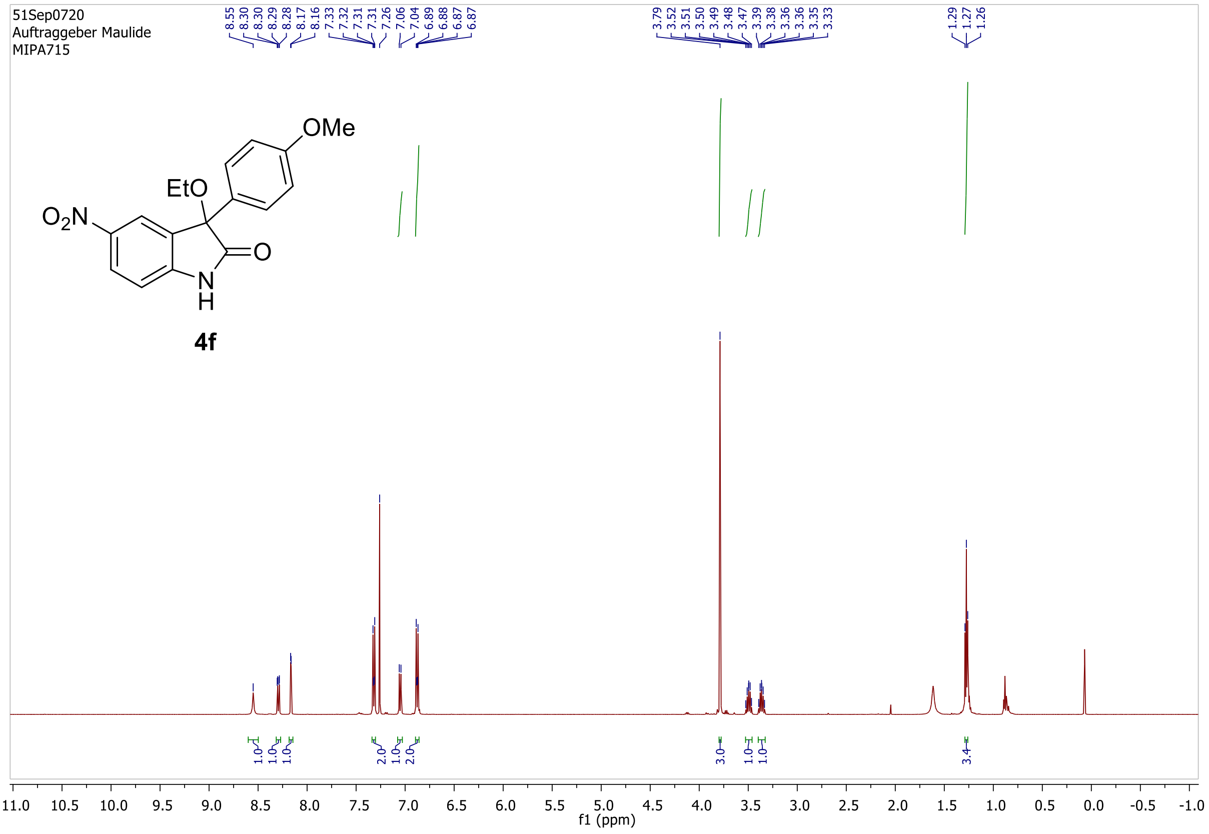

^{1}H NMR in $CDCl_3$ at 500 MHz

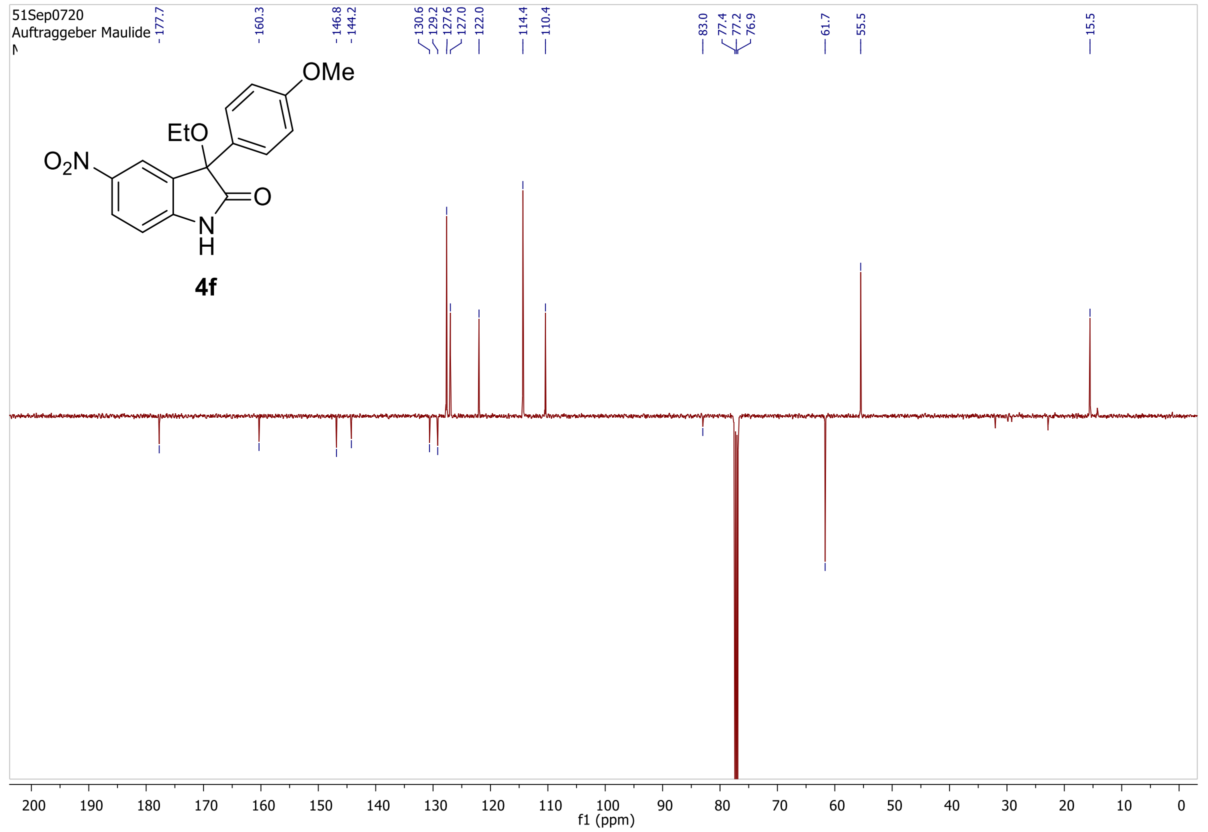

$^{13}C\{^{1}H\}$ NMR in $CDCl_3$ at 126 MHz

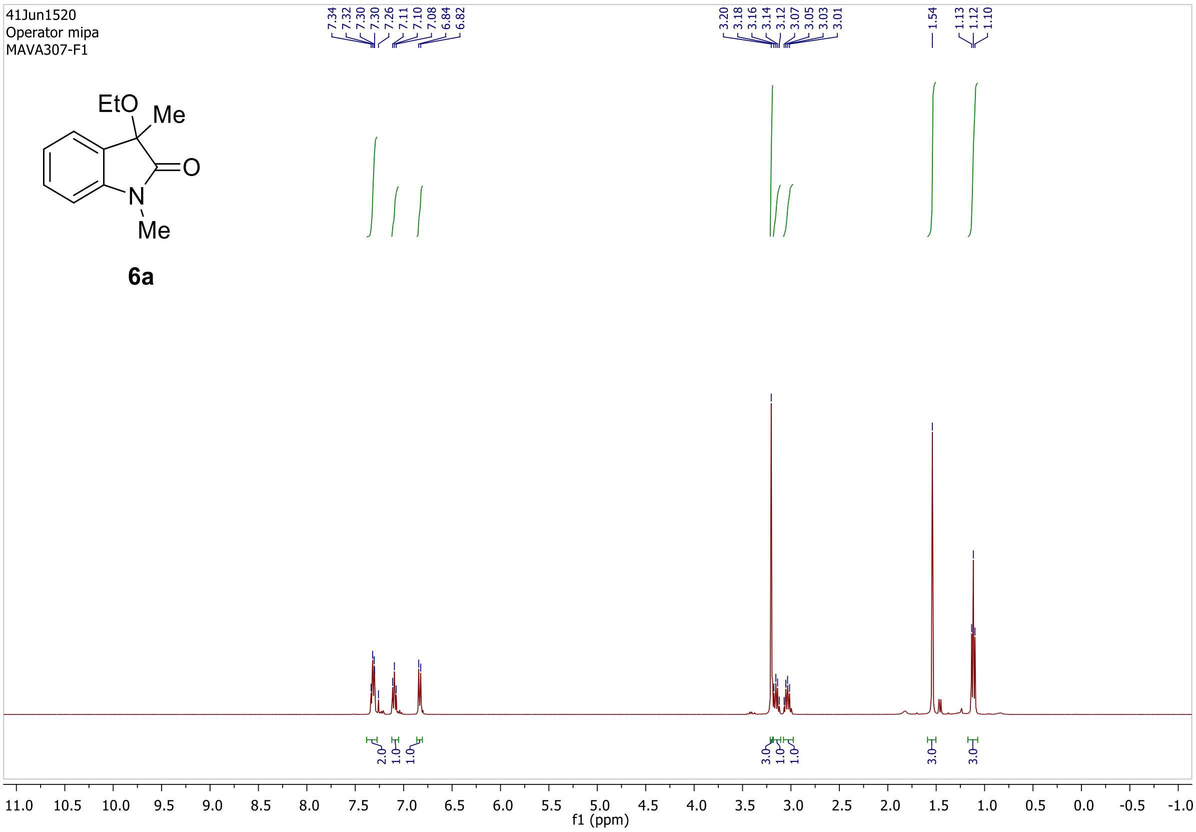

^{1}H NMR in $CDCl_3$ at 400 MHz

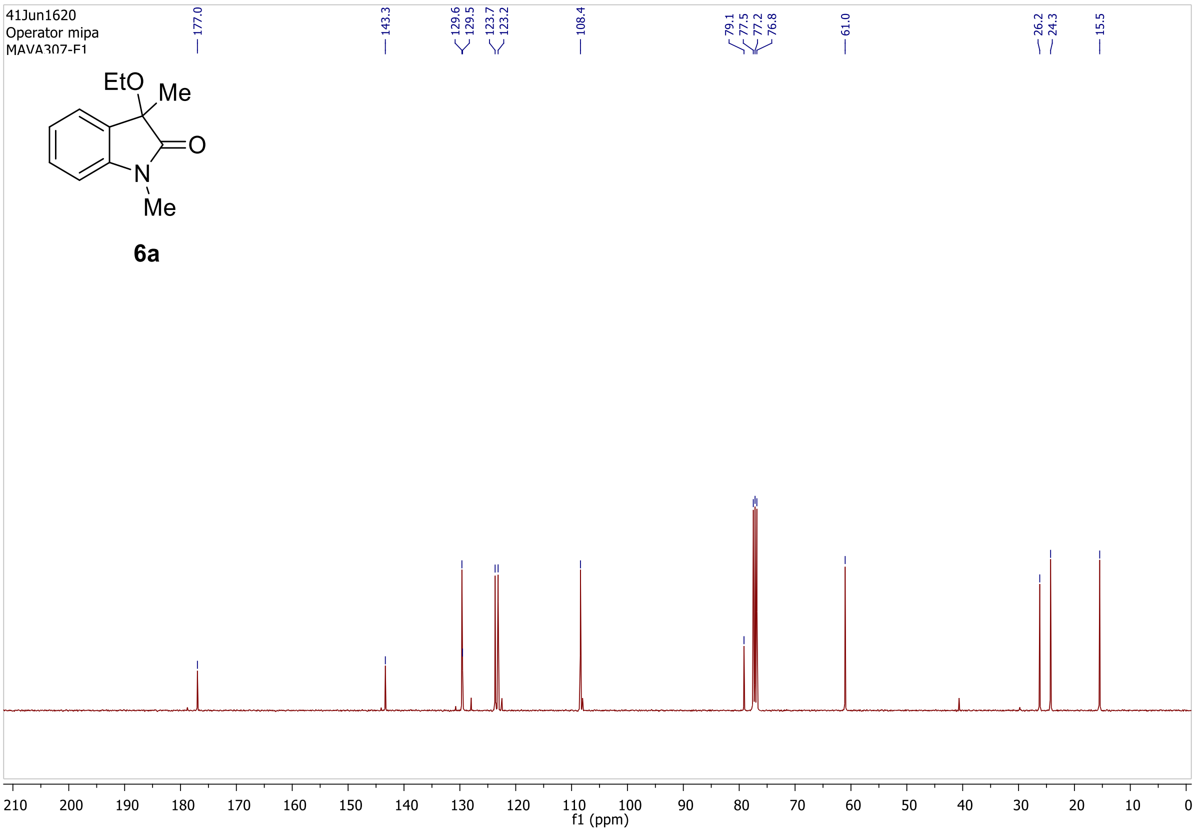

$^{13}C\{^{1}H\}$ NMR in $CDCl_3$ at 100 MHz

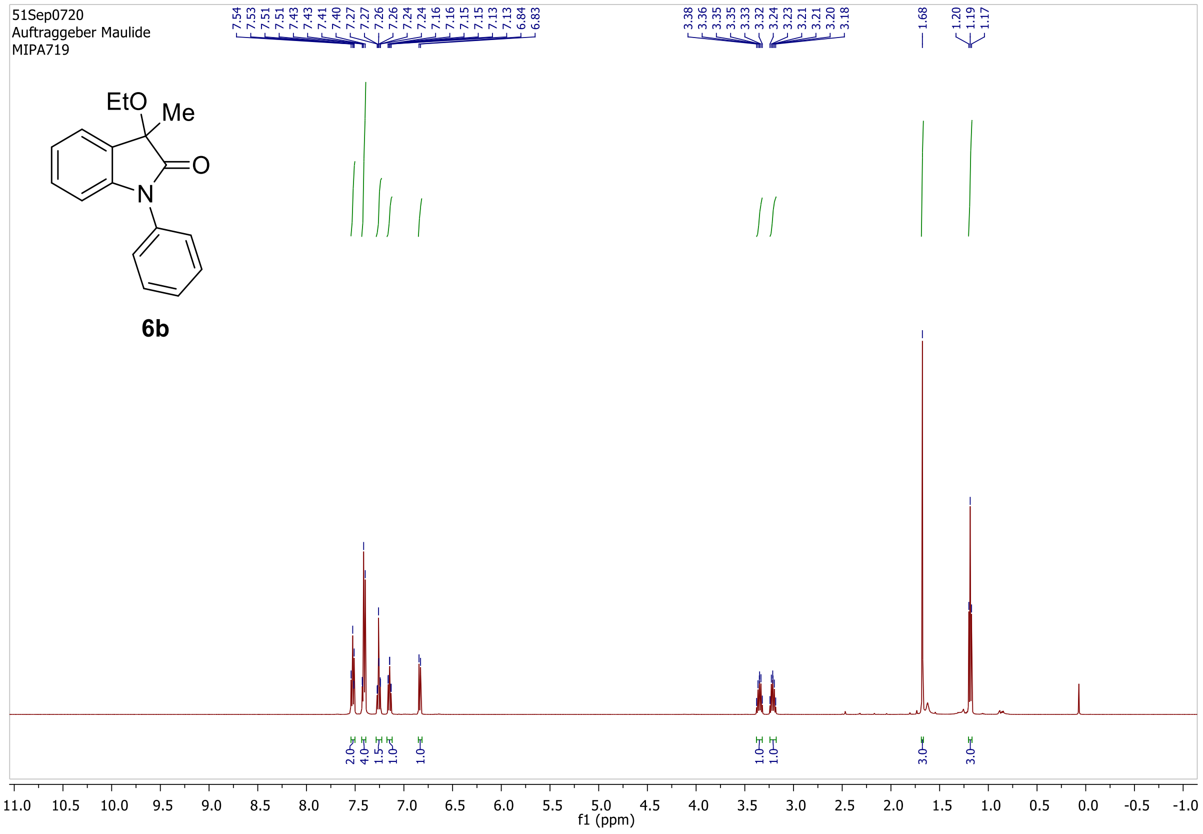

^{1}H NMR in $CDCl_3$ at 500 MHz

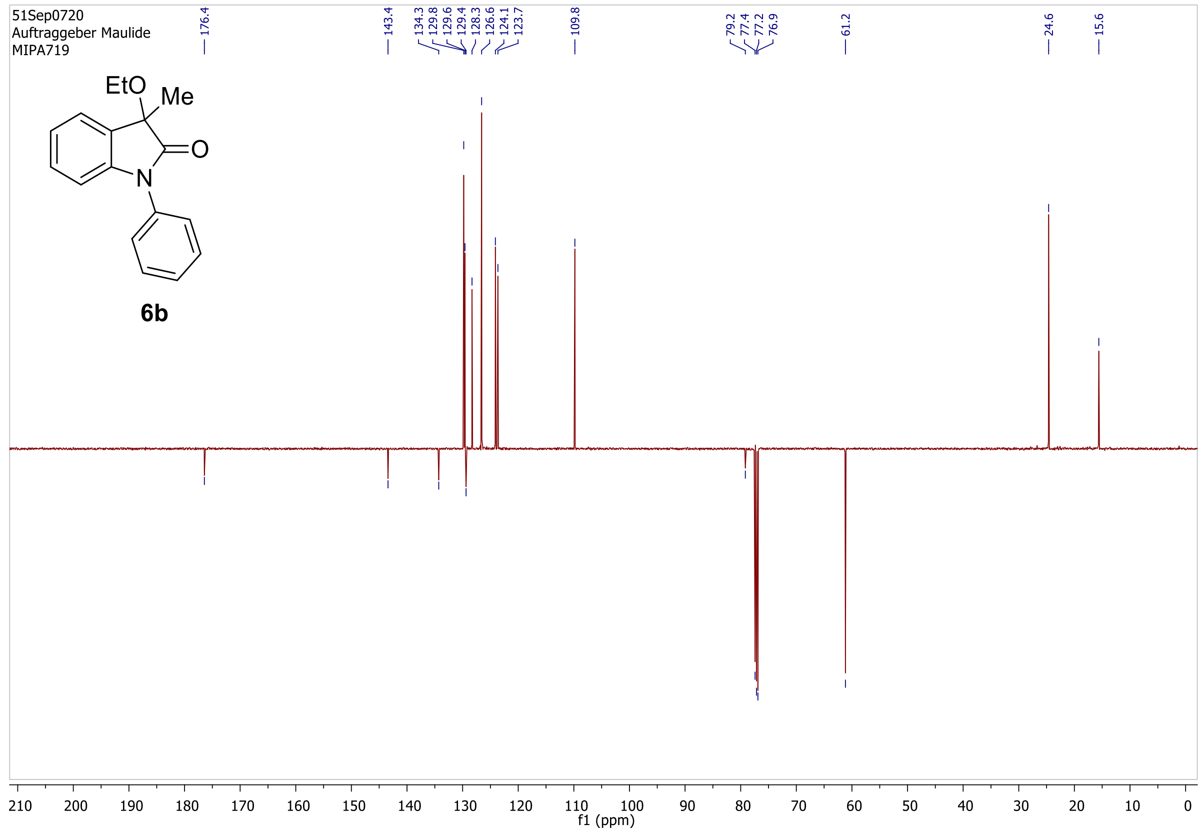

$^{13}C\{^{1}H\}$ NMR in $CDCl_3$ at 126 MHz

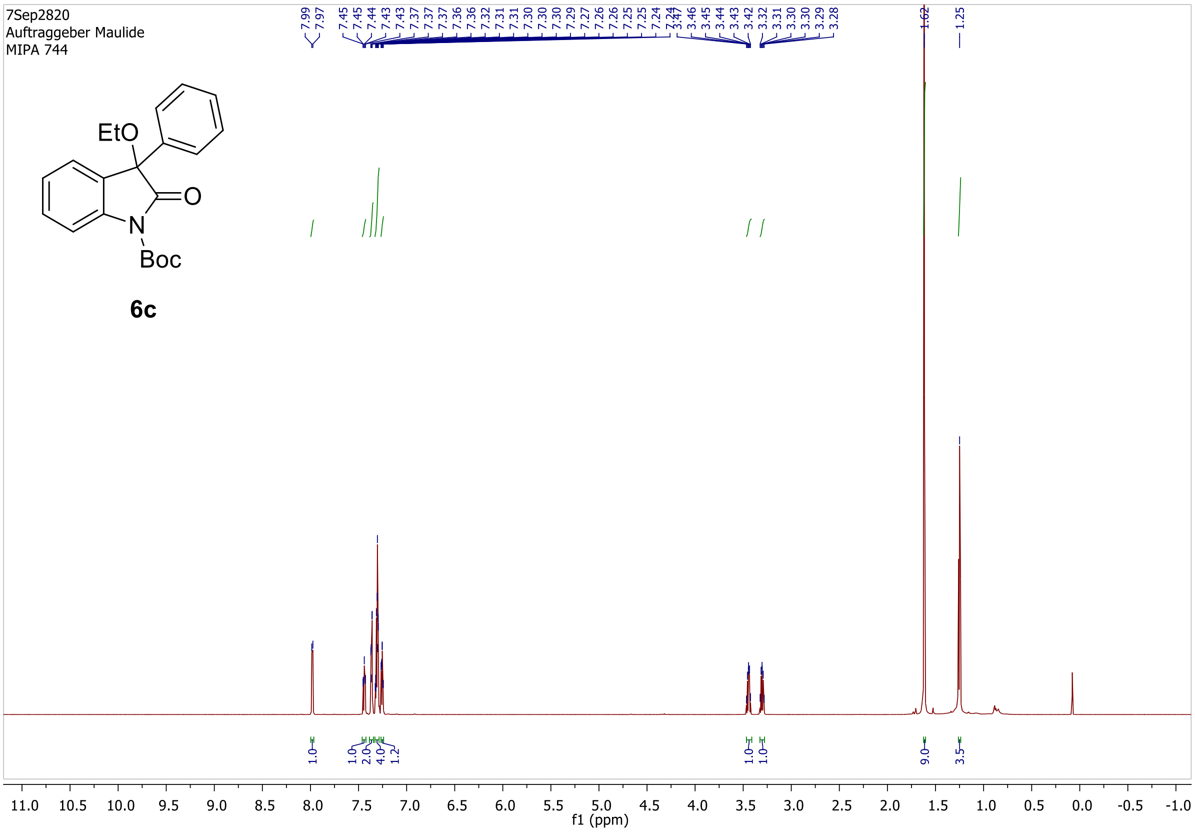

^{1}H NMR in $CDCl_3$ at 700 MHz

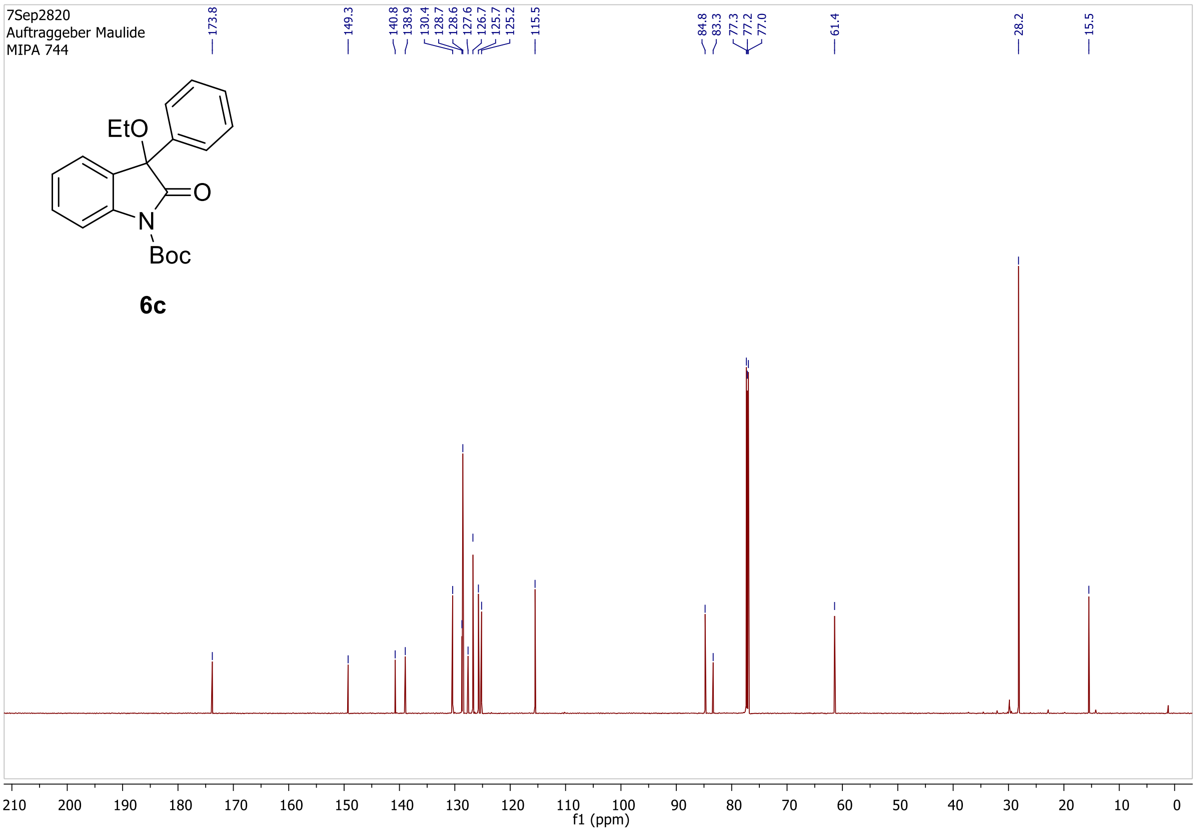

$^{13}C\{^{1}H\}$ NMR in $CDCl_3$ at 176 MHz

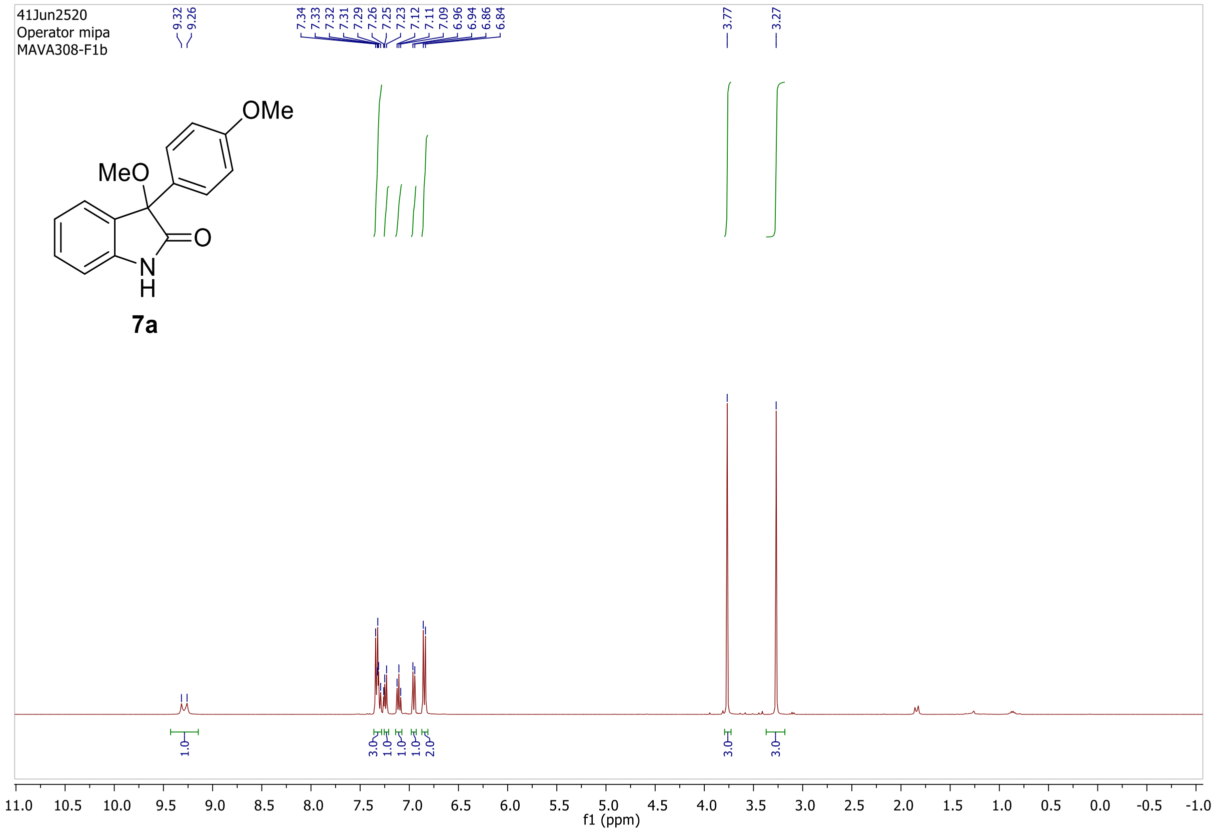

^{1}H NMR in $CDCl_3$ at 400 MHz

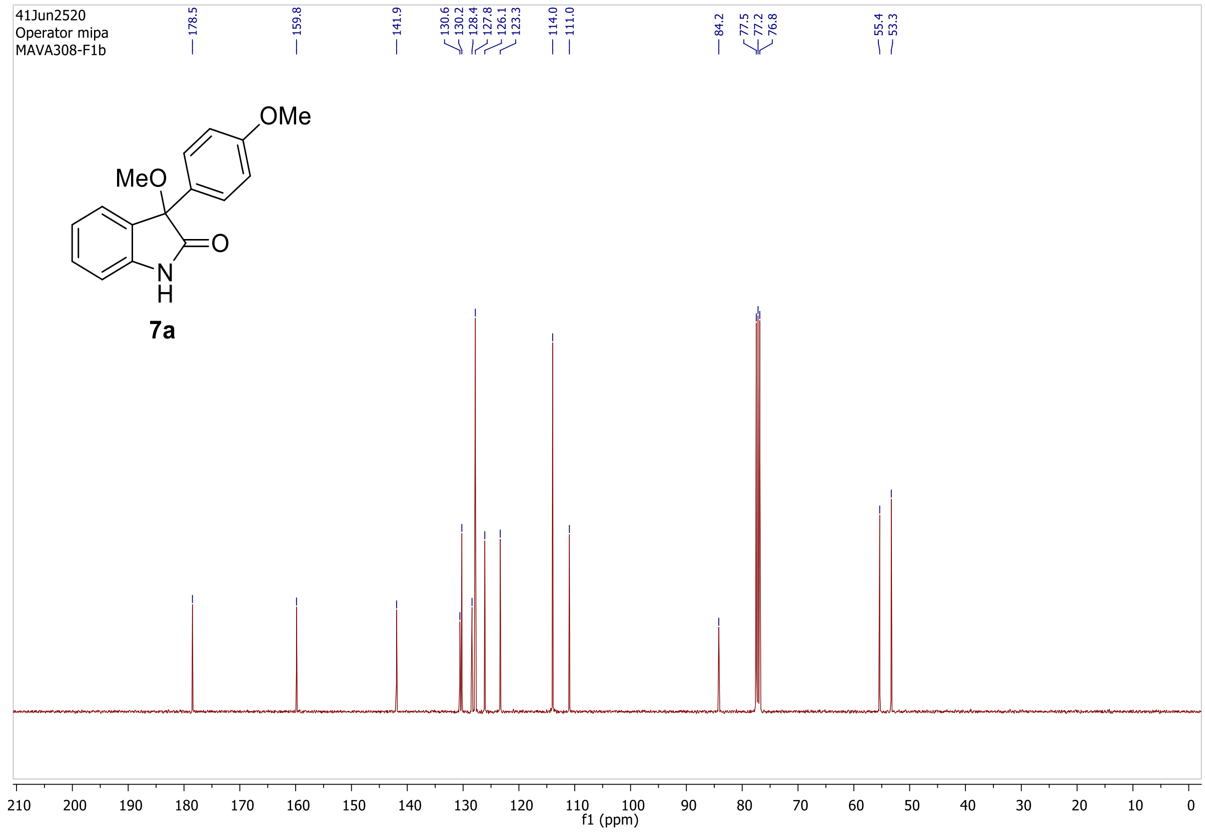

$^{13}C\{^{1}H\}$ NMR in $CDCl_3$ at 100 MHz

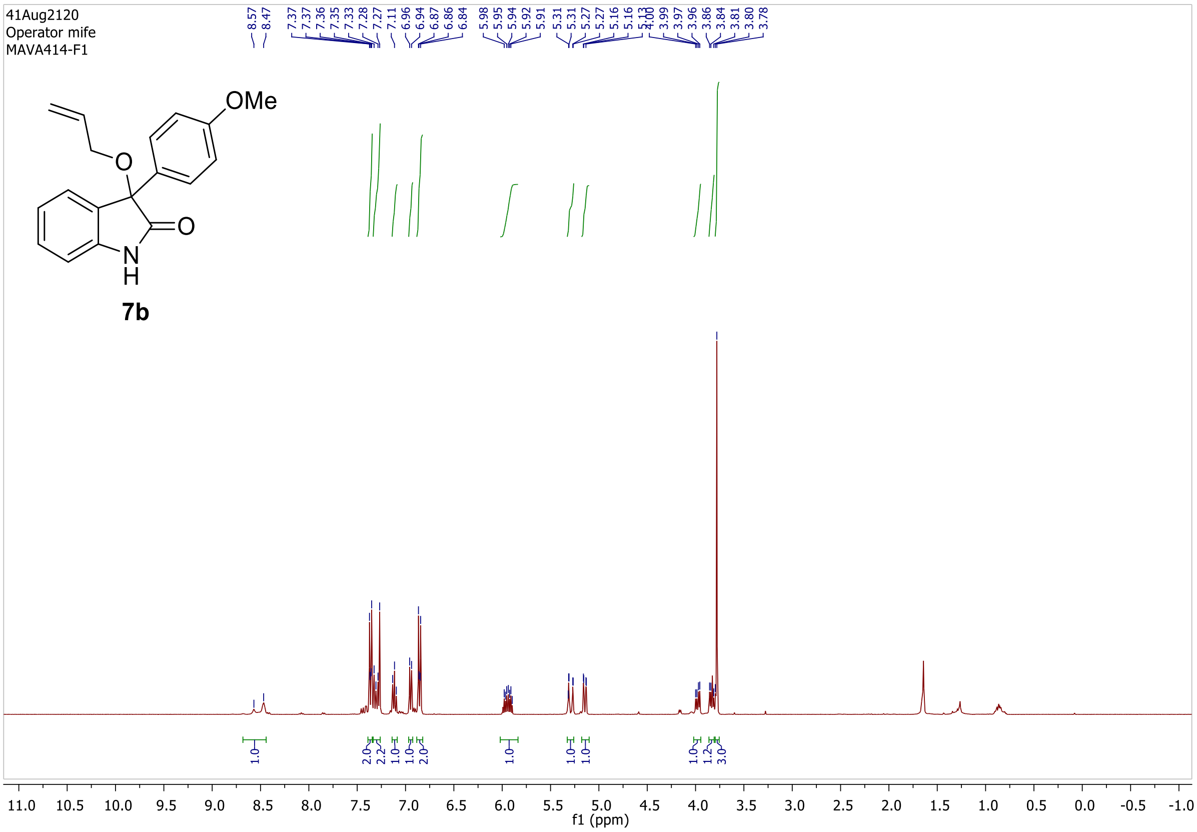

^{1}H NMR in $CDCl_3$ at 400 MHz

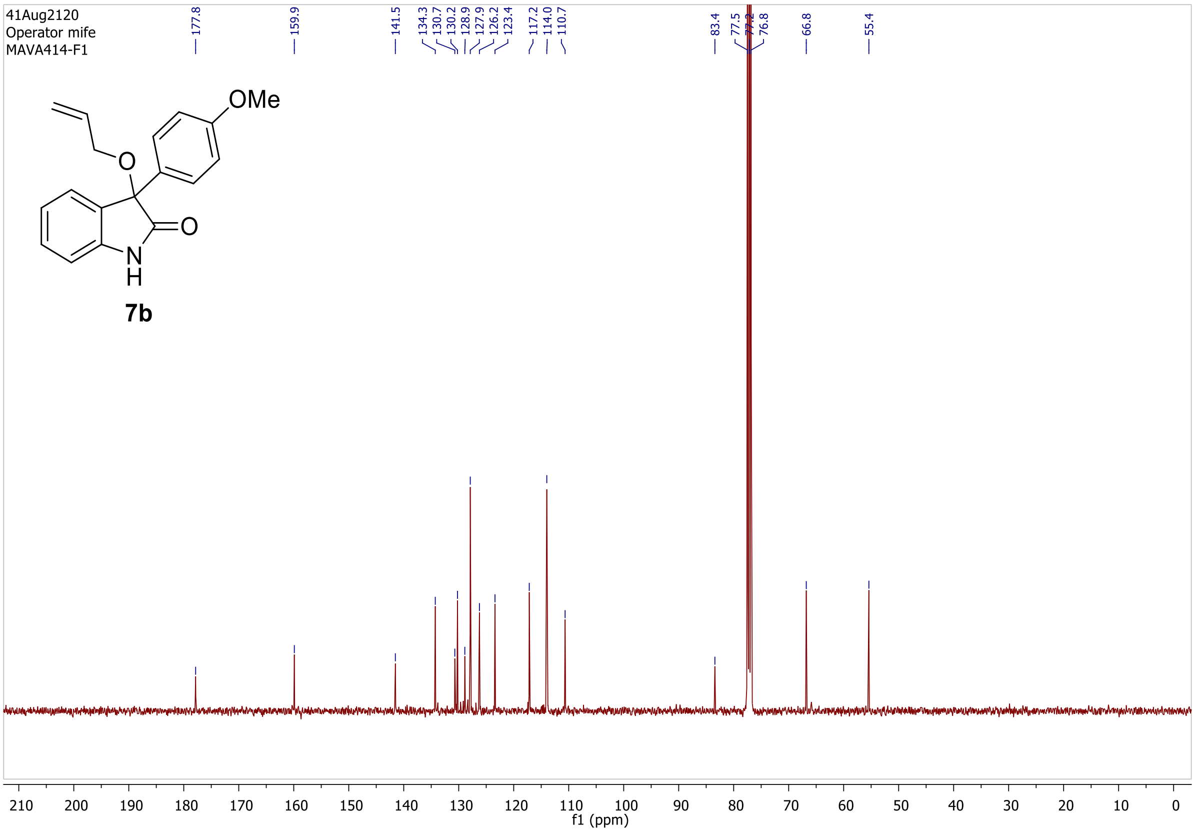

$^{13}C\{^{1}H\}$ NMR in $CDCl_3$ at 100 MHz

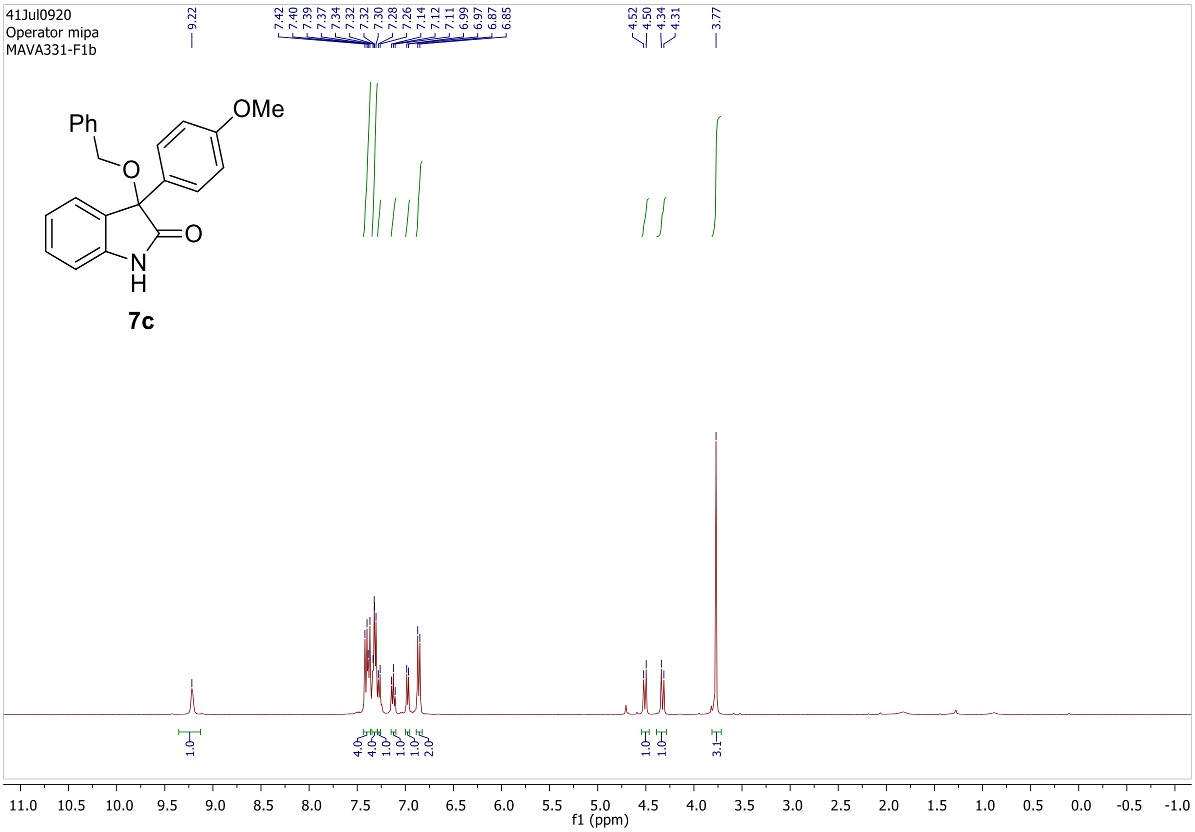

^{1}H NMR in $CDCl_3$ at 400 MHz

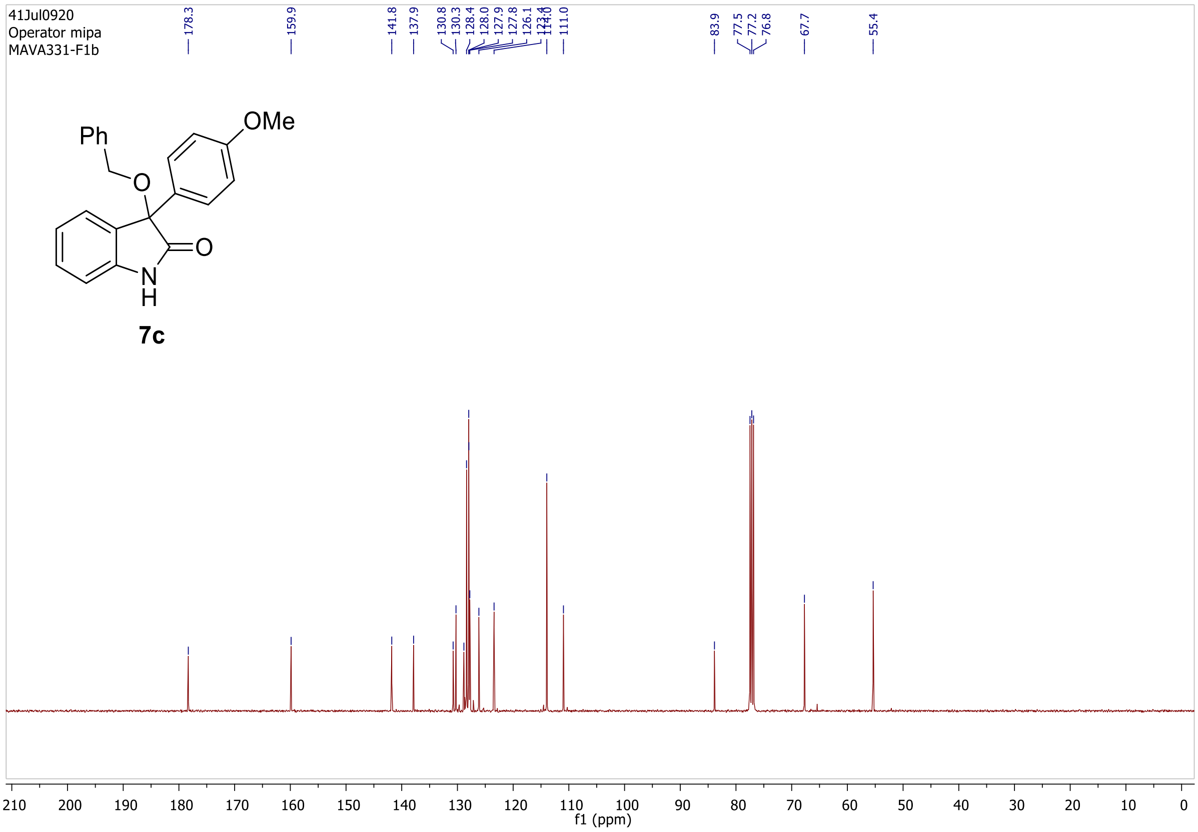

$^{13}C\{^{1}H\}$ NMR in $CDCl_3$ at 100 MHz

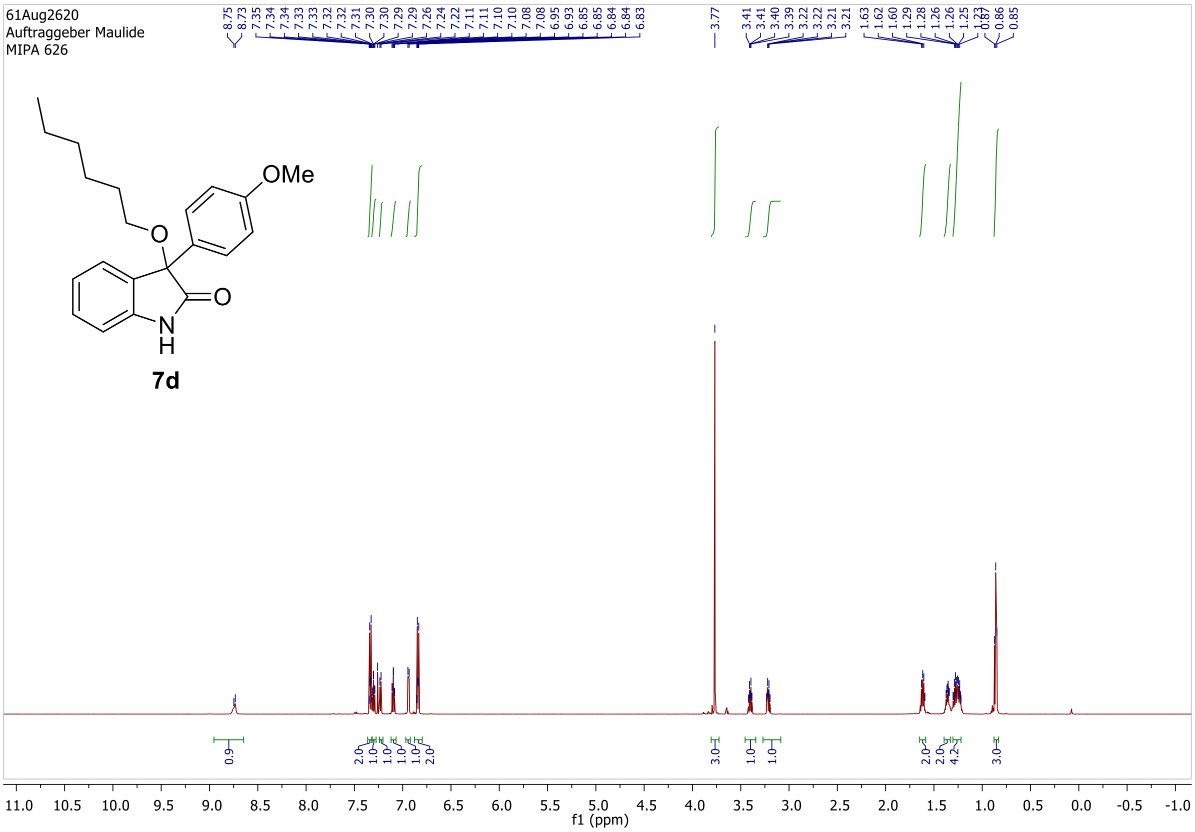

^{1}H NMR in $CDCl_3$ at 600 MHz

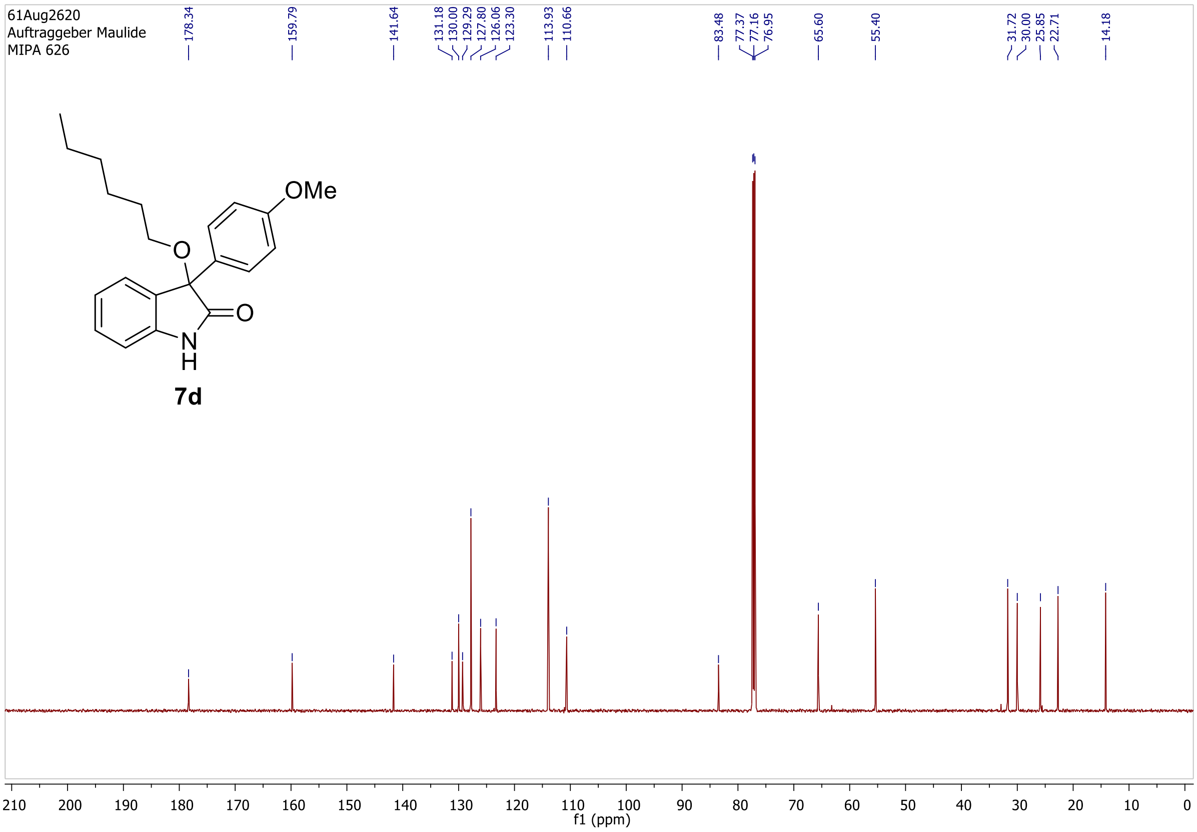

$^{13}C\{^{1}H\}$ NMR in $CDCl_3$ at 150 MHz

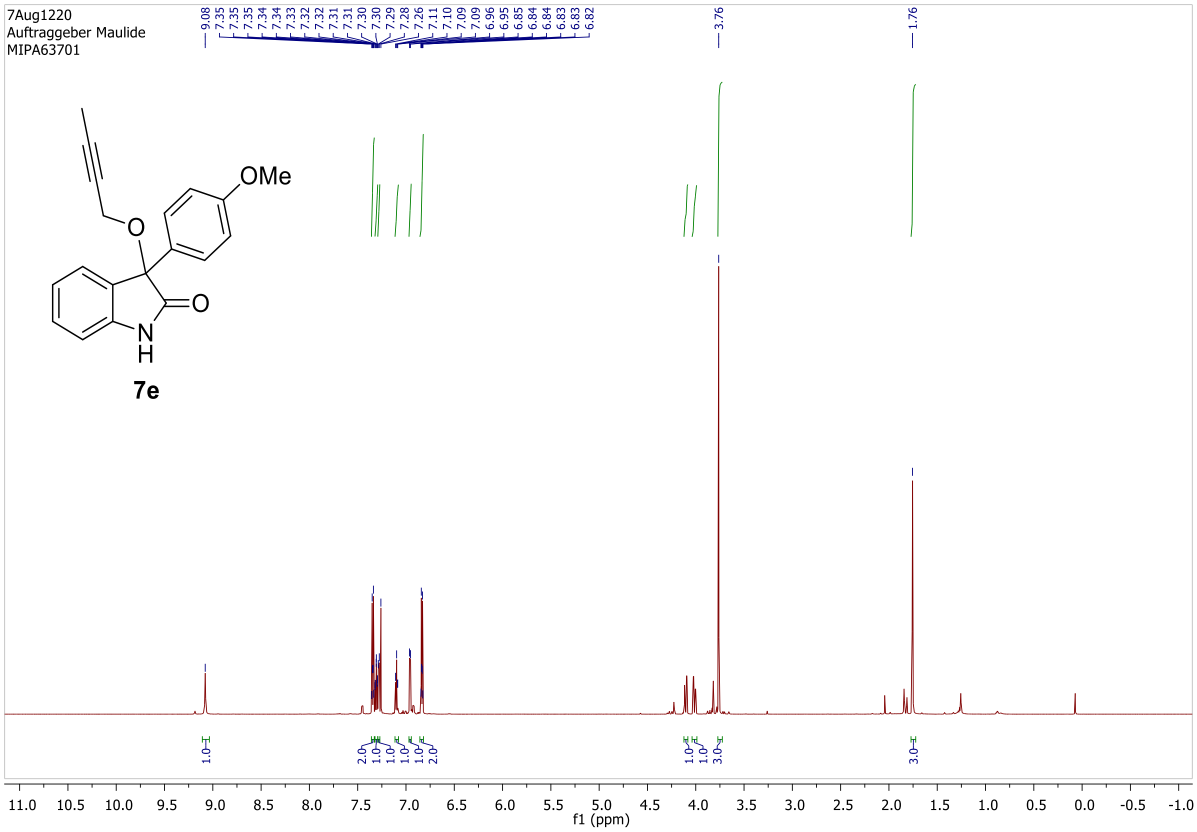

^{1}H NMR in $CDCl_3$ at 700 MHz

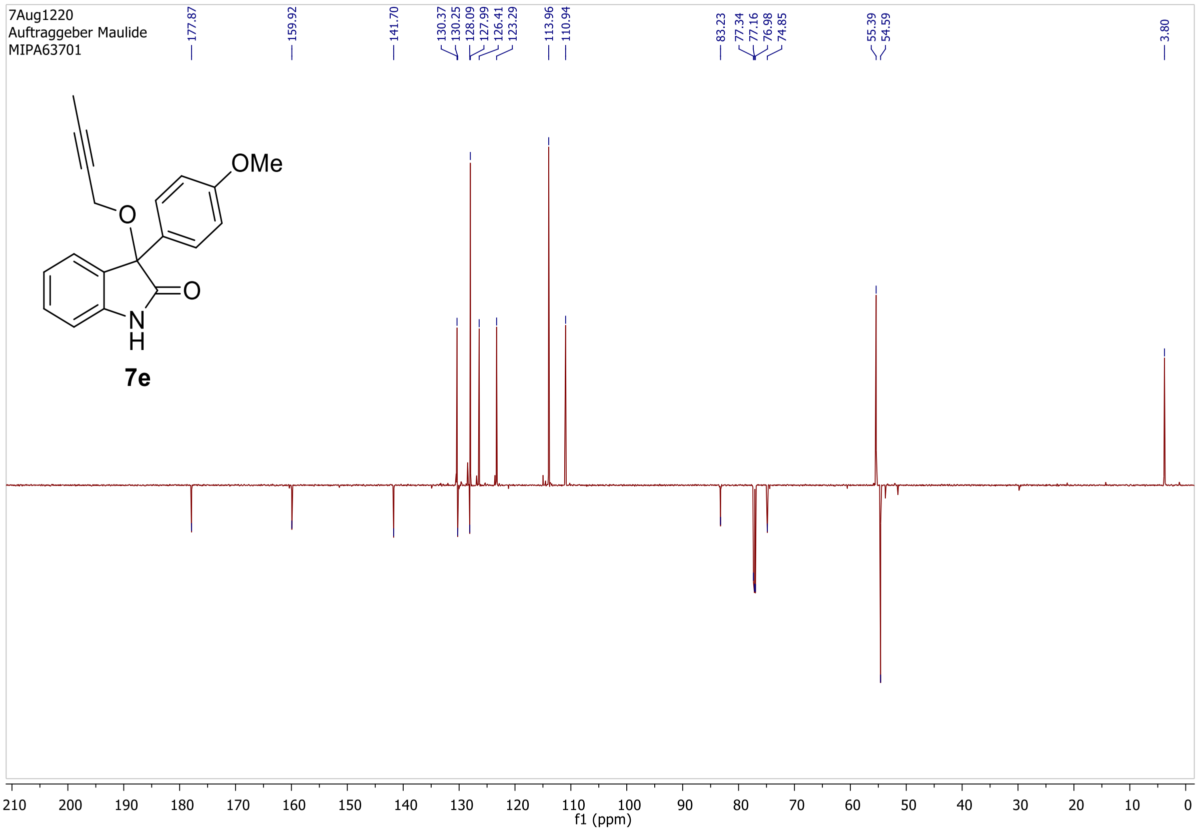

$^{13}C\{^1H\}$ NMR in $CDCl_3$ at 176 MHz

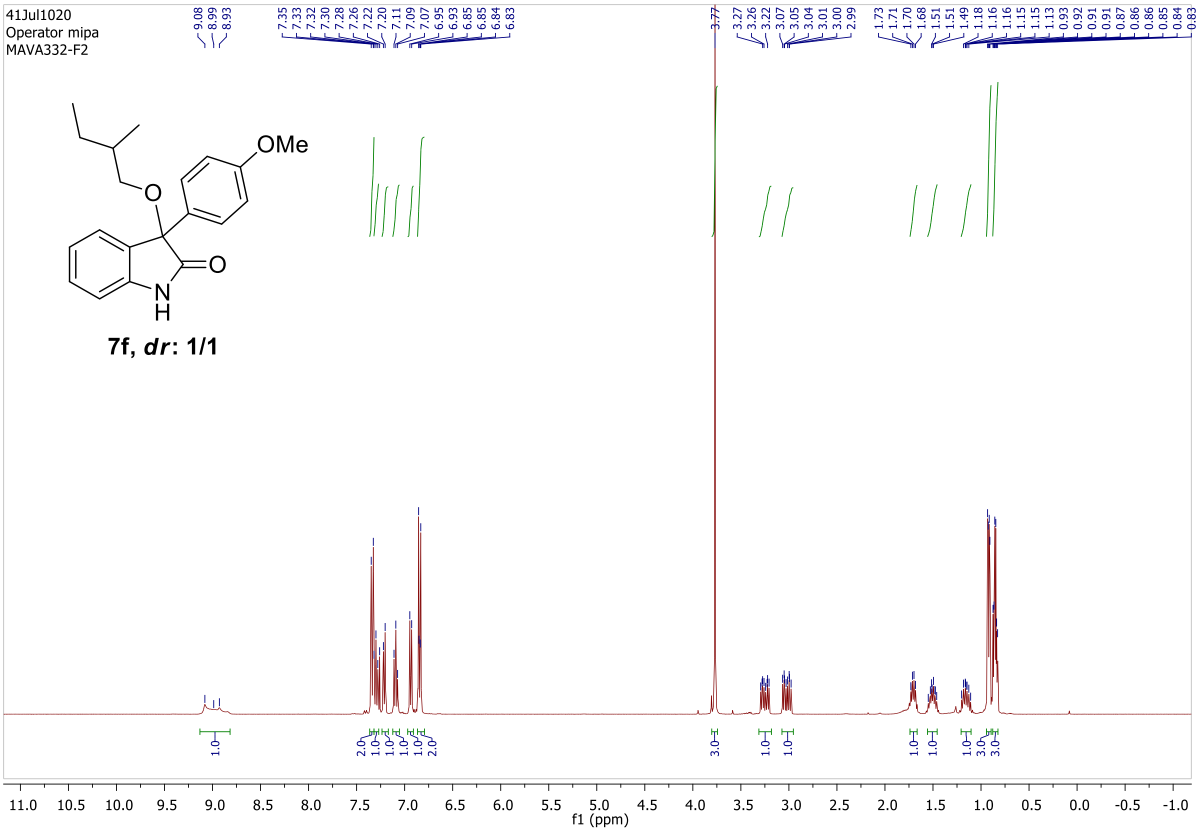

^{1}H NMR in $CDCl_3$ at 400 MHz

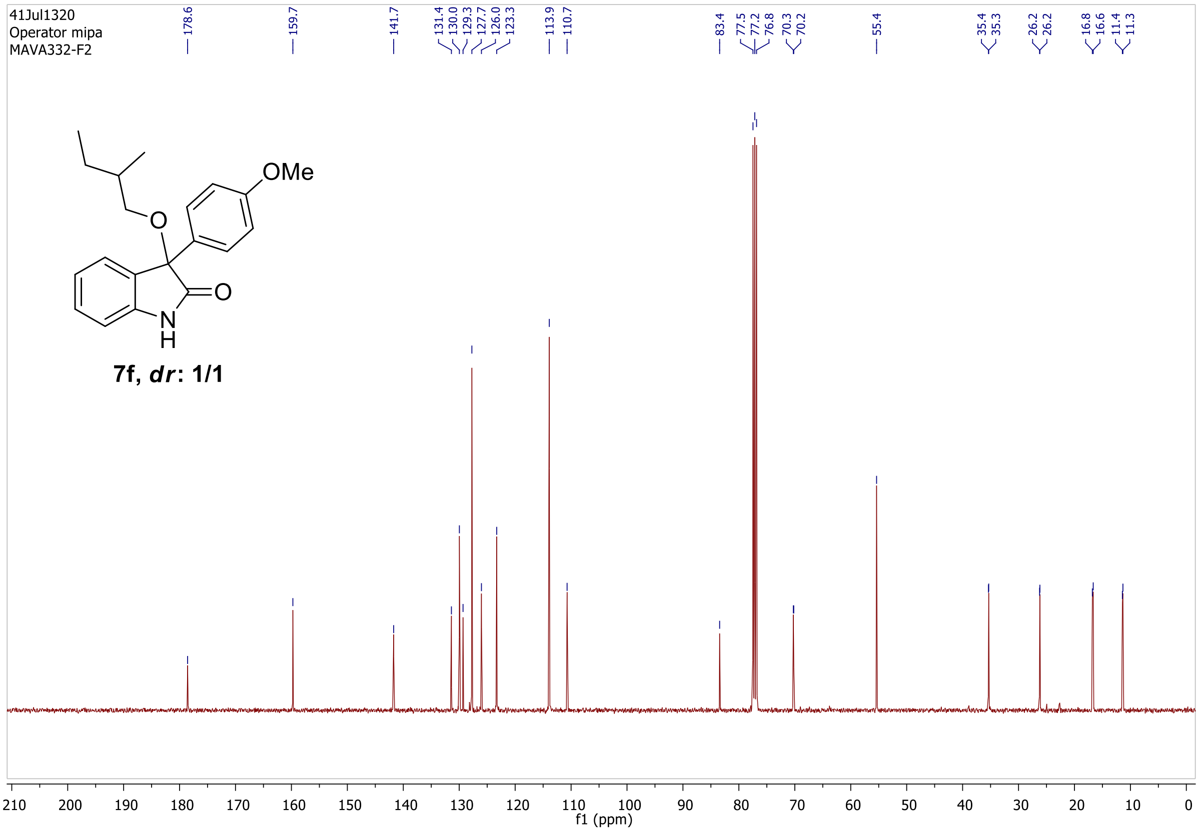

$^{13}C\{^{1}H\}$ NMR in $CDCl_3$ at 100 MHz

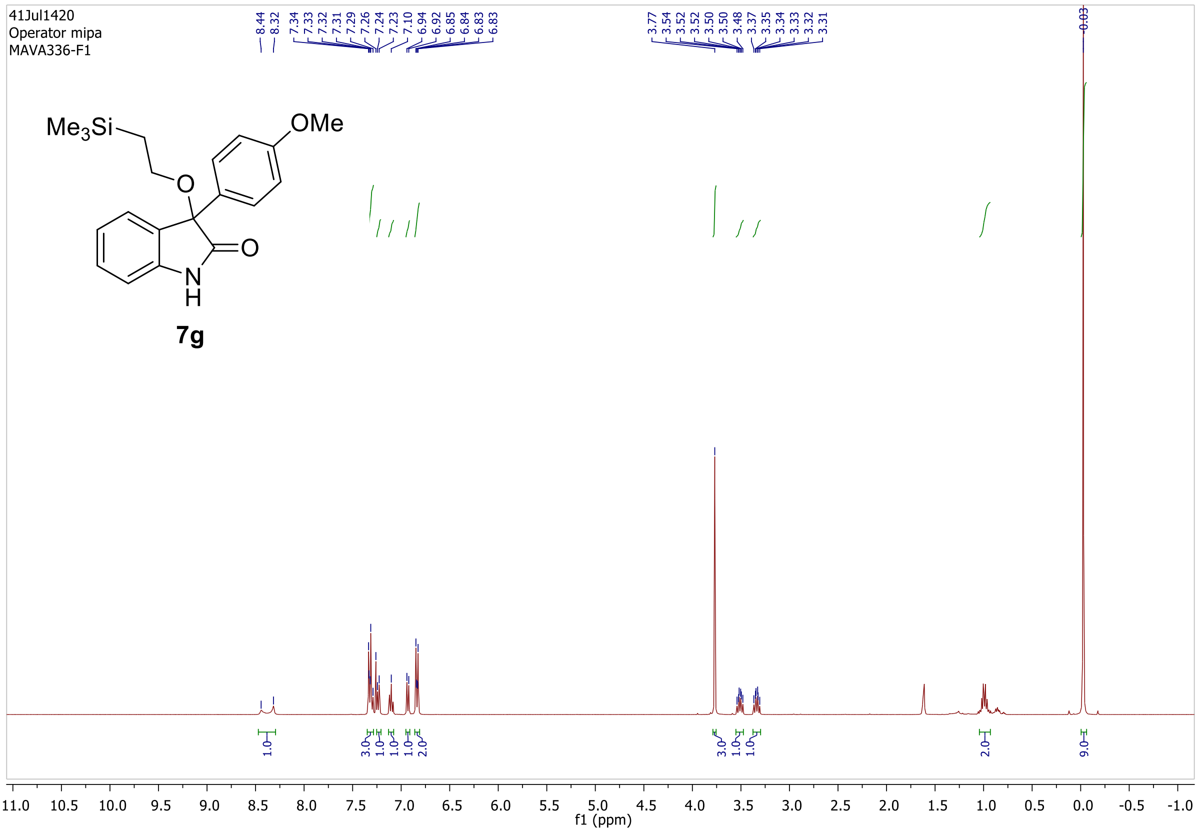

^{1}H NMR in $CDCl_3$ at 400 MHz

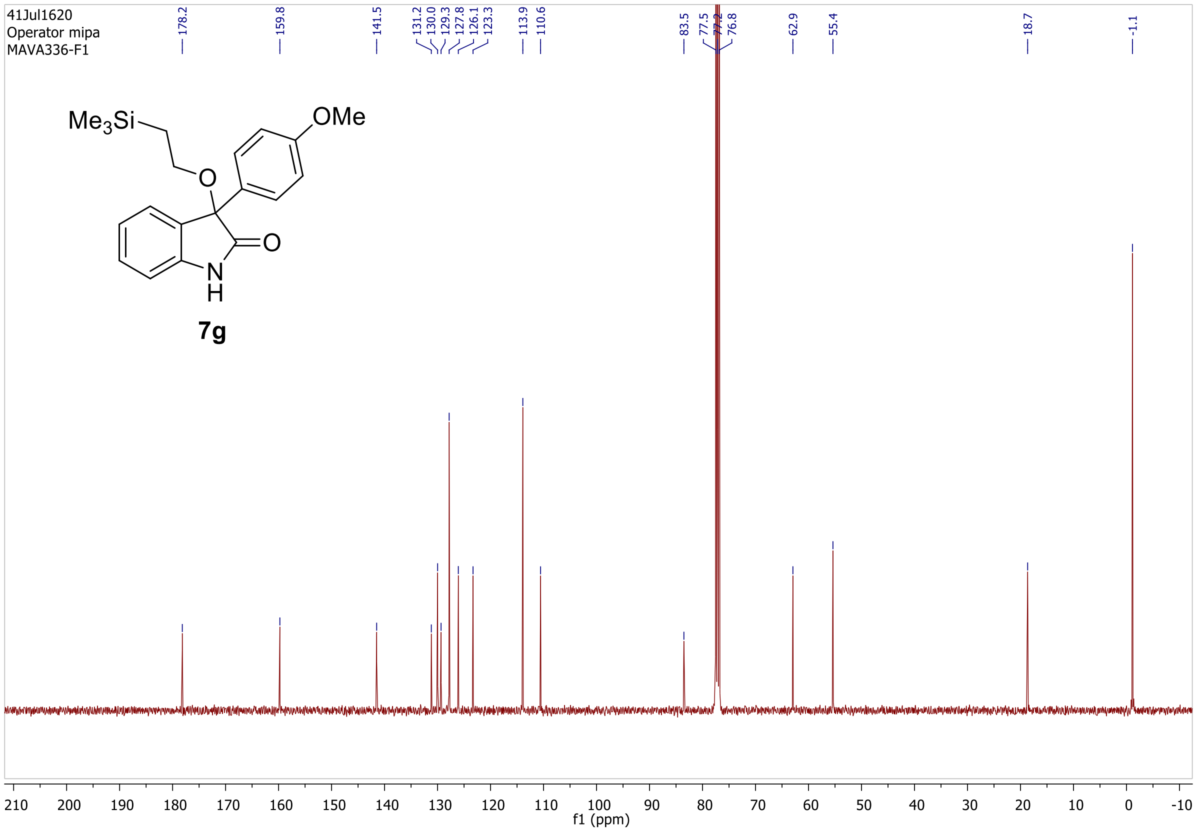

$^{13}C\{^{1}H\}$ NMR in $CDCl_3$ at 100 MHz

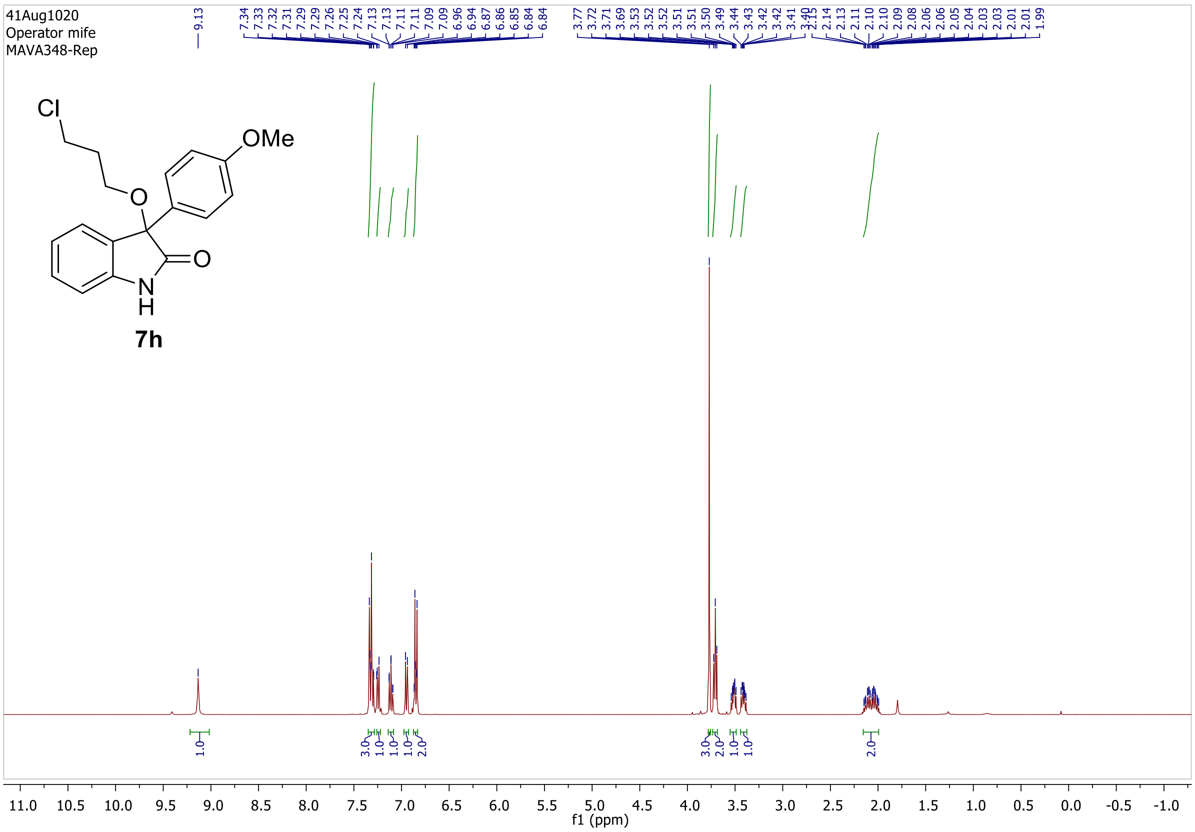

^{1}H NMR in $CDCl_3$ at 400 MHz

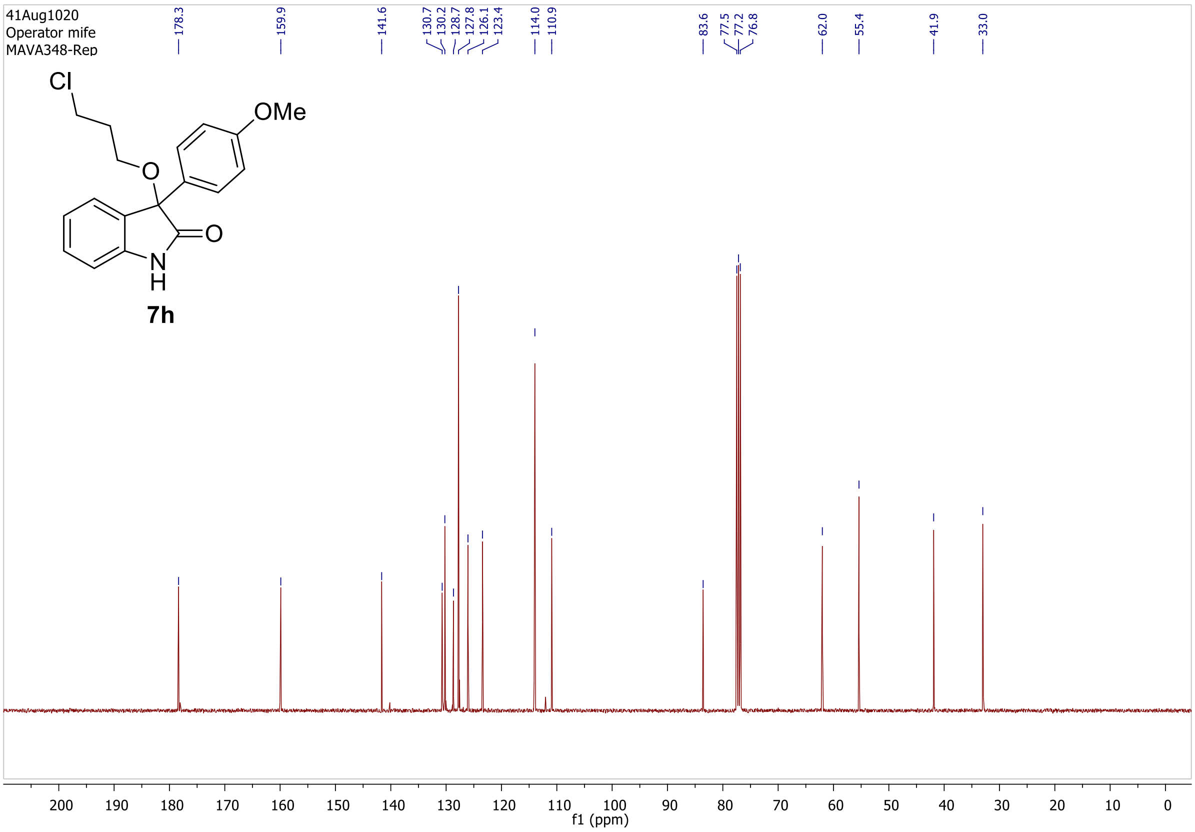

$^{13}C\{^{1}H\}$ NMR in $CDCl_3$ at 100 MHz

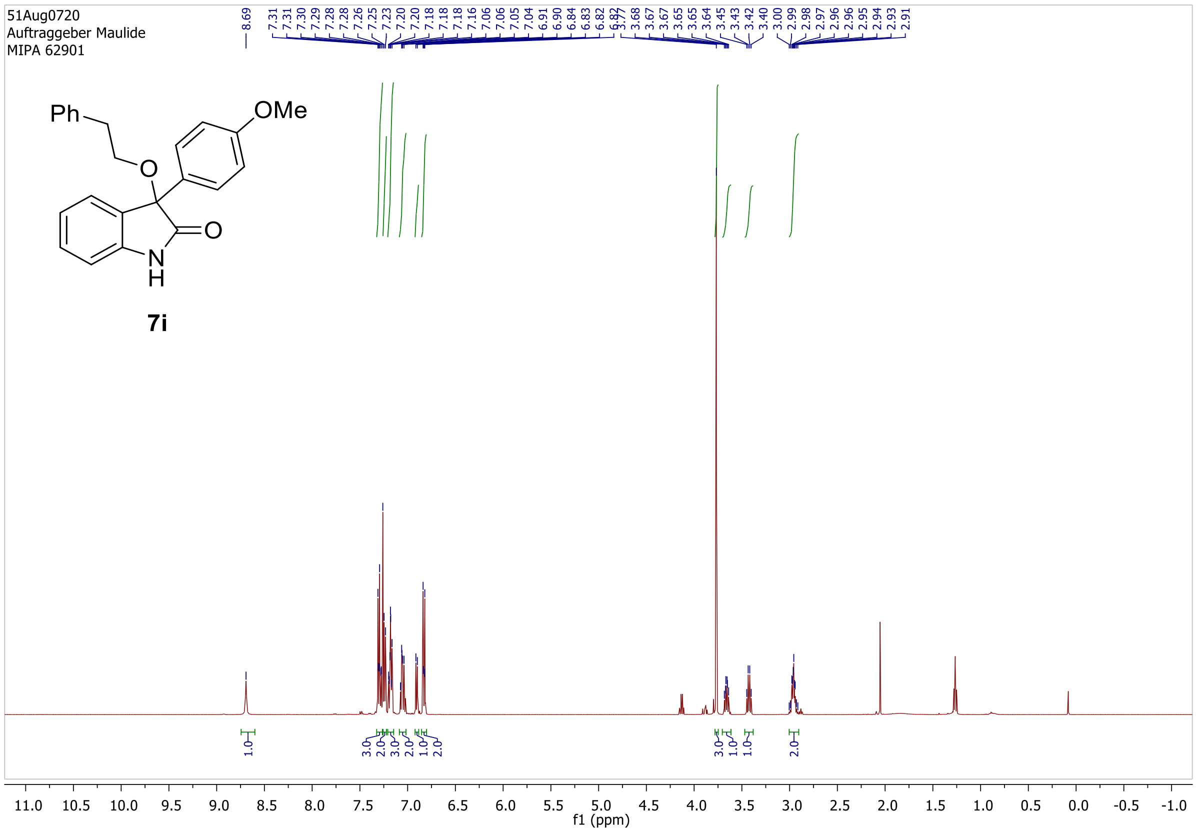

^{1}H NMR in $CDCl_3$ at 500 MHz

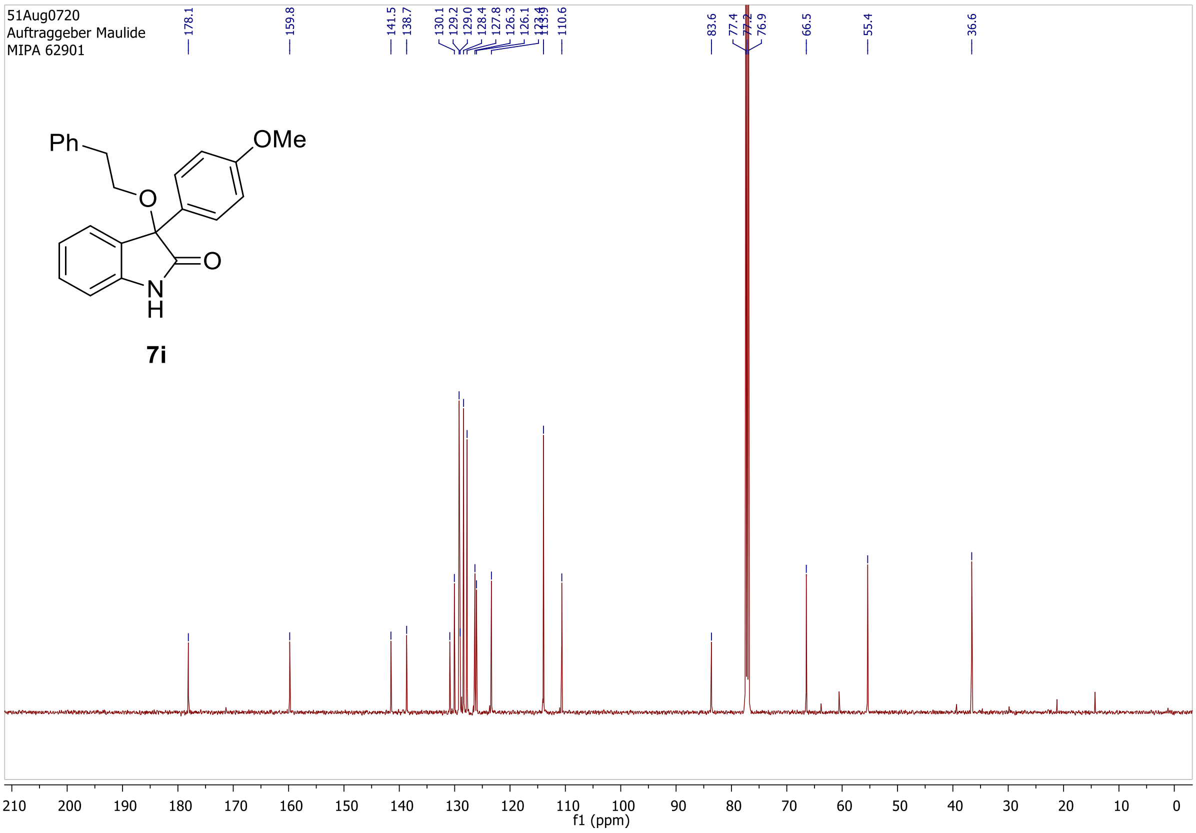

$^{13}C\{^1H\}$ NMR in $CDCl_3$ at 126 MHz

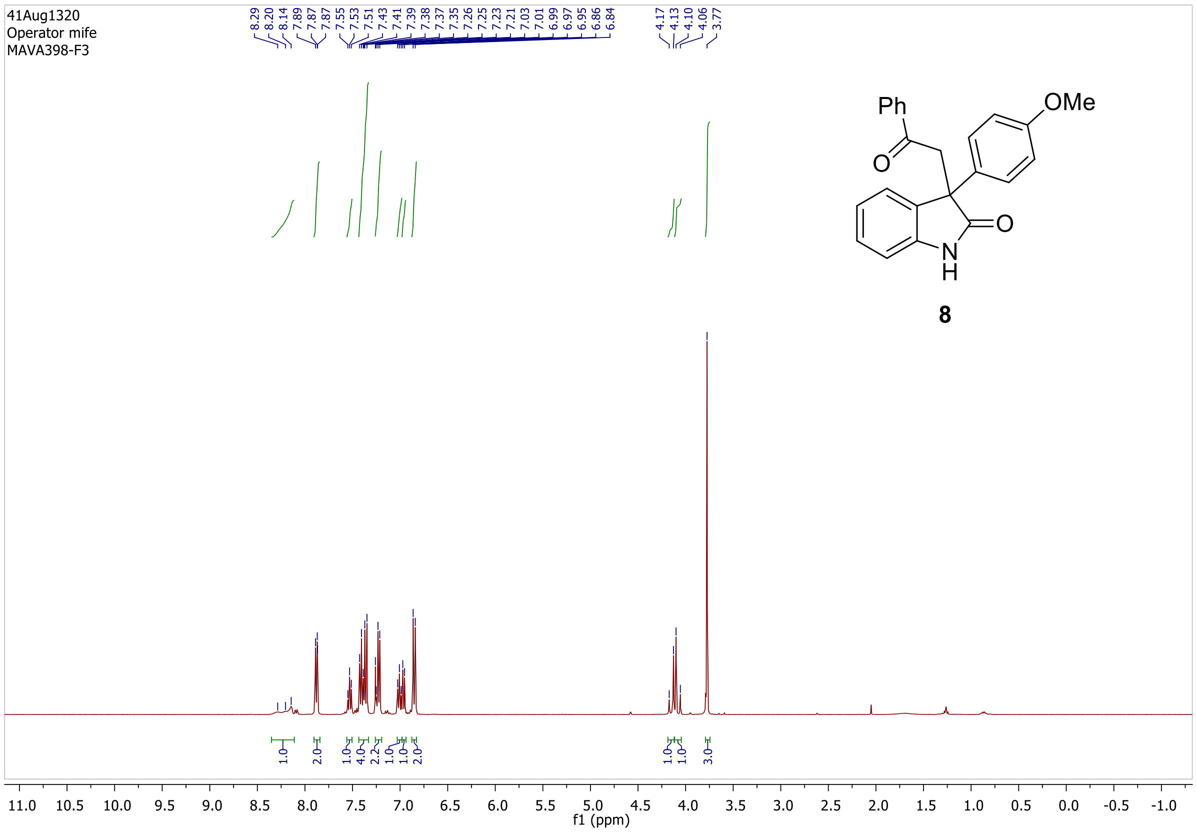

^{1}H NMR in $CDCl_3$ at 400 MHz

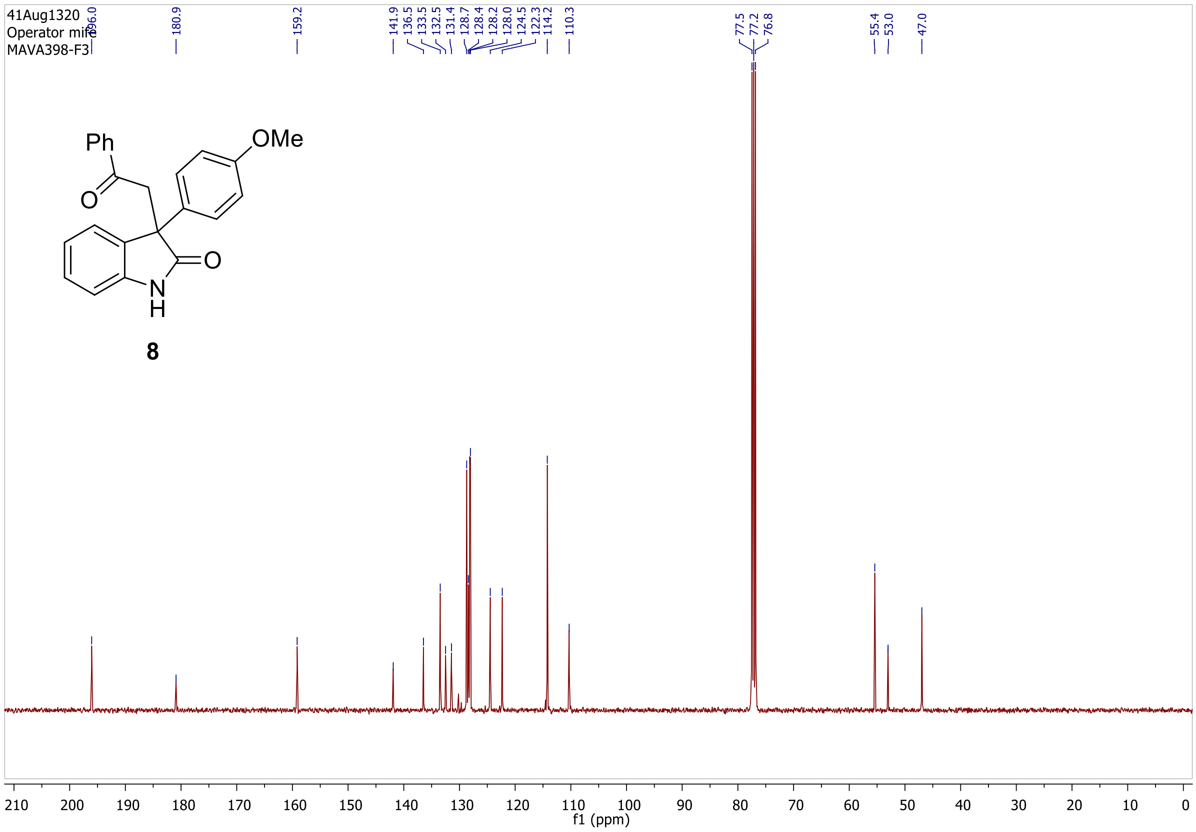

$^{13}C\{^{1}H\}$ NMR in $CDCl_3$ at 100 MHz

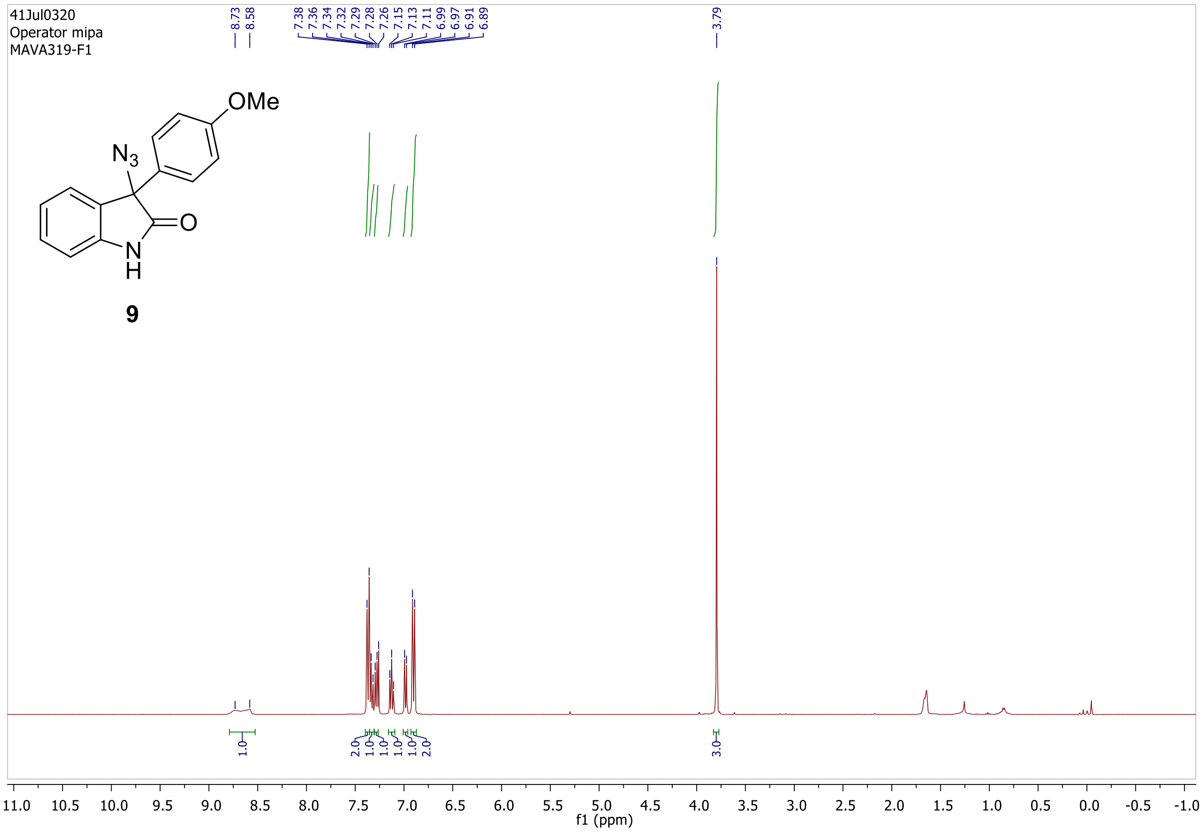

^{1}H NMR in $CDCl_3$ at 400 MHz

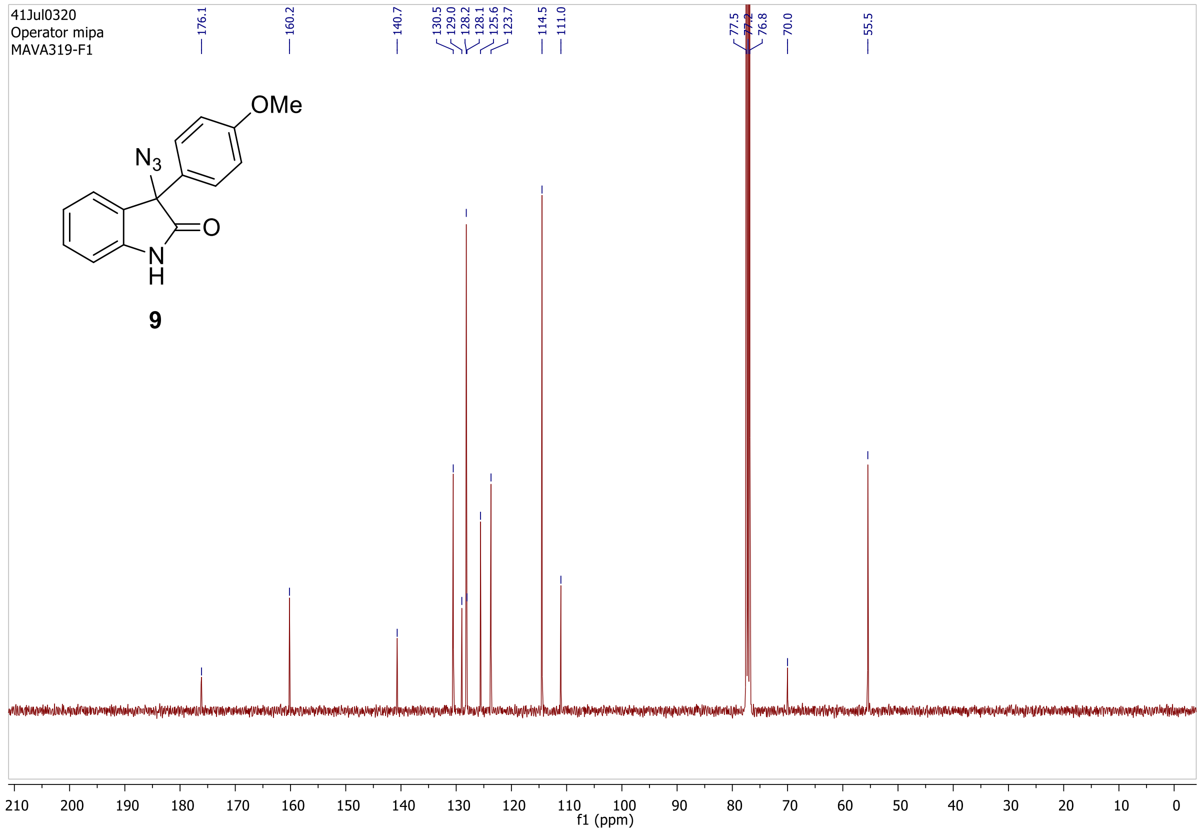

^{13}C{^{1}H} NMR in $CDCl_3$ at 100 MHz

11. References

[1] Trost, B. M.; Zhang, Y. Mo-Catalyzed Regio-, Diastereo-, and Enantioselective Allylic Alkylation of 3-Aryloxindoles. *J. Am. Chem. Soc.* **2007**, 129, 14548–14549.

[2] Jensen, T.; Madsen, R. Ruthenium-Catalyzed Alkylation of Oxindole with Alcohols. *J. Org. Chem.* **2009**, *74*, 3990-3992.

[3] Trost, B. M.; Zhang, Y.; Zhang, T. Direct N-Carbamoylation of 3-Monosubstituted Oxindoles with Alkyl Imidazole Carboxylates. *J. Org. Chem.* **2009**, *74*, 5115-5117.

[4] Dua, T. -P.; Zhu, G. -G.; Zhou, J. A Facile Method for the Synthesis of 3-Alkyloxindole. *Lett. Org. Chem.* **2012**, *9*, 225-232.

[5] Xu, J.; Liang, L.; Zheng, H.; Chi, Y. R.; Tong, R. Green oxidation of indoles using halide catalysis. *Nat. Commun* **2019**, *10*, 4754.

[6] Cao, S. -H.; Zhang, X. -C.; Wei, Y.; Shi, M. Chemoselective Reduction of Isatin-Derived Electron-Deficient Alkenes Using Alkylphosphanes as Reduction Reagents. *Eur. J. Org. Chem.* **2011**, 2668-2672.

[7] Altman, R. A.; Hyde, A. M.; Huang, X.; Buchwald, S. L. Orthogonal Pd- and Cu-Based Catalyst Systems for C- and N-Arylation of Oxindoles. *J. Am. Chem. Soc.* **2008**, *130*, 9613-9620.

[8] Toullec, P. Y.; Jagt, R. B. C.; de Vries, J. G.; Feringa, B. L.; Minnaard, A. J. Rhodium-Catalyzed Addition of Arylboronic Acids to Isatins: An Entry to Diversity in 3-Aryl-3-Hydroxyoxindoles. *Org. Lett.* **2006**, *8*, 2715-2718.

[9] Xiao, Z. -K.; Yin, H. -Y.; Shao, L. -X. N-Heterocyclic Carbene-Palladium(II)-1-Methylimidazole Complex Catalyzed α-Arylation of Oxindoles with Aryl Chlorides and Aerobic Oxidation of the Products in a One-Pot Procedure. *Org. Lett.* **2013**, *15*, 1254-1257.

[10] Gade, A. B.; Bagle, P. N.; Shinde, P. S.; Bhardwaj, V.; Banerjee, S.; Chande, A.; Patil, N. T. Catalytic Enantioselective 1,3-Alkyl Shift in Alkyl Aryl Ethers: Efficient Synthesis of Optically Active 3,3′-Diaryloxindoles. *Angew. Chem. Int. Ed.* **2018**, *57*, 5735-5739.

[11] Zhang, Z.; Smal, V.; Retailleau, P.; Voituriez, A.; Frison, G.; Marinetti, A.; Guinchard, X. Tethered Counterion-Directed Catalysis: Merging the Chiral Ion-Pairing and Bifunctional Ligand Strategies in Enantioselective Gold(I) Catalysis. *J. Am. Chem. Soc.* **2020**, *142*, 3797-3805.

[12] Xia, J. -T.; Hu, X. -P. Copper-Catalyzed Asymmetric Propargylic Alkylation with Oxindoles: Diastereo- and Enantioselective Construction of Vicinal Tertiary and All-Carbon Quaternary Stereocenters. *Org. Lett.* **2020**, *22*, 1102-1107.

[13] Polidano, K.; Allen, B. D. W.; Williams, J. M. J.; Morrill, L. C. Iron-Catalyzed Methylation Using the Borrowing Hydrogen Approach. *ACS Catal.* **2018**, *8*, 6440-6445.

[14] Wang, H. -L.; Li, Y. -M.; Wang, G. -W.; Zhang, H.; Yang, S. -D. Scandium(III) Triflate Catalyzed Direct Cyclization of Ketoamides for the Synthesis of 3-Hydroxy-2-Oxindoles. *Asian J. Org. Chem.* **2013**, *2*, 486-490.

[15] Liu, X. -L.; Yue, J.; Chen, S.; Liu, H. -H.; Yang, K. -M.; Feng, T. -T.; Zhou, Y. Thermal-mediated catalyst-free heterolytic cleavage of 3-halooxindoles: rapid access to 3-functionalized-2-oxindoles. *Org. Chem. Front.* **2019**, *6*, 256-262.

[16] Hino, T.; Endo, M.; Tonozuka, M.; Hashimoto, Y.; Nakagawa, M. Bromination of 2-Bromo, 2-Ethylthio, and 2-Ethylsulfonylindoles with N-Bromosuccinimide. Isolation and Reactions of 1-Bromoindoles and 3-Bromoindolenines. *Chem. Pharm. Bull.* **1977**, *25*, 2350-2358.

[17] Escolano, C.; Vallverdú, L.; Jones, K. Reaction of Indolin-2-ones with Cerium(IV) Ammonium Nitrate. *Tetrahedron* **2002**, *58*, 9541-9545.
